# Synthesis of *N*-acetyl diazocine derivatives via cross-coupling reaction

**DOI:** 10.3762/bjoc.21.36

**Published:** 2025-03-04

**Authors:** Thomas Brandt, Pascal Lentes, Jeremy Rudtke, Michael Hösgen, Christian Näther, Rainer Herges

**Affiliations:** 1 Otto Diels Institute for Organic Chemistry, Kiel University, Otto-Hahn-Platz 4, 24118 Kiel, Germany; 2 Institute for Inorganic Chemistry, Kiel University, Max-Eyth-Straße 2, 24118 Kiel, Germany

**Keywords:** cross-coupling, diazocine, *N*-acetyl diazocine, photoisomerization, photoswitch, thermal relaxation

## Abstract

Diazocines are photoswitches derived from azobenzenes by bridging the two phenyl rings in *ortho* position with a CH_2_CH_2_ group forming an eight membered (diazocine) ring. Diazocine is superior to most azobenzenes in almost all photophysical properties (switching efficiency, quantum yield, wavelengths etc.). The biggest advantage, especially in photopharmacology and when used in photoswitchable materials, is the inverted thermodynamic stability of the two switching states (isomers). The *Z* isomer is more stable than the *E* form. However, one disadvantage that it shares with the frequently used azobenzene is that the switching efficiency decreases sharply with increasing water content in the solvent. In a recently published paper, we reported that replacing one CH_2_ group in the bridge with NCOCH_3_ not only confers intrinsic water solubility, but also largely eliminates the problem of reduced switching efficiency in aqueous solutions. In order to investigate the chemistry of this promising photoswitch and to unlock further applications, we now investigate strategies for the synthesis of derivatives, which are based on cross-coupling reactions. Fourteen vinyl-, aryl-, cyano-, and amino-substituted diazocines were prepared via Stille, Suzuki, and Buchwald–Hartwig reactions. X-ray structures are presented for derivatives **1**, **2** and **7**.

## Introduction

**Figure 1:**
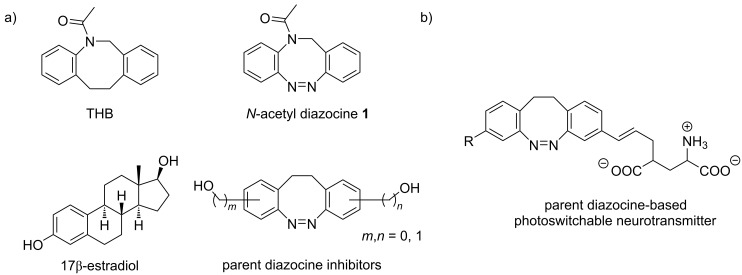
a) Structural similarity of *N*-acetyl diazocine **1** with known 17βHSD3-inhibitor tetrahydrodibenzazocine (THB) [17] and parent diazocine with steroid scaffolds [18]. b) Parent diazocine attached to glutamate to form a photoswitchable neurotransmitter [10].

Diazocines are frequently used photoswitches with superior photophysical properties. The parent ethylene-bridged diazocine shows excellent switching photoconversion between the *Z* and the *E* configurations (Γ(*Z* → *E*)_385nm_ = 92% and Γ(E → Z)_520nm_ > 99% in *n*-hexane) due to well-separated n–π*-transitions in the visible part of the electromagnetic spectra [1]. Moreover, the ethylene bridge creates a cyclic 8-membered core, inverting the thermodynamically stability in favor of the *Z* boat conformation compared to parent azobenzene, which has a stable *E* configuration [1–4]. Preceding studies including azobenzene-based photopharmacophores showed that, in most cases, the sterically demanding *Z* configuration is biologically inactive, while the stretched *E* configuration is biologically active [5–7]. Because of the inverted thermodynamic stability compared to azobenzene, the stable *Z* configuration of the diazocine can be administered and subsequently activated with light at the site of illness with high spatiotemporal resolution. Thus, collateral damage in the surrounding healthy tissue can be avoided. In addition, the quantitative thermal back-isomerization from the active *E* to the inactive *Z* configuration prevents contamination and accumulation in the environment after excretion [1,6–7]. These superior properties of diazocines have been exploited in several applications such as the control of protein folding by implementation as cross-linkers between protein side chains [8] or in peptide backbones [9], as photoswitchable neurotransmitters [10–11] or as switching units for potential dependent potassium channels [12]. Compared to the *Z* → *E* conversion rate of 92% (in *n*-hexane) of the parent diazocine the conversion in water/DMSO mixtures is decreasing with increasing water concentration (73% in water/DMSO 9:1) [8–12]. Moreover, the parent diazocine is insoluble in water (precipitation in water/DMSO > 9:1). Substitution with polar substituents such as CH_2_NH_2_ provides water solubility, however, it does not restore the high *Z* → *E* conversion rates of the parent system in organic solvents, which is a disadvantage, since biochemical reactions usually take place in aqueous environments [13]. The substitution of one CH_2_ group in the CH_2_CH_2_ bridge by N–C(=O)–CH_3_ leads to an intrinsic water solubility of the *N*-acetyl diazocine **1** (Figure 1) [3]. Furthermore the photoconversion of **1** shows no significant drop in pure water in contrast to the solubilized parent diazocine. These superior properties make *N*-acetyl diazocine **1** an ideal candidate for application in the field of photopharmacology especially in aqueous environments [13–14]. The same applies for the quantum yields. While the quantum yields Φ*_Z_*_→_*_E_* and Φ*_E_*_→_*_Z_* of the parent diazocine drop significantly in aqueous media, the corresponding quantum yields of the *N*-acetyl diazocine stay the same or even slightly improve (Table 1). In general, the quantum yields of parent diazocine and its’ nitrogen bridged derivative exceed the quantum yields of other molecular photoswitches like azobenzenes, spiropyranes and diarylethenes in organic solvents [3,13,15].

**Table 1:** Quantum yields of *N*-acetyl diazocine **1** in organic and aqueous media compared to parent diazocine [3,13].

	parent diazocine		*N*-acetyl diazocine	
solvent	Φ*_Z_*_→_*_E_* (385 nm)	Φ*_E_*_→_*_Z_* (520 nm)	Φ*_Z_*_→_*_E_* (400 nm)	Φ*_E_*_→_*_Z_* (520 nm)

acetone [3]	0.72	0.90	0.48	0.85
acetonitrile [13]	0.43	0.56	0.48	0.79
MeCN/H_2_O 9:1 [13]	0.37	0.56	–	–
H_2_O [3]	–	–	0.51	0.85

There are two strategies of applying diazocines in photopharmacology. The first one exploits the structural similarity of the tricyclic diazocine framework to the tricyclic structure of, e.g., tetrahydrodibenzazocines [16–17] and tetracyclic steroid scaffolds such as 17β-estradiol [18], where the diazocine core mimics the framework of the bioactive compound (Figure 1a). The other option is to attach the diazocine photoswitch as a substituent (appendix) to the biologically active molecule (Figure 1b) [6,10,19–21]. The art of designing a photoswitchable drug is to place the switch at a position in the pharmacophore that allows switching of the biological effect by irradiation with light without greatly reducing the overall activity by unselective interference with the inhibitor–receptor interaction. This is a difficult task because the design of a photoswitchable agent usually starts with a known, non-switchable drug or a known biological molecule, which is already carefully "optimized" either by pharmaceutical industry or by nature. Hence, there is a high risk that any change in structure will also lead to a reduction in efficiency. In any case the light-induced geometry change via isomerization should selectively control the interaction between the inhibitor and the receptor [21].

**Figure 2:**
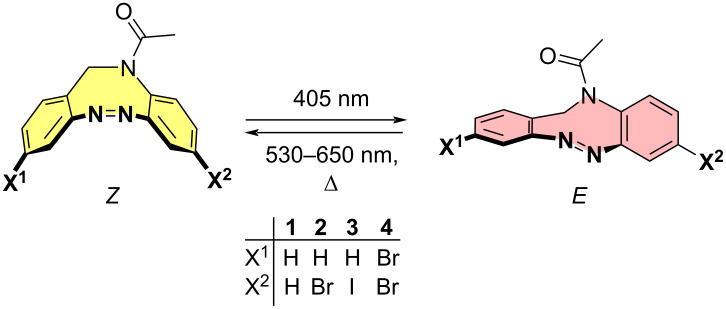
The halogen-substituted *N*-acetyl diazocines **2–4** were used as the starting compounds for further derivatization via Pd-catalyzed cross-coupling reactions. Solutions of the *Z* isomers are yellow. The *E* isomers are red.

Currently there is only one example reported in the literature for the incorporation of *N*-acetyl diazocines into biologically active molecules [17]. As a starting point for further derivatization, the synthesis and characterization of monohalogenated *N*-acetyl diazocines **2** and **3** (Figure 1) have been performed [22]. Unfortunately, diazocines in general, and *N*-acetyl diazocines in particular cannot be derivatized by electrophilic aromatic substitution. Substituents such as halogen atoms must be introduced into the *N*-acetyl diazocine structure during the synthesis of the building blocks. In the present work we start from mono- and dihalogenated *N*-acetyl diazocine **2**–**4** (Figure 2) and focus on the further derivatization via cross-coupling reactions and the synthesis of a new dihalogenated *N*-acetyl diazocine **4** (Figure 2).

## Results and Discussion

The monosubstituted *N*-acetyl diazocines **2** and **3** were synthesized according to the procedure published by our group recently [22]. The synthesis of disubstituted compound **4** followed the same procedure except the preparation of the aminoaniline building block *tert*-butyl (2-amino-5-bromophenyl)carbamate (**5**), which was prepared by Boc-protection of the 5-bromo-2-nitroaniline (**6**) and subsequent reduction of the nitro group (see Supporting Information File 1, section II.1).

### Cross-coupling reactions

Stille cross-coupling reactions were performed by an organic halide reacting with an organotin compound. A great advantage of the used organostannanes is the easy accessibility, and their high air and moisture stability, so that usually a wide range of functional groups can be introduced under mild conditions [23]. Nevertheless, the arylation of monohalogenated *N*-acetyl diazocines via Stille coupling in our case gave unsatisfying results (Table 2). Reactions with tetrakis(triphenylphosphine)palladium(0) as catalyst resulted in no product **7** formation. Bis(tri-*tert*-butylphosphine)palladium(0) as catalyst gave rise to the product in very low yields independent from the used halogenated diazocine. In contrast to other cross coupling reactions described in this work, most of the starting material decomposed during the reaction and could not be re-isolated.

**Table 2:** Reaction conditions of the arylation of halogenated *N*-acetyl diazocines via Stille coupling reaction. Equivalents are normalized to the used amount of *N*-acetyl diazocine starting material.

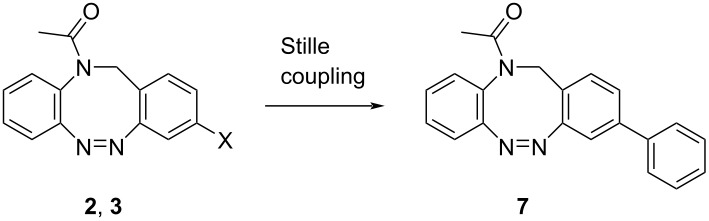			

cat. system	organotin compound	conditions	yield

Pd(OAc)_2_ (0.1 equiv) + PPh_3_ (0.8 equiv)	Ph_3_SnCl (1 equiv)	dry DMF, N_2_, 100 °C, 16 h	–
Pd(*t-*Bu_3_P)_2_ (0.1 equiv)	Ph_3_SnCl (1 equiv)	dry THF, N_2_, 65 °C, 16 h	X = Br, 10% X = I, 10%

In contrast, the vinylation of diazocines **2** and **3** provides good yields of 65% and 71%, respectively, for the vinyl *N*-acetyl diazocine **8** (Table 3). An alternative way of vinylating *N*-acetyl diazocines is the Pd-catalyzed vinylation with polyvinylsiloxanes and TBAF as activating agent following the method by Denmark et al. giving rise to even higher yields of 74% for bromine **2** and 78% for iodo starting material **3** (Table 3) [24].

**Table 3:** Vinylation of halogenated *N*-acetyl diazocines via Pd-catalyzed coupling reactions. Equivalents are normalized to the used amount of *N*-acetyl diazocine starting material.

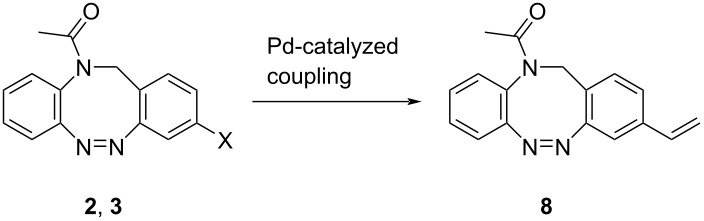			

cat. system	reaction partner or additive	conditions	yield

Pd(OAc)_2_ (0.1 equiv) + PPh_3_ (0.8 equiv)	tributylvinyltin (1 equiv)	dry DMF, N_2_, 100 °C, 16 h	X = Br, 65% X = I, 71%
PdBr_2_ (0.1 equiv) + JohnPhos (0.1 equiv)	D_4_^Va^ (0.66 equiv) TBAF (4 equiv)	dry THF, N_2_, 50° C, 16 h	X = Br, 74% X = I, 78%

^a^D_4_^V^: 1,3,5,7-tetramethyl-1,3,5,7-tetravinylcyclotetrasiloxane.

To overcome the problems of poor yields in the arylation of *N*-acetyl diazocines via Stille coupling we used Suzuki–Miyaura reactions of the diazocines **2** and **3** with different arylboronic acids [25–26]. There are several examples of last-step modifications of azobenzenes via Suzuki–Miyaura reactions in the current literature, which indicate that the reaction conditions are compatible with azo groups [27–28]. Suzuki–Miyaura reaction of **2** and **3** with different phenylboronic acids resulted in the formation of the corresponding arylated *N*-acetyl diazocines **7**, and **9**–**13** in yields from 68 to 88% (Table 4). The yields increased slightly if boronic acids with electron-withdrawing groups were used. An influence of bulky substituents like carboxyl groups in *ortho*-position of the phenylboronic acids on the reaction was not observed. The synthesis of *N*-acetyl diazocines connected to heteroaromatic aromatic systems **14**–**16** was less successful. The pyridine-substituted *N*-acetyl diazocine **14** was formed in yields of 7% or 19% while furan- **15** and thiophene-substituted *N*-acetyl diazocine **16** could not be obtained. The reaction with benzylboronic acid gave the corresponding *N*-acetyl diazocine **17** (45%, Table 4). Interestingly, this reaction only took place if brominated *N*-acetyl diazocine **2** was used as starting material although iodoaryl compounds are in general more reactive [25]. The reaction of halogenated *N*-acetyl diazocines **2** and **3** with bis(pinacolato)diboron did not lead to the formation of the pinacolborane-substituted *N*-acetyl diazocine **18**. Accordingly, the Suzuki–Miyaura reaction with inversed roles between *N*-acetyl diazocine boronic acid pinacol ester and aryl or alkyl halides could not be investigated.

**Table 4:** Derivatization of halogen-substituted *N*-acetyl diazocines via Suzuki–Miyaura reaction. Equivalents are normalized to the used amount of *N*-acetyl diazocine starting material.

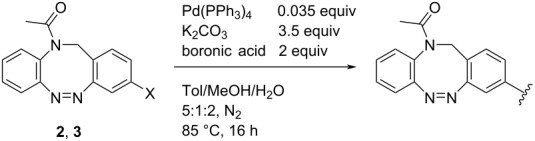		

boronic acid	product	yield

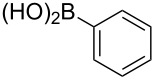	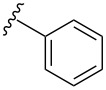 **7**	X = Br, 74% X = I, 83%
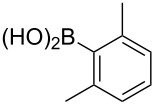	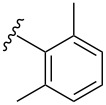 **9**	X = Br, 68% X = I, 74%
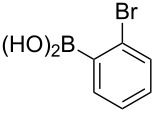	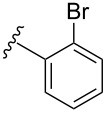 **10**	X = I, 82%
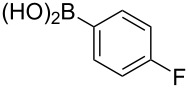	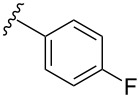 **11**	X = Br, 81% X = I, 88%
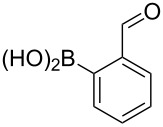	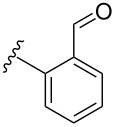 **12**	X = Br, 70% X = I, 77%
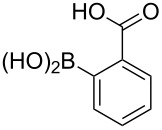	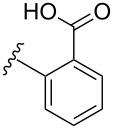 **13**	X = Br, 76%^a^ X = I, 79%^a^
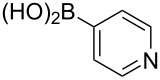	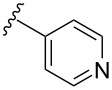 **14**	X = Br, 7% X = I, 19%
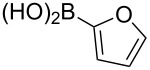	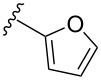 **15**	X = Br, – X = I, –
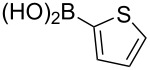	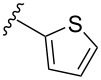 **16**	X = Br, – X = I, –
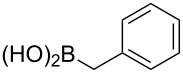	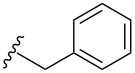 **17**	X = Br, 45% X = I, –
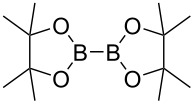	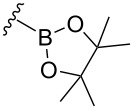 **18**	X = Br, – X = I, –

^a^The reaction was carried out in dry DMF at 100 °C because no reaction took place if the Suzuki–Miyaura standard procedure was applied.

The Buchwald–Hartwig amination is a versatile and powerful tool for C–N bond formation and widely applied in the synthesis of new pharmaceutical substances [29–31]. Furthermore, azobenzenes [32–33], as well as diazocines [34–35], have been derivatized via Buchwald–Hartwig amination. The Buchwald–Hartwig amination of halogenated *N*-acetyl diazocines according to the procedure of Maier et al. [34] with *tert*-butyl carbamate resulted in the formation of Boc-protected amino-substituted *N*-acetyl diazocine **19** in a yield of 72%. However, the reaction only took place if iodo *N*-acetyl diazocine **3** was used as starting material. Using diphenylamine as a more electron-rich amine resulted in the formation of diphenylamino-substituted *N*-acetyl diazocine **20** in a significantly lower yield of 25% starting from the bromide **2** and 47% starting from the iodo precursor **3** (Table 5).

**Table 5:** Derivatization of halogenated *N*-acetyl diazocines **2** and **3** via Buchwald–Hartwig amination. Equivalents are normalized to the used amount of *N*-acetyl diazocine starting material.

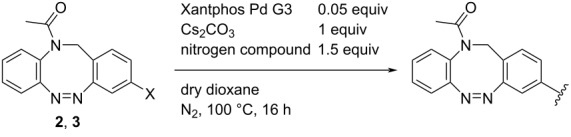		

nitrogen compound	product	yield

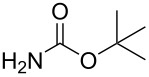	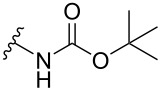 **19**	X = Br, – X = I, 72%
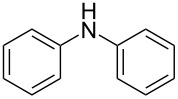	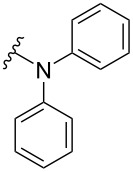 **20**	X = Br, 25% X = I, 47%

**Scheme 1:**
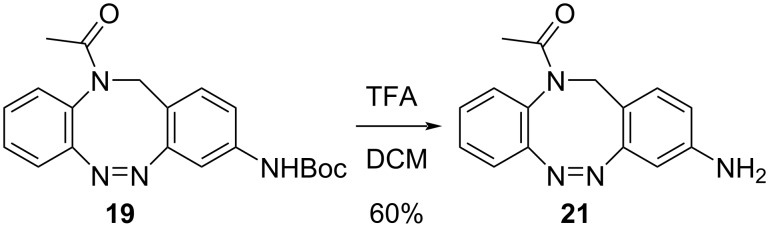
Synthesis of amino-*N*-acetyl diazocine by deprotection of the carbamate.

Deprotection of carbamate **19** with trifluoroacetic acid provided the corresponding amino-substituted *N*-acetyl diazocine **21** (Scheme 1).

**Scheme 2:**
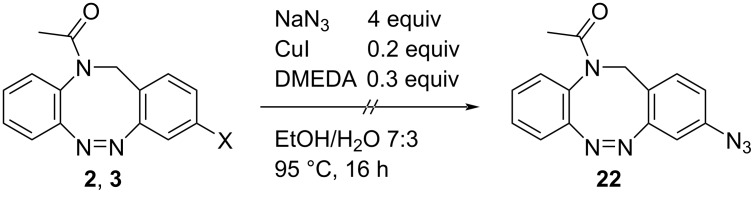
Reaction conditions for the attempted Ullmann-type reaction with sodium azide.

Another option for carbon–heteroatom bond formation reactions are copper-catalyzed Ullmann-type reactions, which have already been applied to the parent diazocine [36–37]. The attempted synthesis of azide-functionalized *N*-acetyl diazocine **22** under the conditions described by Hugenbusch et al. [37] showed no product formation and only starting material was isolated (Scheme 2).

**Scheme 3:**
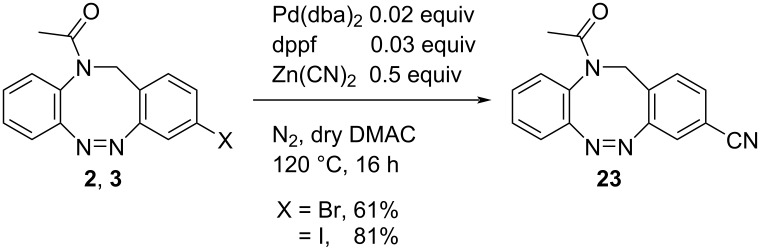
Reaction conditions for the palladium-catalyzed introduction of a nitrile functionality.

The palladium-catalyzed introduction of cyano groups under mild conditions in analogy to Iqbal et al. [38] gave the cyano-substituted *N*-acetyl diazocine **23** in yields of 61% from bromide **2** and 81% from iodide **3** (Scheme 3). Nitriles are a good starting point for further functional group interconversions [39].

### Photochemical characterization

With these new *N*-acetyl diazocine derivatives at hand we turned towards the photochemical characterization, in particular to gain insight into the effects of different substituents on UV spectra and switching behavior. For the determination of the n–π*-absorption maxima of the *E* and *Z* isomers 250 µM solutions of each compound in acetonitrile were prepared and measured at 25 °C. All compounds (**4**, **7**–**14**, **17**, **19**–**21**, **23**) exhibit an n*–*π*-transition at approximately 400 nm, matching the n*–*π*-transition of unsubstituted *N*-acetyl diazocine **1** (Table 6). Irradiation with light of 405 nm gives the metastable *E* isomers with photoconversion yields of 76–85% due to a very good separation of the n*–*π*-transitions. *N*-Acetyl diazocine derivatives containing electron-deficient groups (like **4**, **10**, **12**, **14** and **23**) show slightly but not significantly higher *Z*→*E* conversion rates than the other coupling products presented in this work. The nitrogen-substituted derivatives **19**–**21** show significantly lower conversion rates of 41–61%. This behavior has already been observed in other amino-substituted diazocines as well and is probably caused by the overlap of n*–*π*-transitions of the *E* and *Z* isomers and the electron-rich azo group [40]. An almost complete *E*→*Z* conversion (>99%) can be achieved by irradiation with light between 520 and 600 nm for all synthesized compounds.

**Table 6:** Photophysical properties of *N*-acetyl diazocines **1**-**4**, **7**–**14**, **17**, **19**–**21**, and **23** in acetonitrile.

	λ_max_ (Z)	λ_max_ (E)	*t*_1/2_ (25 °C)	*k* (25 °C)	Γ_Z→E_ (405 nm)^a^	Γ_E→Z_ (530 nm)
	nm	nm	min	10^−4^ s^−1^		

**1** [20]	397	513	29.5	3.916	88%	>99%
**2** [20]	397	515	30.9	3.744	81%	>99%
**3** [20]	397	517	28.6	4.036	82%	>99%
**4**	396	519	16.9 ± 0.20	6.820 ± 0.096	85%	>99%
**7**	399	516	29.3 ± 0.11	3.944 ± 0.017	79%	>99%
**8**	398	515	29.6 ± 0.16	3.897 ± 0.025	76%	>99%
**9**	394	515	34.2 ± 0.28	3.374 ± 0.032	77%	>99%
**10**	396	516	34.2 ± 0.28	3.382 ± 0.033	82%	>99%
**11**	397	515	33.8 ± 0.24	3.418 ± 0.029	78%	>99%
**12**	396	517	29.8 ± 0.09	3.877 ± 0.014	80%	>99%
**13**	399	517	31.2 ± 0.18	3.688 ± 0.024	77%	>99%
**14** **^b^**	396	515	39.9 ± 0.44	2.897 ± 0.038	79%	>99%
**17**	398	515	29.3 ± 0.05	3.944 ± 0.008	82%	>99%
**19**	397	514	30.6 ± 0.05	3.779 ± 0.008	61%	>99%
**20**	389	510	31.5 ± 0.05	3.670 ± 0.008	49%	>99%
**21**	396	511	38.9 ± 0.49	2.973 ± 0.043	41%	>99%
**23**	396	518	39.8 ± 0.41	2.906 ± 0.035	83%	>99%

^a^Extrapolated values (for details, see Supporting Information File 1, section IV) in deuterated acetonitrile 5 mM. ^b^UV spectra measured with a concentration of 128 µM in acetonitrile and NMR spectra with a concentration of 2.55 mM.

Thermal half-lives (*t*_1/2_) were determined by monitoring the thermal relaxation of the synthesized diazocines at 25 °C in a UV spectrometer (see Supporting Information File 1, section III). The dihalogenated *N*-acetyl diazocine **4** shows a significantly reduced half-life compared to the mono-halogenated *N*-acetyl diazocines **2** and **3** and the unsubstituted *N*-acetyl diazocine **1**. The substitution with a phenyl group does not exert a significant influence on the thermal half-life. The half-lives of compounds **7** and **17** are nearly identical compared to the parent *N*-acetyl diazocine **1**. If electron-withdrawing substituents are added in *ortho*-position of the additional phenyl ring (**12** and **13**) the half-life is not affected significantly as well. An increase of about 10% of the half-lives has been observed for the weak +M substituents bromine (**10**) and fluorine (**11**) or methyl groups (**9**) in *ortho*- and *para*-positions. The increase is even stronger if electron-withdrawing pyridine- (**14**), cyano- (**23**) or the strong +M amino substituents (**21**) are attached to the *N*-acetyl diazocine in *meta*-position. In contrast to the extended half-life of the unsubstituted amine **21** the Boc-protected **19** and the diphenyl-substituted amine **20** show half-lives not significantly longer than the parent *N*-acetyl diazocine **1**.

Given the water solubility and the excellent switching behavior of parent **1** in aqueous media [3,13,20], the photochemical properties of water-soluble substituted *N*-acetyl diazocines **13** and **21** were also investigated in aqueous solution (**13** and **21** in aqueous PBS buffer solution at pH 7.4 250 µM, **21** at pH 3.5 250 µM, **13** at pH 9 250 µM). Benzoic acid derivative **13** was representatively chosen for polar aromatic substitution and the amino derivative **21** for non-aromatic substitution. Non- or less polar aromatic substituents were not characterized in detail since their solubility in pure water is decreased by the additional phenyl substituent. The pH values were chosen to make sure that the amine **21** is completely protonated and the carboxylic acid **13** is completely deprotonated. UV measurements revealed that the absorption maxima of the n*–*π*-transitions of the *Z* isomers of **13** and **21** (392–398 nm) are almost independent of solvent and pH, while the n–π*-transitions of the *E* isomers at ≈515 nm are significantly shifted to shorter wavelengths (Δλ_max_ = 10–20 nm) in water (Table 7). At the same time the n*–*π*-transitions of the *E* isomers, which strongly overlap with the π–π*-transition in organic solvents are shifted to higher wavelengths (see Figures SIII.15–SIII.20 and SIII.29–SIII.36, Supporting Information File 1). For diazocine **13** this leads to a lower PSS of 53% *E* configuration in water at pH 7.4 and 48% at pH 9 while showing a PSS of 77% in acetonitrile for the *Z* to *E* photoisomerization. This is due to a higher overlap of the n*–*π*-transitions in both isomers in aqueous media. For the amino-substituted diazocine **21** the PSS (Γ_Z→E_) shows a slightly lower value of 37% *E* in water at pH 7.4 compared to 41% in acetonitrile, while in acidic aqueous media a PSS of 62% *E* for the *Z*→*E* photoisomerization was observed. This is due to the complete protonation of the amino group converting it to an electron-deficient substituent. The thermal half-lives of **13** and **21** increase by a factor of 2.5 and 4.2 when changing the solvent from acetonitrile to water at pH 7.4, which is consistent with the current literature for thermal half-lives of substituted parent diazocines in aqueous media [18,39].

**Table 7:** Photophysical properties of *N*-acetyl diazocines **1**, **13**, and **21** in water at various pH values.

	λ_max_ (Z)	λ_max_ (E)	*t*_1/2_ , 25 °C	*k*, 25 °C	Γ_Z→E_ (405 nm)^a^	Γ_E→Z_ (530 nm)
	nm	nm	min	10^−4^ s^−1^		

**1** [20]	393	502	72.8	1.587	72%	>99%
**13** at pH 9	392	502	106.4 ± 1.34	1.086 ± 0.016	48%	>99%
**13** at pH 7.4	392	505	78.2 ± 0.33	1.477 ± 0.007	53%	>99%
**21** at pH 3.5	392	495	118.0 ± 1.72	0.975 ± 0.017	62%	>99%
**21** at pH 7.4	392	489	162.5 ± 1.79	0.711 ± 0.009	37%	>99%

^a^Extrapolated values (for details, see Supporting Information File 1, section IV) in deuterated water 5 mM.

## Conclusion

Fourteen mono-*meta*-substituted (**7**–**14**, **17**, **19**–**21**, **23**) and one di-*meta*-substituted (**4**) *N*-acetyl diazocines have been synthesized and characterized. The synthesis has been performed from halogenated precursors and cross-coupling reactions for further functionalization. The reaction conditions of various cross-coupling reactions have been correspondingly adjusted. The arylation of the *N*-acetyl diazocine system could be achieved via Suzuki coupling reactions in high yields (**7**, **9**–**14**, **17**) as well as the vinylation via Stille coupling (**8**). These compounds also exhibit excellent switching properties. Electron-withdrawing substituents at the aryl substituents have no significant influence on the switching behavior while weak +M substituents like bromine and fluorine as well as electron-poor heteroaromatic systems lead to increased thermal half-lives. An amino-substituted derivative **21** was obtained via Buchwald–Hartwig coupling with Boc-carbamate and subsequent deprotection. Amino-substituted *N*-acetyl diazocines **19**–**21** exhibit photostationary states (PSS) lower in their metastable configurations in analogy to amino-substituted azobenzenes and previously synthesized diazocines. We also investigated the switching properties of the water-soluble derivatives **13** and **21** in water at different pH values. The half-lives of the metastable *E* isomers are significantly longer in water than in less polar solvents like acetonitrile. For carboxylic acid-substituted **13**, the *Z*→*E* conversion upon irradiation with 405 nm drops from 77% to 53% upon changing the solvent from acetonitrile to water (pH 7.4). The reverse effect was observed with amino-substituted **21**.

The photophysical properties of photoswitchable drugs in photopharmacology are usually determined in organic solvents. Their natural environment, however, is the aqueous phase. There is a risk of overestimating the performance of photochromic drugs because photoconversion to the active state usually drops considerably in water, and also half-lives are different. Light-activatable drugs based on *N*-acetyl diazocines are more hydrophilic than those derived from the parent system diazocine and corresponding azobenzenes. They retain their switching properties even in an aqueous environment and are therefore promising switches in photopharmacological applications.

## Supporting Information

CCDC-2329263 (**1**), CCDC-2329261 (**2**), and CCDC-2329262 (**7**) contain the supplementary crystallographic data for this paper. These data can be obtained free of charge from the Cambridge Crystallographic Data Centre via http://www.ccdc.cam.ac.uk/data_request/cif. Cambridge CB2 1EZ, UK; fax: +44 1223 336033.

File 1 Synthetic procedures, UV–vis and NMR switching experiments, copies of UV–vis and NMR spectra, and X-ray crystallographic data.

## Data Availability

All data that supports the findings of this study is available in the published article and/or the supporting information of this article.
