# Supplementary material for: Synthesis of N-acetyl diazocine derivatives via cross-coupling reaction
Source: Beilstein J Org Chem. 2025 Mar 4;21:490–9. doi: 10.3762/bjoc.21.36 (PMC11897653; doi:10.3762/bjoc.21.36)
Supplement: File 1 — Synthetic procedures, UV–vis and NMR switching experiments, copies of UV–vis and NMR spectra, and X-ray crystallographic data. [file Beilstein_J_Org_Chem-21-490-s001.pdf]

BEILSTEIN JOURNAL OF ORGANIC CHEMISTRY

# Supporting Information

for

# Synthesis of *N*-acetyl diazocine derivatives via cross-coupling reaction

Thomas Brandt, Pascal Lentes, Jeremy Rudtke, Michael Hösgen, Christian Näther and Rainer Herges

*Beilstein J. Org. Chem.* **2025,** *21,* 490–499. doi:10.3762/bjoc.21.36

# Synthetic procedures, UV–vis and NMR switching experiments, copies of UV–vis and NMR spectra, and X-ray crystallographic data

License and Terms: This is a supporting information file under the terms of the Creative Commons Attribution License (https://creativecommons.org/licenses/by/4.0). Please note that the reuse, redistribution and reproduction in particular requires that the author(s) and source are credited and that individual graphics may be subject to special legal provisions.
The license is subject to the *Beilstein Journal of Organic Chemistry* terms and conditions: (https://www.beilstein-journals.org/bjoc/terms)

# Table of Contents

I. Analytical Equipment ........ S2
II. Syntheses ........ S4
II.1 Synthesis of halogenated *N*-acetyl diazocines ........ S4
II.2 Syntheses of diazocine derivatives via cross-coupling reactions ........ S51
III. UV-vis switching experiments ........ S103
IV. $^{1}$H-NMR switching experiments ........ S116
V. X-ray crystallographic data ........ S132
VI. References ........ S135

# I. Analytical Equipment

## NMR spectroscopy

NMR spectra were measured in deuterated solvents (Deutero). The spectra were referenced to the following solvent residual signals:

| solvent | degree of deuteration | $^{1}H$ signal ppm | $^{13}C$-signal ppm |
|---|---|---|---|
| acetone-$d_6$ | 99.8% | 2.05 (quintet) | 29.84 (septet) |
| chloroform-$d_1$ | 99.8% | 7.26 (singlet) | 77.16 (triplet) |
| acetonitrile-$d_3$ | 99.8% | 1.94 (singlet) | 1.32 (septet), 118.26 (mulitplet) |
| water-$d_2$ | 99.9% | 4.79 (singlet) | - |

The spectra were recorded with a Bruker DRX 500 ($^{1}H$-NMR: 500 MHz, $^{13}C$-NMR: 125 MHz) and a Bruker AV 600 ($^{1}H$-NMR: 600 MHz, $^{13}C$-NMR: 150 MHz). The Multiplicities of the signals were abbreviated with s (singlet), d (doublet), t (triplet), q (quartet), quint. (quintet), m (multiplet), and br. (broad) in addition for broad signals. DEPT data were used to assign carbon types. Structural assignments were made with additional information from HSQC and HMBC experiments. Baseline correction of the spectra in $D_2O$ was performed with Baseline Correction “Witthaker Smoother” tool and Signal Suppression tool in MestReNova 14.3.1 NMR analytic software by Mestrelab Research. Spectra were plotted with TopSpin 4.1.0 NMR analytic software by Bruker. Major impuritites in the 1D-NMR spectra used for compound characterization have been identified as far as possible and labeled accordingly.

## Melting Point

Melting Points were measured with a Melting Point B-560 (Büchi) in melting point tubes without further correction.

## Mass spectrometry

Mass spectra (EI) and high-resolution mass spectra (HR-EI) were measured with an AccuTOF GCv 4G EI-time of flight mass spectrometer from the company Joel. An ionization energy of 70 eV was used.
HR-ESI mass spectra were measured with a Q Exactive Plus Hybrid Quadrupole-Orbitrap ESI-mass spectrometer of the company Thermo Scientific was used.

## IR spectroscopy

Infrared spectra were measured with a Perkin-Elmer 1600 FT-IR spectrometer with an A531-G Golden-Gate-Diamond-ATR-unit. Signals were abbreviated with w (weak), m (medium) or s (strong) for its intensity.

## UV-vis spectroscopy

UV-vis spectra were measured with a Shimadzu UV-2600 i UV-vis spectrometer. Quartz cuvettes of 10 mm optical path length were used.

## Chromatography

Silica gel (Merck, particle size 0.040-0.063 mm) was used for column chromatography purifications. $R_f$ values were determined via thin layer chromatography on Polygram® SilG/UV254 (Macherey Nagel, 0.2 mm particle size).

## Chemicals

All commercially available chemicals were used without further purification.

## Light sources

For irradiation different custom-built light sources with a wavelength of 405 nm and 530 nm were used (Sahlmann Photochemistry Solutions & in-house built). Irradiation wavelengths for all (405 nm and 530 nm) LED units were measured via a mobile UV-vis spectrometer (USB4000-UV-VIS, Ocean Optics, Largo, FL, USA). All emission spectra were normalized. Figure SI1 shows the normalized emission spectra of all LED units used.

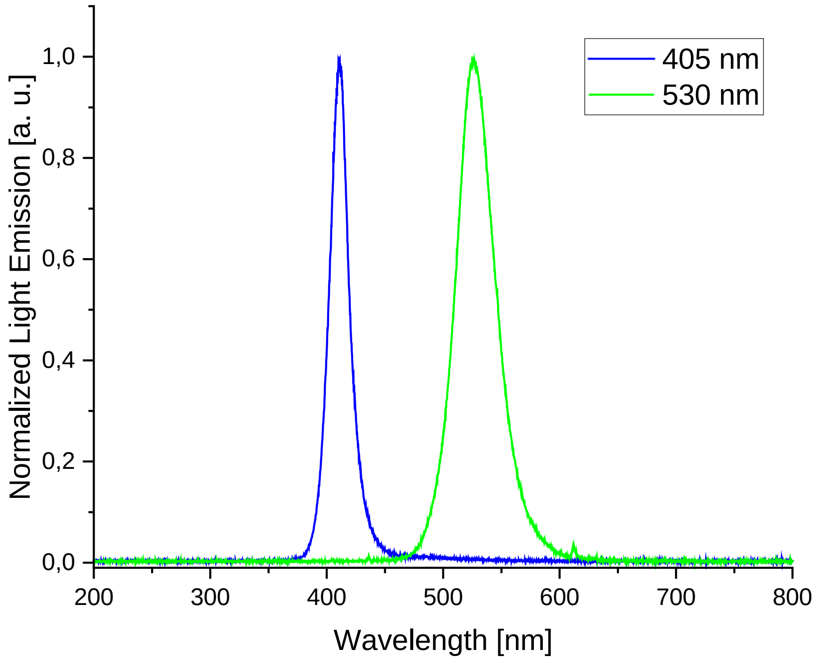


**Fígure SI.1:** Emission spectra of all LED units used. For better comparison all datasets were normalized to its maximum.

The full width at half maximum (FWHM) amounts to 19 nm (401-420 nm, maximum at 410 nm) for the 405 nm LED unit and to 38 nm (509-547 nm, maximum at 525 nm) for the 530 nm LED unit.

# II. Syntheses

## II.1 Synthesis of halogenated *N*-acetyl diazocines

The halogenated *N*-acetyl diazocine precursors **2-4** were synthesized following the procedure of LENTES *et al.*[1] (Scheme 1). Therefore, the dianiline and bromide building blocks were connected in a nucleophile substitution in the first step followed by Fmoc-protection of the secondary amine. After cleaving the Boc-protecting group the nitro-group was reduced using stannous chloride and zink. In the following the oxidative azo-cyclization adapted from the procedure of MAIER *et al.*[2] was performed to receive the Fmoc-protected *N*-acetyl diazocine intermediates. After the Fmoc-group was cleaved the acetyl protecting group was introduced as final step.

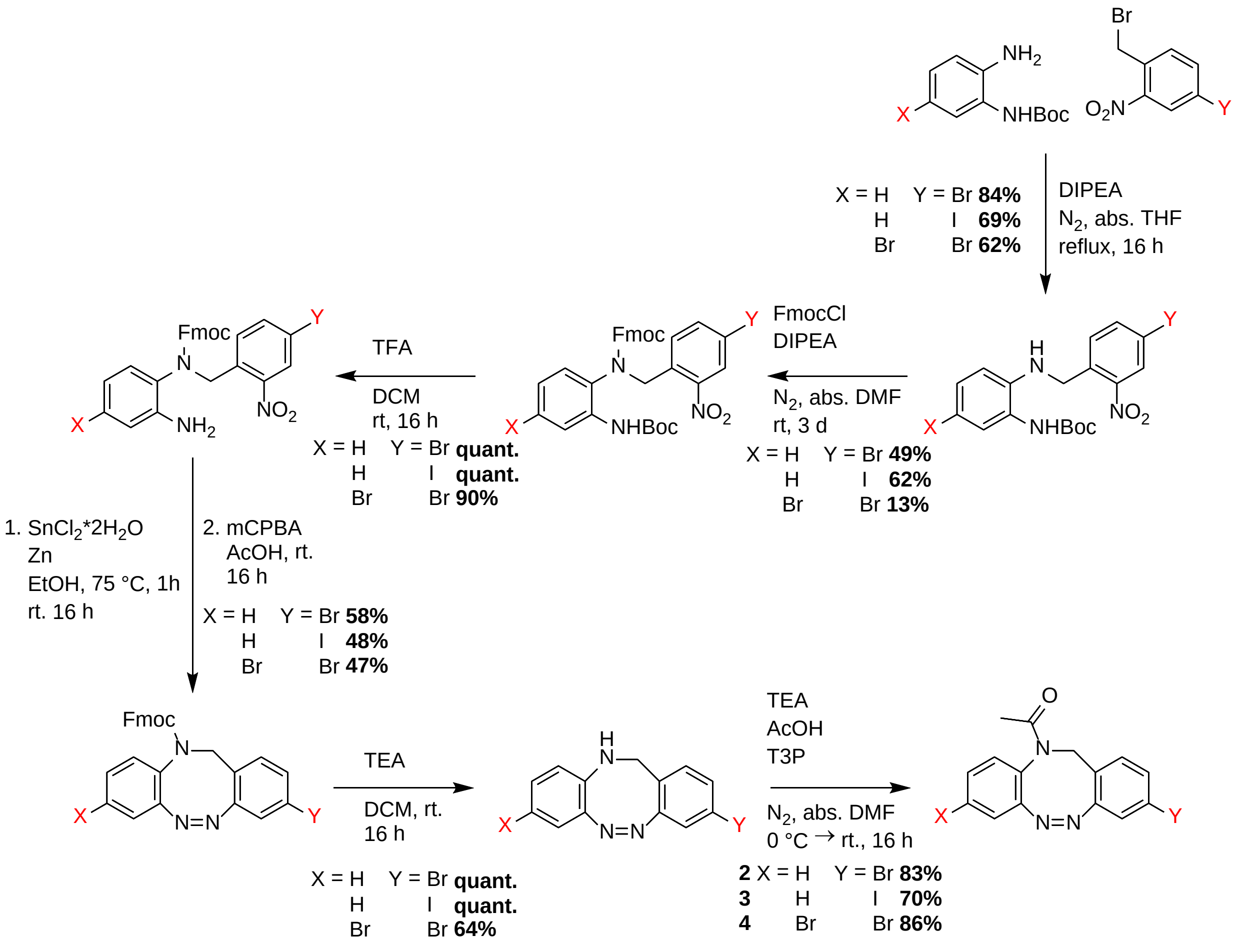


**Scheme S1:** Synthesis of the halogenated *N*-acetyl diazocine precursors **2-4** for the cross-coupling reactions according to the procedure of Lentes *et al*.[1]

For the synthesis of dibromo-*N*-acetyl diazocine **4** the Boc-protected dianiline building block **5** was prepared from commercially available 5-bromo-2-nitroaniline **6**. In the first step the Boc protecting group was introduced after deprotonation of the aniline with sodium hydride. The nitro group was reduced with sodium dithionite and sodium hydrogencarbonate subsequently to obtain dianiline building block **5**.

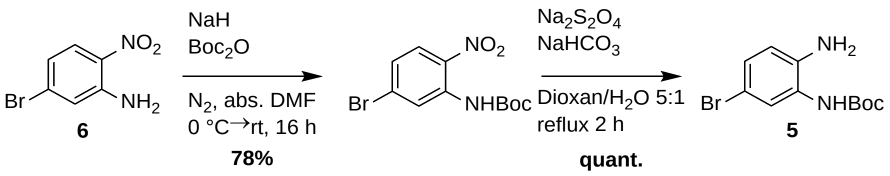


**Scheme S2:** Synthesis of dianiline building block **5** from *o*-nitroaniline **6**.

## II.1.1 Synthesis of *tert*-butyl (2-((4-bromo-2-nitrobenzyl)amino)phenyl)carbamate[1]]

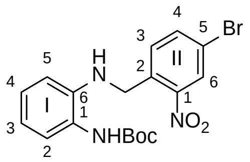


In a manner analogous to [1] triethylamine (3.87 mL, 27.9 mmol) and 4-bromo-1-(bromomethyl)-2-nitrobenzene (7.50 g, 25.4 mmol) were added to solution of *tert*-butyl (2-aminophenyl)carbamate (5.30 g, 25.4 mmol) in 100 mL dry. THF under a nitrogen atmosphere. The reaction mixture was heated to reflux with an oil bath and stirred for 16 h at that temperature. Afterwards the solvent was evaporated and 100 mL of deionized water and 100 mL DCM were added to the residue. The organic layer was separated and the aqueous layer was extracted twice with 50 mL of DCM. The combined organic layers were dried over $MgSO_4$ and the solvent was evaporated. Recrystallization from cyclohexane/ethyl acetate (1:1) gave the product as yellow solid (9.06 g, 21.4 mmol, 84%, lit. 71%[1]).

**melting point:** 147 °C

**$^1$H-NMR** (500 MHz, acetone-$d_6$, 298 K): $\delta$ = 8.24 (d, $^4J$ = 2.0 Hz, 1 H, $Ar^{II}$-*H*-6), 7.83 (dd, $^3J$ = 8.4 Hz, $^4J$ = 2.1 Hz, 1 H, $Ar^{II}$-*H*-4), 7.73 (d, $^3J$ = 8.4 Hz, 1 H, $Ar^{II}$-*H*-3), 7.63 (br. s, 1 H, N*H*), 7.25 (d, $^3J$ = 7.7 Hz, 1 H, $Ar^{I}$-*H*-2), 6.93 (td, $^3J$ = 7.8 Hz, $^4J$ = 1.5 Hz, 1 H, $Ar^{I}$-*H*-4), 6.64 (td, $^3J$ = 7.6 Hz, $^4J$ = 1.3 Hz, 1 H, $Ar^{I}$-*H*-3), 6.50 (dd, $^3J$ = 7.6 Hz, $^4J$ = 2.1 Hz, 1 H, $Ar^{I}$-*H*-5), 5.51 (br. s, 1 H, N*H*), 4.76 (s, 2 H, $CH_2$), 1.47 (s, 9 H, $CH_3$) ppm.

**$^{13}$C{$^1$H}-NMR** (125 MHz, acetone-$d_6$, 298 K): $\delta$ = 155.0 (*C*=O), 150.0 ($Ar^{II}$-*C*-1), 142.7 ($Ar^{I}$-*C*-6), 137.1 ($Ar^{II}$-*C*-4), 136.2 ($Ar^{II}$-*C*-6), 132.4 ($Ar^{II}$-*C*-3), 128.3 ($Ar^{II}$-*C*-6), 127.1 ($Ar^{I}$-*C*-4), 126.8 ($Ar^{I}$-*C*-2), 125.5 ($Ar^{I}$-*C*-1), 120.9 ($Ar^{II}$-*C*-5), 118.0 ($Ar^{I}$-*C*-3), 112.4 ($Ar^{I}$-*C*-5), 79.9 (*C*-$(CH_3)_3$), 45.1 (-$CH_2$), 28.6 (-$CH_3$) ppm.

**IR** (ATR): $\tilde{\nu}$ = 3398 (m), 3270 (m), 2975 (w), 1707 (w), 1684 (s), 1604 (m), 1528 (s), 1502 (s), 1462 (m), 1421 (w), 1393 (w), 1346 (m), 1336 (m), 1307 (w), 1242 (s), 1153 (s), 1083 (w), 1066 (w), 1052 (m), 1028 (w), 906 (w), 874 (w), 845 (w), 831 (m), 792 (w), 754 (s), 717 (w), 647 (w), 616 (w) $cm^{-1}$.

**HR-MS** (ESI, DCM): *m/z* $[M+H]^+$ calculated for $C_{18}H_{20}O_4N_3{}^{79}Br+H^+$: 422.0710; found: 422.0700 ± 2.36 ppm.

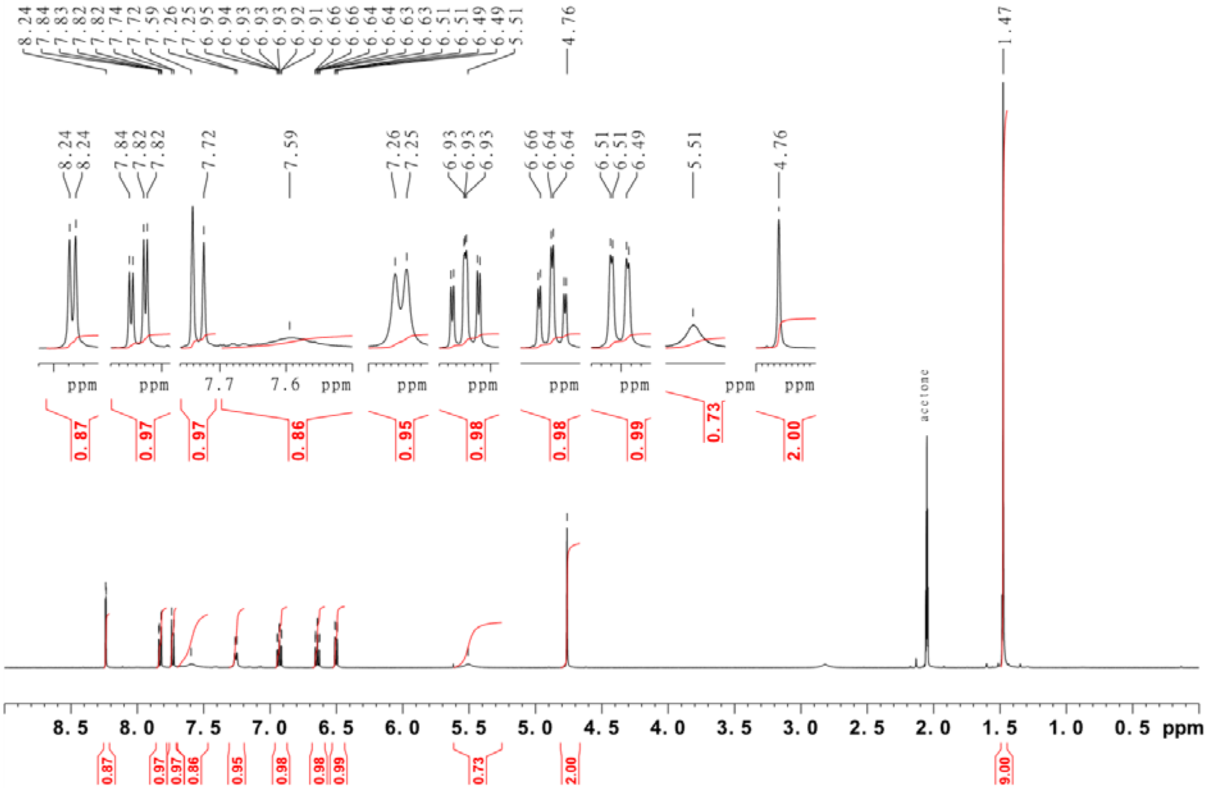

**Figure SII.1:** 500 MHz $^{1}$H-NMR spectrum of *tert*-butyl (2-((4-bromo-2-nitrobenzyl)amino)phenyl)carbamate.

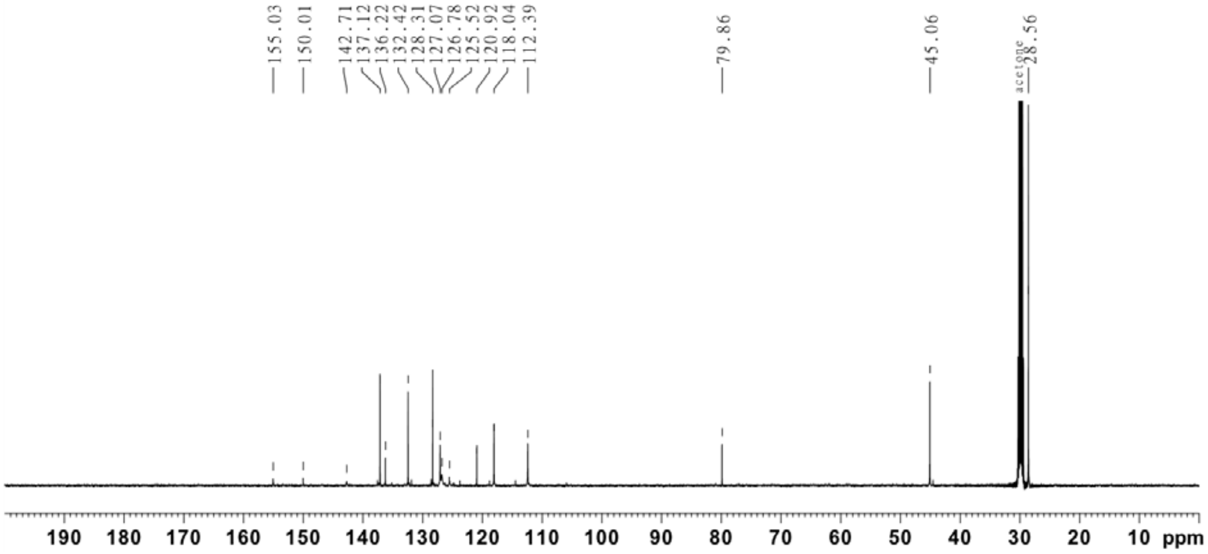

**Figure SII.2:** 125 MHz $^{13}$C{$^{1}$H}-NMR spectrum of *tert*-butyl (2-((4-bromo-2-nitrobenzyl)amino)phenyl)carbamate.

## II.1.2 Synthesis of (9*H*-fluoren-9-yl)methyl (4-bromo-2-nitrobenzyl)(2-((*tert*-butoxycarbonyl)amino)phenyl)carbamate[1]

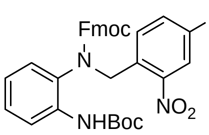


In a manner analogous to [1] DIPEA (3.21 mL, 18.9 mmol) and 9-Fluorenylmethoxycarbonyl chlorid (9.77 g, 37.8 mmol) were added to a solution of *tert*-butyl (2-((4-bromo-2-nitrobenzyl)amino)phenyl)carbamate (8.00 g, 18.9 mmol) in 50 mL dry DMF under a nitrogen atmosphere. The reaction mixture was stirred for 48 h at rt. and the solvent was evaporated subsequently. 100 mL of deionized water and 100 mL of DCM were added to the residue. The organic layer was separated and the aqueous layer was extracted twice with 100 mL DCM. The combined organic layers were dried over $MgSO_4$ and the solvent was evaporated. Column chromatography on silica (ethyl acetate/cyclohexane 1:4, $R_f$ = 0.34) gave a colorless solid (5.91 g, 9.18 mmol, 49%, lit. 56%[1]).

**melting point:** 157 °C

**$^1$H-NMR** (500 MHz, acetone-$d_6$, 298 K): $\delta$ = 8.14 (d, $^4J$ = 2.1 Hz, 1 H), 8.05-7.61 (m,5 H), 7.53-7.01 (m,10 H), 5.24-4.92 (m, 2 H, aliph.-*H*), 4.56-4.00 (m, 3 H, aliph.-*H*), 1.45 (s, 9 H, C*H*$_3$) ppm.

**$^{13}$C{$^1$H}-NMR** (125 MHz, acetone-$d_6$, 298 K): $\delta$ = 153.5 (*C*=O), 142.1, 137.0, 129.2, 128.5, 128.2, 127.8, 126.1, 124.4, 120.7, 80.4, 68.4, 47.8, 28.5 (*C*H$_3$) ppm.

**IR** (ATR): $\tilde{\nu}$ = 3381 (m), 2974 (w), 2348 (w), 2253 (w), 1717 (s), 1596 (m), 1536 (m), 1518 (s), 1450 (m), 1390 (m), 1365 (w), 1292 (m), 1278 (w), 1234 (m), 1217 (w), 1152 (s), 1042 (w), 1024 (w), 976 (m), 942 (w), 884 (m), 842 (w), 756 (s), 739 (w), 634 (w), 617 (m), 543(m) cm$^{-1}$.

**HR-MS** (ESI, DCM): *m/z* $[M+H]^+$ calculated for $C_{33}H_{30}O_6N_3{}^{79}Br+H^+$: 644.1391; found: 644.1393 ± 0.41 ppm.

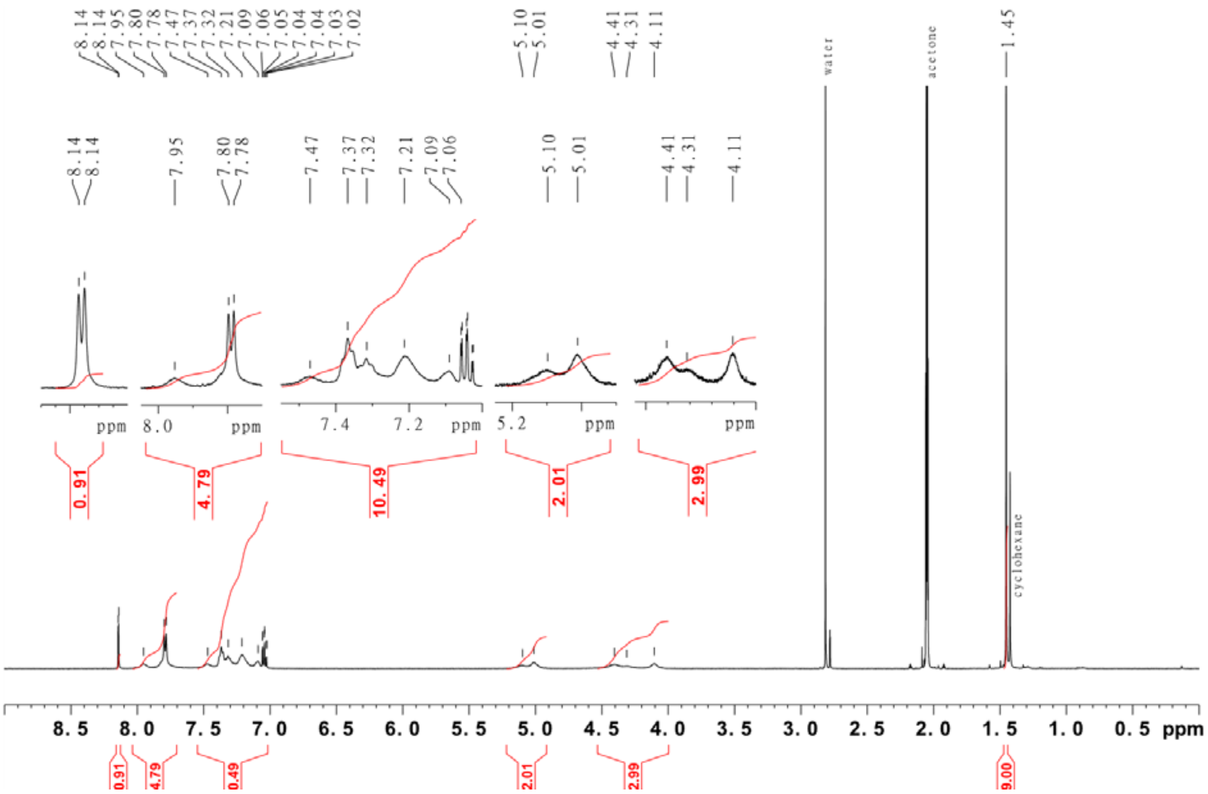

**Figure SII.3:** 500 MHz $^{1}$H-NMR spectrum of (9*H*-fluoren-9-yl)methyl (4-bromo-2-nitrobenzyl)(2-((*tert*-butoxycarbonyl)amino)phenyl)carbamate.

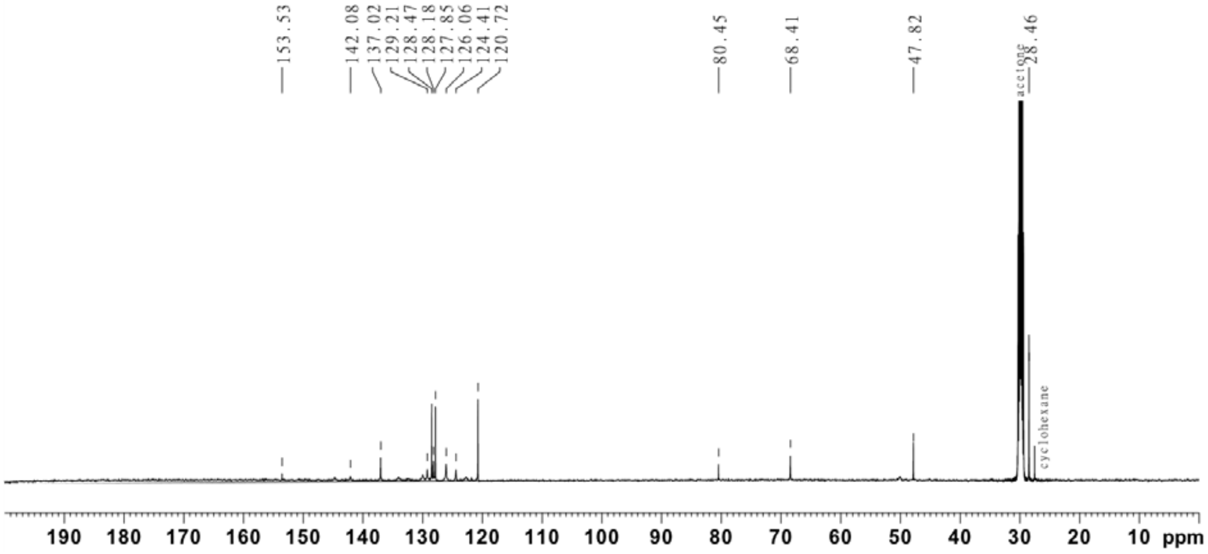

**Figure SII.4:** 125 MHz $^{13}$C{$^{1}$H}-NMR spectrum of (9*H*-fluoren-9-yl)methyl (4-bromo-2-nitrobenzyl)(2-((*tert*-butoxycarbonyl)amino)phenyl)carbamate.

### II.1.3 Synthesis of (9*H*-fluoren-9-yl)methyl (2-aminophenyl)(4-bromo-2-nitrobenzyl)carbamate[1]

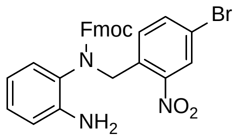


In a manner analogous to [1] (9*H*-fluoren-9-yl)methyl (4-bromo-2-nitrobenzyl)(2-((*tert*-butoxycarbonyl)amino)phenyl)carbamate (5.00 g, 7.76 mol) was dissolved in 100 mL DCM and 30 mL TFA was added. The reaction mixture was stirred at rt. for 16 h and neutralized with saturated aqueous $NaHCO_3$ subsequently. The organic layer was separated and the aqueous layer was extracted twice with 100 mL. The combined organic layers were dried over $MgSO_4$ and the solvent was evaporated to obtain a pale yellow solid (4.22 g, 7.75 mmol, quant., lit. quant.[1][1]) which was used without further purification.

**melting point:** 81 °C

**$^{1}$H-NMR** (600 MHz, acetone-$d_6$, 298 K): $\delta$ = 8.12 (s, 1 H), 7.92-7.63 (m,4 H), 7.50-6.52 (m,10 H), 5.60-4.02 (m, 5 H, aliph.-*H*) ppm.

**$^{13}$C{$^{1}$H}-NMR** (150 MHz, acetone-$d_6$, 298 K): $\delta$ = 144.7, 142.1, 136.9, 133.2, 130.3, 129.0, 128.4, 127.84, 127.79, 126.1, 124.4, 121.5, 120.72, 120.67, 68.0, 49.66, 47.97, 47.89 ppm.

**IR** (ATR): $\tilde{\nu}$ = 3425 (w), 3353 (w), 2348 (w), 2185 (w), 2163 (s), 2052 (m), 1978 (m), 1712 (s), 1619 (m), 1521 (s), 1503 (w), 1451 (w), 1433 (m), 1340 (m), 1253 (s), 1189 (w), 1172 (m), 1150 (w), 1102 (m), 1063 (m), 1051 (w), 876 (m), 764 (m), 745 (s), 659 (m), 638 (w), 621(m), 568 (w), 543 (m) $cm^{-1}$.

**HR-MS** (ESI, DCM): *m/z* $[M+H]^+$ calculated for $C_{28}H_{22}O_4N_3{}^{79}Br+H^+$: 544.0867; found: 544.0870 ± 0.58 ppm.

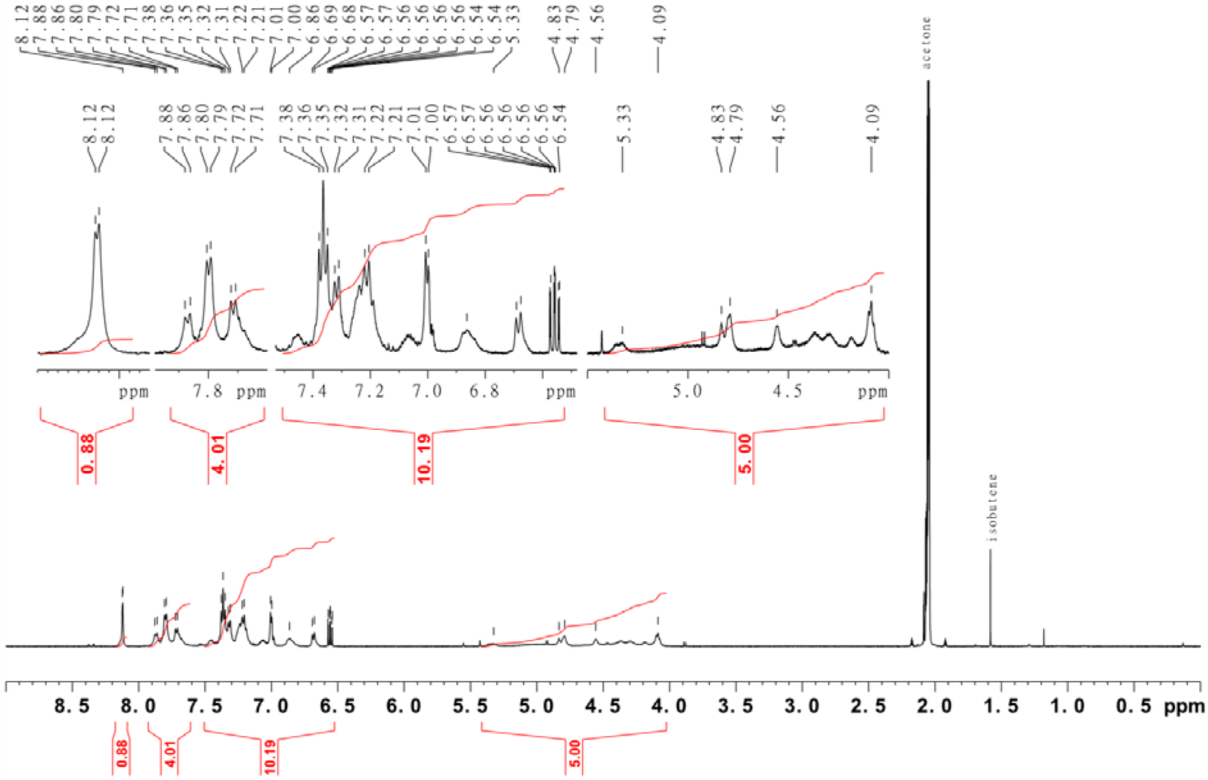


**Figure SII.5:** 600 MHz $^{1}$H-NMR spectrum of (9*H*-fluoren-9-yl)methyl (2-aminophenyl)(4-bromo-2-nitrobenzyl)carbamate.

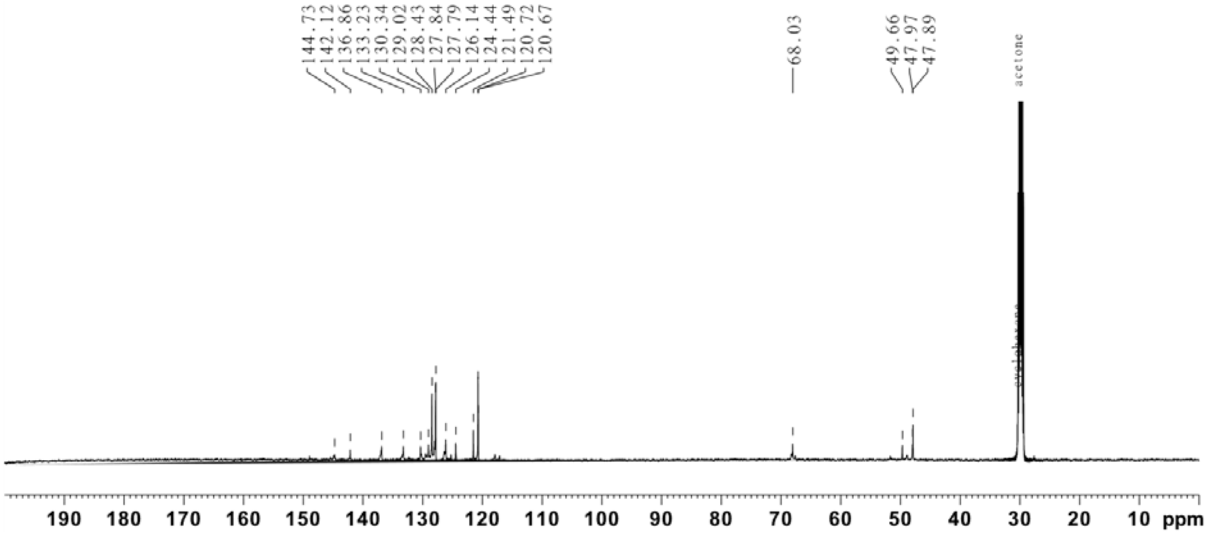


**Figure SII.6:** 150 MHz $^{13}$C{$^{1}$H}-NMR spectrum of (9*H*-fluoren-9-yl)methyl (2-aminophenyl)(4-bromo-2-nitrobenzyl)carbamate.

### II.1.4 Synthesis of (9*H*-fluoren-9-yl)methyl-(*Z*)-3-bromodibenzo[*c,g*][1,2,5]triazocin-11-(12*H*)-carboxylate[1]

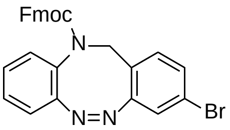


In a manner analogous to [1] (9*H*-fluoren-9-yl)methyl (2-aminophenyl)(4-bromo-2-nitrobenzyl)carbamate (4.18 g, 7.68 mmol) was suspended in 250 mL EtOH and $SnCl_2 \cdot 2\,H_2O$ (10.4 g, 46.1 mmol) was added. The reaction mixture was heated to 75 °C with an oil bath and zinc-powder (4.02 g, 61.4 mmol) was added. The reaction mixture was stirred at 75 °C for 1 h and cooled to rt. afterwards. It was stirred at rt. for 16 h and neutralized with saturated aqueous $NaHCO_3$ solution subsequently. The solvent was evaporated and 100 mL water and 100 mL DCM were added to the residue. The organic layer was separated and the aqueous layer was extracted twice with 100 mL DCM. The combined organic layers were dried over $MgSO_4$ and the solvent was evaporated. The residue was dissolved in 300 mL of acetic acid and *m*CPBA (2.66 g, 15.4 mmol) dissolved in 300 mL acetic acid was added dropwise to the reaction mixture. The reaction mixture was stirred at rt. for 16 h and the solvent was evaporated in the following. 100 mL DCM and 100 mL half-concentrated aqueous $NaHCO_3$ solution were added to the residue. The organic layer was separated and the aqueous layer was extracted twice with 100 mL DCM. The combined organic layers were dried over $MgSO_4$ and the solvent was evaporated. After column chromatography on silica (ethyl acetate/cyclohexane = 1:4, $R_f$ = 0.43) the product could be obtained as yellow solid (2.27 g, 4.45 mmol, 58%, lit. 56%[1]).

**melting point:** 75 °C

**$^1$H-NMR** (600 MHz, acetone-$d_6$, 298 K): $\delta$ = 7.99-7.77 (s, 2 H, Ar-*H*), 7.50-6.95 (m, 13 H, Ar-*H*), 4.99-3.95 (m, 5 H, aliph.-*H*) ppm.

**$^{13}$C{$^1$H}-NMR** (150 MHz, acetone-$d_6$, 298 K): $\delta$ = 154.7, 142.1, 132.7, 131.3, 130.5, 129.5, 129.1, 128.5, 127.9, 126.1, 124.2, 122.6, 120.8, 119.7, 68.5, 52.7, 47.8 ppm.

**IR** (ATR): $\tilde{\nu}$ = 2946 (w), 2320 (w), 2256 (w), 2178 (w), 2038 (w), 1999 (w), 1701 (s), 1591 (m), 1475 (m), 1447 (m), 1389 (s), 1316 (s), 1243 (m), 1132 (m), 1074 (w), 1042 (m), 1023 (m), 1003 (w), 976 (w), 864 (w), 843 (w), 822 (m), 755 (s), 736 (s), 698 (m), 622 (m), 547 (m), 515 (w), 503 (m) $cm^{-1}$.

**HR-MS** (ESI, DCM): *m/z* $[M+H]^+$ calculated for $C_{28}H_{22}O_3N_2{}^{79}Br+H^+$: 510.0812, found: 510.0817 ± 0.55 ppm.

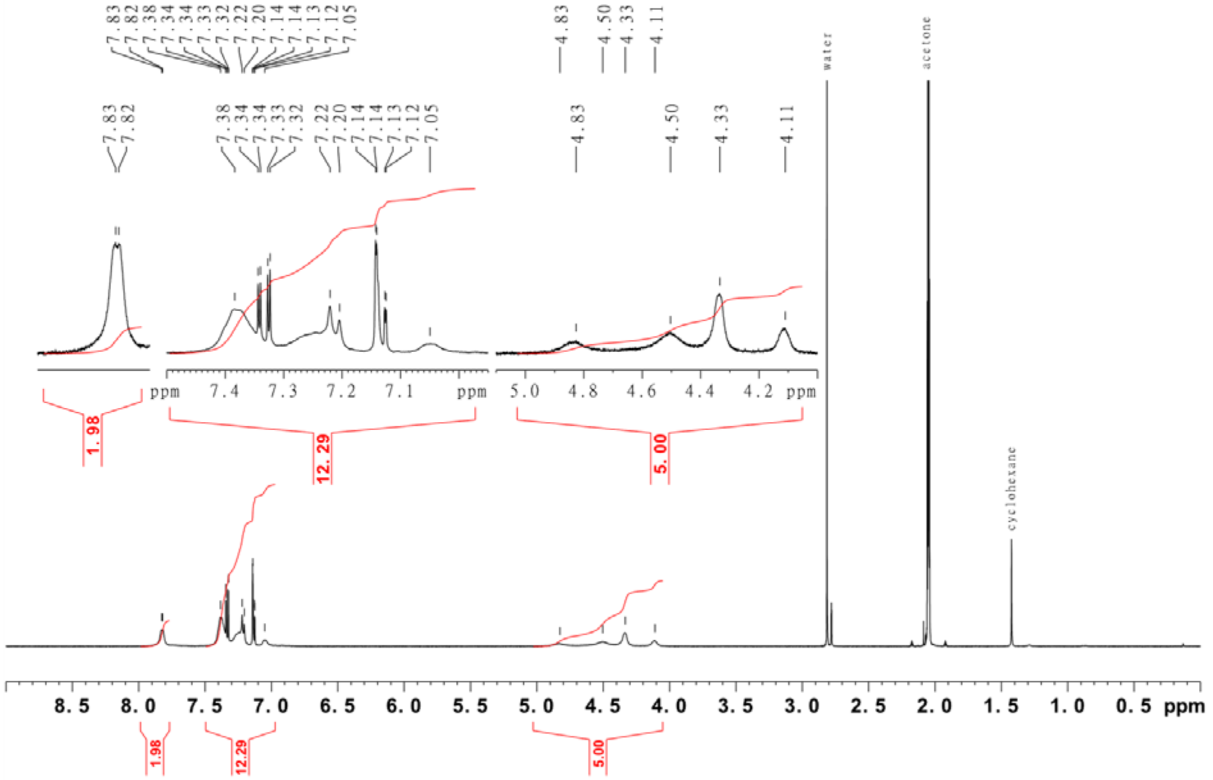

**Figure SII.7:** 600 MHz $^{1}$H-NMR spectrum of (9*H*-fluoren-9-yl)methyl (*Z*)-3-bromodibenzo[*c,g*][1,2,5]triazocin-11-(12*H*)-carboxylate.

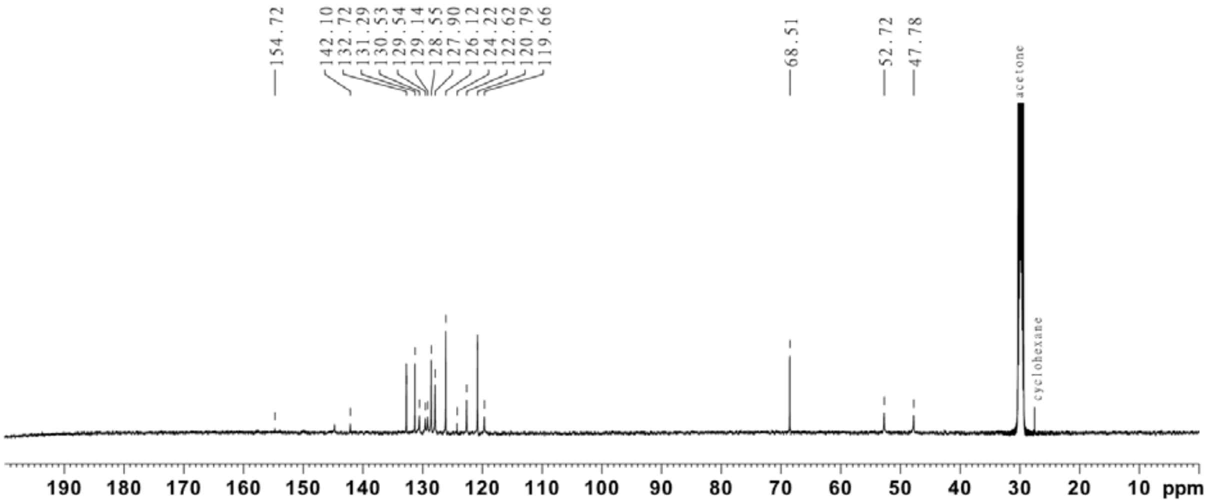

**Figure SII.8:** 150 MHz $^{13}$C{$^{1}$H}-NMR spectrum of (9*H*-fluoren-9-yl)methyl (*Z*)-3-bromodibenzo[*c,g*][1,2,5]triazocin-11-(12*H*)-carboxylate.

## II.1.5 Synthesis of (*Z*)-bromo-11,12-dihydrodibenzo[*c,g*][1,2,5]triazocine[1]

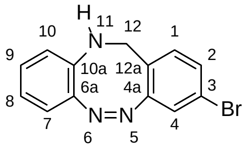

In a manner analogous to [1] 9*H*-fluoren-9-yl)methyl (*Z*)-3-bromodibenzo[*c,g*][1,2,5]triazocin-11-(12*H*)-carboxylate (1.63 g, 3.19 mmol) was dissolved in 50 mL of DCM and 50 mL $NEt_3$ was added. The reaction mixture was stirred at rt. for 16 h. the solvent was evaporated and column chromatography on silica (ethyl acetate/cyclohexane 1:2, $R_f$ = 0.32) gave a red solid (893 mg, 3.17 mmol, quant., lit. 93%[1]).

**melting point:** 149 °C

**$^1$H-NMR** (500 MHz, acetone-$d_6$, 298 K): $\delta$ = 7.42 (dd, $^3J$ = 8.1 Hz, $^4J$ = 2.0 Hz, 1 H, *H*-2), 7.33 (d, $^4J$ = 2.1 Hz, 1 H, *H*-4), 7.31 (d, $^3J$ = 8.1 Hz, 1 H, *H*-1), 6.87 (td, $^3J$ = 7.7 Hz, $^4J$ = 1.6 Hz, 1 H, *H*-9), 6.74 (dd, $^3J$ = 8.0 Hz, $^4J$ = 1.6 Hz, 1 H, *H*-7), 6.64 (td, $^3J$ = 7.4 Hz, $^4J$ = 1.2 Hz, 1 H, *H*-8), 6.56 (dd, $^3J$ = 8.1 Hz, $^4J$ = 1.1 Hz, 1 H, *H*-10), 5.51 (s, 1 H, N*H*-), 4.04 (s, 1 H, -*H*-12), 3.88 (s, 1 H, -*H*-12') ppm.

**$^{13}$C{$^1$H}-NMR** (125 MHz, acetone-$d_6$, 298 K): $\delta$ = 160.1 (*C*-4a), 144.5 (*C*-6a), 137.0 (*C*-10a), 132.4 (*C*-1), 131.8 (*C*-2), 129.1 (*C*-9), 124.0 (*C*-3), 123.5 (*C*-7), 122.6 (*C*-4), 121.7 (*C*-12a), 119.9 (*C*-10), 118.2 (*C*-8), 47.0 (*C*-12) ppm.

**IR** (ATR): $\tilde{\nu}$ = 3317 (s), 3026 (w), 2959 (w), 2878 (w), 1601 (m), 1587 (m), 1567 (w), 1511 (m), 1481 (s), 1455 (w), 1389 (m), 1363 (w), 1321 (s), 1272 (w), 1251 (s), 1191 (w), 1161 (m), 1139 (w), 1119 (w), 1093 (m), 1072 (m), 1034 (w), 956 (w), 898 (m), 831 (m), 812 (s), 740 (s), 720 (m), 702 (w), 651 (w), 603 (w) $cm^{-1}$.

**HR-MS** (ESI, DCM): *m/z* $[M+H]^+$ calculated for $C_{13}H_{10}N_3{}^{79}Br+H^+$: 288.01301; found: 288.0127 ± 1.44 ppm.

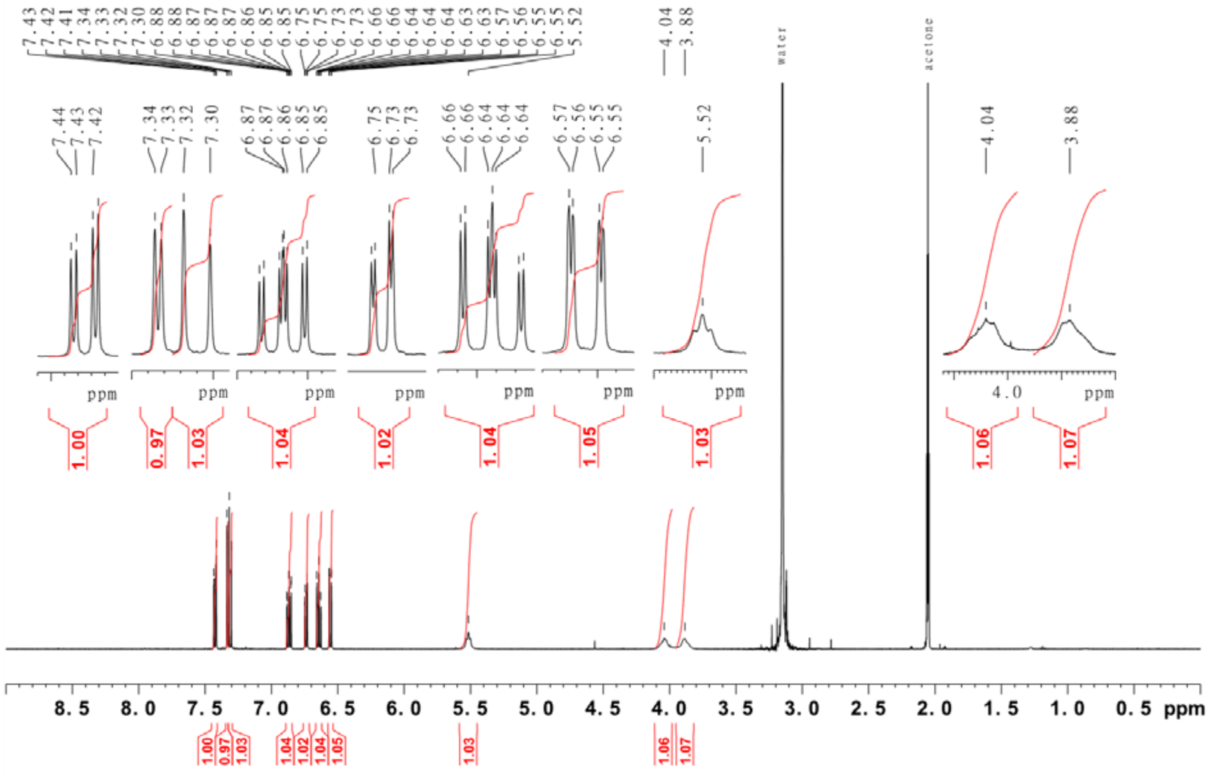

**Figure SII.9:** 500 MHz $^{1}$H-NMR spectrum of (*Z*)-bromo-11,12-dihydrodibenzo[*c,g*][1,2,5]triazocine.

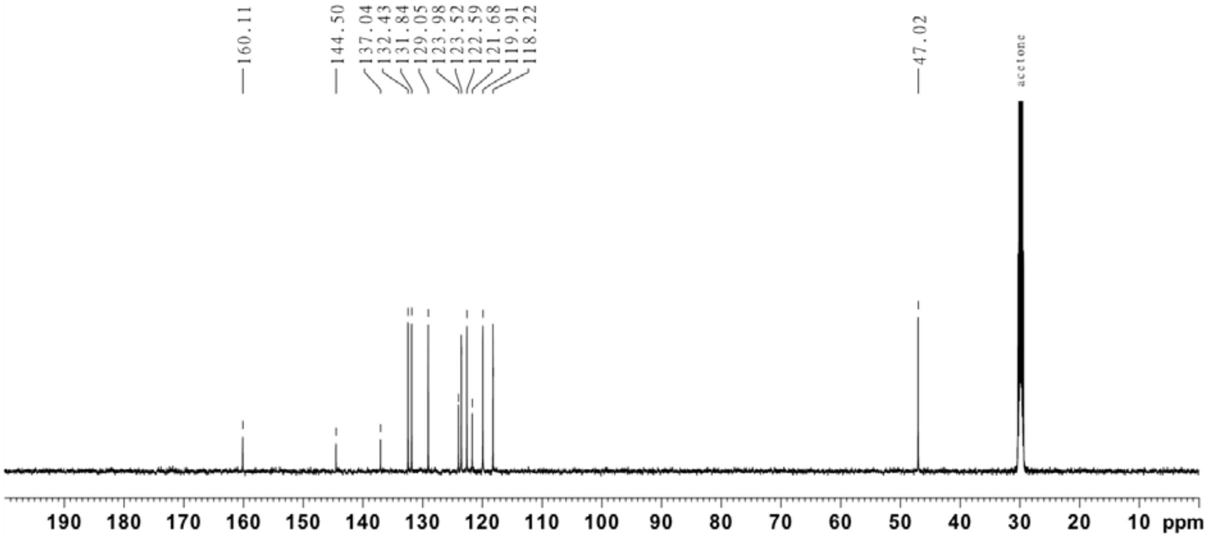

**Figure SII.10:** 125 MHz $^{13}$C{$^{1}$H}-NMR spectrum of (*Z*)-bromo-11,12-dihydrodibenzo[*c,g*][1,2,5]triazocine.

### II.1.6 Synthesis of (*Z*)-1-(3-bromodibenzo[*c,g*][1,2,5]triazocin-11(12*H*)-yl)-ethan-1-one (2)[1]

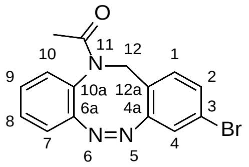

In a manner analogous to [1] (*Z*)-bromo-11,12-dihydrodibenzo[*c,g*][1,2,5]triazocine (500 mg, 1.8 mmol) were dissolved in 50 mL anhydrous DMF under a nitrogen atmosphere. $NEt_3$ (5 mL, 36 mmol) and AcOH (1.03 mL, 18 mmol) were added. The reaction mixture was cooled to 0 °C and T3P (10 mL, 18 mmol, 50% in ethyl acetate) was added dropwise. The reaction mixture was stirred at rt. for 16 h. 100 mL DCM and 100 mL deionized water were added and the organic layer was separated. The aqueous layer was extracted twice with 100 mL DCM and the combined organic layers were dried over $MgSO_4$. The solvent was removed and column chromatography on silica (ethyl acetate/cyclohexane 1:1, $R_f$ = 0.39) gave a yellow solid (492 mg, 1.49 mmol, 83%, lit. 86%[1]).

**melting point:** 163 °C

**$^1$H-NMR** (600 MHz, acetone-$d_6$, 298 K): $\delta$ = 7.40 (dd, $^3J$ = 7.4 Hz, $^4J$ = 1.6 Hz, 1 H, *H*-8/9), 7.34-7.27 (m, 3 H, *H*-2, *H*-7/10, *H*-8/9), 7.18 (d, $^3J$ = 8.2 Hz, 1 H, *H*-1), 7.11 (d, $^4J$ = 2.0 Hz, 1 H, *H*-4), 7.06 (dd, $^3J$ = 7.8 Hz, $^4J$ = 0.9 Hz, 1 H, *H*-10), 5.02 (d, $^2J$ = 14.8 Hz, 1 H, *H*-12a), 4.32 (d, $^2J$ = 14.7 Hz, 1 H, *H*-12b), 1.79 (s, 3 H, C*H*3) ppm.

**$^{13}$C{$^1$H}-NMR** (150 MHz, acetone-$d_6$, 198 K): $\delta$ = 169.4 (*C*=O), 157.2 (*C*-4a), 154.1 (*C*-6a/10a), 132.8 (*C*-1), 131.3 (*C*-2), 130.28 (*C*-8/9), 130.22 (*C*-8/9), 129.56 (*C*-7/10), 129.44 (*C*-6a/10a), 124.7 (*C*-12a), 122.7 (*C*-4), 122.3 (*C*-3), 120.0 (*C*-7/10), 51.5 (*C*-12), 23.0 ($CH_3$) ppm.

**IR** (ATR): $\tilde{\nu}$ = 3049 (m), 2928 (m), 1658 (s), 1590 (m), 1518 (w), 1475 (m), 1380 (s), 1338 (s), 1294 (m), 1242 (w), 1161 (m), 1115 (w), 1085 (m), 1031 (m), 1007 (w), 962 (m), 925 (w), 887 (w), 872 (w), 847 (m), 815 (s), 782 (w), 762 (s), 738 (m), 689 (w), 659 (m), 629 (w), 584s (w), 532 (w), 441 (s), 405 (m) $cm^{-1}$.

**HR-MS** (ESI, DCM): *m/z* $[M+H]^+$ calculated for $C_{15}H_{12}N_3O^{79}Br+H^+$: 330.0237; found: 330.0233 ± 1.18 ppm.

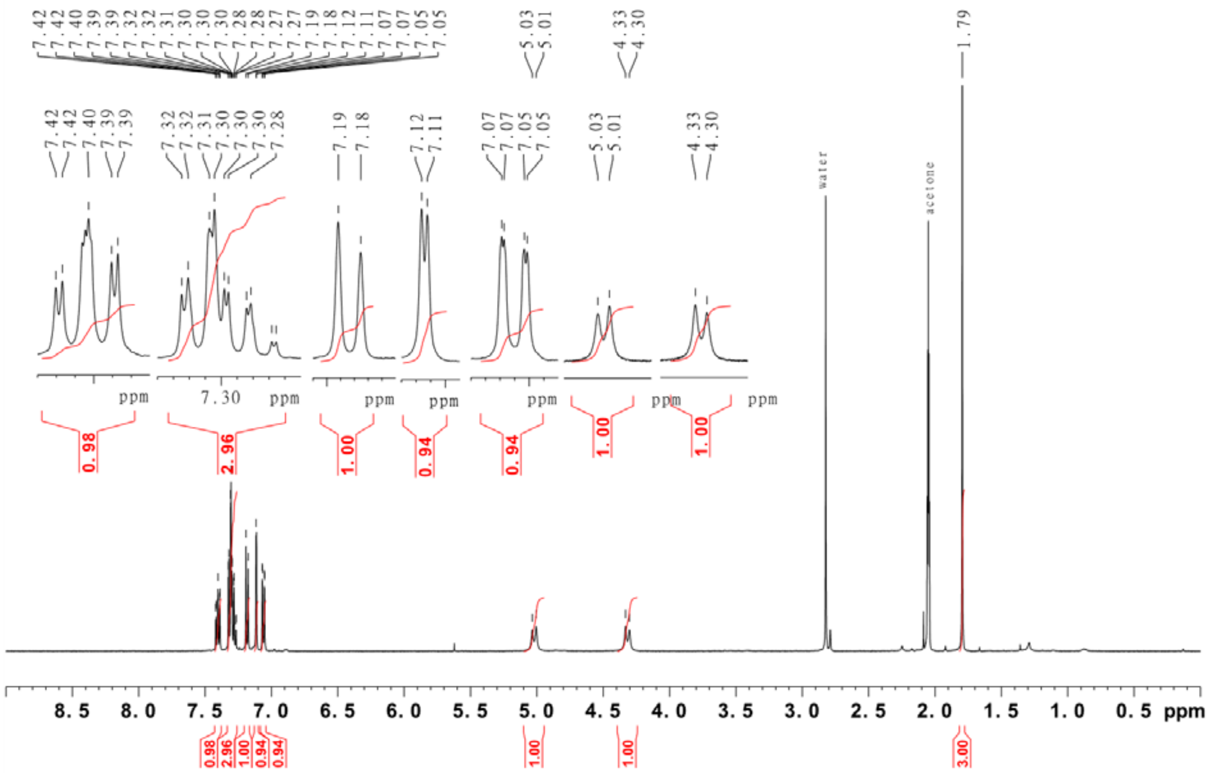


**Figure SII.11:** 600 MHz $^{1}$H-NMR spectrum of (*Z*)-1-(3-bromodibenzo[*c*,*g*][1,2,5]triazocin-11(12*H*)-yl)-ethan-1-one (**2**).

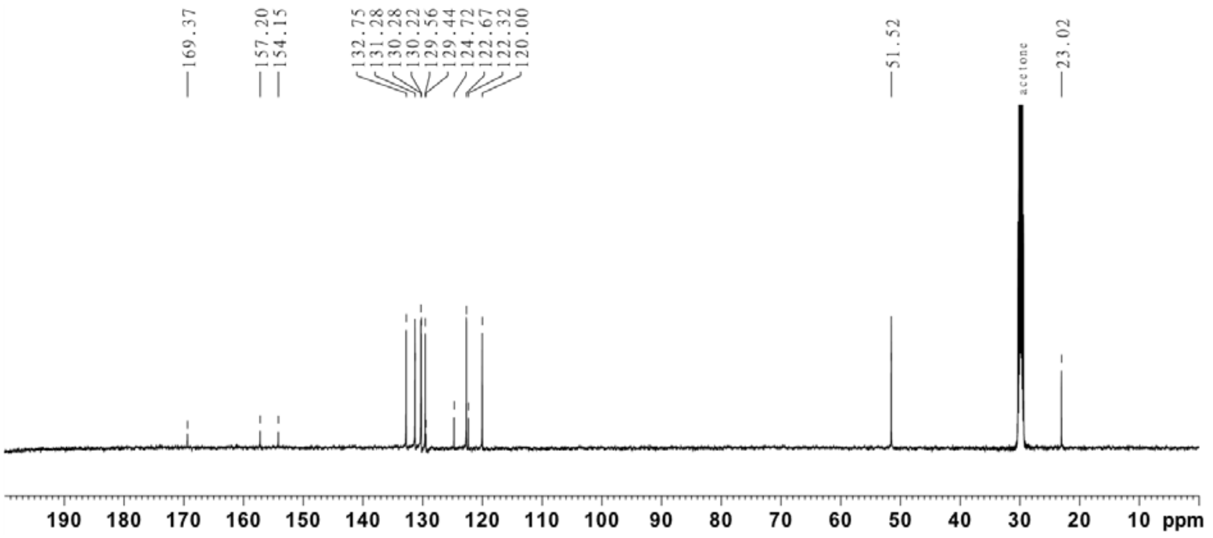


**Figure SII.12:** 150 MHz $^{13}$C{$^{1}$H}-NMR spectrum of (*Z*)-1-(3-bromodibenzo[*c*,*g*][1,2,5]triazocin-11(12*H*)-yl)-ethan-1-one (**2**).

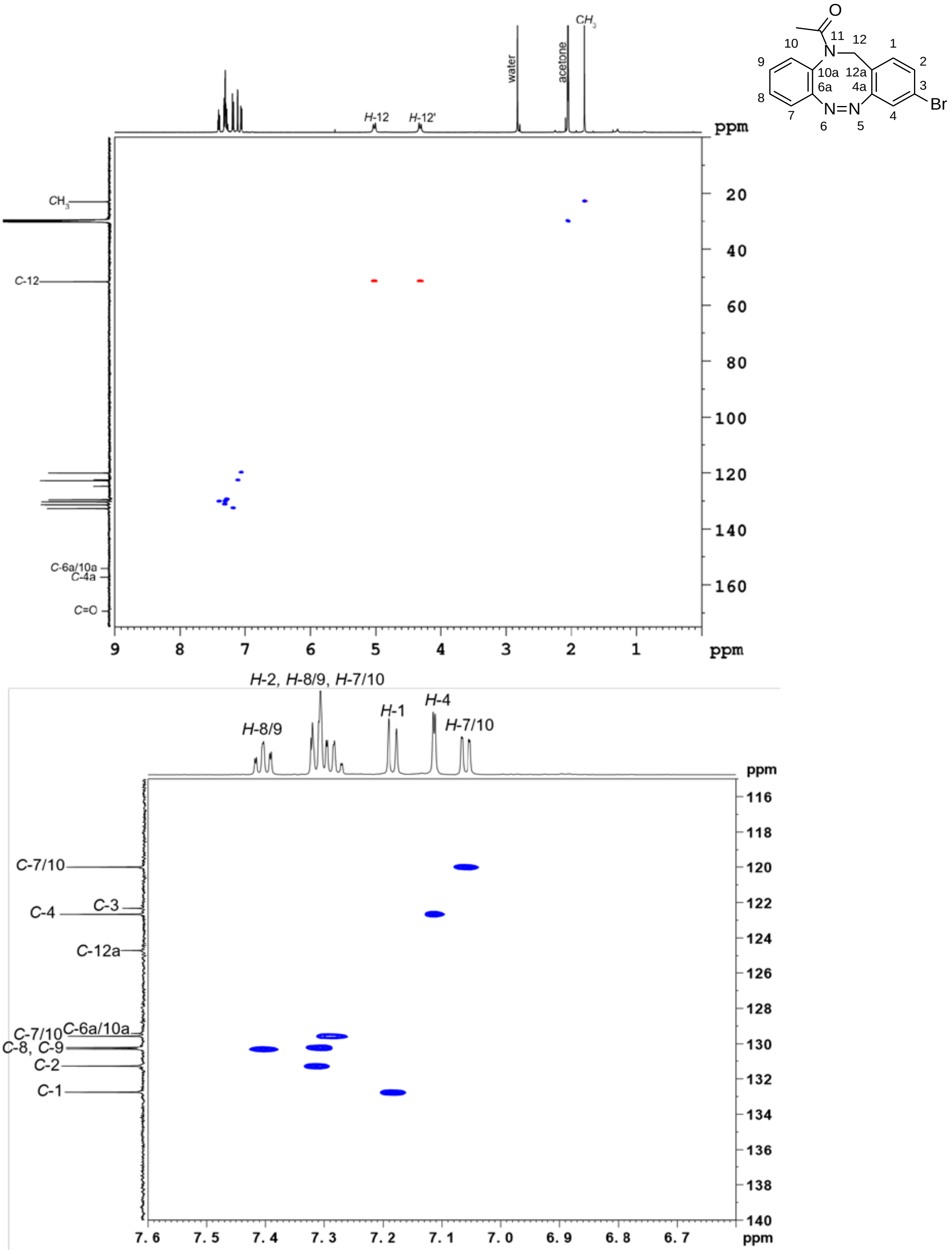


**Figure SII.13:** 600 MHz $^{1}H$-$^{13}C$ HSQC spectrum of (*Z*)-1-(3-bromodibenzo[*c*,*g*][1,2,5]triazocin-11(12*H*)-yl)-ethan-1-one (**2**).

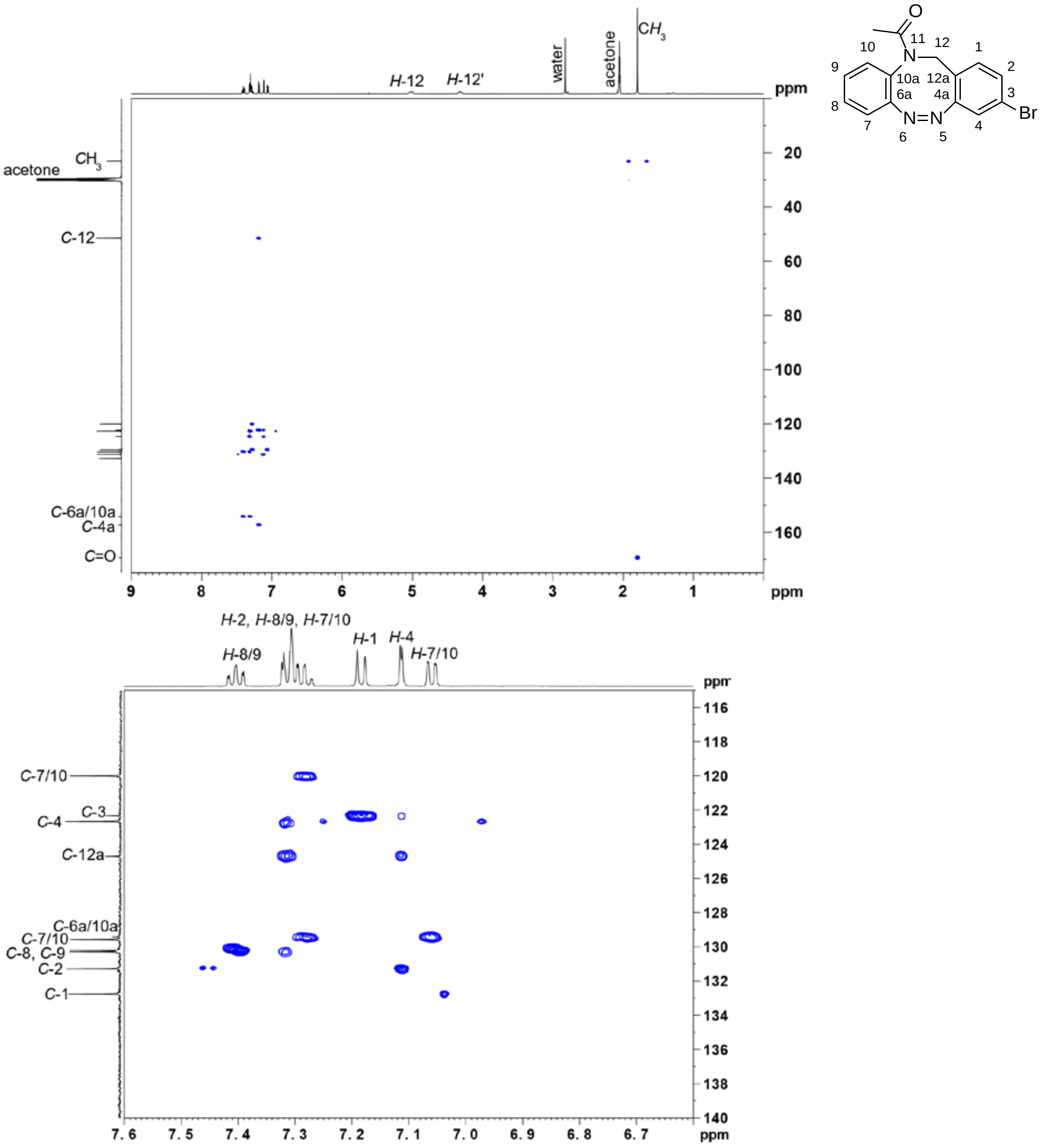


**Figure SII.14:** 600 MHz HMBC spectrum of (*Z*)-1-(3-bromodibenzo[*c*,*g*][1,2,5]triazocin-11(12*H*)-yl)-ethan-1-one (**2**).

## II.1.7 Synthesis of *tert*-butyl (2-((4-iodo-2-nitrobenzyl)amino)phenyl)carbamate[1]

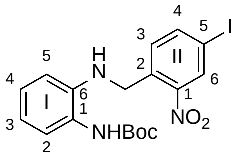

In a manner analogous to [1] triethylamine (4.25 mL, 24.9 mmol) and 1-(bromomethyl)-4-iodo-2-nitrobenzene (7.81 g, 22.9 mmol) were added to solution of tert-butyl (2-aminophenyl)carbamate (4.77 g, 22.9 mmol) in 100 mL dry THF under a nitrogen atmosphere. The reaction mixture was heated to reflux with an oil bath and stirred for 16 h at that temperature. Afterwards the solvent was evaporated and 100 mL of deionized water and 100 mL DCM were added to the residue. The organic layer was separated and the aqueous layer was extracted twice with 50 mL of DCM. The combined organic layers were dried over $MgSO_4$ and the solvent was evaporated. Recrystallization from cyclohexane/ethyl acetate (1:1) gave the product as yellow solid (7.39 g, 15.7 mmol, 69%, lit. 96%[1]).

**melting point:** 140 °C

**$^1$H-NMR** (500 MHz, $CDCl_3$, 298 K): $\delta$ = 8.37 (d, $^4J$ = 1.7 Hz, 1 H, Ar$^{II}$-*H*-3), 7.85 (dd, $^3J$ = 8.3 Hz, $^4J$ = 1.6 Hz, 1 H, Ar$^{II}$-*H*-5), 7.38 (d, $^3J$ = 8.2 Hz, 1 H, Ar$^{II}$-*H*-6), 7.24 (d, $^3J$ = 7.6 Hz, 1 H, Ar$^{I}$-*H*-6), 7.00 (td, $^3J$ = 8.0 Hz, $^4J$ = 1.3 Hz, 1 H, Ar$^{I}$-*H*-4), 6.75 (t, $^3J$ = 7.6 Hz, 1 H, Ar$^{I}$-*H*-5), 6.43 (d, $^3J$ = 8.0 Hz, 1 H, Ar$^{I}$-*H*-3), 6.18 (br. s, 1 H, N*H*), 4.68 (s, 2 H, C$H_2$), 1.51 (s, 9 H, *t*Bu-*H*) ppm.

**$^{13}$C{$^1$H}-NMR** (125 MHz, $CDCl_3$, 298 K): $\delta$ = 154.4 (*C*=O), 148.6 (Ar$^{II}$-*C*-2), 142.7 (Ar$^{II}$-*C*-5), 141.5 (Ar$^{I}$-*C*-2), 135.3 (Ar$^{II}$-*C*-1), 133.8 (Ar$^{II}$-*C*-3), 131.5 (Ar$^{II}$-*C*-6), 127.1 (Ar$^{I}$-*C*-4), 126.0 (Ar$^{I}$-*C*-6), 124.3 (Ar$^{I}$-*C*-1), 118.9 (Ar$^{I}$-*C*-5), 112.9 (Ar$^{I}$-*C*-3), 91.6 (Ar$^{II}$-*C*-4), 81.0 (-*C*-$(CH_3)_3$), 45.4 (-*C*$H_2$), 28.5 (-*C*$H_3$), ppm.

**IR** (ATR): $\tilde{\nu}$ = 3374 (m), 2974 (m), 2931 (w), 2169 (w), 2051 (w), 1978 (w), 1684 (m), 1605 (m), 1509 (s), 1446 (m), 1391 (w), 1366 (w), 1342 (m), 1307 (w), 1272 (w), 1244 (m), 1152 (s), 1047 (m), 1024 (m), 934 (w), 869 (w), 823 (w), 743 (s), 715 (w), 637 (w) $cm^{-1}$..

**HR-MS** (ESI, DCM): *m/z* $[M+H]^+$ calculated for $C_{18}H_{20}O_4N_3{}^{127}I+H^+$: 470.0571; found: 470.0572 ± 0.08 ppm.

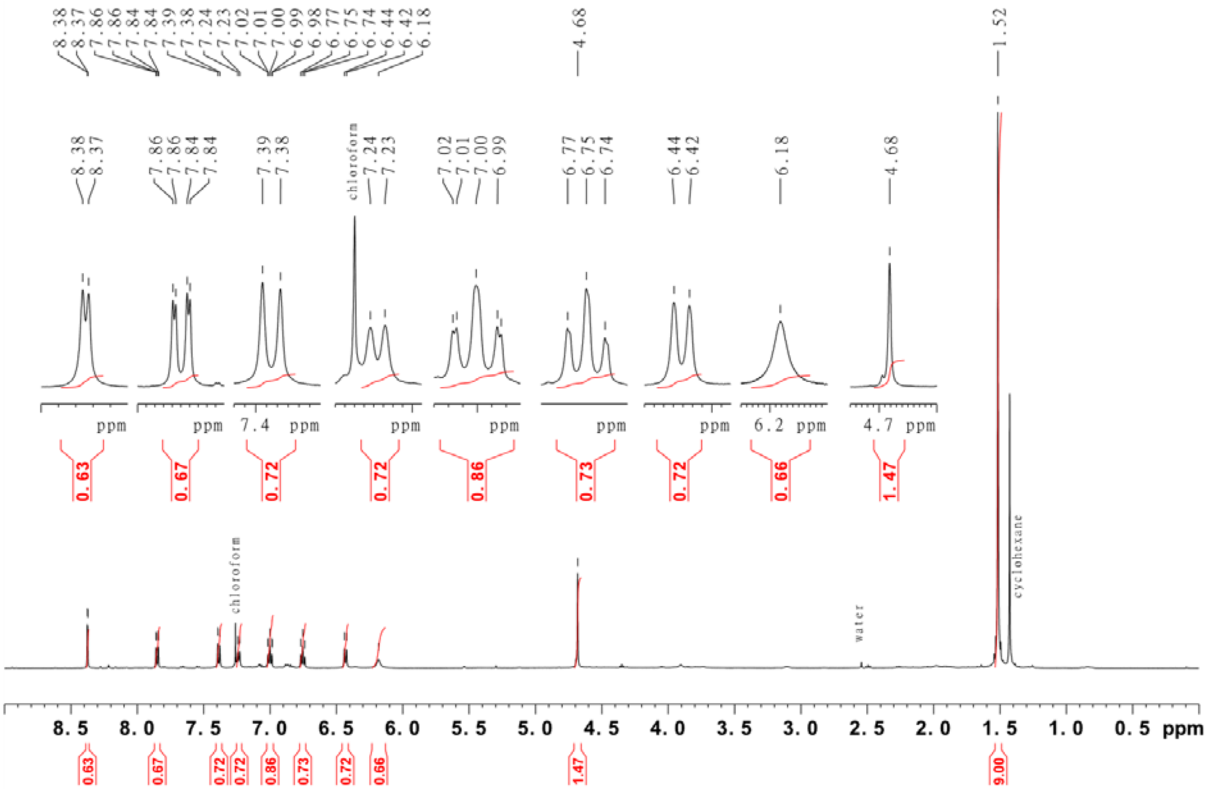


**Figure SII.15:** 500 MHz $^{1}H$-NMR spectrum of *tert*-butyl (2-((4-iodo-2-nitrobenzyl)amino)phenyl)carbamate.

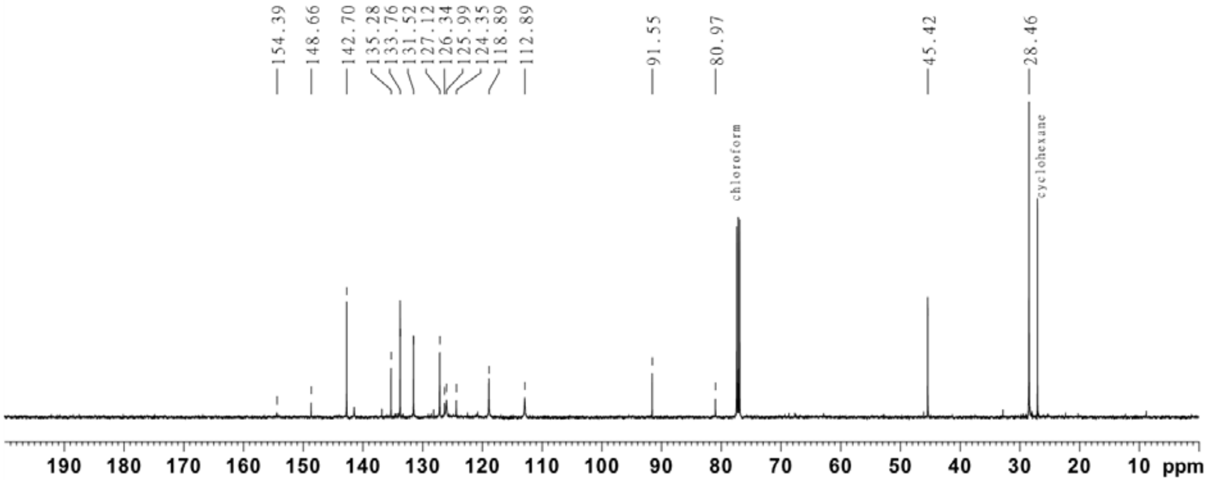


**Figure SII.16:** 125 MHz $^{13}C\{^{1}H\}$-NMR spectrum of *tert*-butyl (2-((4-iodo-2-nitrobenzyl)amino)phenyl)carbamate.

## II.1.8 Synthesis of (9*H*-fluoren-9-yl)methyl (4-iodo-2-nitrobenzyl)(2-((*tert*-butoxycarbonyl)amino)phenyl)carbamate[1]

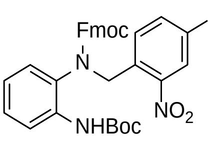


In a manner analogous to [1] DIPEA (2.67 mL, 15.7 mmol) and 9-Fluorenylmethoxycarbonyl chlorid (8.12 g, 31.4 mmol) were added to a solution of *tert*-butyl (2-((4-iodo-2-nitrobenzyl)amino)phenyl)carbamate (7.39 g, 15.7 mmol) in 50 mL dry DMF under a nitrogen atmosphere. The reaction mixture was stirred for 48 h at rt. and the solvent was evaporated subsequently. 100 mL of deionized water and 100 mL of DCM were added to the residue. The organic layer was separated and the aqueous layer was extracted twice with 100 mL DCM. The combined organic layers were dried over $MgSO_4$ and the solvent was evaporated. Column chromatography on silica (ethyl acetate/cyclohexane 1:4, $R_f$ = 0.48) gave a slightly yellow solid (6.66 g, 9.63 mmol, 62%, lit. 33%[1][1]).

**melting point:** 127 °C

**$^1$H-NMR** (600 MHz, acetone-$d_6$, 298 K): $\delta$ = 8.29 (d, $^4J$ = 1.8 Hz, 1 H, Ar-*H*), 8.08-7.70 (m, 5 H), 7.40-7.02 (m, 10 H), 5.18-4.93 (m, 2 H), 4.53-4.01 (m, 3 H), 1.45 (s, 9 H, *t*Bu-*H*) ppm.

**$^{13}$C{$^1$H}-NMR** (150 MHz, acetone-$d_6$, 298 K): $\delta$ = 153.5, 143.0, 133.9, 129.2, 128.5, 127.9, 126.1, 124.4, 120.7, 92.7, 80.4, 68.4, 47.8, 28.5 ppm.

**IR** (ATR): $\tilde{\nu}$ = 3314 (w), 3066 (w), 2051 (w), 2024 (w), 2012 (w), 1709 (s), 1595 (m), 1525 (s), 1477 (w), 1449 (s), 1401 (m), 1345 (m), 1296 (m), 1216 (m), 1155 (m), 1043 (m), 981 (w), 940 (w), 870 (m), 838 (w), 757 (w), 749 (w), 738 (s), 620 (m), 573 (w), 538 (m), 494 (w) $cm^{-1}$.

**HR-MS** (ESI, DCM): *m/z* $[M+H]^+$ calculated for $C_{30}H_{30}O_6N_3{}^{127}I+H^+$: 692.1252; found: 692.1259 ± 1.00 ppm.

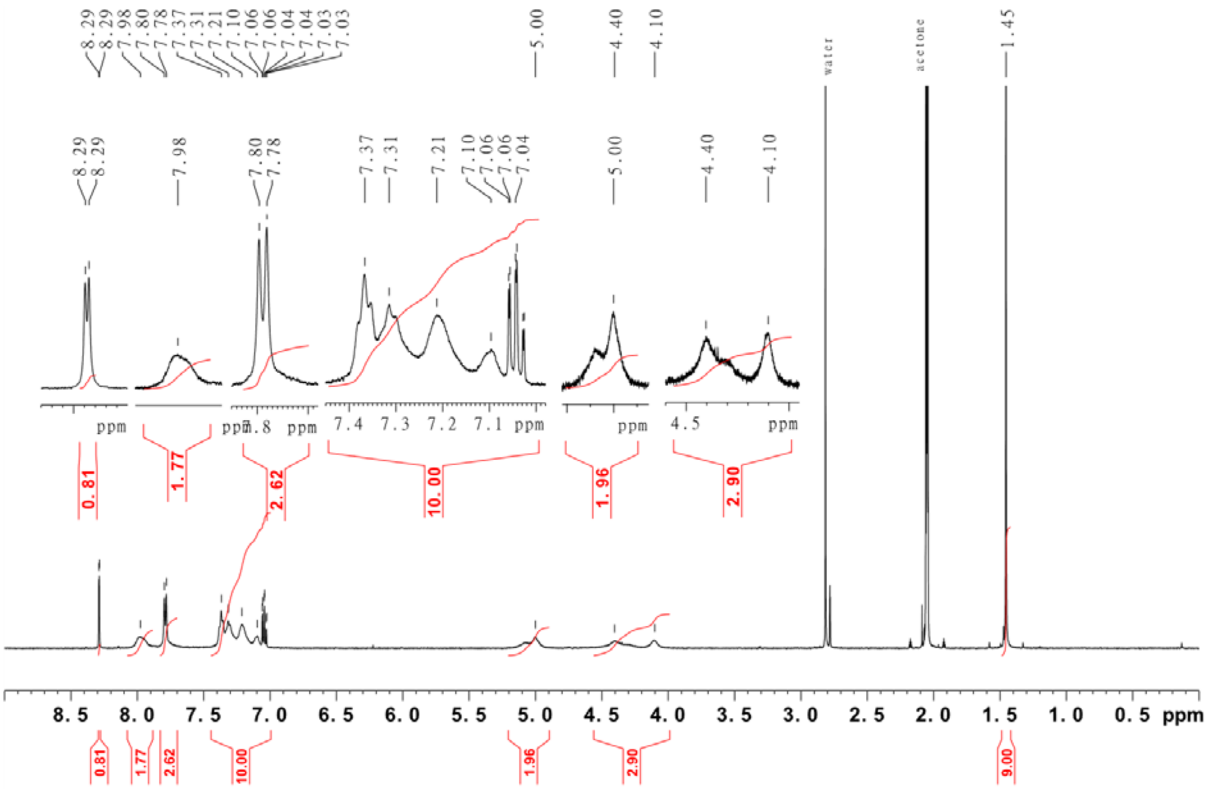

**Figure SII.17:** 600 MHz $^{1}$H-NMR spectrum of (9*H*-fluoren-9-yl)methyl (4-iodo-2-nitrobenzyl)(2-((*tert*-butoxycarbonyl)amino)phenyl)carbamate.

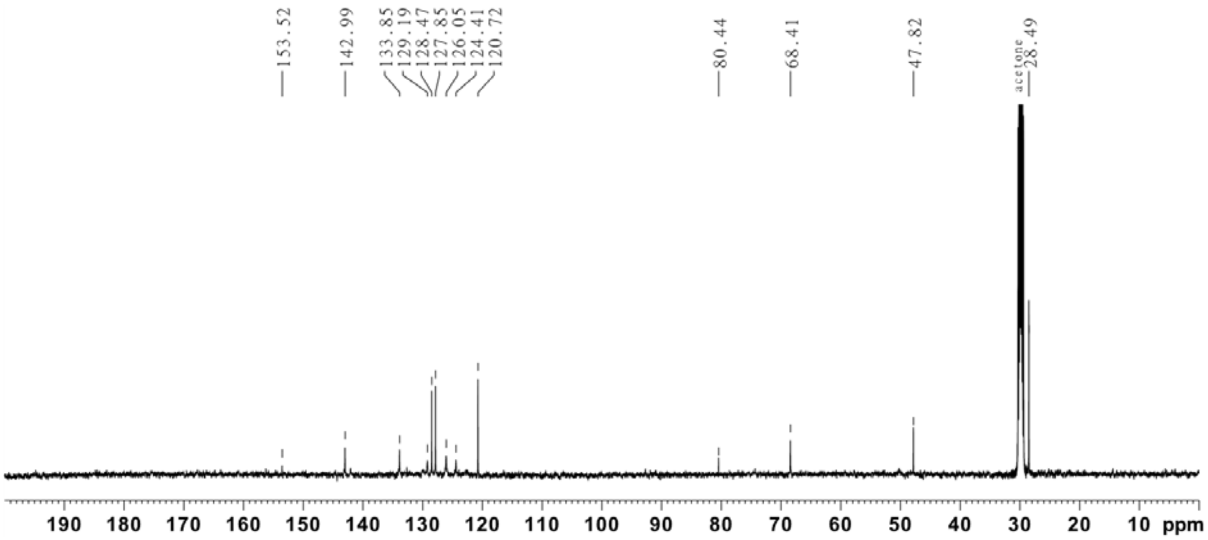

**Figure SII.18:** 150 MHz $^{13}$C{$^{1}$H}-NMR spectrum of (9*H*-fluoren-9-yl)methyl (4-iodo-2-nitrobenzyl)(2-((*tert*-butoxycarbonyl)amino)phenyl)carbamate.

### II.1.9 Synthesis of (9*H*-fluoren-9-yl)methyl (2-aminophenyl)(4-iodo-2-nitrobenzyl)carbamate[1]

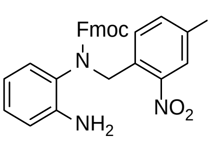


In a manner analogous to [1] (9*H*-fluoren-9-yl)methyl (4-iodo-2-nitrobenzyl)(2-((*tert*-butoxycarbonyl)amino)phenyl)carbamate (6.66 g, 9.63 mol) was dissolved in 100 mL DCM and 30 mL TFA was added. The reaction mixture was stirred at rt. for 16 h and neutralized with saturated aqueous $NaHCO_3$ subsequently. The organic layer was separated and the aqueous layer was extracted twice with 100 mL. The combined organic layers were dried over $MgSO_4$ and the solvent was evaporated to obtain a pale yellow solid (5.74 g, 9.60 mmol, quant., lit. 96%[1]) which was used without further purification.

**melting point:** 86 °C

**$^1$H-NMR** (500 MHz, $CDCl_3$, 298 K): $\delta$ = 8.24 (d, $^4J$ = 1.5 Hz, 1 H), 7.87 (br. s, 1 H), 7.72-7.62 (m, 2 H), 7.38-7.32 (m, 3 H), 7.22-7.10 (m, 5 H), 6.78-6.72 (m, 2 H), 6.69-6.65 (m, 1 H), 5.21 (br. s, 1 H, -C*H*$_2$), 5.04-3.98 (m, 4 H) ppm.

**$^{13}$C{$^1$H}-NMR** (125 MHz, $CDCl_3$, 298 K): $\delta$ = 155.2, 143.6, 142.3, 141.4, 133.5, 132.4, 129.2, 128.8, 127.8, 127.1, 125.3, 120.0, 119.0, 92.16, 68.0, 47.2 ppm.

**IR** (ATR): $\tilde{\nu}$ = 3370 (w), 3039 (w), 2252 (w), 2168 (w), 1978 (w), 1695 (s), 1620 (m), 1525 (s), 1503 (w), 1477 (w), 1449 (m), 1402 (w), 1339 (m), 1297 (m), 1156 (w), 1136 (w), 1043 (w), 987 (w), 870 (m), 795 (w), 756 (w), 738 (s), 692 (w), 620 (m), 534 (w), 501 (w), 480 (w) cm$^{-1}$.

**HR-MS** (ESI, DCM): *m/z* [M+H]$^+$ calculated for $C_{28}H_{22}O_4N_3{}^{127}I+H^+$: 592,0729; found: 592.0732 ± 0.78 ppm.

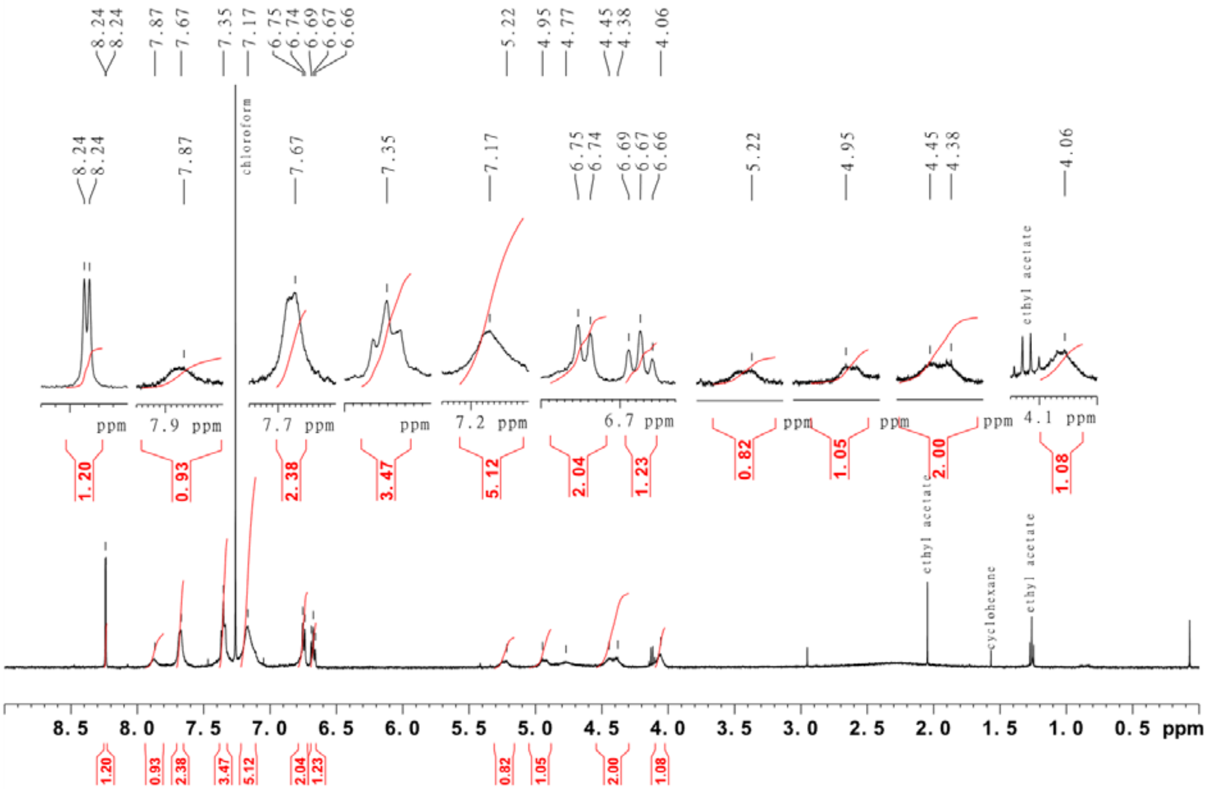

**Figure SII.19:** 500 MHz $^{1}$H-NMR spectrum of (9*H*-fluoren-9-yl)methyl (2-aminophenyl)(4-iodo-2-nitrobenzyl)carbamate.

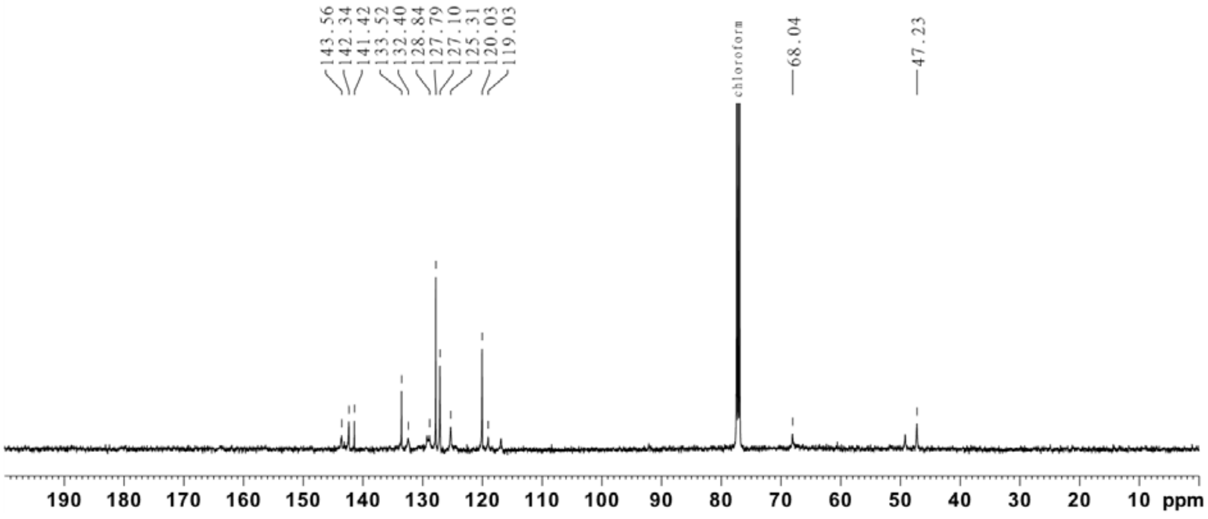

**Figure SII.20:** 125 MHz $^{13}$C{$^{1}$H}-NMR spectrum of (9*H*-fluoren-9-yl)methyl (2-aminophenyl)(4-iodo-2-nitrobenzyl)carbamate.

## II.1.10 Synthesis of (9*H*-fluoren-9-yl)methyl (*Z*)-3-iododibenzo[*c,g*][1,2,5]triazocin-11-(12*H*)-carboxylate[1]

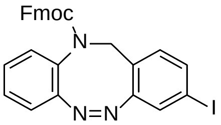


In a manner analogous to [1] (9*H*-fluoren-9-yl)methyl (2-aminophenyl)(4-iodo-2-nitrobenzyl)carbamate (5.74 g, 9.71 mmol) was suspended in 250 mL EtOH and $SnCl_2 \cdot 2\,H_2O$ (13.2 g, 46.1 mmol) was added. The reaction mixture was heated to 75 °C with an oil bath and zinc-powder (5.08 g, 77.7 mmol) was added. The reaction mixture was stirred at that temperature for 1 h and then cooled to rt. It was stirred at rt. for 16 h and neutralized with saturated aqueous $NaHCO_3$ solution subsequently. The solvent was evaporated and 100 mL water and 100 mL DCM were added to the residue. The organic layer was separated and the aqueous layer was extracted twice with 100 mL DCM. The combined organic layers were dried over $MgSO_4$ and the solvent was evaporated. The residue was dissolved in 300 mL of acetic acid and *m*CPBA (3.35 g, 19.42 mmol) dissolved in 300 mL acetic acid was added dropwise to the reaction mixture. The reaction mixture was stirred at rt. for 16 h and the solvent was evaporated in the following. 100 mL DCM and 100 mL half-concentrated aqueous $NaHCO_3$ solution were added to the residue. The organic layer was separated and the aqueous layer was extracted twice with 100 mL DCM. The combined organic layers were dried over $MgSO_4$ and the solvent was evaporated. After column chromatography on silica (ethyl acetate/cyclohexane = 1:4, $R_f$ = 0.38) the product could be obtained as yellow solid (2.59 g, 4.66 mmol, 48%, lit. 56%[1]).

**melting point:** 76 °C

**$^{1}$H-NMR** (500 MHz, $CDCl_3$, 298 K): $\delta$ = 7.97-6.98 (m, 15 H, Ar-*H*), 4.99-3.99 (m, 5 H, aliph. H), ppm.

**$^{13}$C{$^{1}$H}-NMR** (125 MHz, $CDCl_3$, 298 K): $\delta$ = 154.4, 152.6, 144.7, 142.1, 137.4, 132.6, 130.5, 129.5, 129.1 128.54, 128.36, 127.9, 126.1, 124.8, 120.8, 119.7, 93.4, 68.5, 52.8, 47.8 ppm.

**IR** (ATR): $\tilde{\nu}$ = 3071 (w), 2945 (w), 2255 (w), 2037 (w), 1700 (s), 1585 (m), 1485 (m), 1448 (s), 1315 (s), 1130 (s), 1072 (m), 1041 (m), 1021 (m), 976 (w), 878 (w), 863 (m), 820 (m), 753 (s), 735 (s), 696 (m), 670 (w), 640 (w), 546 (s), 512 (m), 487 (w), 464 (w), 434 (m) $cm^{-1}$.

**HR-MS** (ESI, DCM): *m/z* $[M+H]^+$ calculated for $C_{28}H_{20}O_2N_3{}^{127}I+H^+$: 558.0673; found: 558.0667 ± 1.09 ppm.

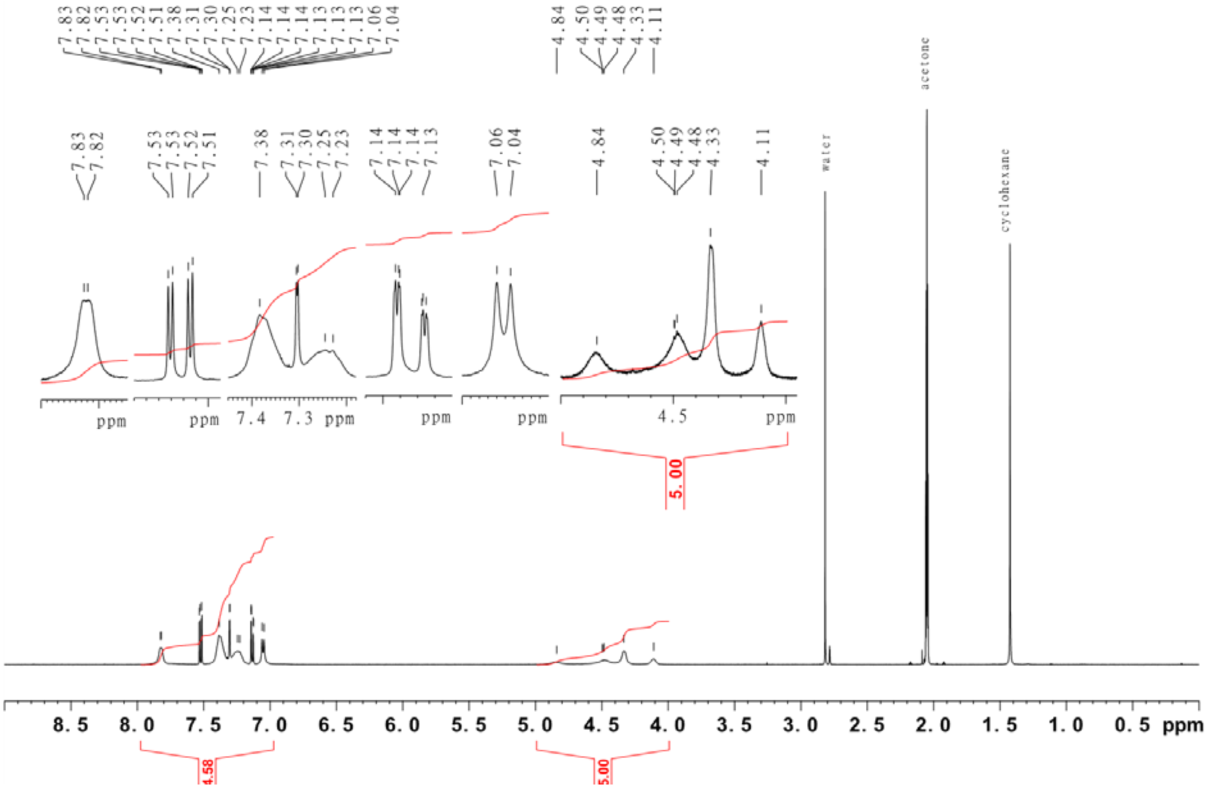


**Figure SII.21:** 500 MHz $^{1}H$-NMR spectrum of (9*H*-fluoren-9-yl)methyl (*Z*)-3-iododibenzo[*c*,*g*][1,2,5]triazocin-11-(12*H*)-carboxylate.

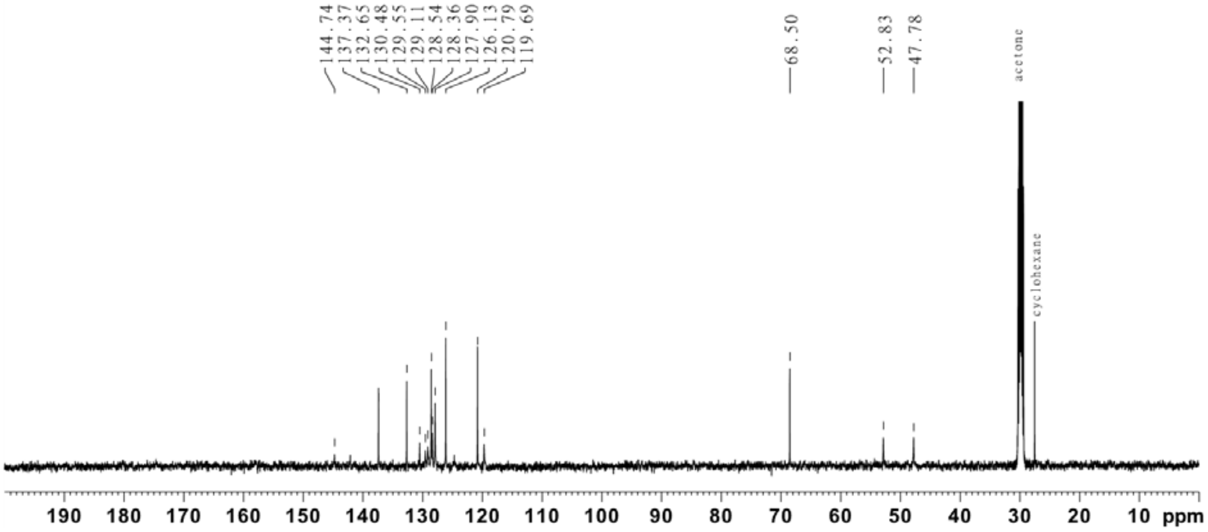


**Figure SII.22:** 125 MHz $^{13}C\{^{1}H\}$-NMR spectrum of (9*H*-fluoren-9-yl)methyl (*Z*)-3-iododibenzo[*c*,*g*][1,2,5]triazocin-11-(12*H*)-carboxylate.

## II.1.11 Synthesis of (*Z*)-3-iodo-11,12-dihydrodibenzo[*c,g*][1,2,5]triazocine[1]

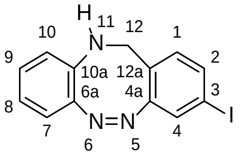


In a manner analogous to [1] (9*H*-fluoren-9-yl)methyl-(*Z*)-3-iododibenzo[*c,g*][1,2,5]triazocin-11-(12*H*)-carboxylate (2.09 g, 3.75 mmol) was dissolved in 50 mL of DCM and 50 mL $NEt_3$ was added. The reaction mixture was stirred at rt. for 16 h. the solvent was evaporated and column chromatography on silica (ethyl acetate/cyclohexane 1:3, $R_f$ = 0.25) gave a red solid (1.25 g, 3.72 mmol, quant., lit. 84%[1]).

**melting point:** 151 °C

**$^1$H-NMR** (500 MHz, acetone-$d_6$, 298 K): $\delta$ = 7.63 (dd, $^3J$ = 8.0 Hz, $^4J$ = 1.8 Hz, 1 H, *H*-2), 7.52 (d, $^4J$ = 1.6 Hz, 1 H, *H*-4), 7.16 (d, $^3J$ = 7.8 Hz, 1 H, *H*-1), 6.88 (td, $^3J$ = 8.3 Hz, $^4J$ = 1.6 Hz, 1 H, *H*-9), 6.75 (dd, $^3J$ = 8.0 Hz, $^4J$ = 1.6 Hz, 1 H, *H*-7), 6.65 (td, $^3J$ = 7.9 Hz, $^4J$ = 1.2 Hz, 1 H, *H*-8), 6.56 (dd, $^3J$ = 8.2 Hz, $^4J$ = 1.0 Hz, 1 H, *H*-10), 5.42 ($m_c$, 1 H, -N*H*), 3.97 ($m_c$, $^2$ H, -C$H_2$) ppm.

**$^{13}$C{$^1$H}-NMR** (125 MHz, acetone-$d_6$, 298 K): $\delta$ = 160.3 (*C*-4a), 144.6 (*C*-6a), 137.9 (*C*-2), 137.1 (*C*-10a), 131.8 (*C*-1), 129.0 (*C*-9), 128.5 (*C*-4), 124.5 (*C*-12a), 123.6 (*C*-7), 119.9 (*C*-10), 118.3 (*C*-8), 93.1 (*C*-3), 47.2 ($CH_2$) ppm.

**IR** (ATR): $\tilde{\nu}$ = 3381 (m), 3042 (w), 2255 (w), 2169 (w), 1597 (m), 1582 (m), 1514 (w), 1479 (s), 1385 (w), 1315 (m), 1246 (w), 1161 (m), 1145 (w), 1117 (w), 1066 (m), 970 (w), 938 (w), 912 (m), 875 (w), 835 (m), 810 (m), 749 (s), 721 (w), 703 (w), 650 (w), 601 (m), 543 (w), 501 (w), 466 (m), 454 (w) $cm^{-1}$.

**HR-MS** (ESI, DCM): *m/z* $[M+H]^+$ calculated for $C_{13}H_{10}N_3{}^{127}I+H^+$: 335.9992; found: 335.9986 ± 0.57 ppm.

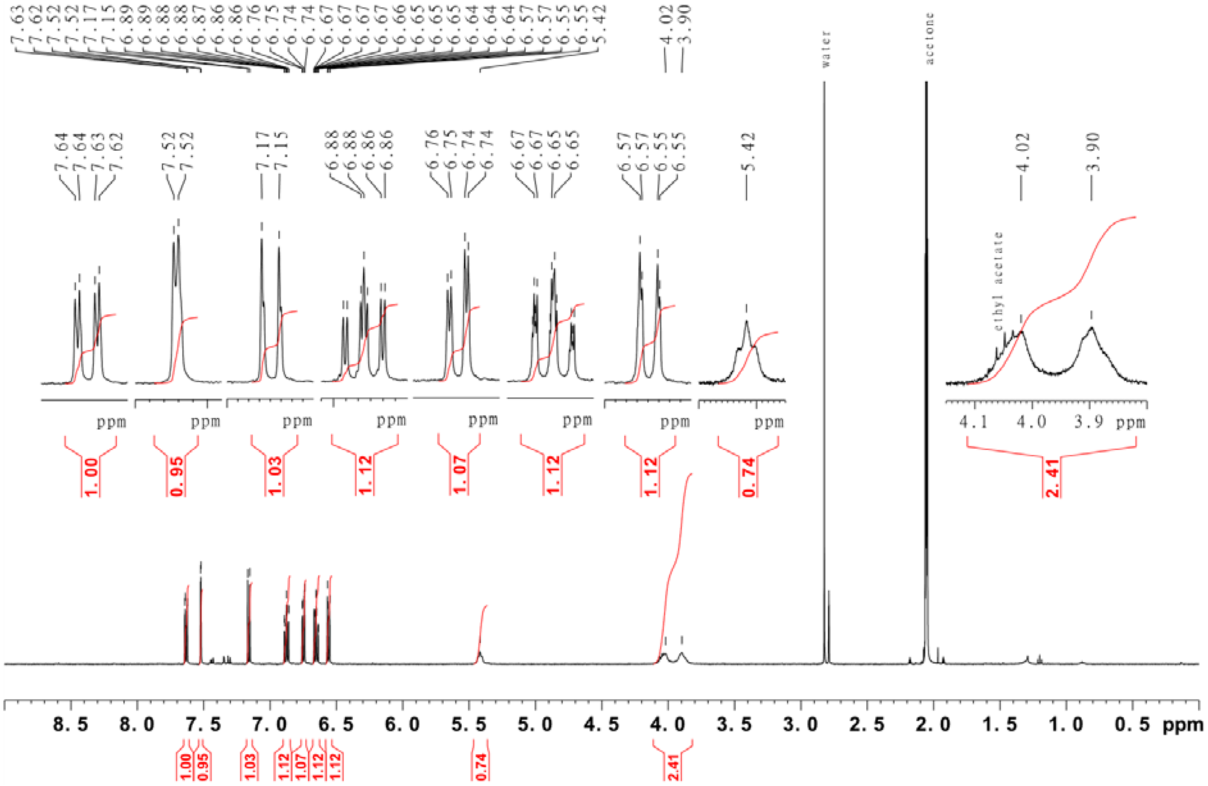

**Figure SII.23:** 500 MHz $^{1}H$-NMR spectrum of (*Z*)-3-iodo-11,12-dihydrodibenzo[*c*,*g*][1,2,5]triazocine.

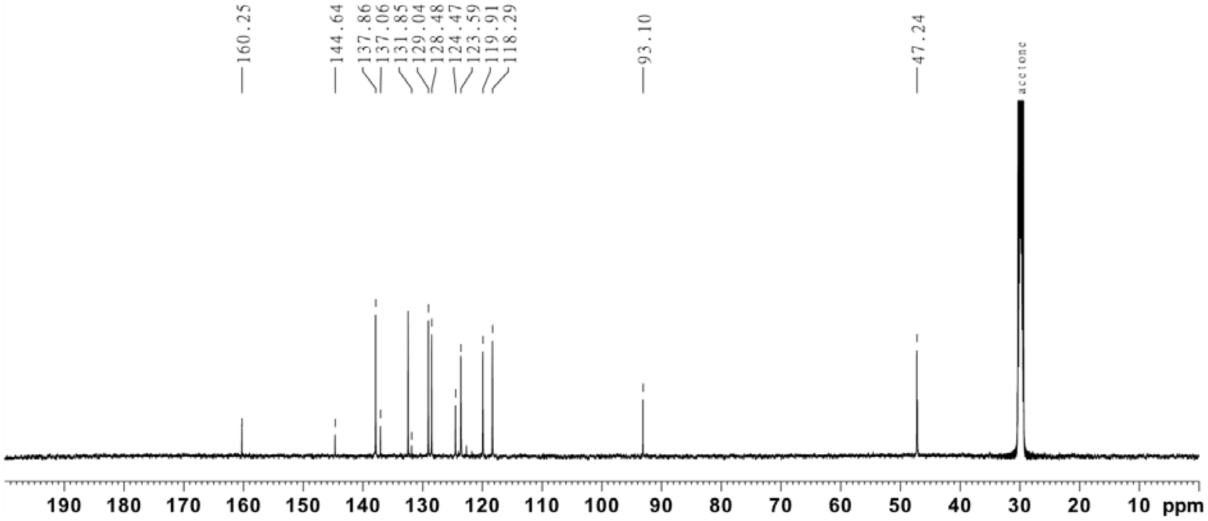

**Figure SII.24:** 125 MHz $^{13}C\{^{1}H\}$-NMR spectrum of (*Z*)-3-iodo-11,12-dihydrodibenzo[*c*,*g*][1,2,5]triazocine.

## II.1.12 Synthesis of (*Z*)-1-(3-iododibenzo[*c,g*][1,2,5]triazocin-11(12*H*)-yl)-ethan-1-one (3)[1]

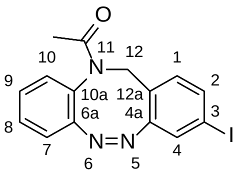


In a manner analogous to [1] (*Z*)-3-iodo-11,12-dihydrodibenzo[*c,g*][1,2,5]triazocine (603 mg, 1.8 mmol) were dissolved in 50 mL anhydrous DMF under a nitrogen atmosphere. $NEt_3$ (2.5 mL, 3.6 mmol) and AcOH (1.03 mL, 18 mmol) were added. The reaction mixture was cooled to 0 °C and T3P (10 mL, 18 mmol, 50% in ethyl acetate) was added dropwise. The reaction mixture was stirred at rt. for 16 h. 100 mL DCM and 100 mL deionized water were added and the organic layer was separated. The aqueous layer was extracted twice with 100 mL DCM and the combined organic layers were dried over $MgSO_4$. The solvent was removed and column chromatography on silica (ethyl acetate/cyclohexane 1:1, $R_f$ = 0.39) gave a yellow solid (476 mg, 1.26 mmol, 70%, lit. 60%[1]).

**melting point:** 179 °C

**$^1$H-NMR** (600 MHz, acetone-$d_6$, 298 K): $\delta$ = 7.51 (dd, $^3J$ = 8.0 Hz, $^4J$ = 1.8 Hz, 1 H, *H*-2), 7.40 (td, $^3J$ = 7.5 Hz, $^4J$ = 1.6 Hz, 1 H, *H*-8/9), 7.32-7.26 (m, 3 H, *H*-4, *H*-7/10, *H*-8/9), 7.06 (dd, $^3J$ = 7.8 Hz, $^4J$ = 1.1 Hz, 1 H, *H*-7/10), 7.03 (d, $^3J$ = 8.1 Hz, 1 H, *H*-1), 5.02 (d, $^2J$ = 14.6 Hz, 1 H, *H*-12), 4.29 (d, $^2J$ = 14.7 Hz, 1 H, *H*-12'), 1.79 (s, 3 H, C$H_3$), ppm.

**$^{13}$C{$^1$H}-NMR** (150 MHz, acetone-$d_6$, 298 K): $\delta$ = 169.4 (*C*=O), 157.1 (*C*-4a), 154.2 (*C*-6a/10a), 137.4 (*C*-2), 132.7 (*C*-1), 130.28 (*C*-7/10), 130.18 (*C*-8/9), 129.54 (*C*-8/9), 129.45 (*C*-6a/10a), 128.4 (*C*-4), 125.2 (*C*-12a), 120.0 (*C*-7/10), 93.5 (*C*-3), 51.6 (*C*-12), 23.0 ($CH_3$) ppm.

**IR** (ATR): $\tilde{\nu}$ = 3057 (w), 3010 (w), 2930 (w), 2021 (w), 1654 (s), 1582 (m), 1554 (w), 1513 (w), 1473 (m), 1432 (w), 1377 (s), 1336 (s), 1296 (m), 1250 (w), 1110 (m), 1084 (m), 1030 (m), 1009 (w), 970 (m), 917 (w), 842 (m), 823 (s), 767 (s), 735 (w), 652 (m), 598 (m), 582 (m), 514 (m), 485 (m), 470 (w), 437 (s) $cm^{-1}$.

**HR-MS** (ESI, DCM): *m/z* $[M+H]^+$ calculated for $C_{15}H_{11}ON_3{}^{127}I+H^+$: 378.0098; found: 378.0090 ± 2.03 ppm.

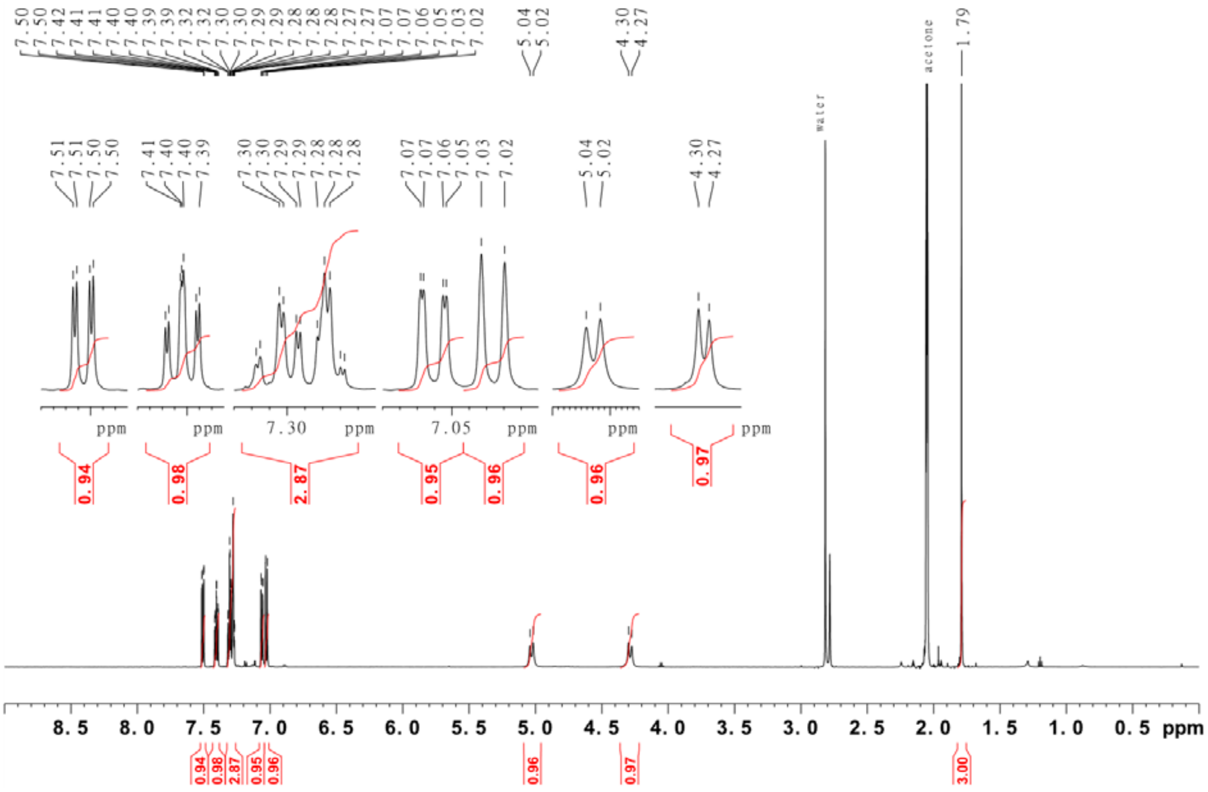

**Figure SII.25:** 600 MHz $^{1}$H-NMR spectrum of (*Z*)-1-(3-iododibenzo[*c*,*g*][1,2,5]triazocin-11(12*H*)-yl)-ethan-1-one (**3**).

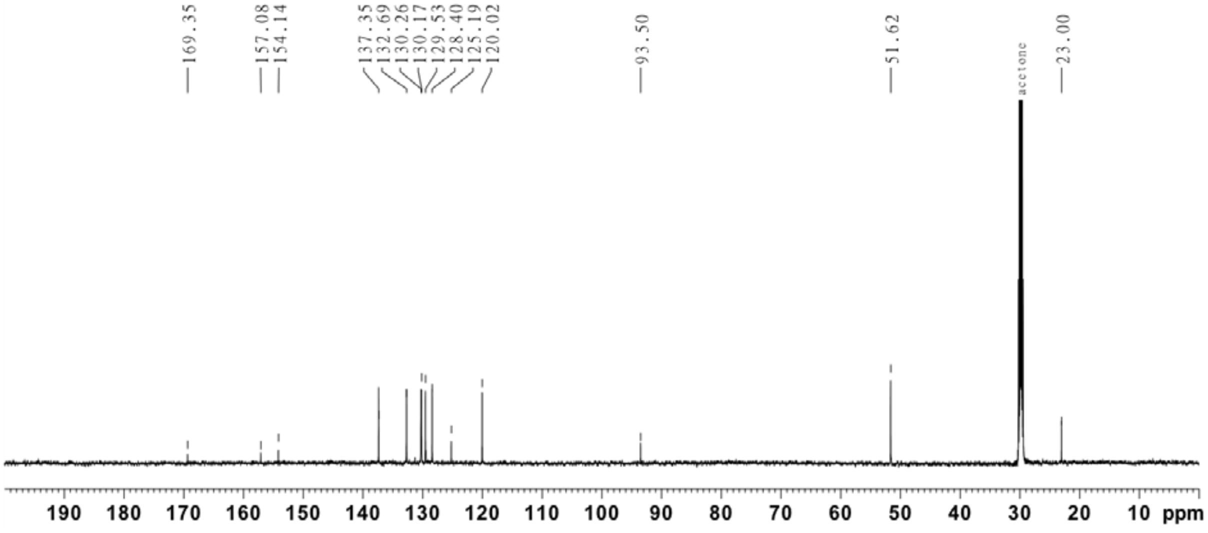

**Figure SII.26:** 150 MHz $^{13}$C{$^{1}$H}-NMR spectrum of (*Z*)-1-(3-iododibenzo[*c*,*g*][1,2,5]triazocin-11(12*H*)-yl)-ethan-1-one (**3**).

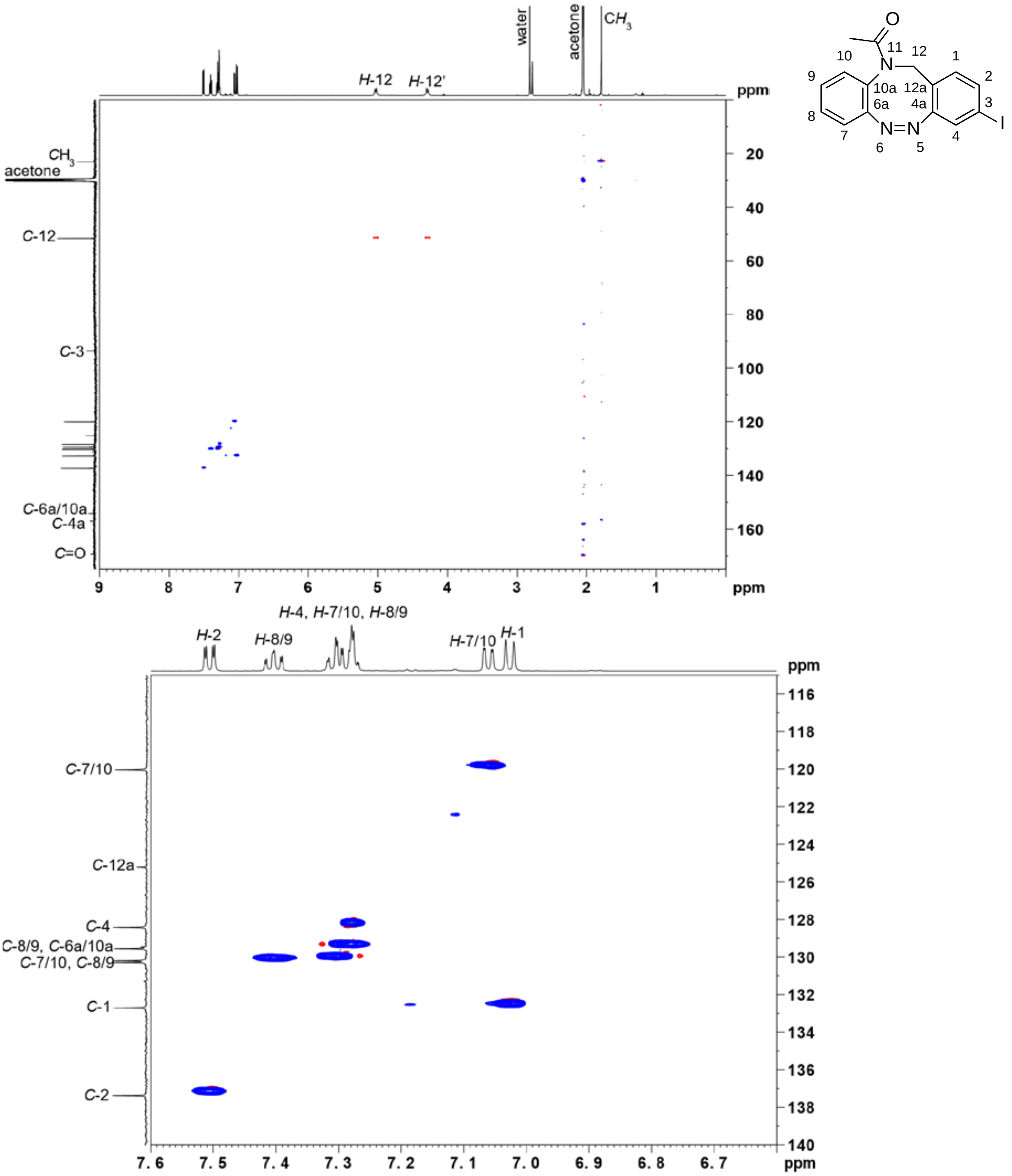


**Figure SII.27:** 600 MHz $^{1}$H-$^{13}$C HSQC spectrum of (*Z*)-1-(3-iododibenzo[*c*,*g*][1,2,5]triazocin-11(12*H*)-yl)-ethan-1-one (**3**).

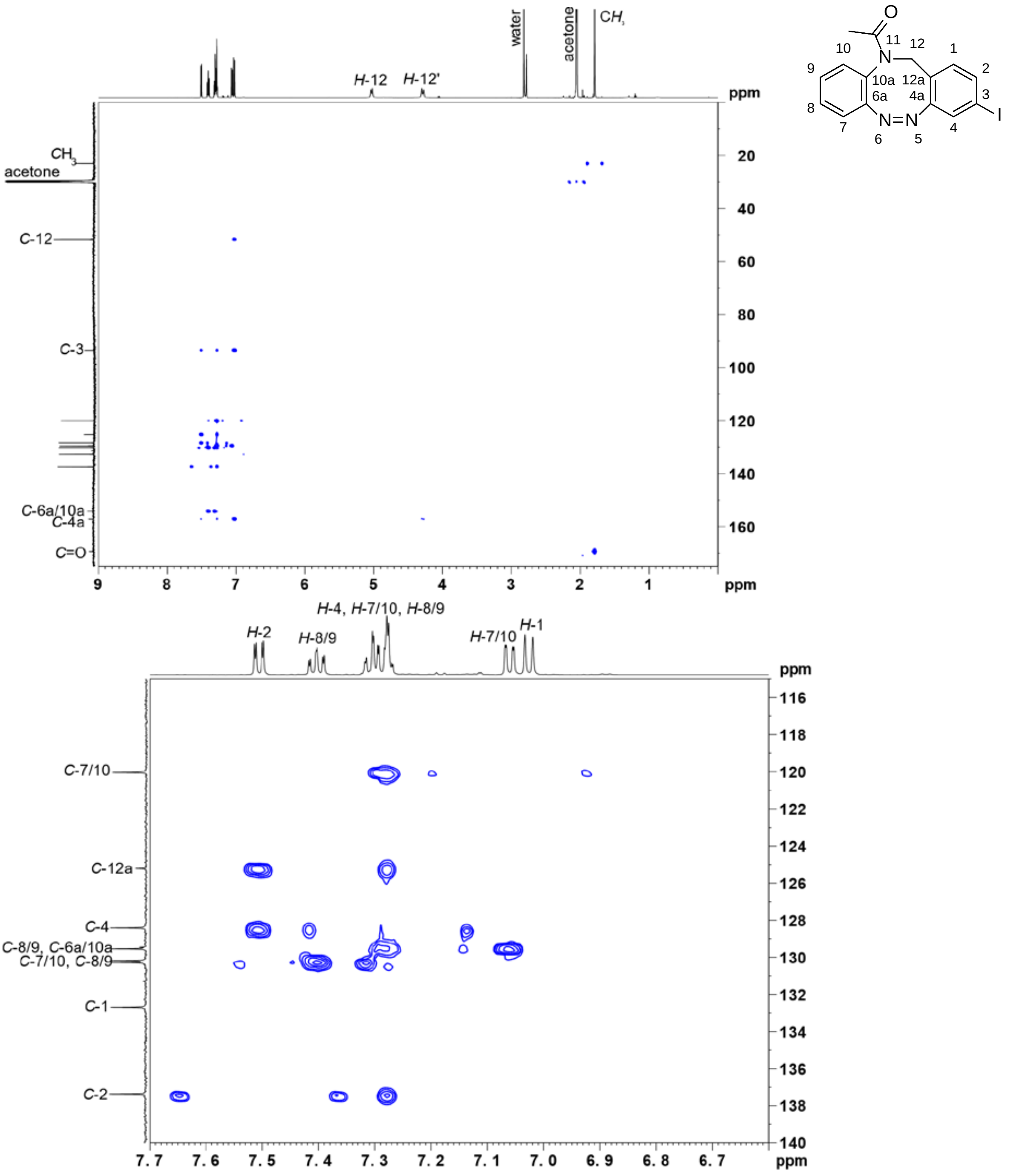


**Figure SII.28:** 600 MHz HMBC spectrum of (*Z*)-1-(3-iododibenzo[*c*,*g*][1,2,5]triazocin-11(12*H*)-yl)-ethan-1-one (**3**).

## II.1.13 Synthesis of *tert*-butyl (5-bromo-2-nitrophenyl)carbamate

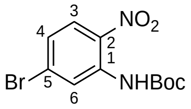


Under a nitrogen atmosphere NaH (444 mg, 18.5 mmol, 60% in mineral oil) was suspended in 10 mL anhydrous DMF and 5-bromo-2-nitroaniline (**6**, 1.00 g, 4.61 mmol) was added at 0 °C. The reaction mixture was stirred at 0 °C for 10 min. and at rt. for 30 min. Di-*tert*-butyldicarbonate (1.00 g, 4.66 mmol) dissolved in 10 mL anhydrous DMF was added dropwise and the reaction mixture was stirred at rt. for 16 h and poured on ice subsequently. The organic layer was separated and the aqueous layer was extracted twice with 50 mL ethyl acetate. The combined organic layers were dried over $MgSO_4$ and the solvent was evaporated. After column chromatography on silica (ethyl acetate/cyclohexane 1:10, $R_f$ = 0.70) the product could be obtained as pale green solid (1.14 g, 3.60 mmol, 78%).

**melting point:** 83 °C

**$^1$H-NMR** (500 MHz, $CDCl_3$, 298 K): $\delta$ = 9.73 (br. s, 1 H, N*H*), 8.85 (d, $^4J$ = 2.1 Hz, 1 H, *H*-6), 8.06 (d, $^3J$ = 9.1 Hz, 1 H, *H*-3), 7.21 (dd, $^3J$ = 9.0 Hz, $^4J$ = 2.2 Hz, 1 H, *H*-4), 1.55 (s, 10 H, C*H*$_3$) ppm.

**$^{13}$C{$^1$H}-NM**R (125 MHz, $CDCl_3$, 198 K): $\delta$ = 151.9 (*C*=O), 137.0 (*C*-1), 134.6 (*C*-2), 131.5 (*C*-4), 127.1 (*C*-6), 125.2 (*C*-5), 123.4 (*C*-3), 82.6 (*C*-($CH_3$)$_3$), 28.3 (*C*H$_3$), ppm.

**IR** (ATR): $\tilde{\nu}$ = 3356 (w), 3115 (w), 2980 (w), 1729 (m), 1605 (m), 1569 (m), 1537 (w), 1489 (m), 1470 (m), 1427 (m), 1392 (w), 1368 (w), 1327 (m), 1298 (m), 1244 (m), 1204 (w), 1144 (s), 1093 (w), 1073 (m), 1049 (w), 1027 (w), 923 (w), 837 (m), 818 (m), 773 (w), 761 (w), 750 (m), 672 (m), 603 (w), 583 (w) cm$^{-1}$.

**MS** (EI, 70 eV): *m/z*(%) = 318.00 $[C_{11}H_{13}{}^{81}BrN_2O_4]^+$, 316.01 $[C_{11}H_{13}{}^{79}BrN_2O_4]^+$, 260.95 $[C_8H_8{}^{79}BrN_2O_3]^+$, 215.95 $[C_6H_3{}^{79}BrN_2O_2]^+$, 185.95 $[C_6H_3{}^{79}BrN_1O_1]^+$, 169.94 $[C_6H_3{}^{79}BrN]^+$, 90.03 $[C_6H_3{}^{79}BrN]^+$.

**HR-MS** (EI, 70 eV, acetone): m/z $[M]^+$ calculated for $C_{11}H_{13}N_2O_4{}^{79}Br^+$: 316.0057; found.: 316.0059 ± 0.44 ppm.

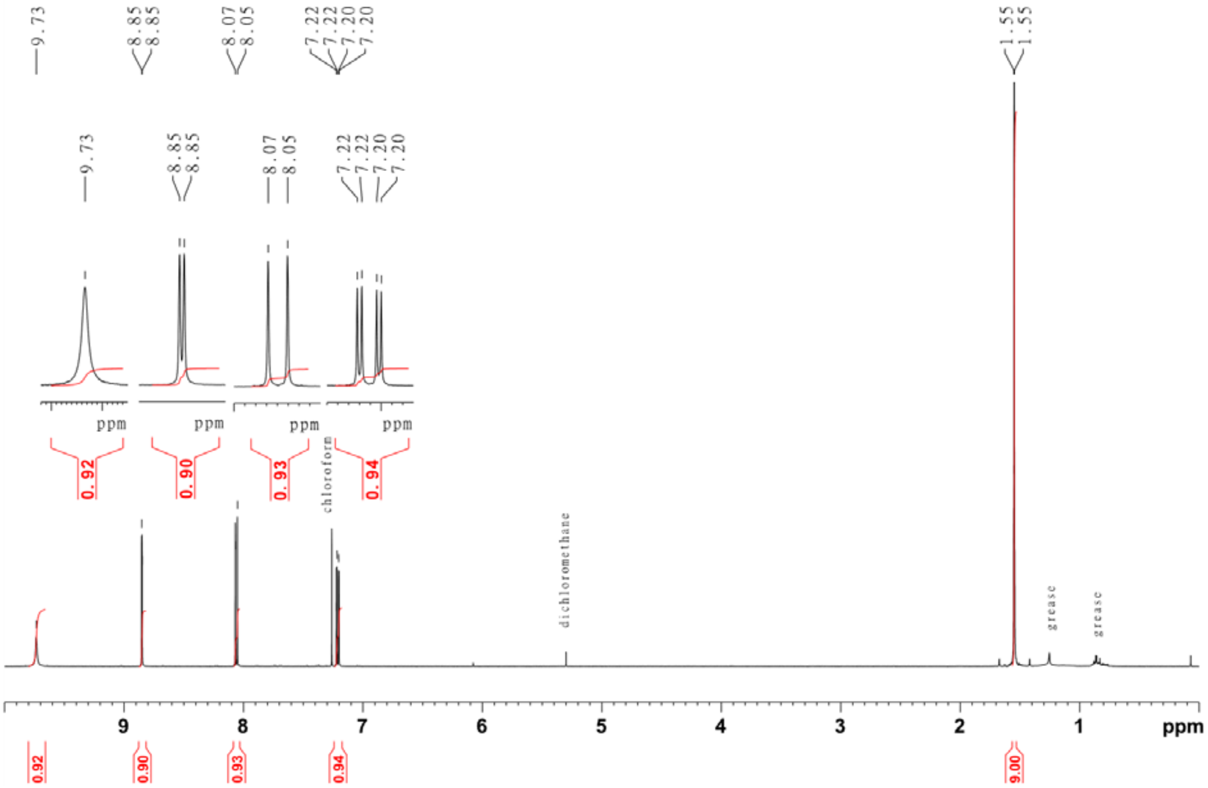


**Figure SII.29:** 500 MHz $^{1}H$-NMR spectrum of *tert*-butyl (5-bromo-2-nitrophenyl)carbamate.

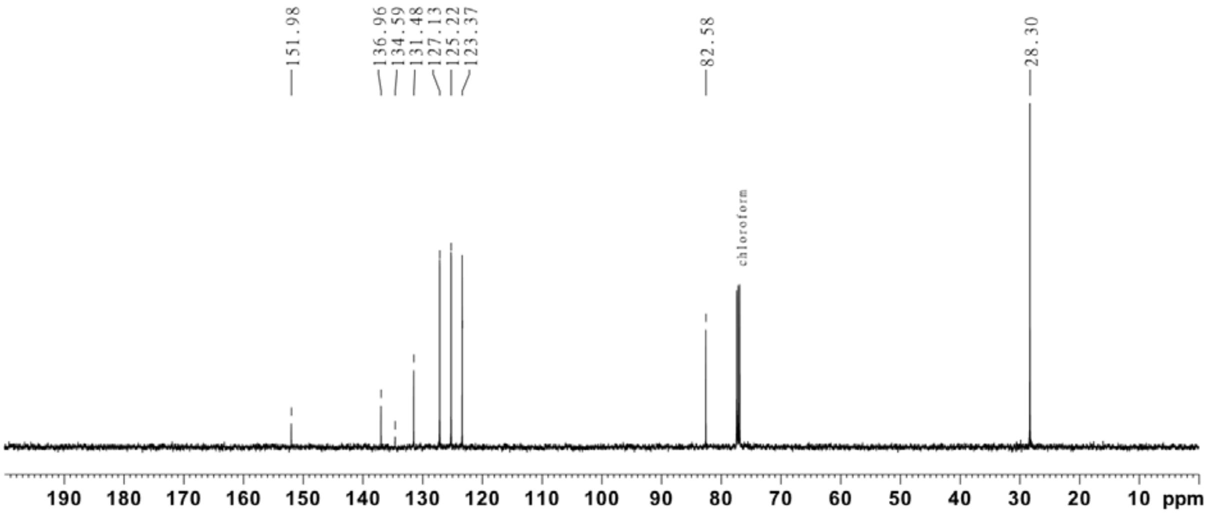


**Figure SII.30:** 125 MHz $^{13}C\{^{1}H\}$-NMR spectrum of *tert*-butyl (5-bromo-2-nitrophenyl)carbamate.

## II.1.14 Synthesis of *tert*-butyl-(2-amino-5-bromophenyl)carbamate (5)

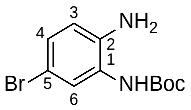

*tert*-butyl (5-bromo-2-nitrophenyl)carbamate (1.80 g, 5.70 mmol), sodium dithionite (3.97 g, 22.7 mmol) and sodium bicarbonate (4.79 g, 57.0 mmol) were suspended in 48 mL 1,4-dioxane/deionized water mixture (5:1), heated to reflux with an oil bath and stirred at given temperature for 2 h. After cooling down 100 mL deionized water were added and the reaction mixture was extracted three times with 50 mL ethyl acetate. After drying the combined organic layers over $MgSO_4$ and evaporation of the solvent the product could be obtained as yellow solid (1.58 g, 5.53 mmol, 97 %)

**melting point:** 141 °C

**$^1$H-NMR** (500 MHz, $CDCl_3$, 298 K): $\delta$ = 7.52 (s, 1 H, *H*-6), 7.08 (dd, $^3J$ = 2.4 Hz, $^4J$ = 8.4 Hz, 1 H, *H*-4), 6.67 (d, $^4J$ = 8.4 Hz, 1 H, *H*-3), 6.32 (s, 1 H, -N*H*), 3.71(s, 2 H, -N*H*2), 1.51 (s, 9 H, -C*H*$_3$) ppm.

**$^{13}$C{$^1$H}-NMR** (125 MHz, $CDCl_3$, 298 K): $\delta$ = 153.5 (*C*=O), 137.8 (*C*-2), 128.6 (*C*-4), 126.8 (*C*-6), 119.3 (*C*-3), 111.8 (*C*-1), 81.2 (*C*-$(CH_3)_3$), 28.4 (*C*H$_3$) ppm.

**IR** (ATR): $\tilde{\nu}$ = 3440 (w), 3359 (m), 3295 (m), 2980 (m), 2929 (w), 2163 (w), 1980 (w), 1673 (s), 1634 (m), 1589 (m), 1515 (s), 1488 (m), 1454 (w), 1416 (s), 1389 (m), 1362 (m), 1322 (m), 1282 (s), 1248 (s), 1224 (w), 1149 (s), 1049 (m), 1024 (m), 882 (m), 857 (m), 817 (w), 807 (m), 777 (m), 760 (w), 742 (m), 627 (m), 601 (m), 556 (s) cm$^{-1}$.

**MS** (EI, 70 eV): m/z (%) = 288.02991 $[C_{11}H_{15}{}^{79}BrN_2O_2]^+$, 286.03176 $[C_{11}H_{15}{}^{79}BrN_2O_2]^+$, 229.97 $[C_7H_6{}^{79}BrN_2O_2]^+$, 185.98 $[C_6H_6{}^{79}BrN^2]^+$.

**HR-MS** (EI, 70 eV, acetone): m/z $[M]^+$ calculated for $C_{11}H_{15}{}^{79}BrN_2O_2{}^+$: 286.0318; found: 286.0317 ± 0.24 ppm.

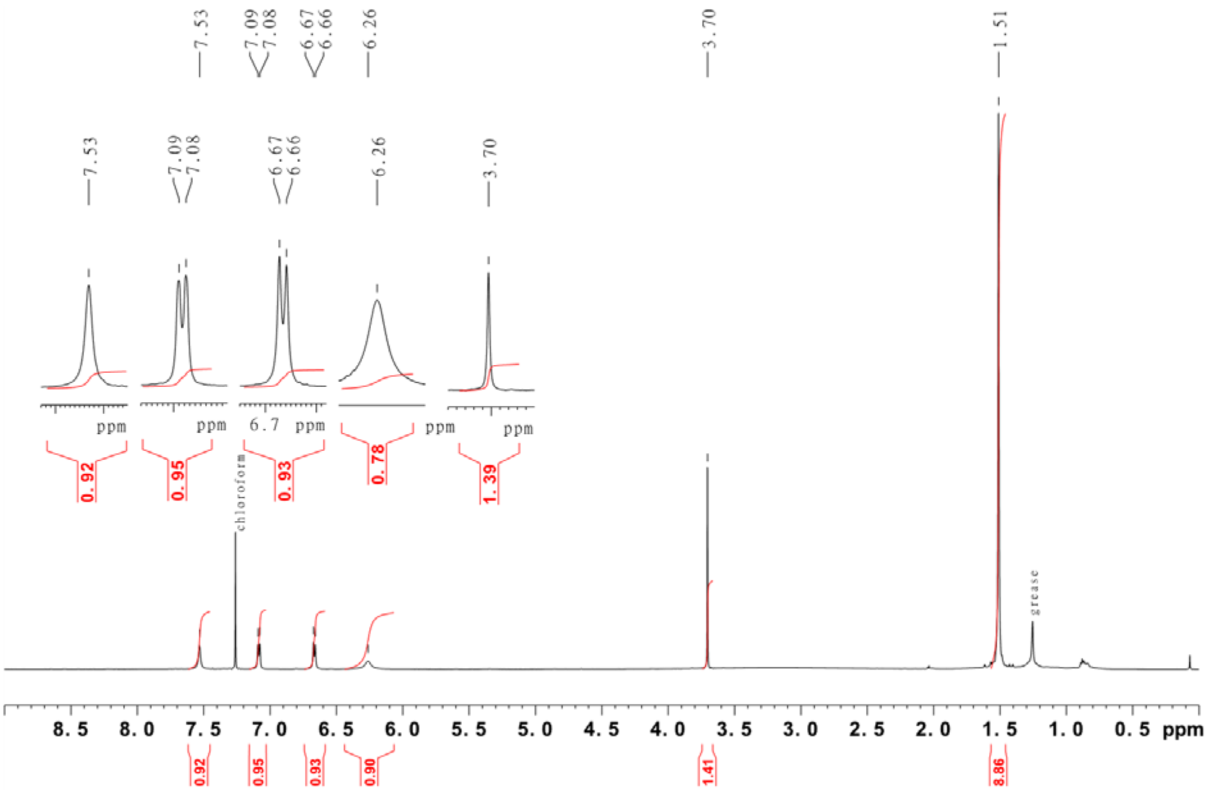


**Figure SII.31:** 500 MHz $^{1}H$-NMR spectrum of *tert*-butyl (2-amino-5-bromophenyl)carbamate (**5**).

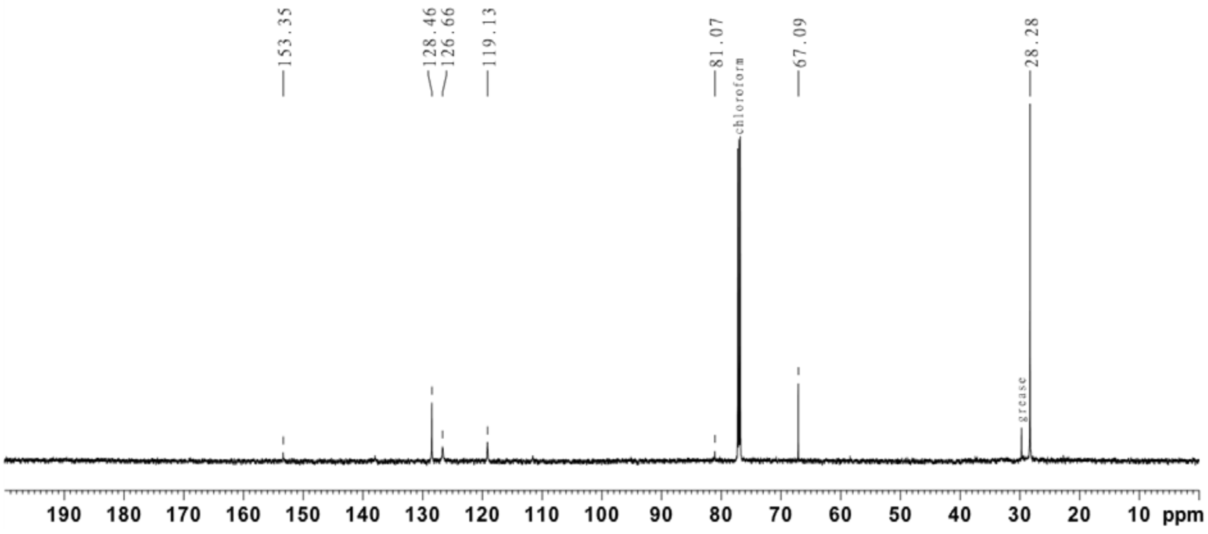


**Figure SII.32:** 125 MHz $^{13}C\{^{1}H\}$-NMR spectrum of *tert*-butyl (2-amino-5-bromophenyl)carbamate (**5**).

## II.1.15 Synthesis of *tert*-butyl (5-bromo-2-((4-bromo-2-nitrobenzyl)amino)phenyl)carbamate

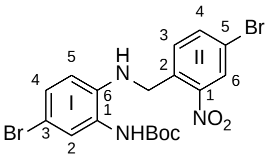


In a manner analogous to [1] triethylamine (3.06 mL, 18.0 mmol) and 1-(bromomethyl)-4-bromo-2-nitrobenzene (5.17 g, 17.5 mmol) were added to solution of *tert*-butyl (2-amino-5-bromophenyl)carbamate (5.02 g, 17.5 mmol) in 100 mL dry THF under a nitrogen atmosphere. The reaction mixture was heated to reflux with an oil bath and stirred for 16 h at given temperature. Afterwards the solvent was evaporated and 100 mL of deionized water and 100 mL DCM were added to the residue. The organic layer was separated and the aqueous layer was extracted twice with 50 mL of DCM. The combined organic layers were dried over $MgSO_4$ and the solvent was evaporated. Recrystallization from cyclohexane/ethyl acetate (1:1) gave the product as yellow solid (7.39 g, 15.7 mmol, 62%).

**melting point:** 186 °C

**$^{1}$H-NMR** (500 MHz, $CDCl_3$, 298 K): $\delta$ = 8.22 (d, $^{4}J$ = 2.0 Hz, 1 H, Ar$^{II}$-*H*-3), 7.68 (dd, $^{4}J$ = 2.1 Hz, $^{3}J$ = 8.3 Hz, 1 H, Ar$^{II}$-*H*-4), 7.49-7.43 (m, 2 H, Ar$^{II}$-*H*-3, Ar$^{I}$-*H*-2), 7.08 (dd, $^{4}J$ = 2.2 Hz, $^{3}J$ = 8.6 Hz, 1 H, Ar$^{I}$-*H*-4), 6.32 (s, 1 H, Ar$^{I}$-*H*-5), 6.20 (br. s, 1 H, -CON*H*), 4.98 (br. S, 1 H, -N*H*), 4.74-4.41 (m, 3 H, -C$H_2$, -N*H*), 1.51 (s, 9 H, -C$H_3$) ppm.

**$^{13}$C{$^{1}$H}-NMR** (125 MHz, $CDCl_3$, 198 K): $\delta$ = 153.9 (*C*=O), 148.8 (Ar$^{II}$-*C*-1), 140.1 (Ar$^{I}$-*C*-2), 136.9 (Ar$^{II}$-*C*-4), 133.9 (Ar$^{II}$-*C*-5), 131.4 (Ar$^{II}$-*C*-3), 129.5 (Ar$^{I}$-*C*-4), 128.30 (Ar$^{II}$-*C*-6), 128.15 (Ar$^{I}$-*C*-6), 126.1 (Ar$^{I}$-*C*-1), 121.5 (Ar$^{II}$-*C*-2), 114.6 (Ar$^{II}$-*C*-3), 110.90 (Ar$^{II}$-*C*-5), 81.5 (-*C*-($CH_3$)$_3$), 45.6 (-*C*$H_2$), 28.4 (-*C*$H_3$) ppm.

**IR** (ATR): $\tilde{\nu}$ = 3421 (m), 2982 (m), 2211 (w), 1685 (s), 1594 (m), 1527 (s), 1497 (m), 1366 (m), 1342 (m), 1281 (m), 1243 (s), 1152 (s), 1054 (w), 875 (s), 857 (w), 831 (w), 807 (m), 790 (w), 751 (m), 627 (w), 551 (m) $cm^{-1}$.

**HR-MS** (ESI, DCM): m/z $[M+H]^+$ calculated for $C_{18}H_{19}{}^{79}Br_2N_3O_4+H^+$: 499.9815; found: 499.9808 ± 151 ppm.

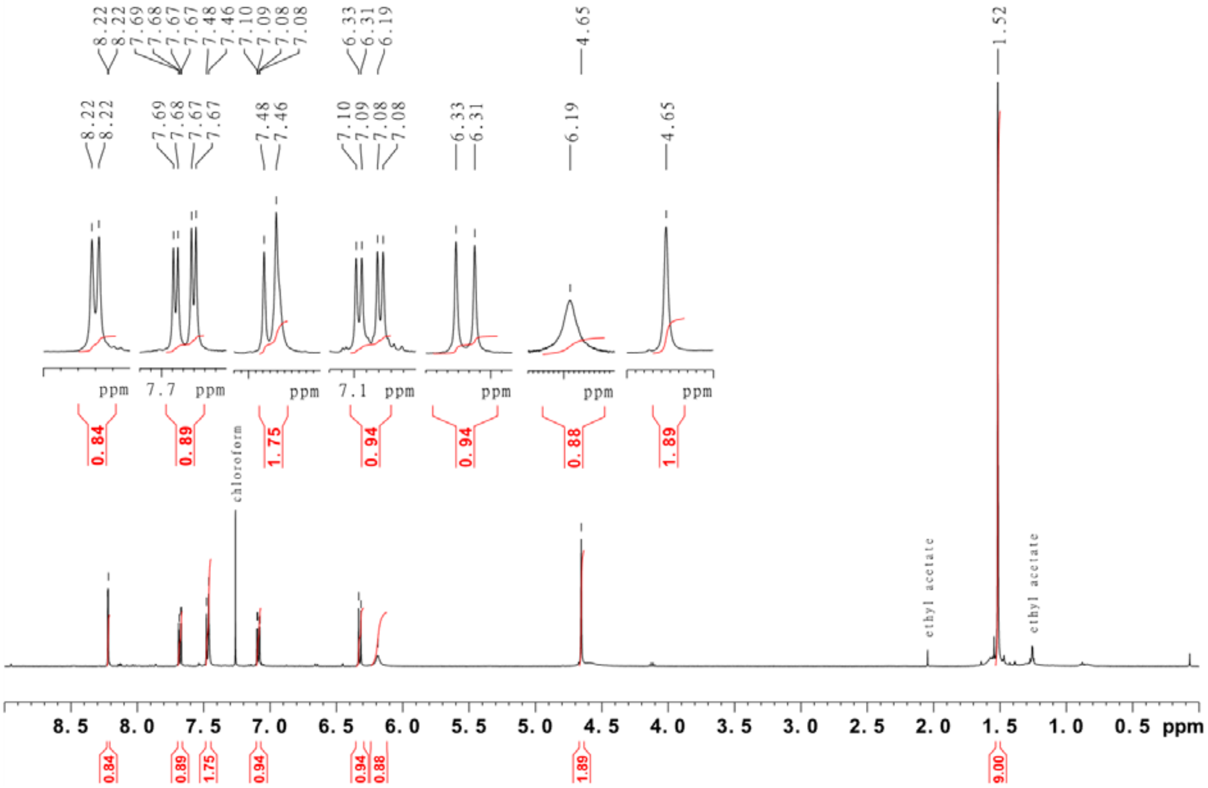

**Figure SII.33:** 500 MHz $^{1}$H-NMR spectrum of *tert*-butyl (5-bromo-2-((4-bromo-2-nitrobenzyl)amino)phenyl)carbamate.

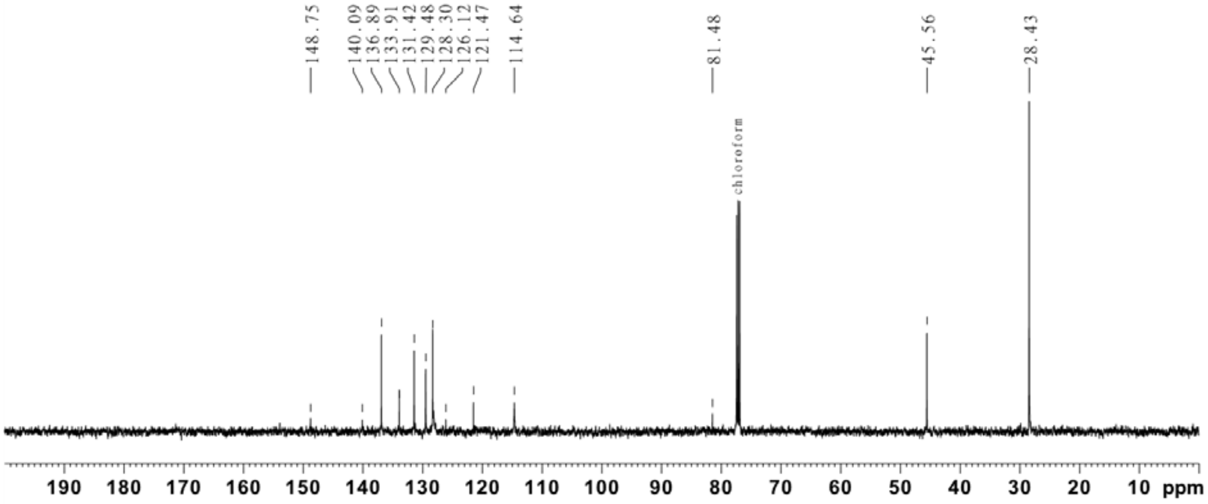

**Figure SII.34:** 125 MHz $^{13}$C{$^{1}$H}-NMR spectrum of *tert*-butyl (5-bromo-2-((4-bromo-2-nitrobenzyl)amino)phenyl)carbamate.

## II.1.16 Synthesis of (9*H*-fluoren-2-yl)methyl (4-bromo-2-((*tert*-butoxycarbonyl)amino)phenyl)(4-bromo-2-nitrobenzyl)carbamate

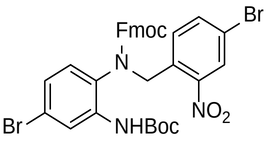


In a manner analogous to [1] DIPEA (820 µL, 5 mmol) and 9-Fluorenylmethoxycarbonyl chlorid (2.50 g, 4.83 mmol) were added to a solution of *tert*-butyl (5-bromo-2-((4-bromo-2-nitrobenzyl)amino)phenyl)carbamate (2.42 g, 4.83 mmol) in 50 mL dry. DMF under a nitrogen atmosphere. The reaction mixture was stirred for 48 h at rt. and the solvent was evaporated subsequently. 100 mL of deionized water and 100 mL of DCM were added to the residue. The organic layer was separated and the aqueous layer was extracted twice with 100 mL DCM. The combined organic layers were dried over $MgSO_4$ and the solvent was evaporated. Column chromatography on silica (ethyl acetate/cyclohexane 1:4, $R_f$ = 0.34) gave a colorless solid (302 mg, 418 µmol, 13%).

**melting point:** 156 °C

**$^1$H-NMR** (500 MHz, $CDCl_3$, 298 K): $\delta$ = 8.16 (br. s, 1 H, -CON*H*), 8.06 (s, 1 H, Ar-*H*), 7.66 (d, $^3J$ = 7.4 Hz, 2 H, Ar-*H*), 7.58 (br. s, 1 H, Ar-*H*), 7.38-7.33 (m, 2 H, Ar-*H*), 7.25-6.98 (s, 6 H, Ar-*H*), 6.61 (br. s, 1 H, Ar-*H*), 6.32 (br. s, 1 H, Ar-*H*), 5.09 (br. s, 1 H, -$CH_2$), 4.78 (br. s, 1 H, -$CH_2$), 4.52 (br. s, 2 H, Fmoc-$CH_2$), 4.05 (s, 1 H, Fmoc-C*H*), 1.46 (s, 9 H, -$CH_3$) ppm.

**$^{13}$C{$^1$H}-NMR** (125 MHz, $CDCl_3$, 298 K): $\delta$ = 155.6, 143.3, 141.4, 136.5, 129.0, 128.08, 127.86, 127.1, 126.5, 124.7, 123.3, 120.1, 81.6, 68.2, 47.0, 28.4 ppm.

**IR** (ATR): $\tilde{\nu}$ = 2929 (w), 2166 (w), 1713 (s), 1583 (m), 1531 (s), 1512 (s), 1450 (m), 1392 (w), 1347 (w), 1276 (w), 1233 (m), 1151 (s), 1022 (w), 877 (m), 758 (s), 749 (s), 739 (s), 621 (w), 545 (w) $cm^{-1}$.

**HR-MS** (ESI, $CHCl_3$/MeOH): *m/z* $[M+H]^+$ calculated for $C_{33}H_{29}O_6N_3Br_2+H^+$: 722.0496; found: 722.0490 ± 0.82 ppm.

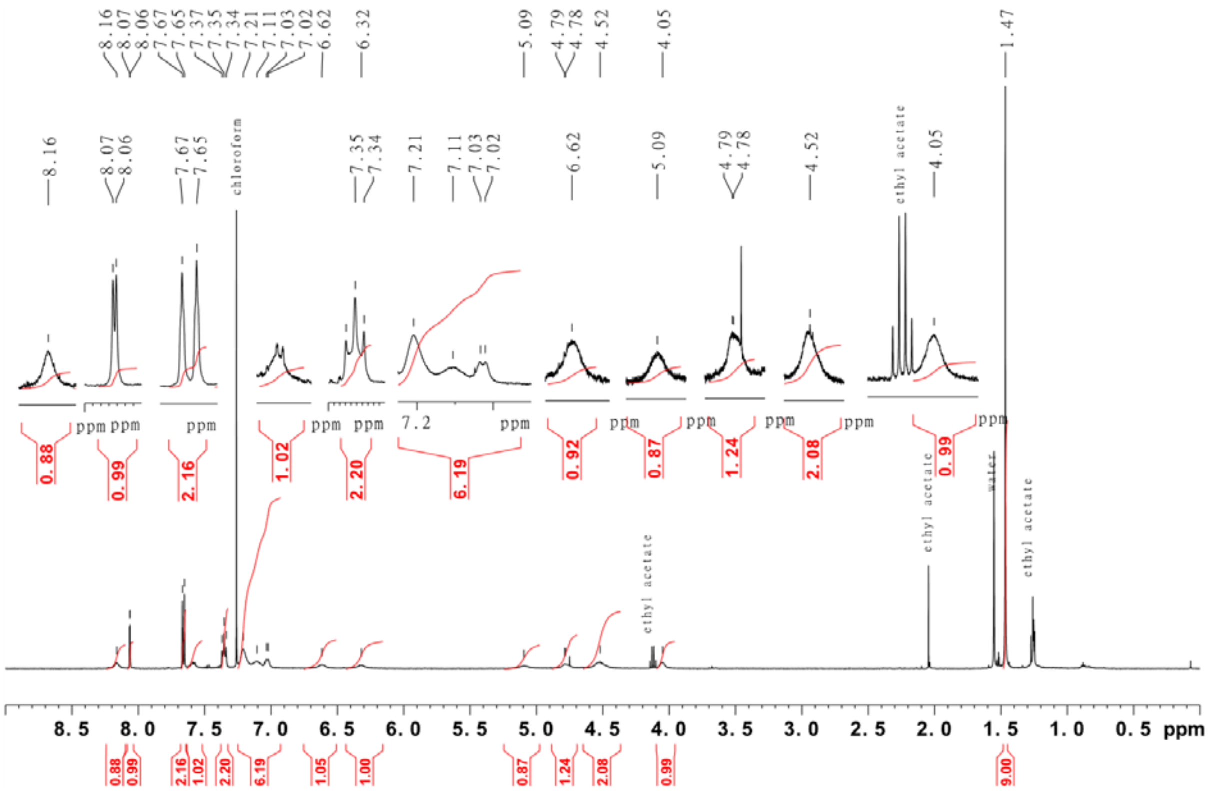

**Figure SII.35:** 500 MHz $^{1}$H-NMR spectrum of (9*H*-fluoren-2-yl)methyl (4-bromo-2-((*tert*-butoxycarbonyl)amino)phenyl)(4-bromo-2-nitrobenzyl)carbamate.

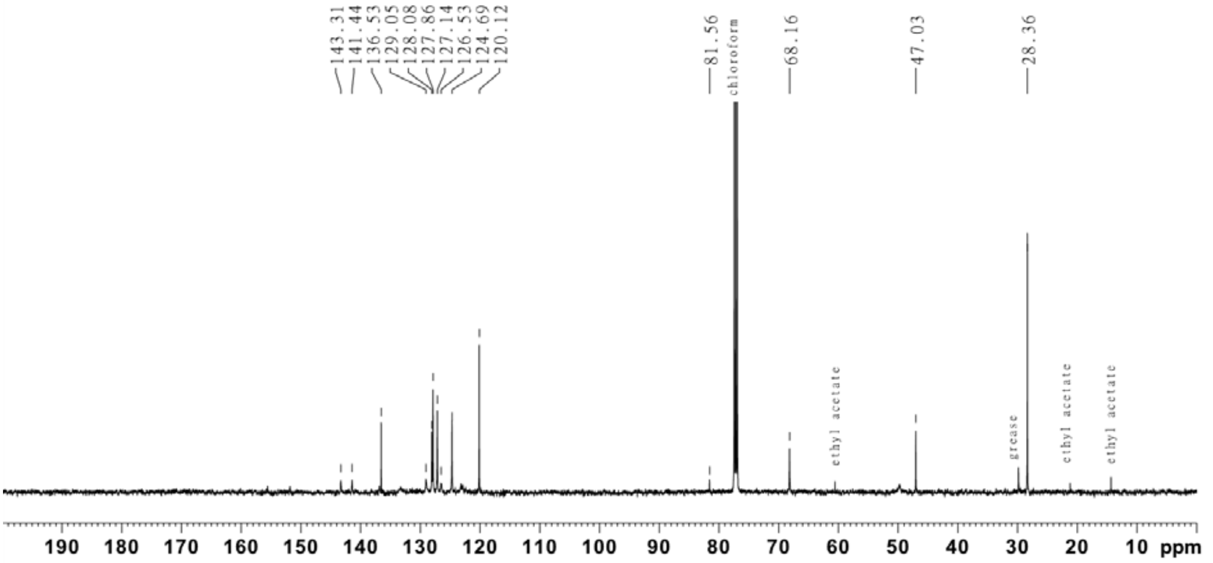

**Figure SII.36:** 125 MHz $^{13}$C{$^{1}$H}-NMR spectrum of (9*H*-fluoren-2-yl)methyl (4-bromo-2-((*tert*-butoxycarbonyl)amino)phenyl)(4-bromo-2-nitrobenzyl)carbamate.

## II.1.17 Synthesis of (9*H*-fluoren-9-yl)methyl (2-amino-4-bromophenyl)(4-bromo-2-nitrobenzyl)carbamate

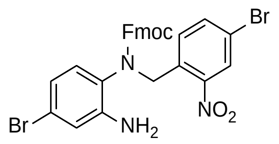


In a manner analogous to [1] (9*H*-fluoren-2-yl)methyl (4-bromo-2-((*tert*-butoxycarbonyl)amino)phenyl)(4-bromo-2-nitrobenzyl)carbamate (599 mg, 828 µmol) was dissolved in 25 mL DCM and 10 mL TFA was added. The reaction mixture was stirred at rt. for 16 h and neutralized with saturated aqueous $NaHCO_3$ subsequently. The organic layer was separated and the aqueous layer was extracted twice with 100 mL. The combined organic layers were dried over $MgSO_4$ and the solvent was evaporated to obtain a pale yellow solid (518 mg, 827 µmol, quant.) which was used without further purification.

**melting point:** 85 °C

**$^1$H-NMR** (600 MHz, $CDCl_3$, 298 K): $\delta$ = 8.06 (d, $^4J$ = 1.9 Hz, 1 H, Ar-*H*), 7.73-7.61 (m, 3 H, Ar-*H*), 7.39-7.33 (m, 3 H, Ar-*H*), 7.25-7.11 (m, 4 H, Ar-*H*), 6.82 (br. s, 1 H, Ar-*H*), 6.70 (br. s, 1 H, Ar-*H*), 6.47 (br. s, 1 H, Ar-*H*), 5.11 (br. s, 1 H, -C$H_2$), 4.88 (br. s, 1 H, -C*H*2), 4.49 (d, $^2J$ = 19.9 Hz 2 H, Fmoc-C$H_2$), 4.07 (br. s, 1 H, Fmoc-C*H*), 3.50 (br. s, 2 H, -N$H_2$) ppm.

**$^{13}$C{$^1$H}-NMR** (150 MHz, $CDCl_3$, 298 K): $\delta$ = 143.2, 140.9, 135.9, 132.3, 129.8, 127.41, 127.25, 126.6, 124.4, 122.3, 119.5, 67.7, 48.3, 46.7 ppm.

**IR** (ATR): $\tilde{\nu}$ = 3388 (m), 2925 (m), 2850 (w), 1704 (s), 1621 (m), 1523 (s), 1496 (s), 1450 (m), 1413 (s), 1394 (w), 1351 (m), 1297 (s), 1278 (w), 1229 (m), 1198 (w), 1182 (w), 1161 (m), 1139 (m), 1101 (w), 1066 (w), 1025 (m), 992 (m), 889 (w), 877 (m), 857 (w), 792 (m), 763 (s), 738 (s), 698 (w), 620 (w), 589 (w), 538 (w), 503 (w) $cm^{-1}$.

**HR-MS** (ESI, $CHCl_3$/MeOH): *m/z* $[M+H]^+$ calculated for $C_{28}H_{21}O_4N_3Br_2+H^+$: 621.9972; found: 621.9975 ± 0.49 ppm.

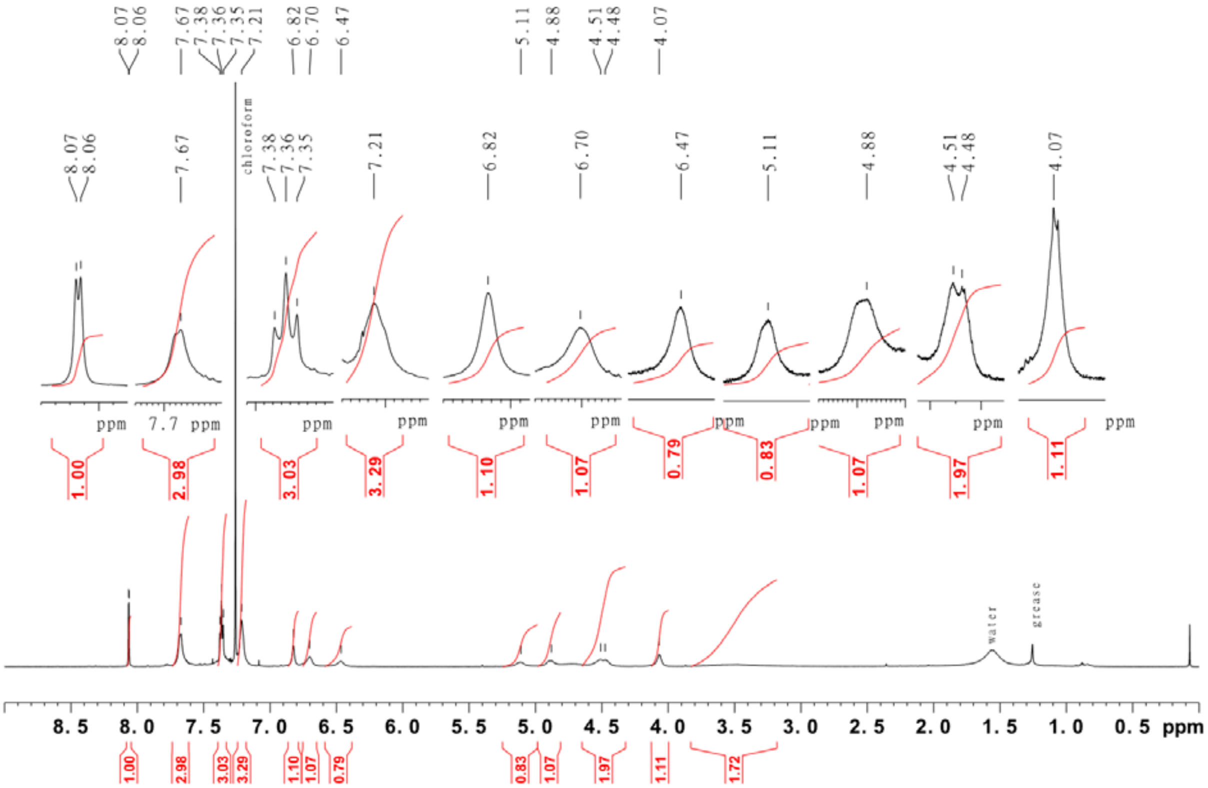


**Figure SII.37:** 600 MHz $^1$H-NMR spectrum of (9*H*-fluoren-9-yl)methyl (2-amino-4-bromophenyl)(4-bromo-2-nitrobenzyl)carbamate.

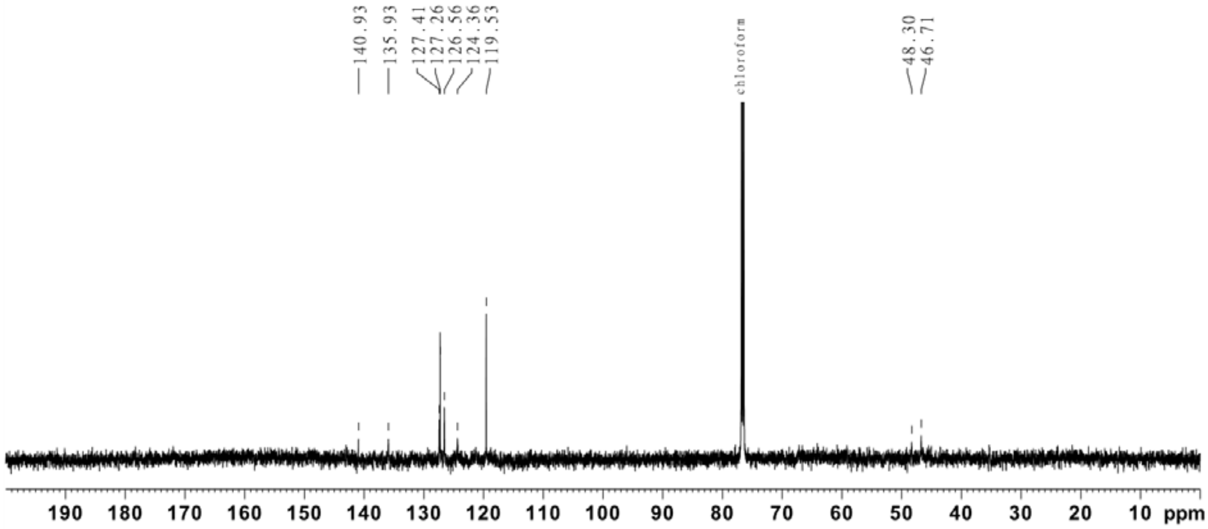


**Figure SII.38:** 150 MHz $^{13}$C{$^1$H}-NMR spectrum of (9*H*-fluoren-9-yl)methyl (2-amino-4-bromophenyl)(4-bromo-2-nitrobenzyl)carbamate.

### II.1.18 Synthesis of (9*H*-fluoren-9-yl)methyl (*Z*)-3,8-dibromodibenzo[*c,g*][1,2,5]triazocin-11(12*H*)carboxylate

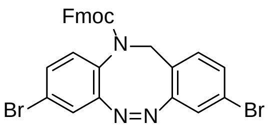


In a manner analogous to [1] (9*H*-fluoren-9-yl)methyl (2-amino-4-bromophenyl)(4-bromo-2-nitrobenzyl)carbamate (500 mg, 802 µmol) was suspended in 50 mL EtOH and $SnCl_2 \cdot 2\ H_2O$ (1.09 g, 4.81 mmol) was added. The reaction mixture was heated to 75 °C with an oil bath, zinc-powder (420 mg, 6.42 mmol) was added and it was stirred at given temperature. After cooling to rt the reaction mixture was stirred at for 16 h and neutralized with saturated aqueous $NaHCO_3$ solution subsequently. The solvent was evaporated and 100 mL water and 100 mL DCM were added to the residue. The organic layer was separated and the aqueous layer was extracted twice with 100 mL DCM. The combined organic layers were dried over $MgSO_4$ and the solvent was evaporated. The residue was dissolved in 25 mL of acetic acid and *m*CPBA (277 mg, 1.60 mmol) dissolved in 25 mL acetic acid was added dropwise to the reaction mixture. The reaction mixture was stirred at rt. for 16 h and the solvent was evaporated in the following. 50 mL DCM and 50 mL half-concentrated aqueous $NaHCO_3$ solution were added to the residue. The organic layer was separated and the aqueous layer was extracted twice with 50 mL DCM. The combined organic layers were dried over $MgSO_4$ and the solvent was evaporated. After column chromatography on silica (ethyl acetate/cyclohexane = 1:4, $R_f$ = 0.58) the product could be obtained as yellow solid (63.6 mg, 108 µmol, 14%).

**melting point:** 91 °C

**$^1$H-NMR** (600 MHz, $CDCl_3$, 298 K): $\delta$ = 7.81-6.58 (m, 14 H, Ar-*H*), 4.92-4.03 (m, 5 H, -C$H_2$, -C$H_2$’, Fmoc-C$H_2$, Fmoc-C*H*) ppm.

**$^{13}$C{$^1$H}-NMR** (150 MHz, $CDCl_3$, 298 K): $\delta$ = 152.8, 143.4, 141.3, 131.23, 131.19, 130.94, 130.71, 127.7, 127.0, 124.8, 122.21, 122.00, 119.9, 67.7, 52.0, 46.9 ppm.

**IR** (ATR): $\tilde{\nu}$ = 2922 (s), 2853 (m), 2360 (w), 2347 (w), 2228 (w), 2169 (w), 2019 (w), 1980 (w), 1961 (w), 1705 (s), 1590 (w), 1464 (w), 1450 (m), 1390 (m), 1317 (m), 1296 (w), 1228 (m), 1176 (m), 1071 (m), 1026 (m), 870 (m), 818 (m), 757 (m), 737 (s), 655 (w), 620 (m), 590 (w), 547 (m), 521 (w), 458 (m) $cm^{-1}$.

**HR-MS** (ESI, DCM): *m/z* $[M+H]^+$ calculated for $C_{28}H_{19}O_2N_3Br_2+H^+$: 587.99169; found: 587.99067 ± 1.74 ppm.

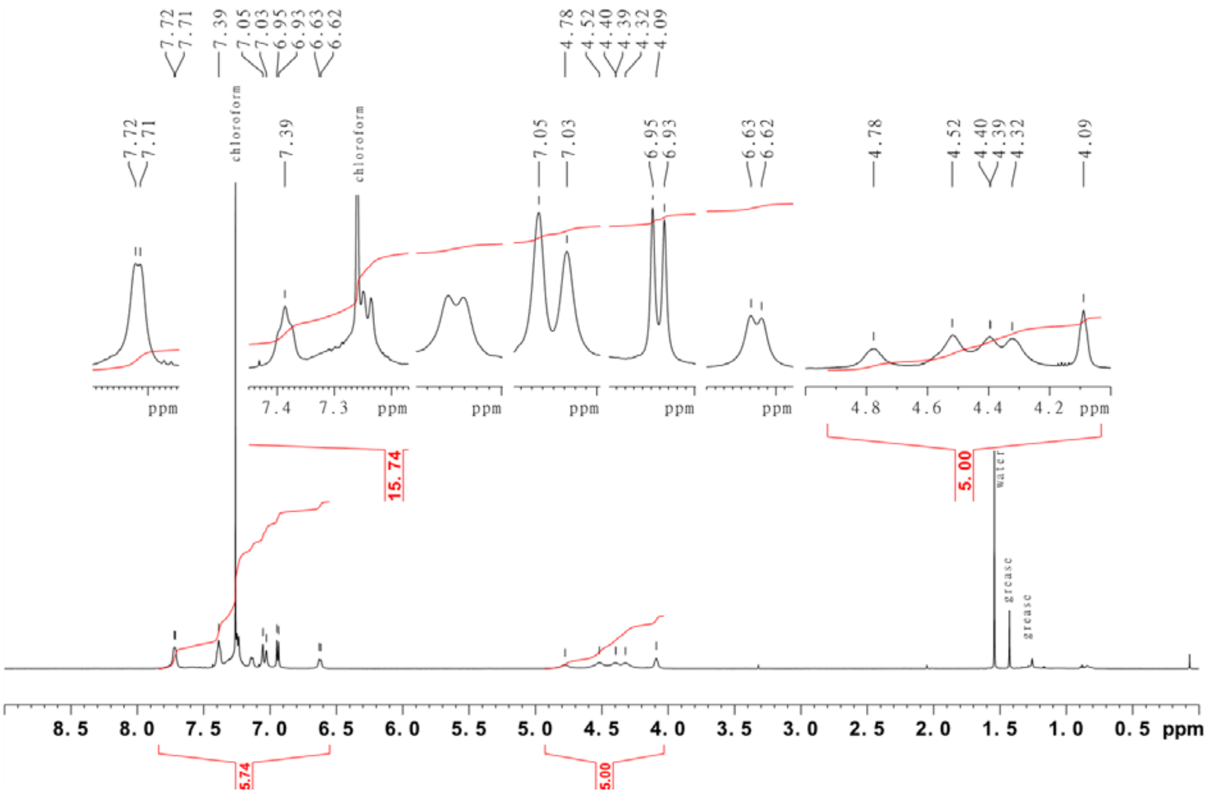

**Figure SII.39:** 600 MHz $^{1}H$-NMR spectrum of (9*H*-fluoren-9-yl)methyl (*Z*)-3,8-dibromodibenzo[*c*,*g*][1,2,5]triazocin-11(12*H*)carboxylate.

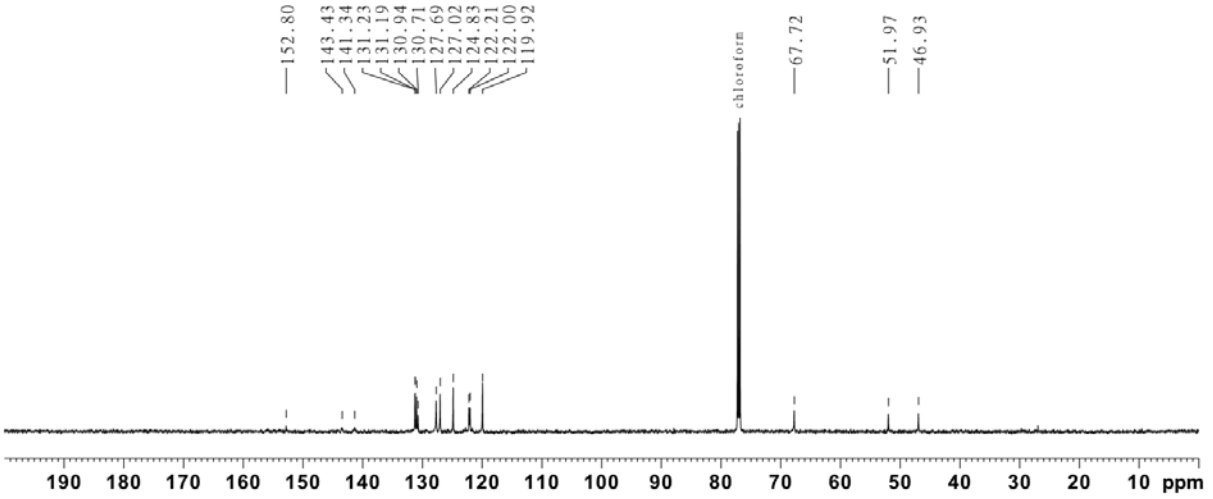

**Figure SII.40:** 150 MHz $^{13}C\{^{1}H\}$-NMR spectrum of (9*H*-fluoren-9-yl)methyl (*Z*)-3,8-dibromodibenzo[*c*,*g*][1,2,5]triazocin-11(12*H*)carboxylate.

## II.1.19 Synthesis of (*Z*)-3,8-dibromo-11,12-dihydrodibenzo[*c,g*][1,2,5]triazocine

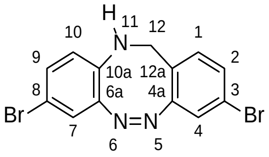


In a manner analogous to [1] (9*H*-fluoren-9-yl)methyl (*Z*)-3,8-dibromodibenzo[*c,g*][1,2,5]triazocin-11(12*H*)carboxylate (30.0 g, 50.9 µmol) was dissolved in 50 mL of DCM and 50 mL $NEt_3$ was added. The reaction mixture was stirred at rt. for 16 h. the solvent was evaporated and column chromatography on silica (ethyl acetate/cyclohexane 1:3, $R_f$ = 0.32) gave a red solid (12.0 mg, 32.7 µmol, 64%).

**melting point:** 191 °C

**$^1$H-NMR** (600 MHz, $CDCl_3$, 298 K): $\delta$ = 7.38 (dd, $^3J$ = 8.1 Hz, $^4J$ = 2.0 Hz, 1 H, *H*-2), 7.33 (d, $^4J$ = 1.9 Hz, 1 H, *H*-4), 7.10 (d, $^3J$ = 8.1 Hz, 1 H, *H*-1), 7.00 (dd, $^3J$ = 8.6 Hz, $^4J$ = 2.3 Hz, 1 H, *H*-9), 6.96 (d, $^4J$ = 2.2 Hz, 1 H, *H*-10), 6.31 (d, $^3J$ = 8.6 Hz, 1 H, *H*-7), 4.08-3.81 (m, 3 H, -N*H, H*-12) ppm.

**$^{13}$C{$^1$H}-NMR** (150 MHz, $CDCl_3$, 298 K): $\delta$ = 158.6 (*C*-4a), 144.4 (*C*-6a), 134.3 (*C*-10-a), 131.8 (*C*-2), 131.4 (*C*-9), 130.8 (*C*-1), 125.7 (*C*-10), 122.8 (*C*-4), 122.3 (*C*-3), 121.6 (*C*-12a), 120.5 (*C*-7), 110.3 (*C*-8), 47.1 (*C*$H_2$) ppm.

**IR** (ATR): $\tilde{\nu}$ = 3307 (m), 2920 (m), 2360 (w), 2177 (w), 2020 (w), 1735 (w), 1588 (m), 1563 (w), 1520 (w), 1501 (w), 1479 (s), 1451 (w), 1378 (m), 1316 (m), 1269 (m), 1244 (m), 1192 (w), 1160 (m), 1120 (m), 1091 (m), 1056 (m), 967 (w), 904 (w), 858 (m), 831 (m), 806 (m), 775 (m), 720 (w), 696 (w), 518 (s) $cm^{-1}$.

**HR-MS** (ESI, EtOAc): *m/z* $[M+H]^+$ calculated for $C_{13}H_9N_3{}^{79}Br_2+H^+$: 365.9236; found: 365.9233 ± 0.89 ppm.

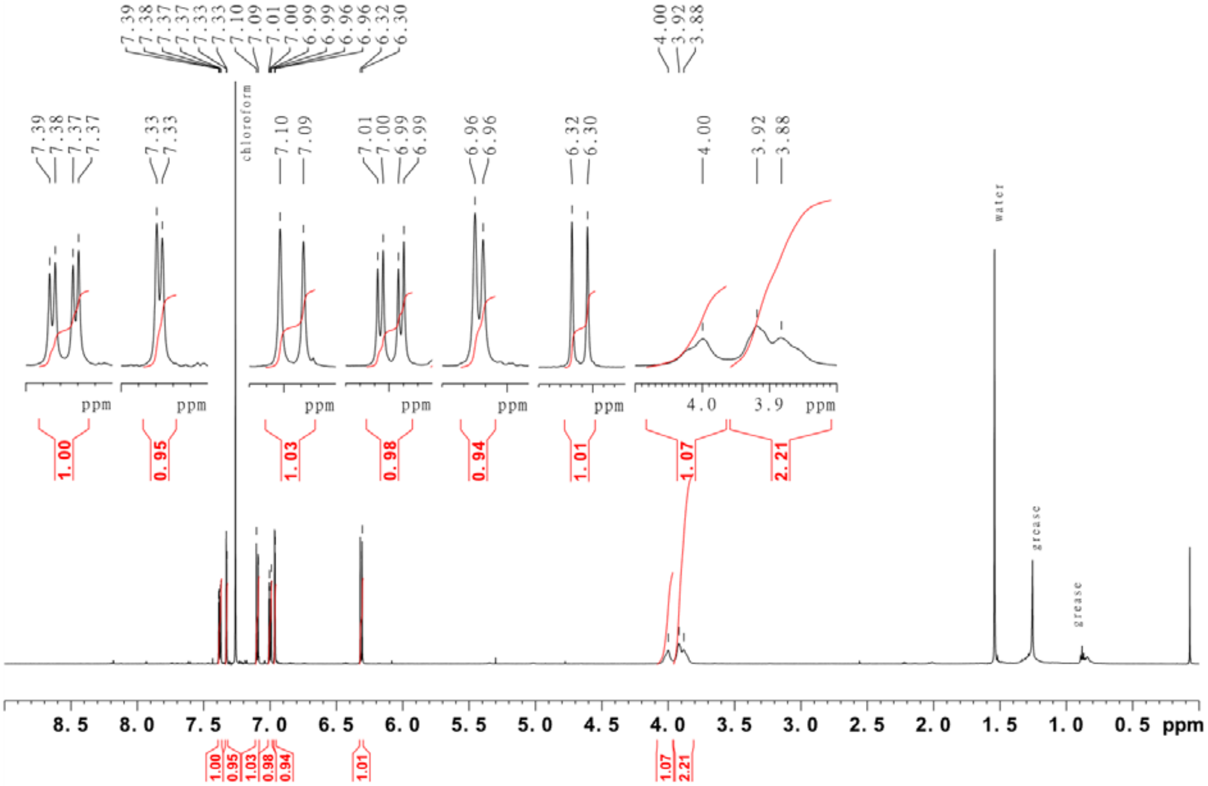

**Figure SII.41:** 600 MHz $^{1}$H-NMR spectrum of (*Z*)-3,8-dibromo-11,12-dihydrodibenzo[*c,g*][1,2,5]triazocine.

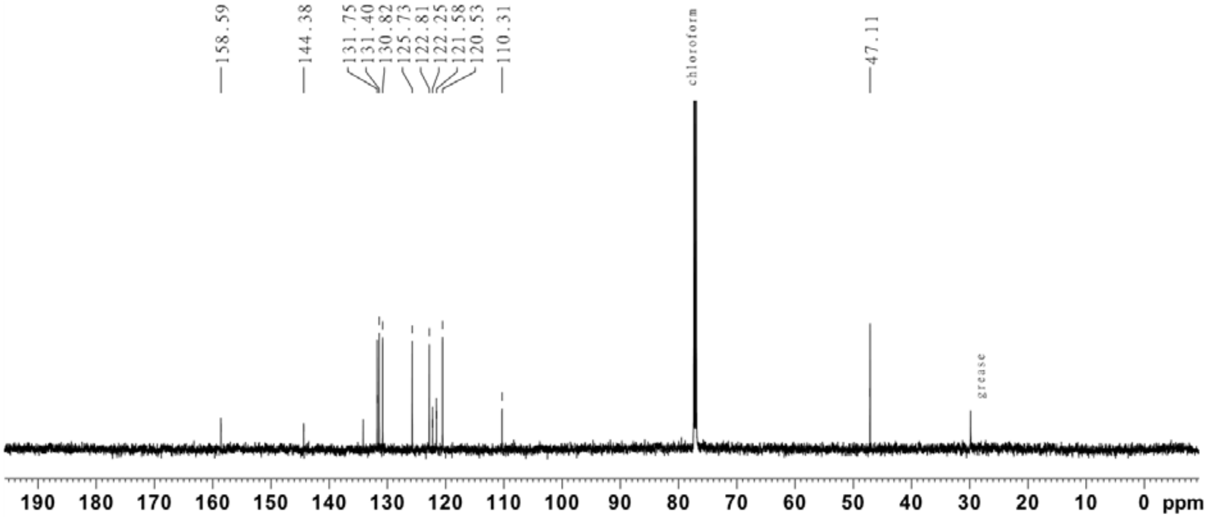

**Figure SII.42:** 150 MHz $^{13}$C{$^{1}$H}-NMR spectrum of (*Z*)-3,8-dibromo-11,12-dihydrodibenzo[*c,g*][1,2,5]triazocine.

## II.1.20 Synthesis of (Z)-(3,8-dibromodibenzo[*c,g*][1,2,5]triazocin-11(12H)-yl)ethan-1-one (4)

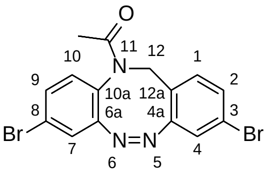

In a manner analogous to [1] (*Z*)-3,8-dibromo-11,12-dihydrodibenzo[*c,g*][1,2,5]triazocine (12.0 mg, 32.7 µmol) were dissolved in 1 mL anhydrous DMF under a nitrogen atmosphere. $NEt_3$ (36.4 µL, 261 µmol) and AcOH (19.0 µL, 32.7 µmol) were added. The reaction mixture was cooled to 0 °C and T3P (167 µL, 327 µmol, 50% in ethyl acetate) was added dropwise. The reaction mixture was stirred at rt. for 16 h. 10 mL DCM and 10 mL deionized water were added and the organic layer was separated. The aqueous layer was extracted twice with 10 mL DCM and the combined organic layers were dried over $MgSO_4$. The solvent was removed and column chromatography on silica (ethyl acetate/cyclohexane 1:1, $R_f$ = 0.37) gave a yellow solid (11.5 mg, 28.1 µmol, 86%).

**melting point:** 222 °C

**$^1$H-NMR** (500 MHz, $CDCl_3$, 298 K): $\delta$ = 7.32 (dd, $^3J$ = 8.4 Hz, $^4J$ = 2.2 Hz, 1 H, *H*-9), 7.25 (dd, $^3J$ = 8.2 Hz, $^4J$ = 2.0 Hz, 1 H, *H*-2), 7.12 (d, $^4J$ = 2.1 Hz, 1 H, *H*-7), 7.07 (d, $^4J$ = 1.8 Hz, 1 H, *H*-4), 6.95 (d, $^3J$ = 8.2 Hz, 1 H, *H*-1), 6.92 (d, $^3J$ = 8.4 Hz, 1 H, *H*-10), 5.01 (d, $^2J$ = 15.2 Hz, 1 H, *H*-12), 4.28 (d, $^2J$ = 14.5 Hz, 1 H, *H*-12'), 1.91 (s, 3 H, -C$H_3$) ppm.

**$^{13}$C{$^1$H}-NMR** (125 MHz, $CDCl_3$, 198 K): $\delta$ = 169.8 (*C*=O), 155.8 (*C*-4a), 153.5 (*C*-6a), 131.9 (*C*-9), 131.44 (*C*-1), 131.17 (*C*-2), 130.3 (*C*-10), 127.6 (*C*-10a), 122.93 (*C*-8), 122.68 (*C*-7), 122.59 (*C*-12a), 122.44 (*C*-4), 51.4 (*C*-12), 23.2 (*C*$H_3$) ppm.

**IR** (ATR): $\tilde{\nu}$ = 2920 (m), 2851 (w), 1654 (s), 1589 (m), 1557 (w), 1523 (w), 1469 (m), 1372 (s), 1332 (m), 1291 (m), 1268 (w), 1241 (w), 1159 (m), 1112 (m), 1075 (m), 1034 (w), 968 (m), 940 (w), 925 (w), 882 (m), 858 (m), 844 (s), 807 (s), 760 (m), 731 (m), 689 (w), 657 (m), 601 (m), 572 (s), 465 (s) cm$^{-1}$.

**HR-MS** (ESI, DCM): *m/z* $[M+H]^+$ calculated for $C_{15}H_{11}O_1N_3Br_2+H^+$: 407.9342; found: 407.9335 ± 1.62 ppm.

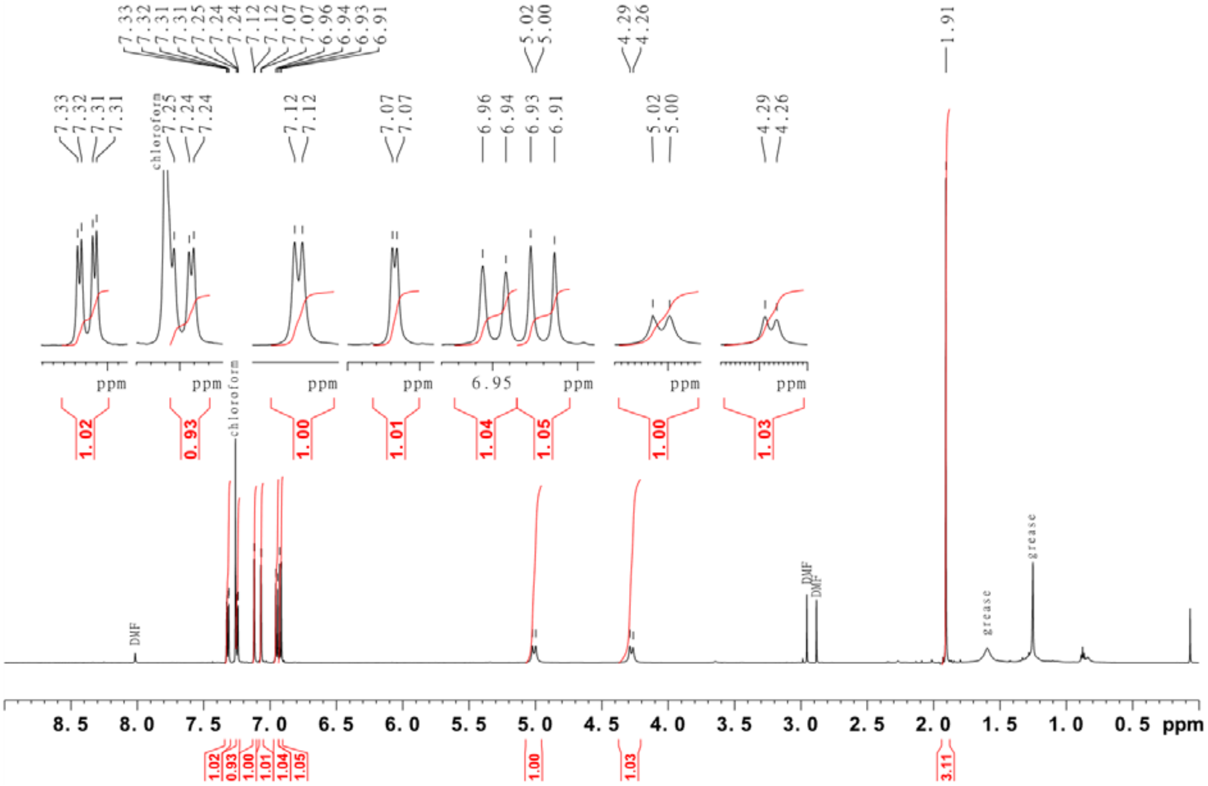


**Figure SII.43:** 500 MHz $^{1}H$-NMR spectrum of (Z)-(3,8-dibromodibenzo[*c*,*g*][1,2,5]triazocin-11(12H)-yl)ethan-1-one (**4**).

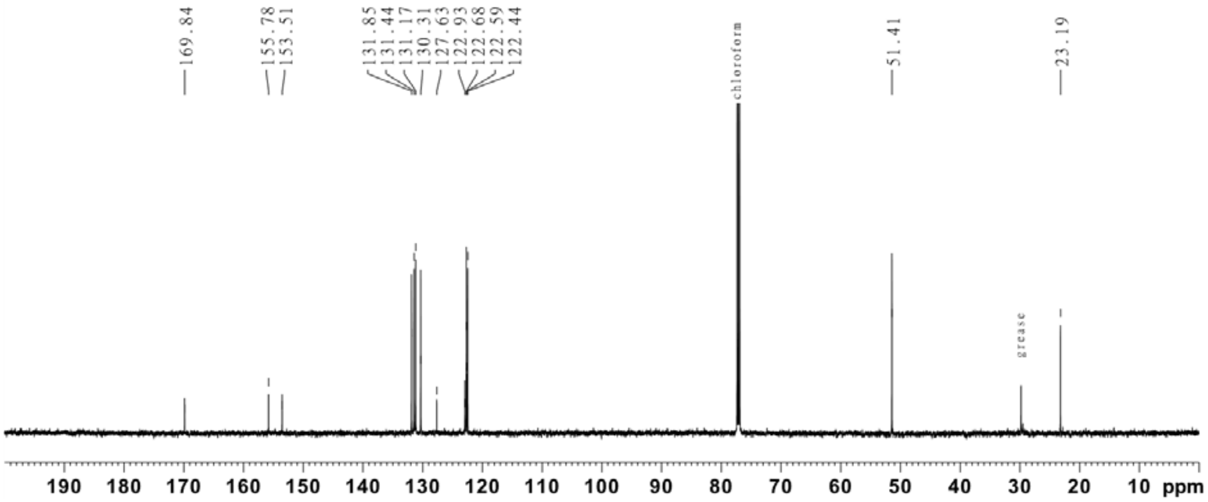


**Figure SII.44:** 125 MHz $^{13}C\{^{1}H\}$-NMR spectrum of (Z)-(3,8-dibromodibenzo[*c*,*g*][1,2,5]triazocin-11(12H)-yl)ethan-1-one (**4**).

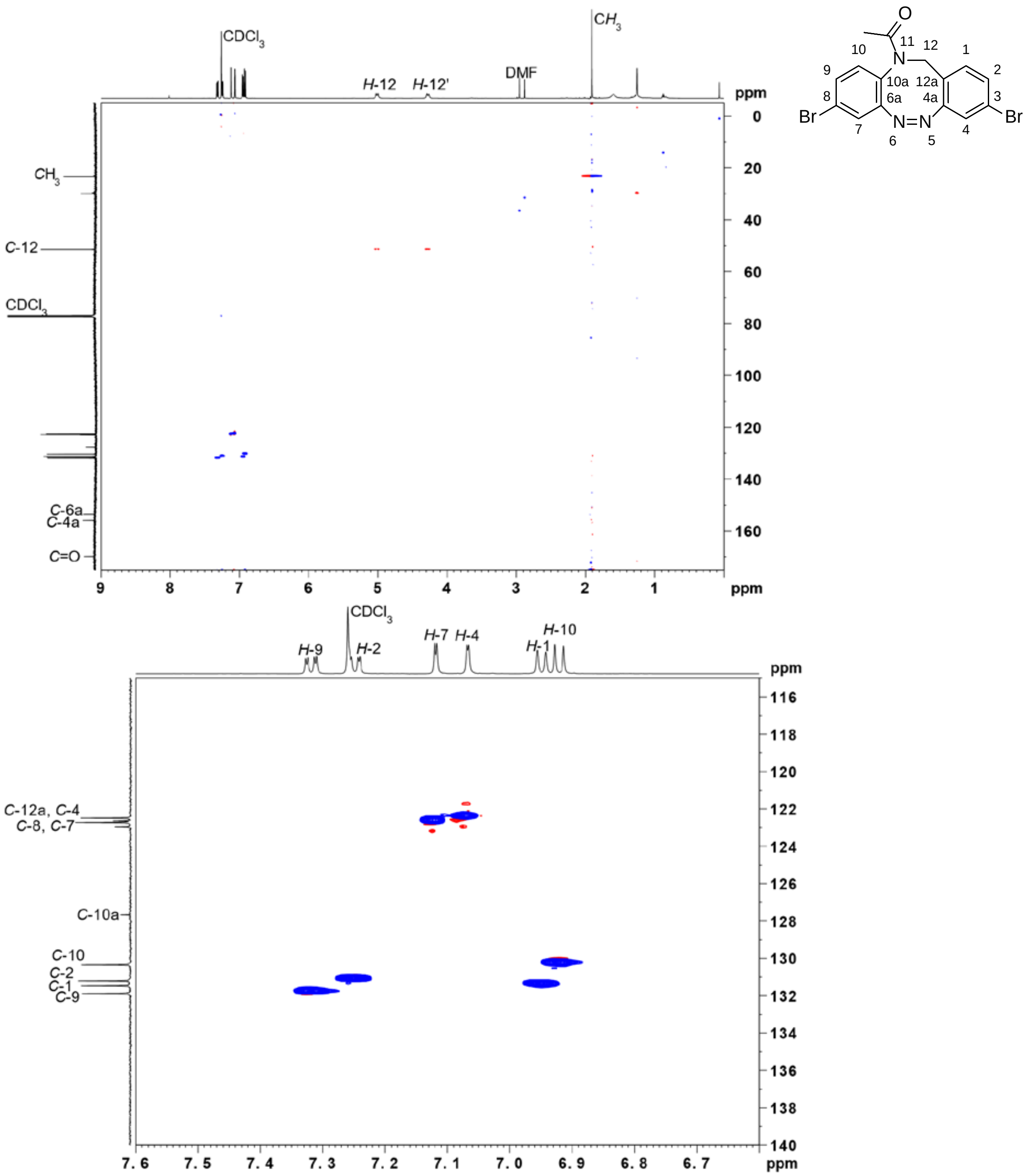


**Figure SII.45:** 500 MHz $^{1}H$-$^{13}C$ HSQC spectrum of (Z)-(3,8-dibromodibenzo[*c*,*g*][1,2,5]triazocin-11(12H)-yl)ethan-1-one (**4**).

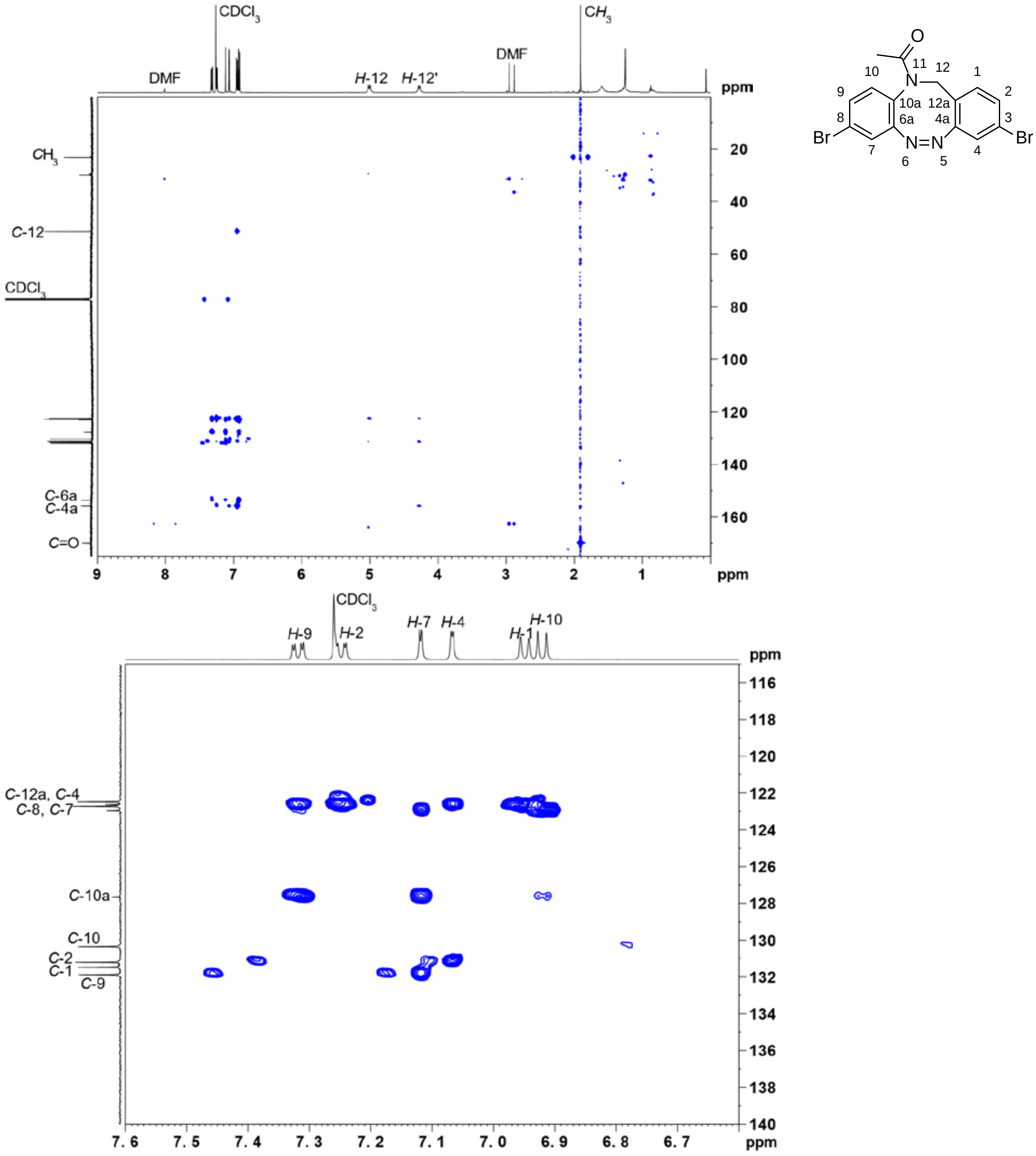


**Figure SII.46:** 500 MHz HMBC spectrum of (Z)-(3,8-dibromodibenzo[*c*,*g*][1,2,5]triazocin-11(12H)-yl)ethan-1-one (**4**).

## II.2 Syntheses of diazocine derivatives via cross-coupling reactions

### II.2.1 Synthesis of (*Z*)-1-(3-phenyldibenzo[*c,g*][1,2,5]triazocin-11(12*H*)-yl)ethan-1-one (7)

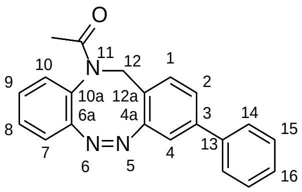


**Preparation via Stille-coupling:**

Halogenated *N*-acetyl-diazocine (**2**: 20.0 mg, 60.6 µmol, **3**: 23.0 mg, 60.6 µmol) and $Pd(PtBu_3)_2$ (3.1 mg, 6.06 µmol) were dissolved in 2 mL anhydrous THF and stirred at rt. for 10 min. $Ph_3SnCl$ (23.4 mg, 60.6 µmol) was added and the reaction mixture was heated to 65 °C with an oil bath and stirred for 16 h at given temperature. After cooling to rt. deionized water and DCM were added and the reaction mixture was extracted with 10 mL DCM twice. The combined organic layers were dried over $MgSO_4$ and the solvent was evaporated. Column chromatography on silica (ethyl acetate/cyclohexane 1:1, $R_f = 0.39$) gave a mixture of starting material and product. The yield was determined via NMR (from bromide **2**: 1.9 mg, 5.67 µmol, 10%; from iodide **3**: 1.9 mg, 5.67 µmol, 10%).

**Preparation via Suzuki-coupling:**

Halogenated *N*-acetyl-diazocine (**2**: 20.0 mg, 60.6 µmol, **3**: 23.0 mg, 60.6 µmol), phenylboronic acid (14.8 mg, 121 µmol), $Pd(PPh_3)_4$ (2.45 mg, 2.12 µmol) and potassium carbonate (33.4 mg, 212 µmol) were dissolved in 2.4 mL of toluene/MeOH/deionized $H_2O$ (5 : 1 : 2) under a nitrogen atmosphere, heated to 85 °C with an oil bath and stirred for 16 h at given temperature. After cooling to rt. the reaction mixture was extracted with 10 mL of DCM twice, dried over $MgSO_4$ and the solvent was evaporated. Column chromatography on silica (ethyl acetate/cyclohexane 1:1, $R_f = 0.39$) gave the product as yellow solid (from bromide **2**: 14.7 mg, 44.8 µmol, 74%; from iodide **3**: 16.5 mg, 50.3 µmol, 83%).

**melting point:** 187 °C

**$^1$H-NMR** (600 MHz, acetone-$d_6$, 298 K): $\delta$ = 7.61 (d, $^3J$ = 8.4 Hz, 2 H, *H*-14), 7.46-7.42 (m, 3 H, *H*-2, *H*-15), 7.38-7.23 (m, 5 H, *H*-16, *H*-7/8/9/10, *H*-7/8/9/10, *H*-1, *H*-8/9), 7.18 (d, $^4J$ = 1.8 Hz, 1 H, *H*-4), 7.04 (dd, $^3J$ = 7.8 Hz, $^4J$ = 1.2 Hz, 1 H, *H*-7/10), 5.11 (d, $^2J$ = 14.6 Hz, 1 H, *H*-12), 4.35 (d, $^2J$ = 14.6 Hz, 1 H, *H*-12'), 1.82 (s, 3 H, -C$H_3$) ppm.

**$^{13}$C{$^1$H}-NMR** (150 MHz, acetone-$d_6$, 298 K): $\delta$ = 169.4 (*C*=O), 156.8 (*C*-4a), 154.4 (*C*-6a), 142.1 (*C*-3), 140.0 (*C*-13), 131.5 (*C*-1), 130.13 (*C*-7/8/9/10), 130.08 (*C*-7/8/9/10), 129.84 (*C*-15), 129.69 (*C*-10a), 129.26 (*C*-8/9), 128.9 (*C*-16), 127.7 (*C*-14), 126.6 (*C*-2), 124.1 (*C*-12a), 120.0 (*C*-7/10), 118.0 (*C*-4), 51.8 (*C*-12), 23.1 (*C*$H_3$), ppm.

**IR** (ATR): $\tilde{\nu}$ = 2925 (m), 1656 (s), 1612 (w), 1590 (w), 1478 (m), 1448 (w), 1373 (s), 1335 (m), 1287 (w), 1157 (w), 1106 (w), 1076 (w), 1035 (w), 967 (w), 950 (w), 922 (w), 891 (w), 873 (w), 835 (m), 759 (s), 702 (m), 633 (m), 600 (w), 570 (m), 542 (w), 514 (m), 491 (m), 451 (w), 439 (w), 405 (m) cm$^{-1}$.

**HR-MS** (ESI, acetone): *m/z* $[M+H]^+$ calculated for $C_{21}H_{17}ON_3+H^+$: 328.1444; found: 328.1440 ± 1.35 ppm.

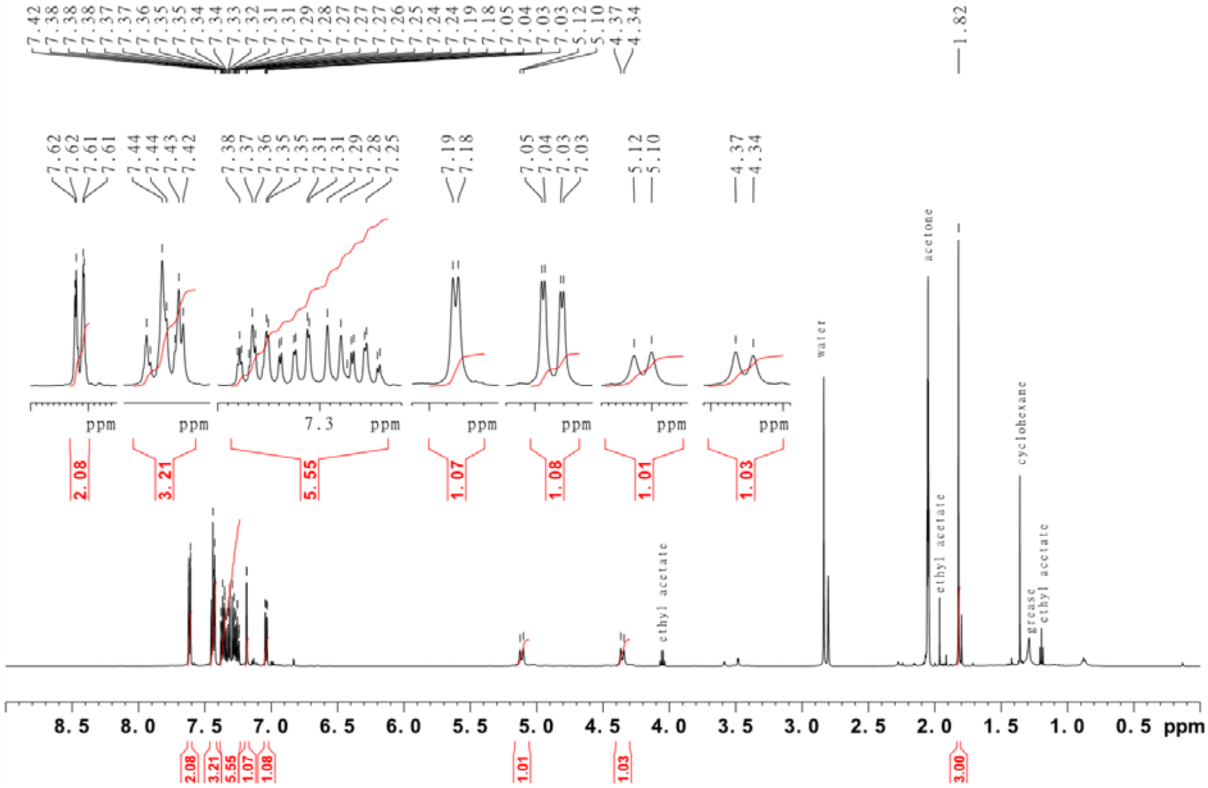


**Figure SII.47:** 600 MHz $^{1}$H-NMR spectrum of (*Z*)-1-(3-phenyldibenzo[*c*,*g*][1,2,5]triazocin-11(12*H*)-yl)ethan-1-one (**7**).

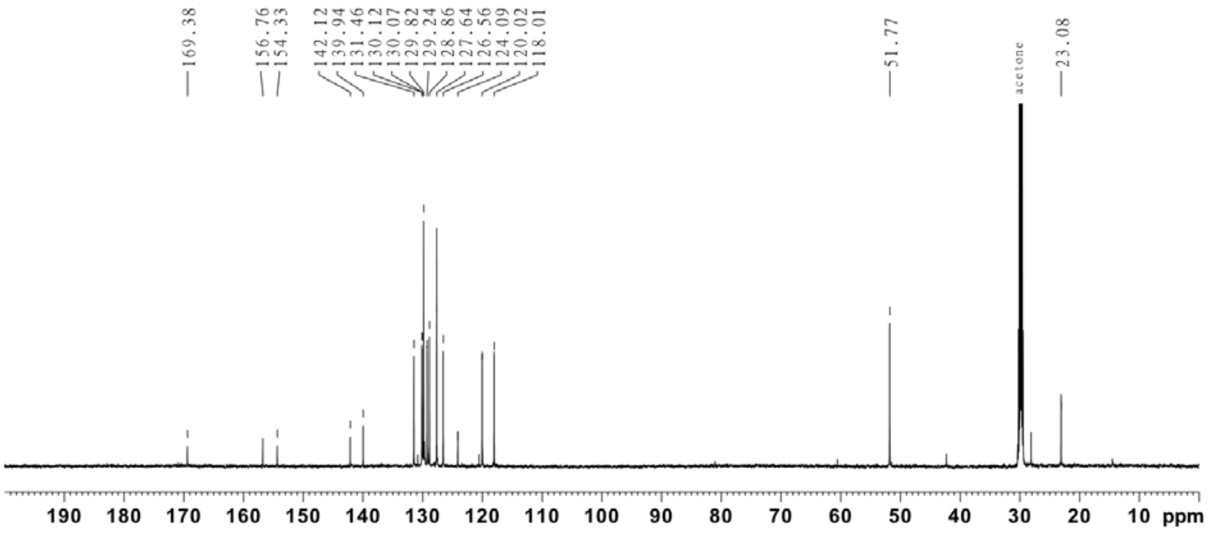


**Figure SII.48:** 150 MHz $^{13}$C{$^{1}$H}-NMR spectrum of (*Z*)-1-(3-phenyldibenzo[*c*,*g*][1,2,5]triazocin-11(12*H*)-yl)ethan-1-one (**7**).

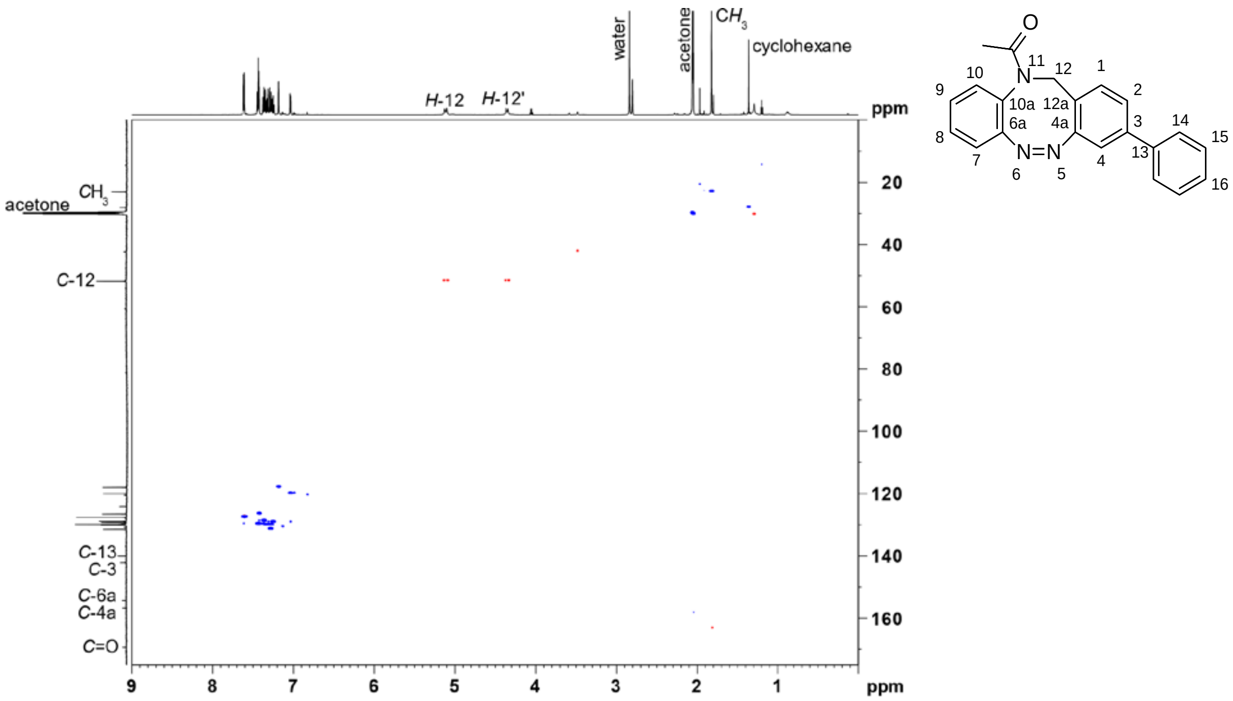


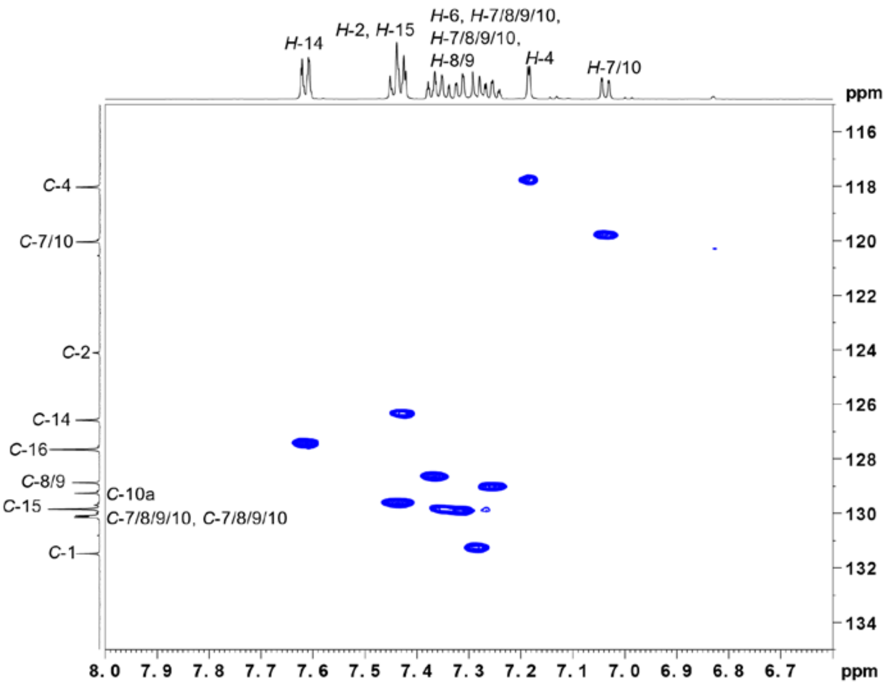


**Figure SII.49:** 600 MHz $^{1}H$-$^{13}C$ HSQC spectrum of (*Z*)-1-(3-phenyldibenzo[*c*,*g*][1,2,5]triazocin-11(12*H*)-yl)ethan-1-one (**7**).

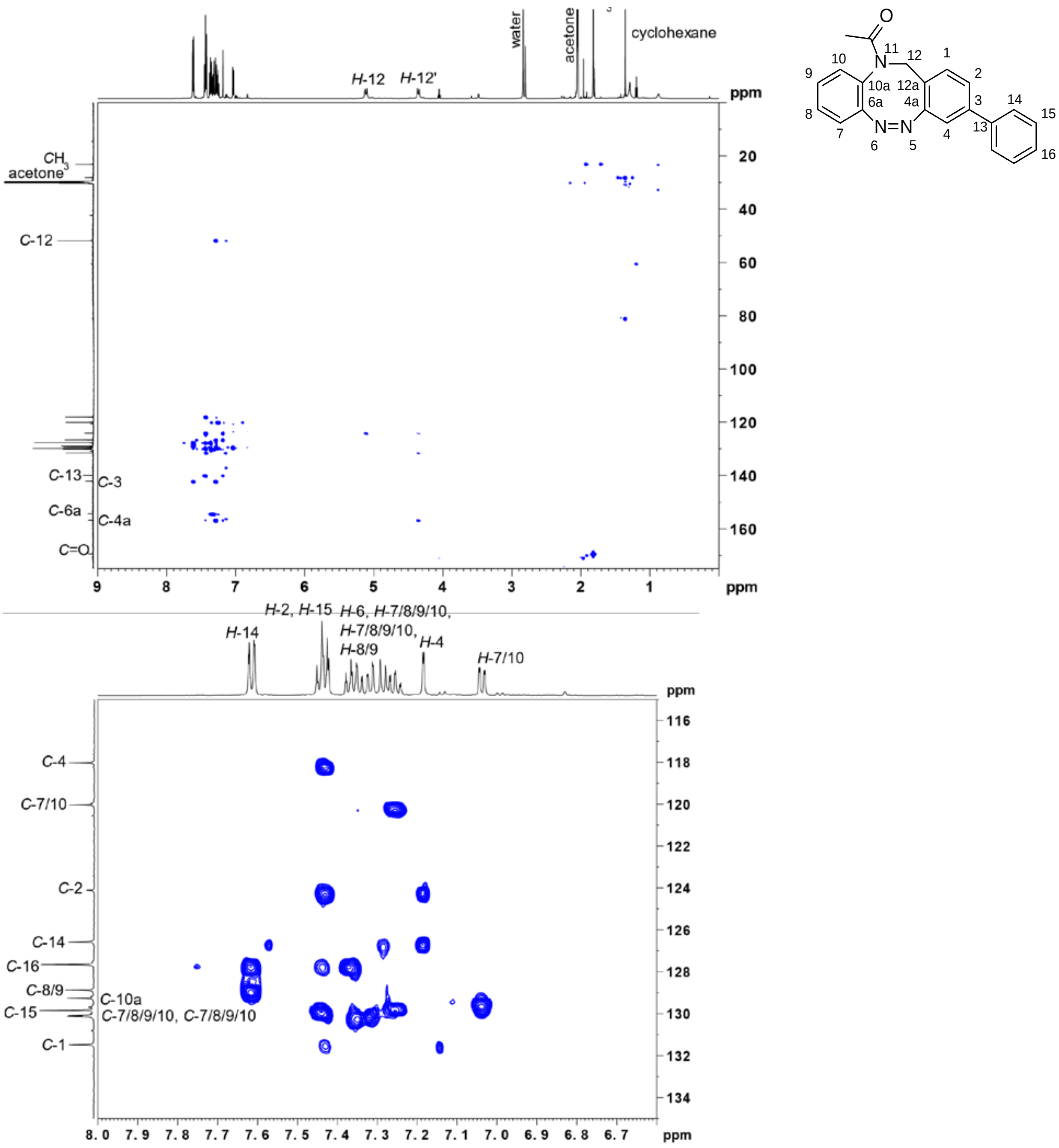


**Figure SII.50:** 600 MHz HMBC spectrum of (*Z*)-1-(3-phenyldibenzo[*c,g*][1,2,5]triazocin-11(12*H*)-yl)ethan-1-one (**7**).

## II.2.2 Synthesis of (*Z*)-1-(3-vinyldibenzo[*c,g*][1,2,5]triazocin-11(12*H*)-yl)ethan-1-one (8)

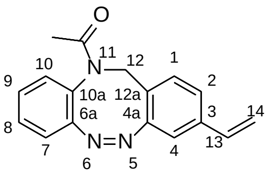


**Preparation via Stille-coupling:**

Halogenated *N*-acetyl-diazocine (**2**: 20.0 mg, 60.6 µmol, **3**: 23.0 mg, 60.6 µmol), $Pd(OAc)_2$ (1.36 mg, 6.06 µmol)and $PPh_3$ (12.7 mg, 48.5 µmol) were dissolved in 2 mL anhydrous DMF and stirred at rt. for 15 min. vinyltributyltin (57.7 mg, 181 µmol) was added and the reaction mixture was heated to 100 °C with an oil bath and stirred for 16 h at given temperature. After cooling to rt. brine and ethyl acetate were added and the organic layer was separated. The aqueous layer was extracted with 10 mL ethyl acetate twice. The combined organic layers were dried over $MgSO_4$ and the solvent was evaporated. Column chromatography on silica (ethyl acetate/cyclohexane 1:1, $R_f$ = 0.41) gave the product as yellow solid (from bromide **2**: 10.9 mg, 39.4 µmol, 65%; from iodide **3**: 11.8 mg, 43.0 µmol, 71%).

**Tin-free preparation following the DENMARK-procedure[3]:**

PdBr2 (1.60 mg, 6.06 µmol) and JohnPhos (1.8 mg, 6.06 µmol) were suspended in 2 mL anhydrous THF, 1,3,5,7-tetramethyl-1,3,5,7-tetravinylcyclotetrasiloxane ($D_4^V$, 13.4 µL, 38.8 µmol) and TBAF (1 M in THF, 242 µL, 242 µmol) were added and the reaction mixture was stirred for 10 min. at rt. The halogenated *N*-acetyl diazocine (**2**: 20.0 mg, 60.6 µmol, **3**: 23.0 mg, 60.6 µmol) was added, the reaction mixture was heated to 50 °C with an oil bath, stirred for 16 h at given temperature and the solvent was evaporated subsequently. After column chromatography on silica (ethyl acetate/cyclohexane, $R_f$ = 0.39) the product could be obtained as yellow solid (from bromide **2**: 12.4 mg, 44.8 µmol, 74%; from iodide **3**: 13.0 mg, 46.9 µmol, 78%).

**melting point:** 144 °C

**$^1$H-NMR** (500 MHz, acetone-$d_6$, 298 K): $\delta$ = 7.35 (td, $^3J$ = 7.6 Hz, $^4J$ = 1.4 Hz, 1 H, *H*-8), 7.29 (dd, $^3J$ = 7.9 Hz, $^4J$ = 1.7 Hz, 1 H, *H*-10), 7.25 (dd, $^3J$ = 7.3 Hz, $^4J$ = 1.4 Hz, 1 H, *H*-7), 7.22 (dd, $^3J$ = 7.9 Hz, $^4J$ = 1.8 Hz, 1 H, *H*-2), 7.17 (d, $^3J$ = 7.9 Hz, 1 H, *H*-1), 7.02-6.97 (m, 2 H, *H*-4, *H*-9), 6.68 (dd, $^2J$ = 17.6 Hz, $^3J$ = 10.9 Hz, 1 H, *H*-13), 5.81 (dd, $^2J$ = 17.6 Hz, $^4J$ = 0.7 Hz, 1 H, *H*-14), 5.27 (dd, $^3J$ = 10.9 Hz, $^4J$ = 0.7 Hz, 1 H, *H*-14'), 5.04 (d, $^2J$ = 14.5 Hz, 1 H, *H*-12), 4.30 (d, $^2J$ = 14.7 Hz, 1 H, *H*-12'), 1.80 (s, 3 H, -C*H*3) ppm.

**$^{13}$C{$^1$H}-NMR** (125 MHz, acetone-$d_6$, 298 K): $\delta$ = 169.3 (*C*=O), 156.5 (*C*-4a), 154.3 (*C*-6a), 139.0 (*C*-3), 136.3 (*C*-13), 131.1 (*C*-1), 130.11 (*C*-10), 130.04 (*C*-8), 129.7 (*C*-10a), 129.2 (*C*-7), 126.0 (*C*-2), 124.5 (*C*-12a), 120.0 (*C*-9), 117.4 (*C*-4), 116.0 (*C*-14), 51.8 (*C*-12), 23.1 (*C*$H_3$), ppm.

**IR** (ATR): $\tilde{\nu}$ = 2956 (w), 2922 (m), 1651 (s), 1591 (w), 1513 (w), 1479 (m), 1450 (w), 1431 (w), 1378 (s), 1339 (s), 1303 (m), 1252 (w), 1147 (w), 1109 (w), 1077 (w), 1031 (w), 995 (m), 962 (w), 926 (w), 911 (m), 885 (w), 845 (m), 766 (m), 742 (m), 665 (m), 630 (w), 601 (w), 585 (m), 570 (w), 529 (w) $cm^{-1}$.

**HR-MS** (ESI, acetone): *m/z* $[M+H]^+$ calculated for $C_{17}H_{15}ON_3+H^+$: 278.1288 found: 278.1287 ± 0.49 ppm.

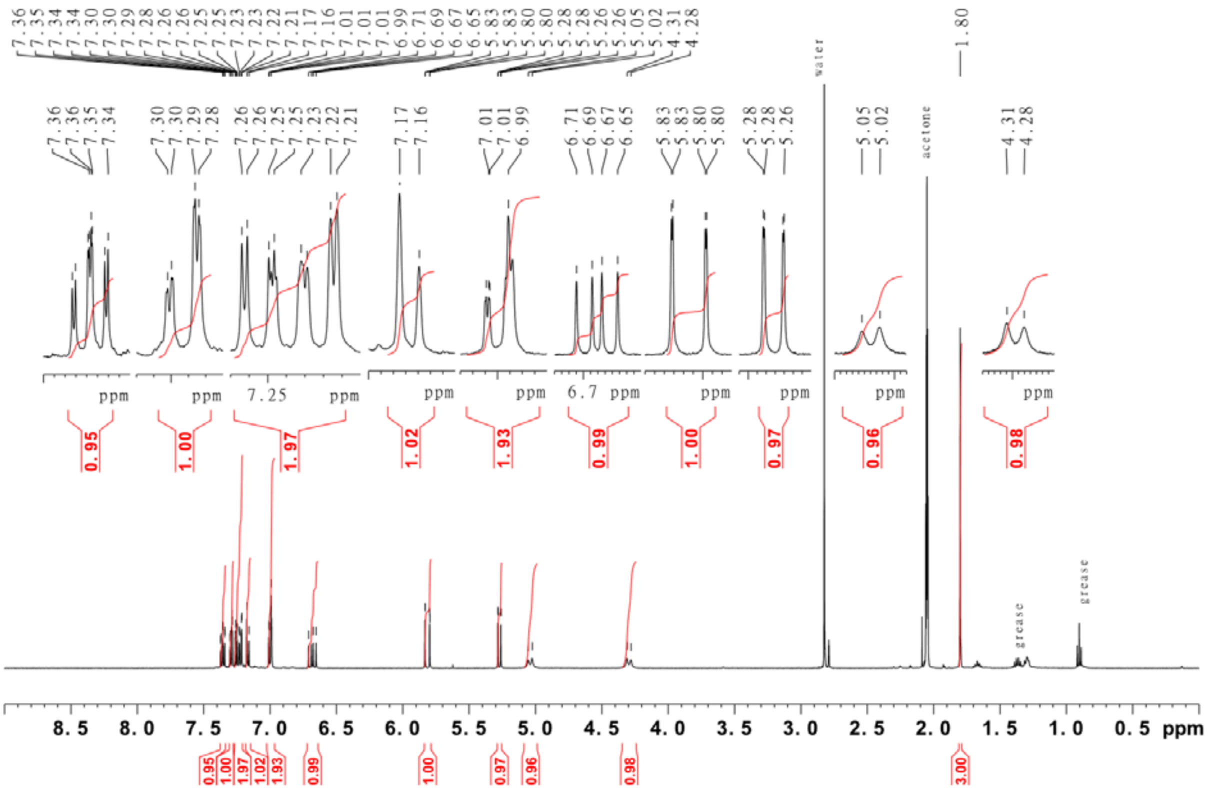

**Figure SII.51:** 500 MHz $^{1}$H-NMR spectrum of (*Z*)-1-(3-vinyldibenzo[*c*,*g*][1,2,5]triazocin-11(12*H*)-yl)ethan-1-one (**8**).

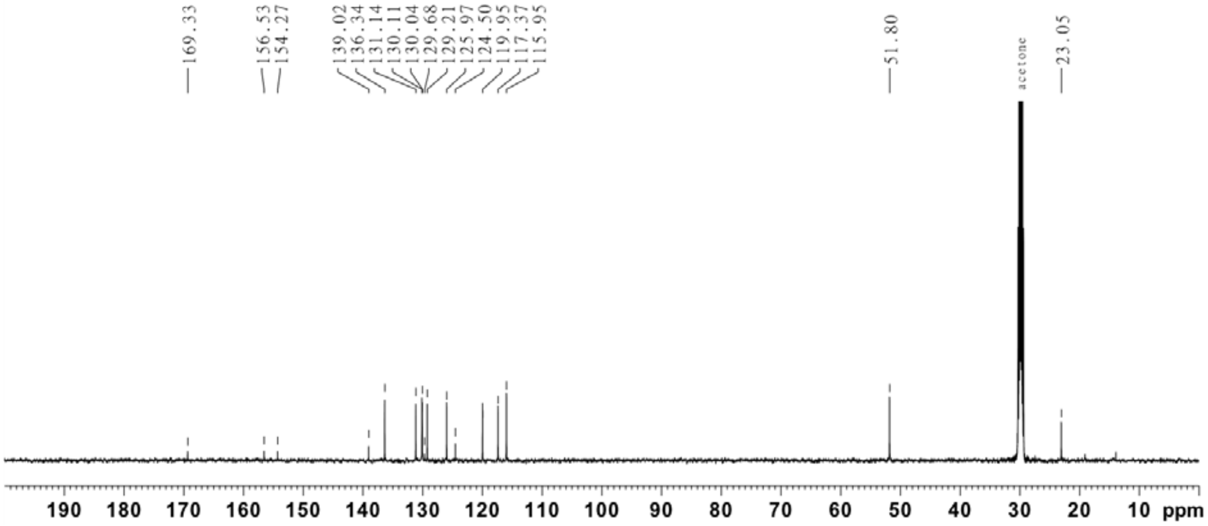

**Figure SII.52:** 125 MHz $^{13}$C{$^{1}$H}-NMR spectrum of (*Z*)-1-(3-vinyldibenzo[*c*,*g*][1,2,5]triazocin-11(12*H*)-yl)ethan-1-one (**8**).

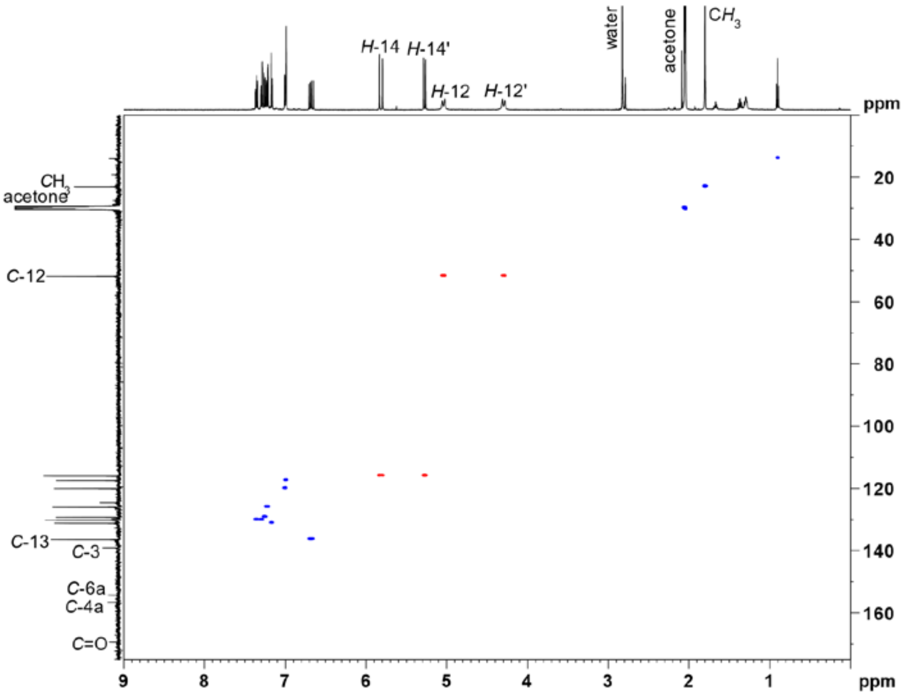


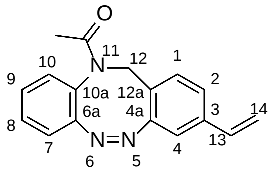


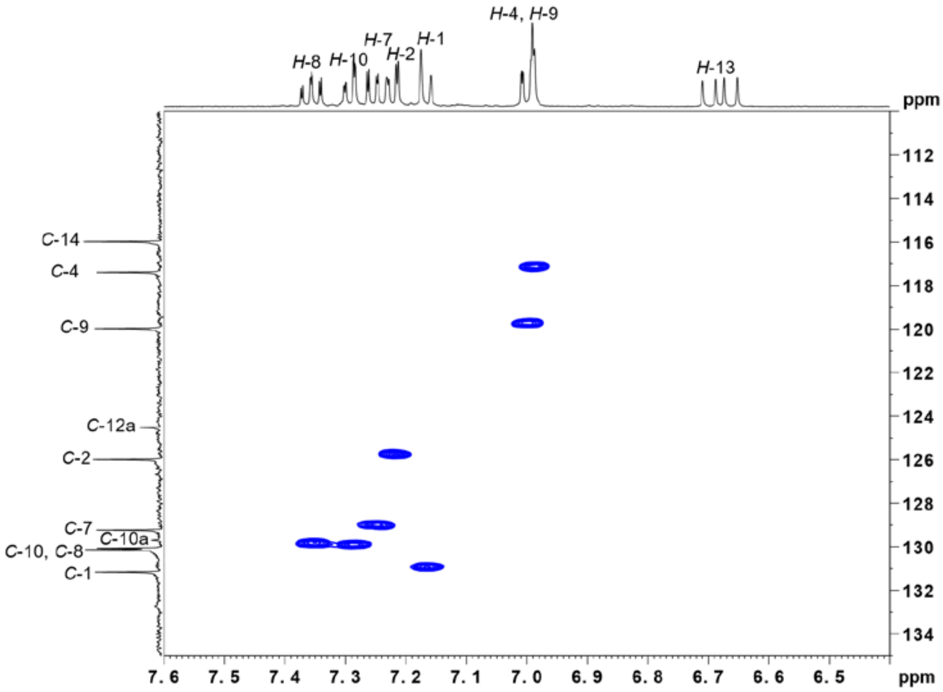


**Figure SII.53:** 500 MHz $^{1}H$-$^{13}C$ HSQC spectrum of (*Z*)-1-(3-vinyldibenzo[*c*,*g*][1,2,5]triazocin-11(12*H*)-yl)ethan-1-one (**8**).

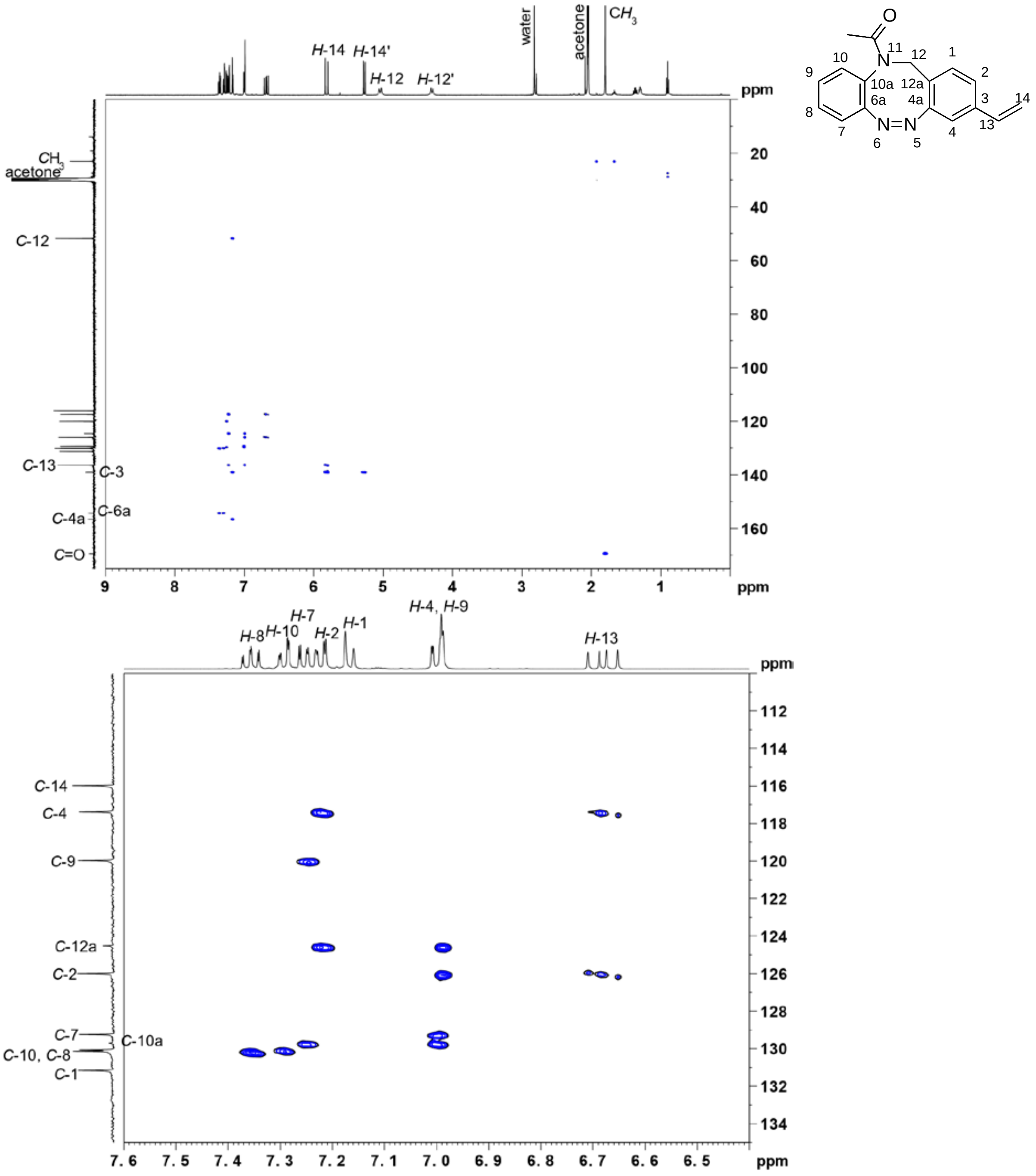


**Figure SII.54:** 500 MHz HMBC spectrum of (*Z*)-1-(3-vinyldibenzo[*c*,*g*][1,2,5]triazocin-11(12*H*)-yl)ethan-1-one (**8**).

## II.2.3 Synthesis of (*Z*)-1-(3-(2,6-dimethylphenyl)dibenzo[*c,g*][1,2,5]triazocin-11(12*H*)-yl)ethan-1-one (9)

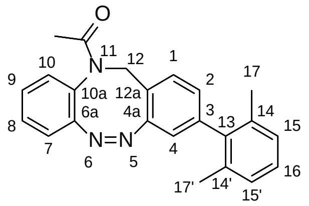


Halogenated *N*-acetyl-diazocine (**2**: 20.0 mg, 60.6 µmol, **3**: 23.0 mg, 60.6 µmol), phenylboronic acid (18.2 mg, 121 µmol), $Pd(PPh_3)_4$ (2.45 mg, 2.12 µmol) and potassium carbonate (33.4 mg, 212 µmol) were dissolved in 2.4 mL of toluene/MeOH/deionized $H_2O$ (5 : 1 : 2) under a nitrogen atmosphere, heated to 85 °C with an oil bath and stirred for 16 h at given temperature. After cooling to rt. the reaction mixture was extracted with 10 mL of DCM twice, dried over $MgSO_4$ and the solvent was evaporated. Column chromatography on silica (ethyl acetate/cyclohexane 1:1, $R_f$ = 0.40) gave the product as yellow solid (from bromide **2**: 14.6 mg, 41.1 µmol, 68%; from iodide **3**: 15.9 mg, 44.8 µmol, 74%).

**melting point:** 203 °C

**$^1$H-NMR** (600 MHz, acetone-$d_6$, 298 K): $\delta$ = 7.35 (td, $^3J$ = 7.5 Hz, $^4J$ = 1.5 Hz, 1 H, *H*-8), 7.29-7.24 (m, 3 H, *H*-1, *H*-9, *H*-10), 7.12 (t, $^3J$ = 7.3 Hz, 1 H, *H*-14), 7.10 (d, $^3J$ = 7.2 Hz, 1 H, *H*-15), 7.01 (d, $^3J$ = 6.6 Hz, 1 H, *H*-15'), 6.99 (dd, $^3J$ = 7.9 Hz, $^4J$ = 1.1 Hz, 1 H, *H*-7), 6.88 (dd, $^3J$ = 7.7 Hz, $^4J$ = 1.7 Hz, 1 H, *H*-2), 6.60 (d, $^4J$ = 1.6 Hz, 1 H, *H*-4), 5.01 (d, $^2J$ = 14.2 Hz, 1 H, *H*-12), 4.38 (d, $^2J$ = 14.3 Hz, 1 H, *H*-12'), 2.08 (s, 3 H, *H*-17), 1.80 (s, 3 H, -C$H_3$), 1.55 (s, 3 H, -17') ppm.

**$^{13}$C{$^1$H}-NMR** (150 MHz, acetone-$d_6$, 298 K): $\delta$ = 169.3 (*C*=O), 157.1 (*C*-4a), 154.7 (*C*-6a), 142.6 (*C*-3), 141.0 (*C*-13), 136.5 (*C*-14'), 136.1 (*C*-14), 131.3 (*C*-1), 130.3 (*C*-10), 130.0 (*C*-10a), 129.8 (*C*-8), 129.0 (*C*-9), 128.8 (*C*-2), 128.30 (*C*-16), 128.21 (*C*-15), 128.17 (*C*-15'), 123.6 (*C*-12a), 119.72 (*C*-4), 119.64 (*C*-7), 52.0 (*C*-12), 23.2 ($CH_3$), 20.8 (*C*-17), 20.4 (*C*-17') ppm.

**IR** (ATR): $\tilde{\nu}$ = 3751 (w), 2922 (m), 2300 (w), 1728 (w), 1651 (s), 1613 (w), 1589 (w), 1509 (w), 1482 (m), 1464 (w), 1380 (s), 1339 (s), 1297 (w), 1244 (w), 1169 (w), 1104 (w), 1069 (m), 1033 (m), 987 (w), 966 (m), 928 (w), 877 (m), 856 (w), 833 (s), 777 (s), 742 (m), 663 (w), 637 (m), 599 (m), 581 (m) $cm^{-1}$.

**HR-MS** (ESI, acetone): *m/z* $[M+H]^+$ calculated for $C_{23}H_{21}ON_3+H^+$: 356.1757; found: 356.1755 ± 0.75 ppm.

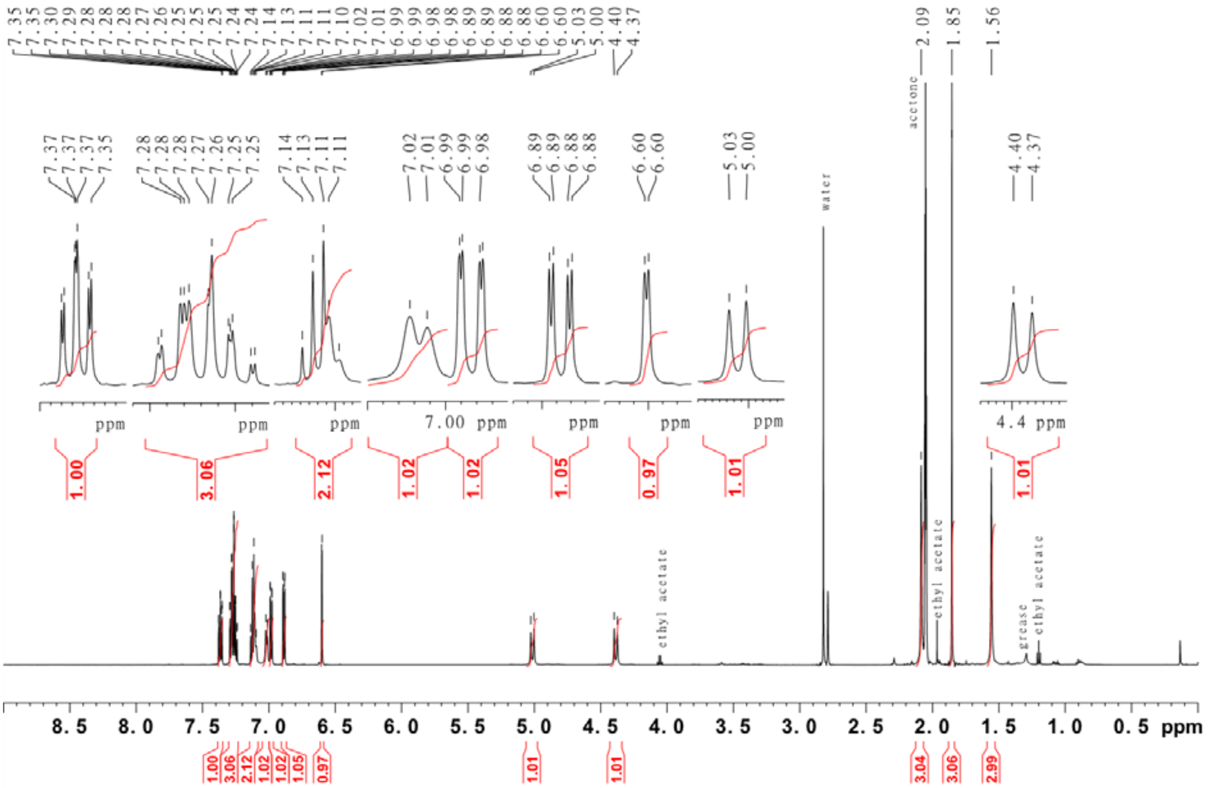

**Figure SII.55:** 600 MHz $^{1}H$-NMR spectrum of (*Z*)-1-(3-(2,6-dimethylphenyl)dibenzo[*c*,*g*][1,2,5]triazocin-11(12*H*)-yl)ethan-1-one (**9**).

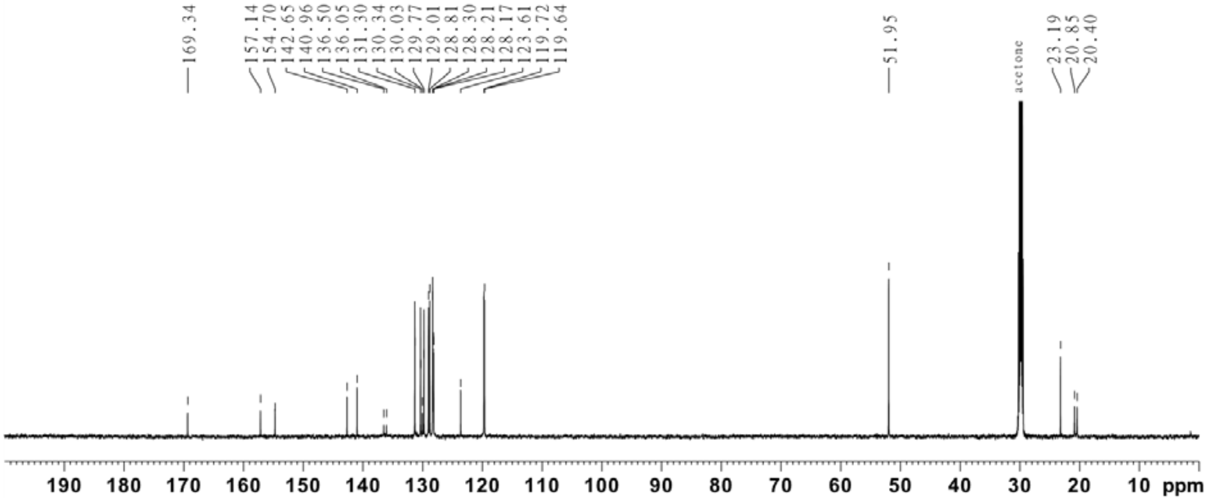

**Figure SII.56:** 150 MHz $^{13}C\{^{1}H\}$-NMR spectrum of (*Z*)-1-(3-(2,6-dimethylphenyl)dibenzo[*c*,*g*][1,2,5]triazocin-11(12*H*)-yl)ethan-1-one (**9**).

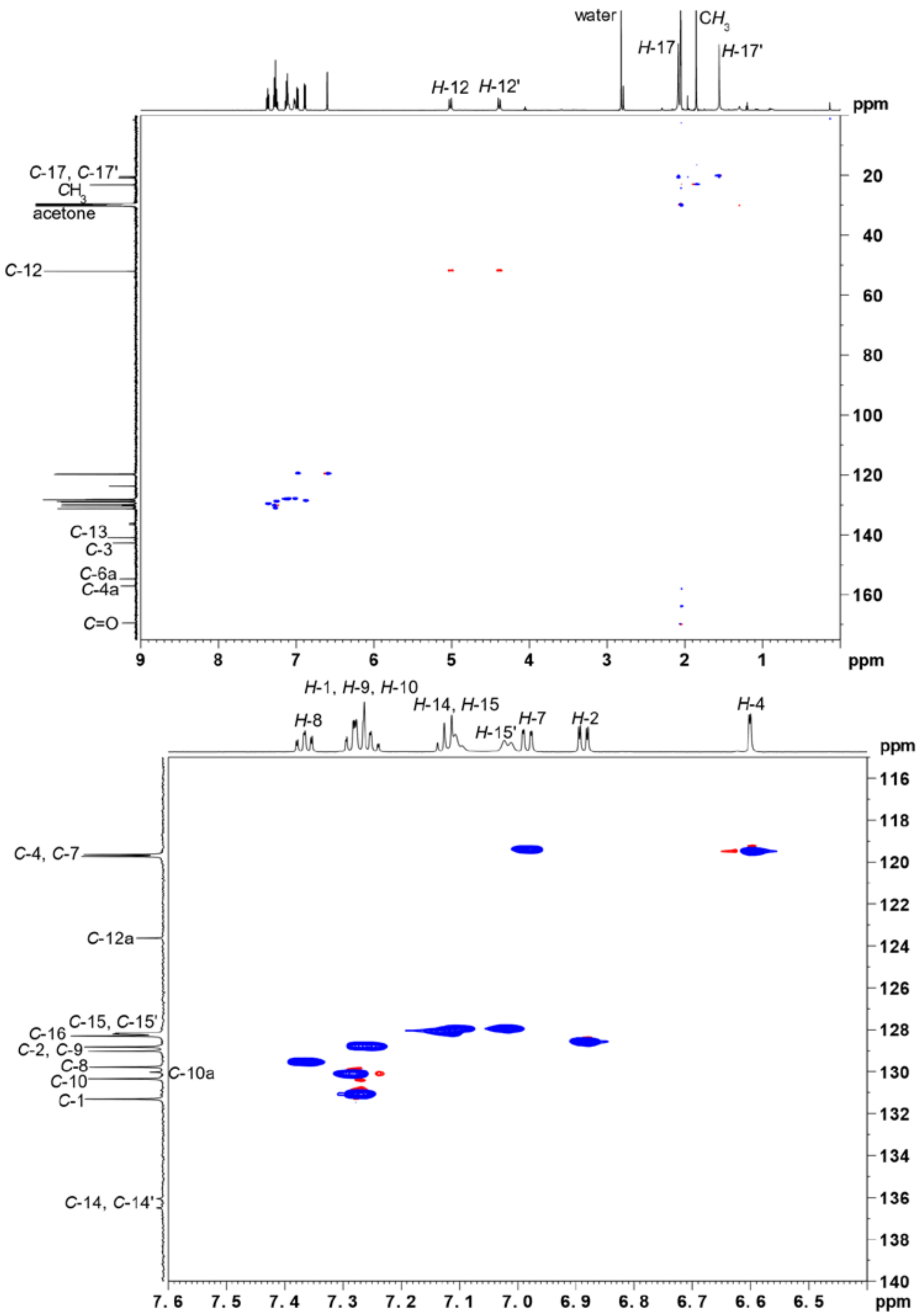


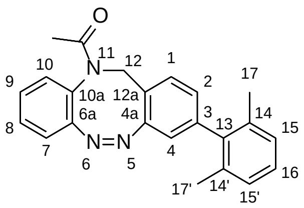


**Figure SII.57:** 600 MHz $^{1}$H-$^{13}$C HSQC spectrum of (*Z*)-1-(3-(2,6-dimethylphenyl)dibenzo[*c*,*g*][1,2,5]triazocin-11(12*H*)-yl)ethan-1-one (**9**).

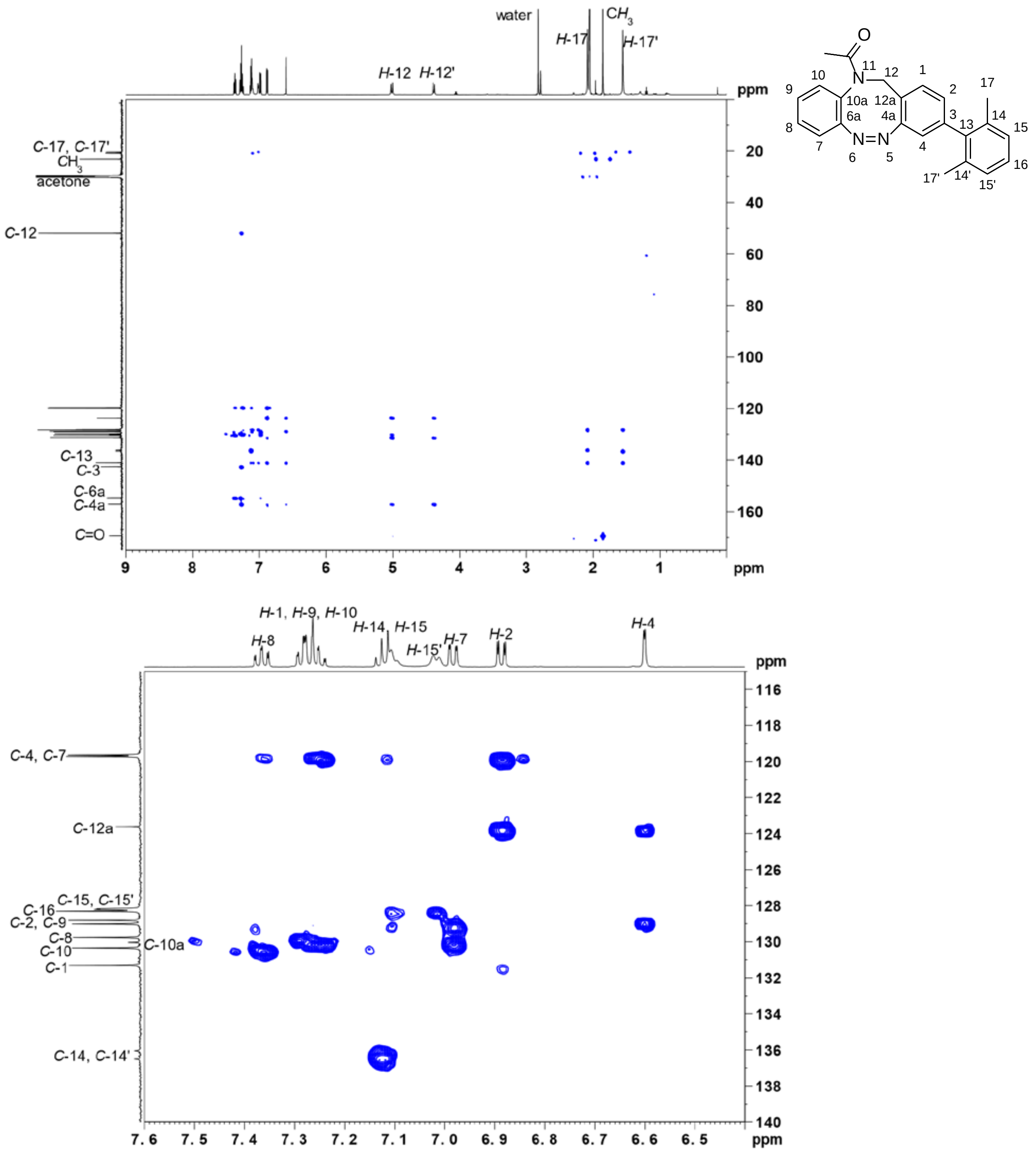


**Figure SII.58:** 600 MHz HMBC spectrum of (*Z*)-1-(3-(2,6-dimethylphenyl)dibenzo[*c*,*g*][1,2,5]triazocin-11(12*H*)-yl)ethan-1-one (**9**).

## II.2.4 Synthesis of (*Z*)-1-(3-(2-bromophenyl)dibenzo[*c,g*][1,2,5]triazocin-11(12*H*)-yl)ethan-1-one (10)

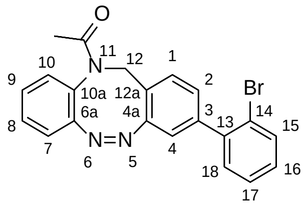


Halogenated *N*-acetyl-diazocine (**3**: 23.0 mg, 60.6 µmol), (2-bromophenyl)boronic acid (24.3 mg, 121 µmol), $Pd(PPh_3)_4$ (2.45 mg, 2.12 µmol) and potassium carbonate (33.4 mg, 212 µmol) were dissolved in 2.4 mL of toluene/MeOH/deionized $H_2O$ (5 : 1 : 2) under a nitrogen atmosphere, heated to 85 °C with an oil bath and stirred for 16 h at given temperature. After cooling to rt. the reaction mixture was extracted with 10 mL of DCM twice, dried over $MgSO_4$ and the solvent was evaporated. Column chromatography on silica (ethyl acetate/cyclohexane 1:1, $R_f$ = 0.45) gave the product as yellow solid (from iodide **3**: 20.2 mg, 49.7 µmol, 82%).

**melting point:** 119 °C

**$^1$H-NMR** (600 MHz, acetone-$d_6$, 298 K): $\delta$ = 7.69 (d, $^3J$ = 9.5 Hz, 1 H, Ar-*H*), 7.43 (td, $^3J$ = 7.5 Hz, $^4J$ = 1.2 Hz, 1 H, Ar-*H*), 7.38 (td, $^3J$ = 7.7 Hz, $^4J$ = 1.3 Hz, 1 H, Ar-*H*), 7.33-7.25 (m, 5 H), 7.16 (dd, $^3J$ = 7.9 Hz, $^4J$ = 1.8 Hz, 1 H, *H*-2), 7.04 (dd, $^3J$ = 7.8 Hz, $^4J$ = 1.2 Hz, 1 H, Ar-*H*), 6.94 (d, $^4J$ = 1.7 Hz, 1 H, *H*-4), 5.11 (d, $^2J$ = 14.4 Hz, 1 H, *H*-12), 4.37 (d, $^2J$ = 14.6 Hz, 1 H, *H*-12'), 1.83 (s, 3 H, -C*H*$_3$) ppm.

**$^{13}$C{$^1$H}-NMR** (150 MHz, acetone-$d_6$, 298 K): $\delta$ = 169.4 (*C*=O), 156.0 (*C*-4a), 154.5 (*C*-6a), 142.2 (*C*-Ar), 141.7 (*C*-3), 134.1 (*C*-Ar), 132.0 (*C*-Ar), 130.7 (*C*-1), 130.5 (*C*-Ar), 130.15 (*C*-Ar), 130.06 (*C*-8), 129.7 (*C*-Ar), 129.3 (*C*-Ar), 128.8 (*C*-Ar), 124.6 (*C*-12a), 122.7 (*C*-Ar), 120.7 (*C*-4), 120.0 (*C*-Ar), 116.6 (*C*-Ar), 51.9 (*C*-12), 23.1 (*C*H$_3$) ppm.

**IR** (ATR): $\tilde{\nu}$ = 2922 (m), 2852 (w), 1654 (s), 1592 (w), 1485 (m), 1464 (m), 1433 (w), 1369 (s), 1334 (s), 1288 (w), 1242 (w), 1066 (w), 1023 (m), 971 (w), 933 (w), 879 (w), 842 (m), 752 (s), 729 (w), 699 (w), 659 (m), 603 (w), 569 (m), 533 (m), 430 (m) cm$^{-1}$.

**HR-MS** (ESI, DCM): *m/z* $[M+H]^+$ calculated for $C_{21}H_{16}ON_3{}^{79}Br+H^+$: 406.0550 found: 406.0547 ± 0.54 ppm.

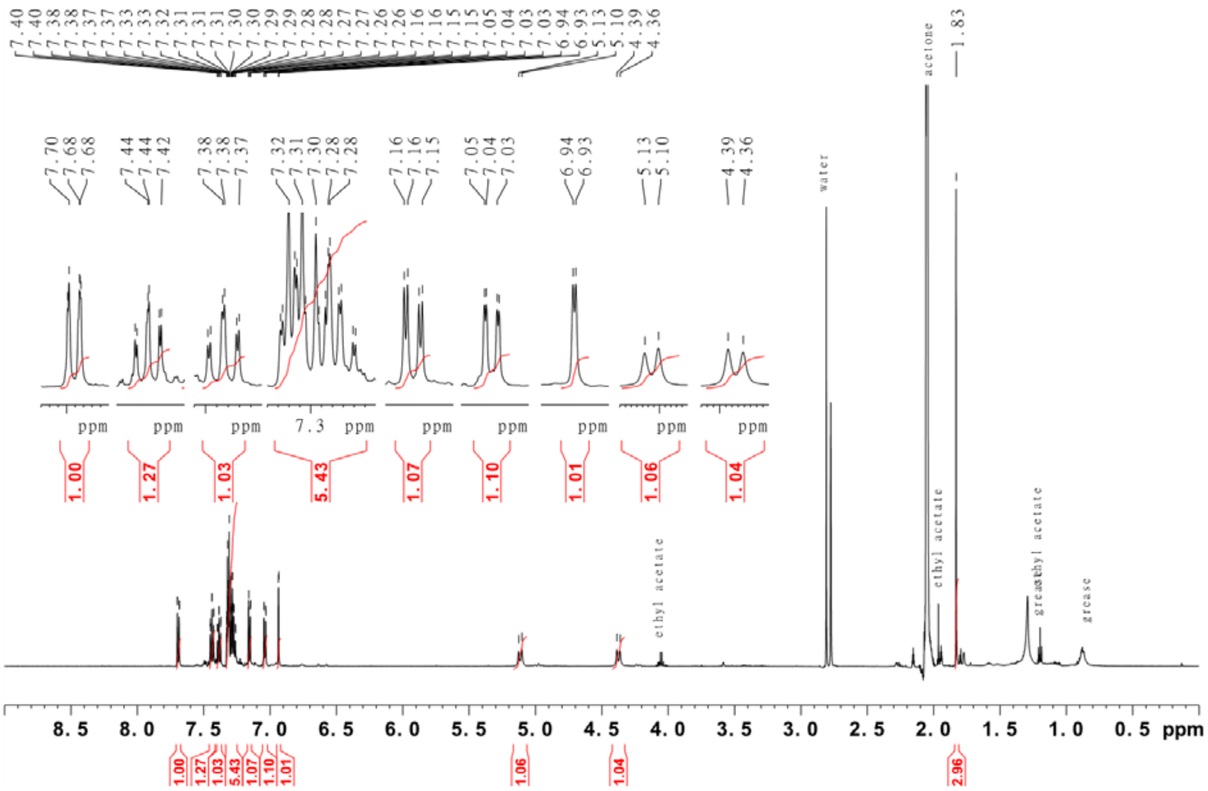

**Figure SII.59:** 600 MHz $^{1}$H-NMR spectrum of (*Z*)-1-(3-(2-bromophenyl)dibenzo[*c*,*g*][1,2,5]triazocin-11(12*H*)-yl)ethan-1-one (**10**).

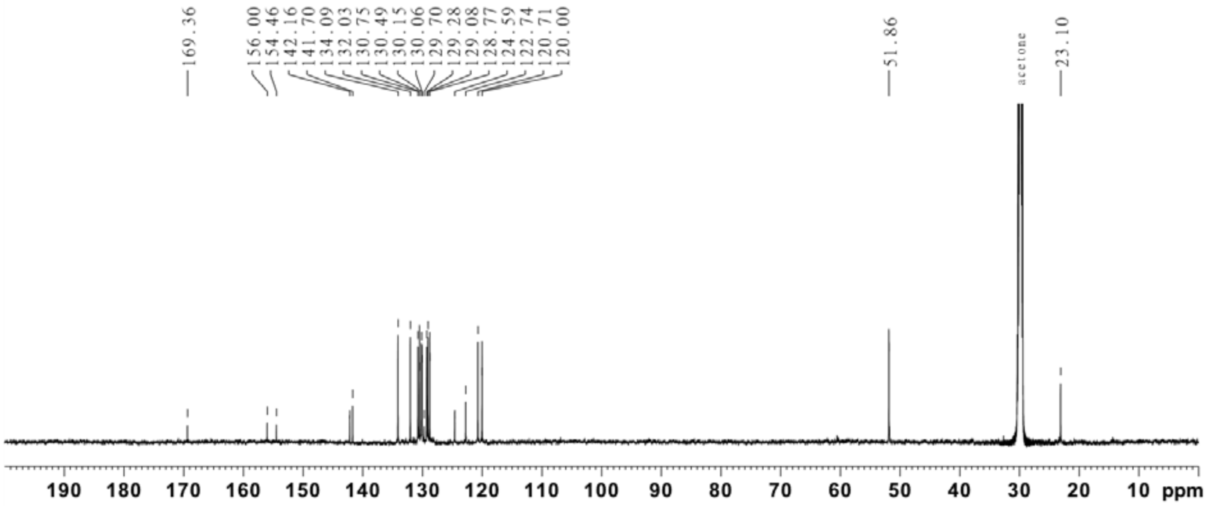

**Figure SII.60:** 150 MHz $^{13}$C{$^{1}$H}-NMR spectrum of (*Z*)-1-(3-(2-bromophenyl)dibenzo[*c*,*g*][1,2,5]triazocin-11(12*H*)-yl)ethan-1-one (**10**).

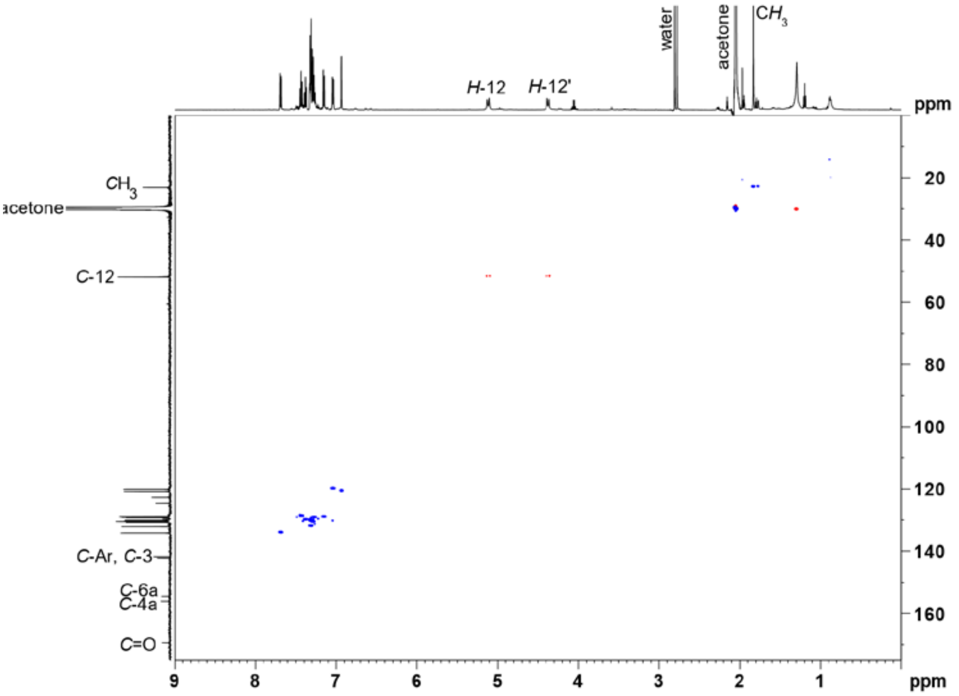


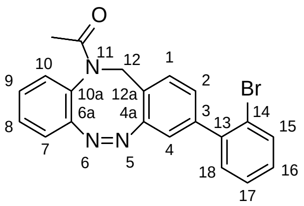


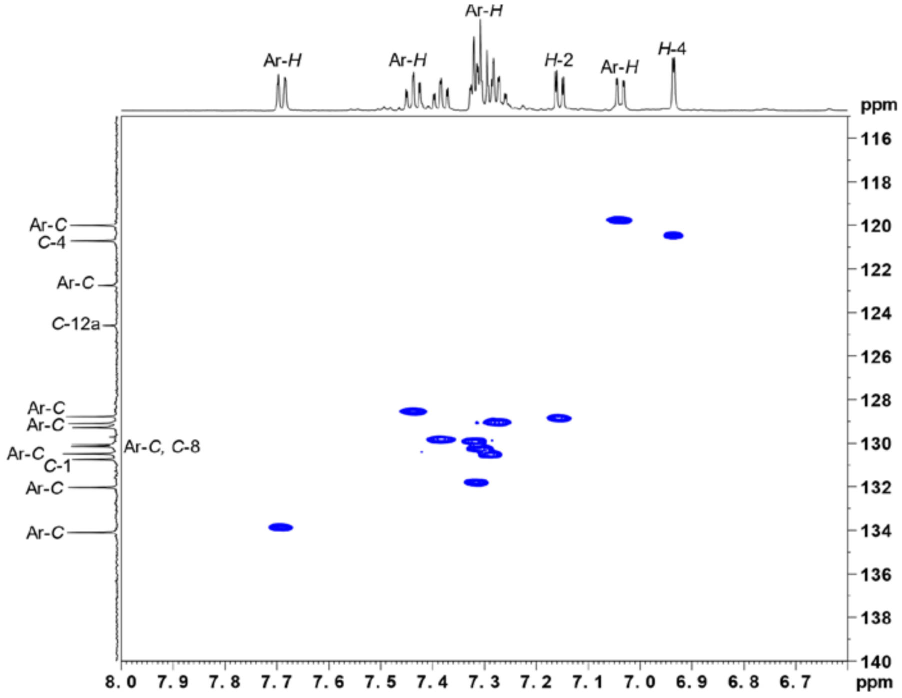


**Figure SII.61:** 600 MHz $^{1}$H-$^{13}$C HSQC spectrum of (*Z*)-1-(3-(2-bromophenyl)dibenzo[*c*,*g*][1,2,5]triazocin-11(12*H*)-yl)ethan-1-one (**10**).

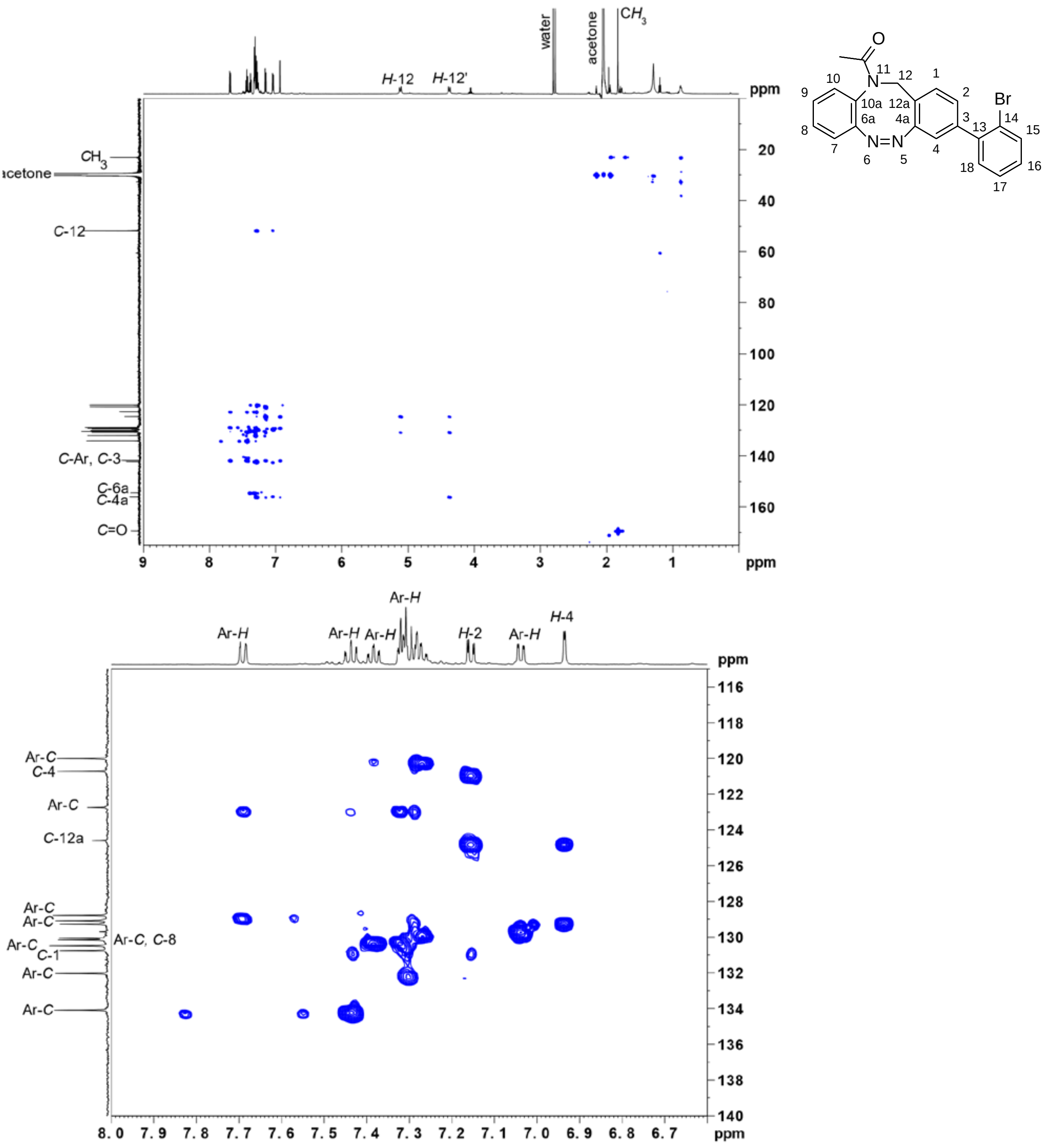


**Figure SII.62:** 600 MHz HMBC spectrum of (*Z*)-1-(3-(2-bromophenyl)dibenzo[*c*,*g*][1,2,5]triazocin-11(12*H*)-yl)ethan-1-one (**10**).

## II.2.5 Synthesis of (*Z*)-1-(3-(4-fluorophenyl)dibenzo[*c,g*][1,2,5]triazocin-11(12*H*)-yl)ethan-1-one (11)

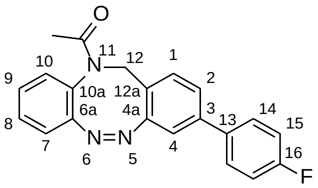


Halogenated *N*-acetyl-diazocine (**2**: 20.0 mg, 60.6 µmol, **3**: 23.0 mg, 60.6 µmol), (2,6-dimethylphenyl)boronic acid (17.0 mg, 121 µmol), $Pd(PPh_3)_4$ (2.45 mg, 2.12 µmol) and potassium carbonate (33.4 mg, 212 µmol) were dissolved in 2.4 mL of toluene/MeOH/deionized $H_2O$ (5 : 1 : 2) under a nitrogen atmosphere, heated to 85 °C with an oil bath and stirred for 16 h at given temperature. After cooling to rt. the reaction mixture was extracted with 10 mL of DCM twice, dried over $MgSO_4$ and the solvent was evaporated. Column chromatography on silica (ethyl acetate/cyclohexane 1:1, $R_f$ = 0.38) gave the product as yellow solid (from bromide **2**: 16.9 mg, 49.1 µmol, 81%; from iodide **3**: 18.4 mg, 53.3 µmol, 88%).

**melting point:** 203 °C

**$^1$H-NMR** (600 MHz, acetone-$d_6$, 298 K): $\delta$ = 7.67 (dd, $^3J$ = 8.9 Hz, $^3J$ = 5.3 Hz, 2 H, *H*-14), 7.41 (dd, $^3J$ = 7.9 Hz, $^4J$ = 1.9 Hz, 1 H, *H*-2), 7.36 (td, $^3J$ = 7.6 Hz, $^4J$ = 1.3 Hz, 1 H, *H*-8), 7.32 (dd, $^3J$ = 7.9 Hz, $^4J$ = 1.1 Hz, 1 H, *H*-10), 7.29 (d, $^3J$ = 7.9 Hz, 1 H, *H*-1), 7.26 (td, $^3J$ = 7.5 Hz, $^4J$ = 1.4 Hz, 1 H, *H*-9), 7.21 (t, $^3J$ = 8.8 Hz, 2 H, *H*-15), 7.17 (d, $^4J$ = 1.8 Hz, 1 H, *H*-4), 7.03 (dd, $^3J$ = 7.8 Hz, $^3J$ = 1.3 Hz, 1 H, *H*-7), 5.11 (d, $^2J$ = 14.6 Hz, 1 H, *H*-12), 4.35 (d, $^2J$ = 14.6 Hz, 1 H, *H*-12'), 1.81 (s, 3 H, -C$H_3$) ppm.

**$^{13}$C{$^1$H}-NMR** (150 MHz, acetone-$d_6$, 298 K): $\delta$ = 169.4 (*C*=O), 163.7 ($^1J$ = 247.3 Hz, *C*-16) 156.8 (*C*-4a), 154.4 (*C*-6a), 141.1 (*C*-3), 136.4 (*C*-13), 131.5 (*C*-1), 130.14 (*C*-10), 130.09 (*C*-8), 129.7 ($^3J$ = 8.2 Hz, *C*-14), 129.3 (*C*-9), 126.5 (*C*-2), 124.2 (*C*-12a), 120.0 (*C*-7), 118.0 (*C*-4), 116.6 ($^2J$ = 21.7 Hz, *C*-15), 51.8 (*C*-12), 23.1 ($CH_3$) ppm.

**IR** (ATR): $\tilde{\nu}$ = 3065 (w), 2924 (m), 2852 (w), 2254 (w), 2038 (w), 1654 (s), 1606 (w), 1518 (m), 1475 (m), 1382 (s), 1290 (m), 1222 (s), 1164 (m), 1107 (w), 1034 (w), 1016 (m), 974 (m), 914 (w), 878 (w), 843 (m), 809 (s), 783 (w), 759 (s), 739 (s), 703 (w), 635 (m), 593 (s), 536 (m) $cm^{-1}$.

**HR-MS** (ESI, acetone): *m/z* $[M+H]^+$ calculated for $C_{21}H_{15}FON_3+H^+$: 346.1350; found: 346.1347 ± 0.33 ppm.

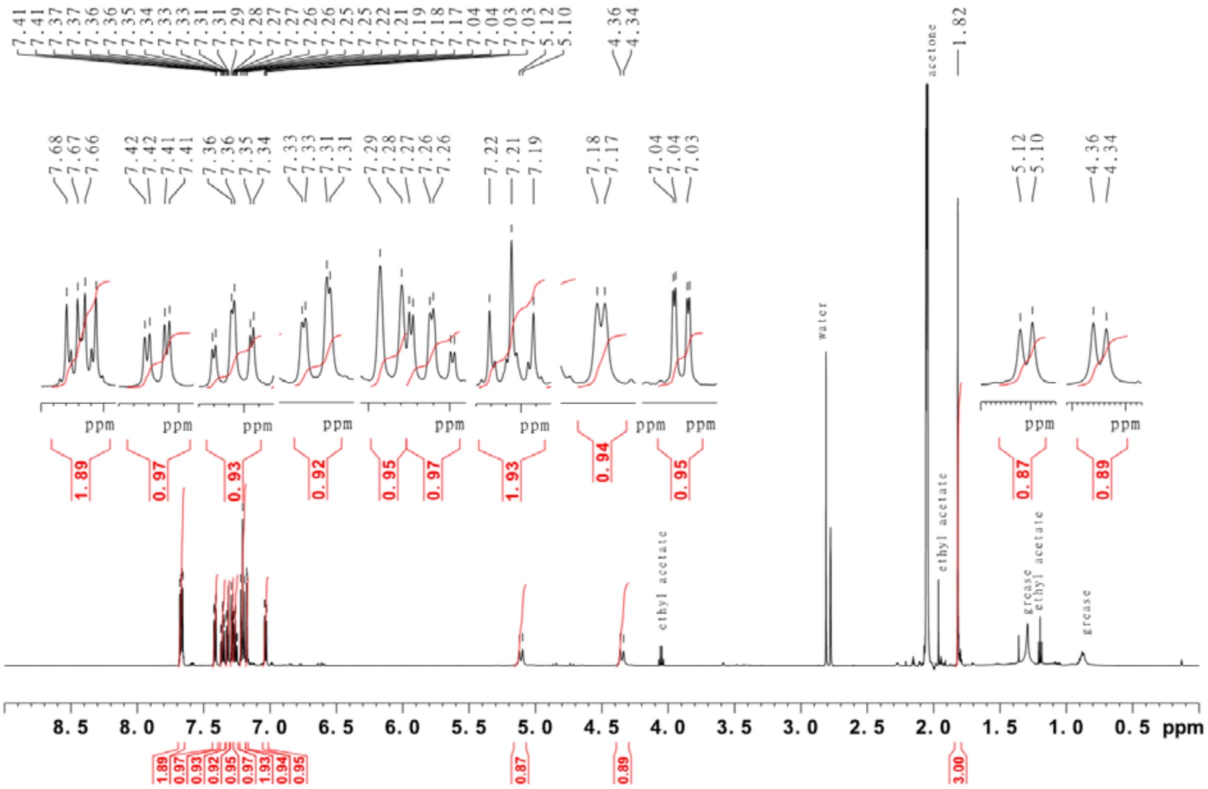


**Figure SII.63:** 600 MHz $^{1}H$-NMR spectrum of (*Z*)-1-(3-(4-fluorophenyl)dibenzo[*c,g*][1,2,5]triazocin-11(12*H*)-yl)ethan-1-one (**11**).

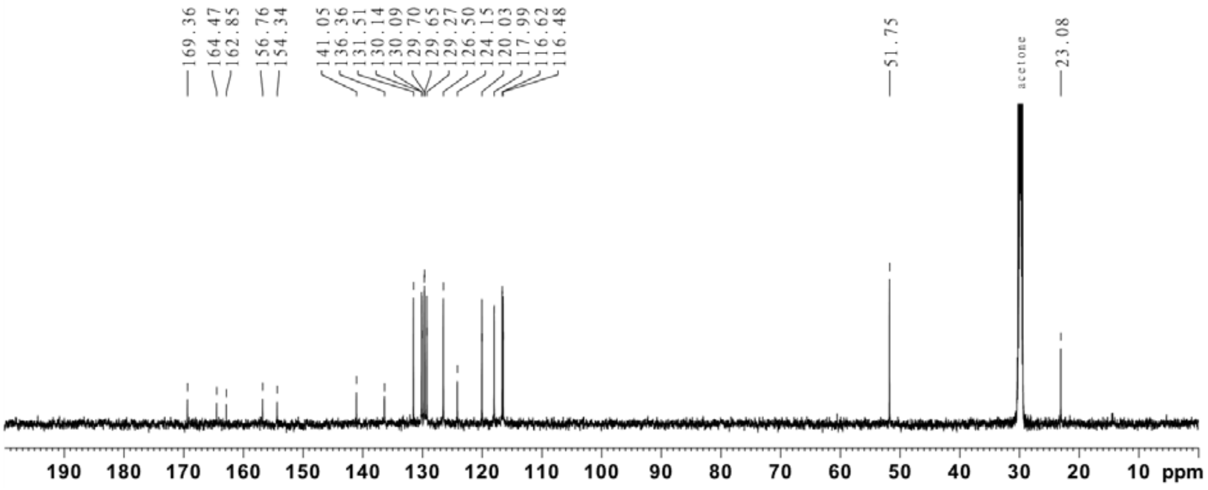


**Figure SII.64:** 150 MHz $^{13}C\{^{1}H\}$-NMR spectrum of (*Z*)-1-(3-(4-fluorophenyl)dibenzo[*c,g*][1,2,5]triazocin-11(12*H*)-yl)ethan-1-one (**11**).

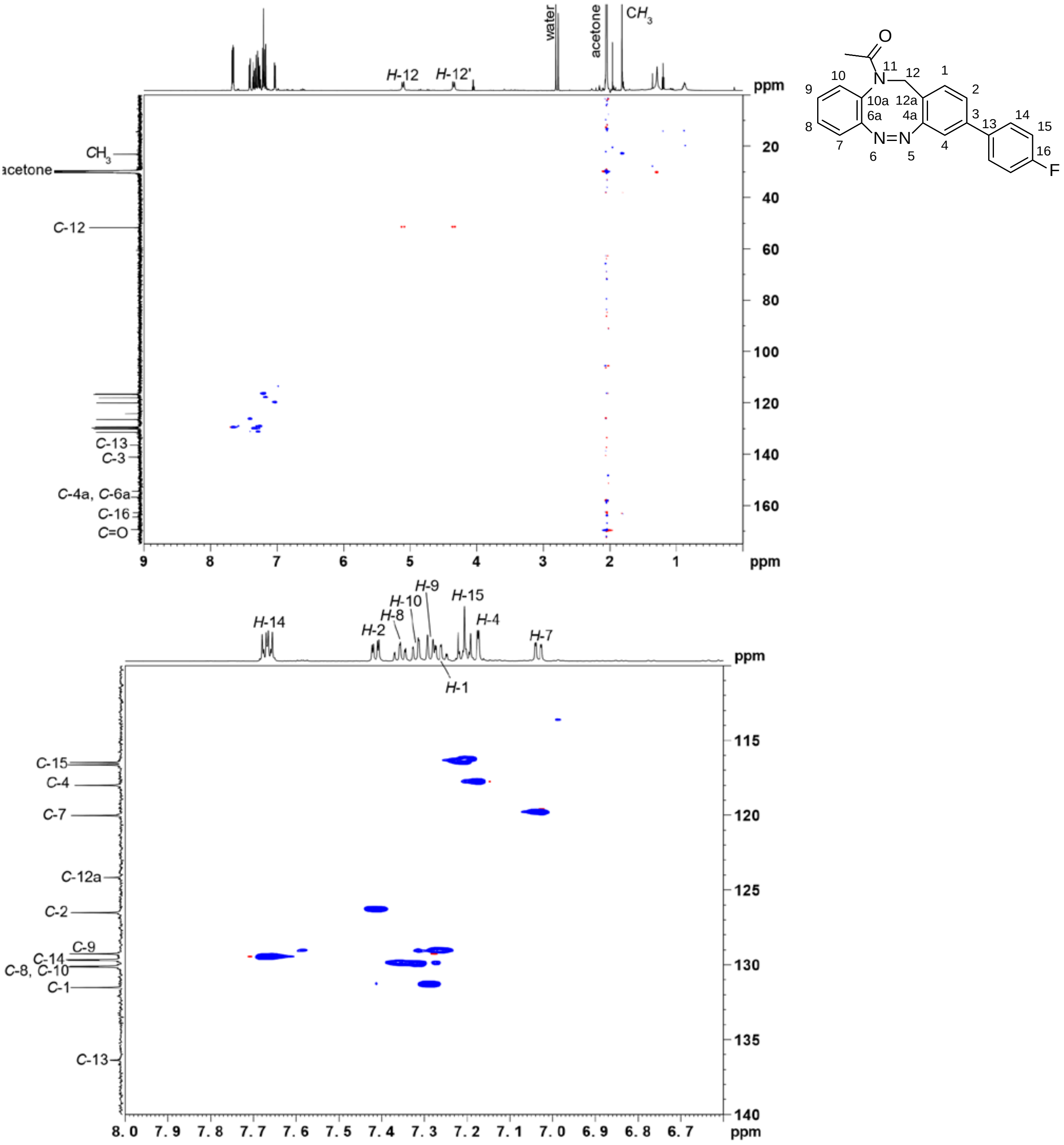


**Figure SII.65:** 600 MHz $^{1}$H-$^{13}$C HSQC spectrum of (*Z*)-1-(3-(4-fluorophenyl)dibenzo[*c*,*g*][1,2,5]triazocin-11(12*H*)-yl)ethan-1-one (**11**).

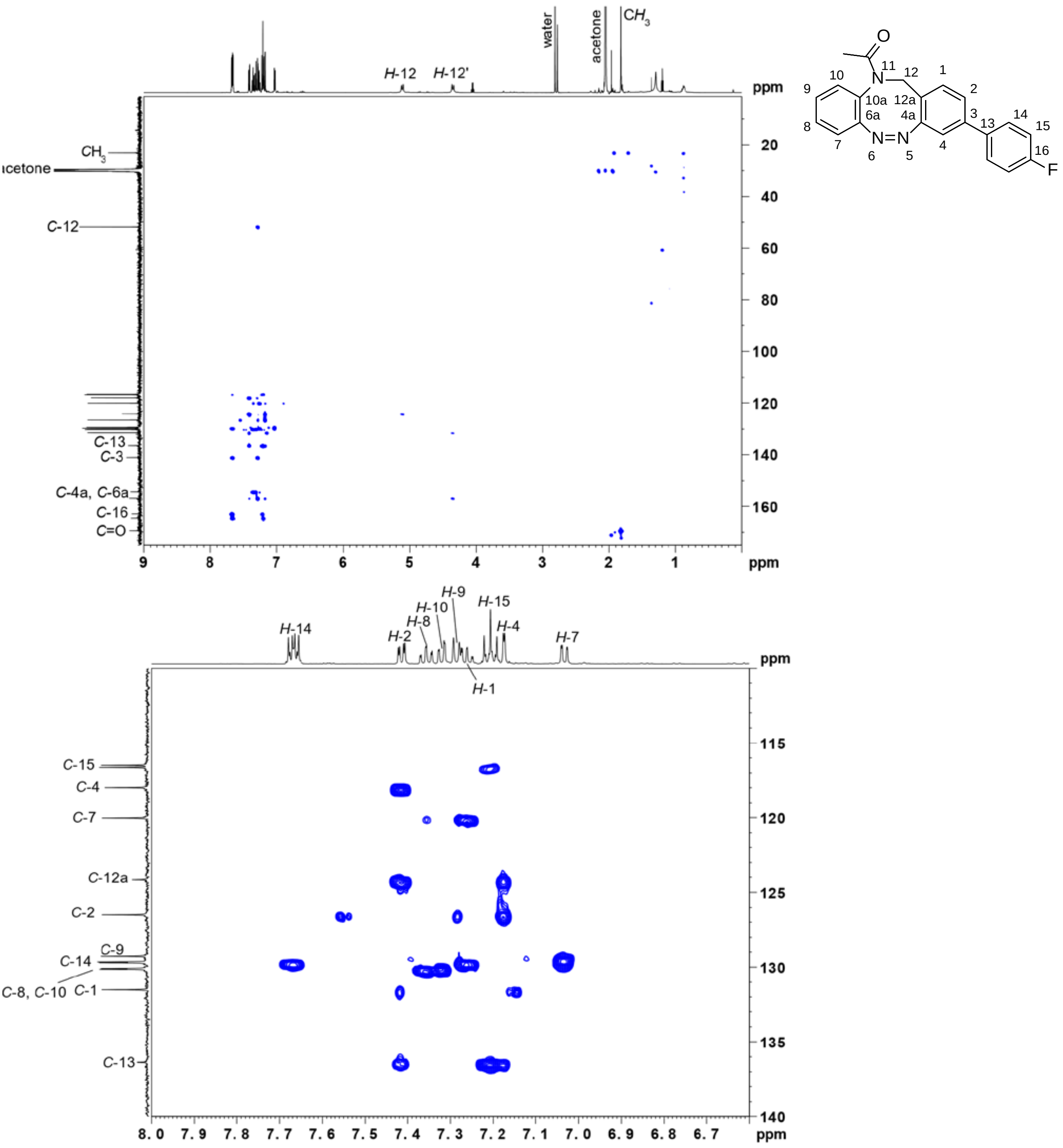


**Figure SII.66:** 600 MHz HMBC spectrum of (*Z*)-1-(3-(4-fluorophenyl)dibenzo[*c*,*g*][1,2,5]triazocin-11(12*H*)-yl)ethan-1-one (**11**).

## II.2.6 Synthesis of (*Z*)-2-(11-acetyl-11,12-dihydrodibenzo[*c,g*][1,2,5]triazocin-3-yl)benzaldehyde (12)

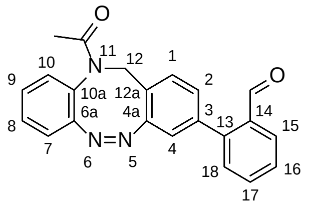


Halogenated *N*-acetyl-diazocine (**2**: 20.0 mg, 60.6 µmol, **3**: 23.0 mg, 60.6 µmol), (2-formylphenyl)boronic acid (18.1 mg, 121 µmol), $Pd(PPh_3)_4$ (2.45 mg, 2.12 µmol) and potassium carbonate (33.4 mg, 212 µmol) were dissolved in 2.4 mL of toluene/MeOH/deionized $H_2O$ (5 : 1 : 2) under a nitrogen atmosphere, heated to 85 °C with an oil bath and stirred for 16 h at given temperature. After cooling to rt. the reaction mixture was extracted with 10 mL of DCM twice, dried over $MgSO_4$ and the solvent was evaporated. Column chromatography on silica (ethyl acetate/cyclohexane 1:1, $R_f$ = 0.26) gave the product as yellow solid (from bromide **2**: 15.1 mg, 42.4 µmol, 70%; from iodide **3**: 16.6 mg, 46.7 µmol, 77%).

**melting point:** 160 °C

**$^1$H-NMR** (600 MHz, acetone-$d_6$, 298 K): $\delta$ = 9.71 (s, $^3J$ = 5.3 Hz, 1 H, C*H*O), 7.93 (dd, $^3J$ = 7.8 Hz, $^4J$ = 1.9 Hz, 1 H, *H*-15), 7.71 (td, $^3J$ = 7.6 Hz, $^4J$ = 1.4 Hz, 1 H, *H*-17), 7.58 (td, $^3J$ = 7.7 Hz, $^4J$ = 2.0 Hz, 1 H, *H*-16), 7.44-7.38 (m, 2 H, *H*-8, *H*-18), 7.37-7.33 (m, 2 H, *H*-1, *H*-10), 7.29 (td, $^3J$ = 7.3 Hz, $^4J$ = 1.5 Hz, 1 H, *H*-9), 7.22 (dd, $^3J$ = 7.8 Hz, $^4J$ = 1.9 Hz, 1 H, *H*-2), 7.05 (dd, $^3J$ = 7.8 Hz, $^3J$ = 1.5 Hz, 1 H, *H*-7), 6.99 (d, $^4J$ = 1.7 Hz, 1 H, *H*-4), 5.14 (d, $^2J$ = 14.6 Hz, 1 H, *H*-12), 4.41 (d, $^2J$ = 14.7 Hz, 1 H, *H*-12'), 1.83 (s, 3 H, -C*H*$_3$) ppm.

**$^{13}$C{$^1$H}-NMR** (150 MHz, acetone-$d_6$, 298 K): $\delta$ = 191.4 (*C*HO), 169.4 (*C*=O), 156.3 (*C*-4a), 154.4 (*C*-6a), 144.7 (*C*-13), 139.29 (*C*-3), 134.62 (*C*-14), 134.57 (*C*-17), 131.6 (*C*-18), 131.1 (*C*-1), 130.26 (*C*-10), 130.10 (*C*-8), 129.85 (*C*-2), 129.66 (*C*-10a), 129.39 (*C*-9), 129.26 (*C*-16), 128.34 (*C*-15), 125.1 (*C*-12a), 121.2 (*C*-4), 119.9 (*C*-7), 51.8 (*C*-12), 23.1 (*C*H$_3$) ppm.

**IR** (ATR): $\tilde{\nu}$ = 3751 (w), 3038 (w), 2924 (m), 2855 (w), 2753 (w), 2255 (w), 1688 (s), 1661 (s), 1600 (m), 1511 (w), 1474 (m), 1456 (w), 1430 (w), 1381 (s), 1337 (s), 1295 (w), 1253 (m), 1194 (m), 1163 (w), 1107 (m), 1074 (w), 1042 (w), 1005 (w), 928 (w), 839 (m), 768 (s), 741 (m), 630 (m), 576 (m), 439 (m) $cm^{-1}$.

**HR-MS** (ESI, acetone): *m/z* $[M+H]^+$ calculated for $C_{22}H_{18}O_2N_3+H^+$: 356.1394 found: 356.1387 ± 1.76 ppm.

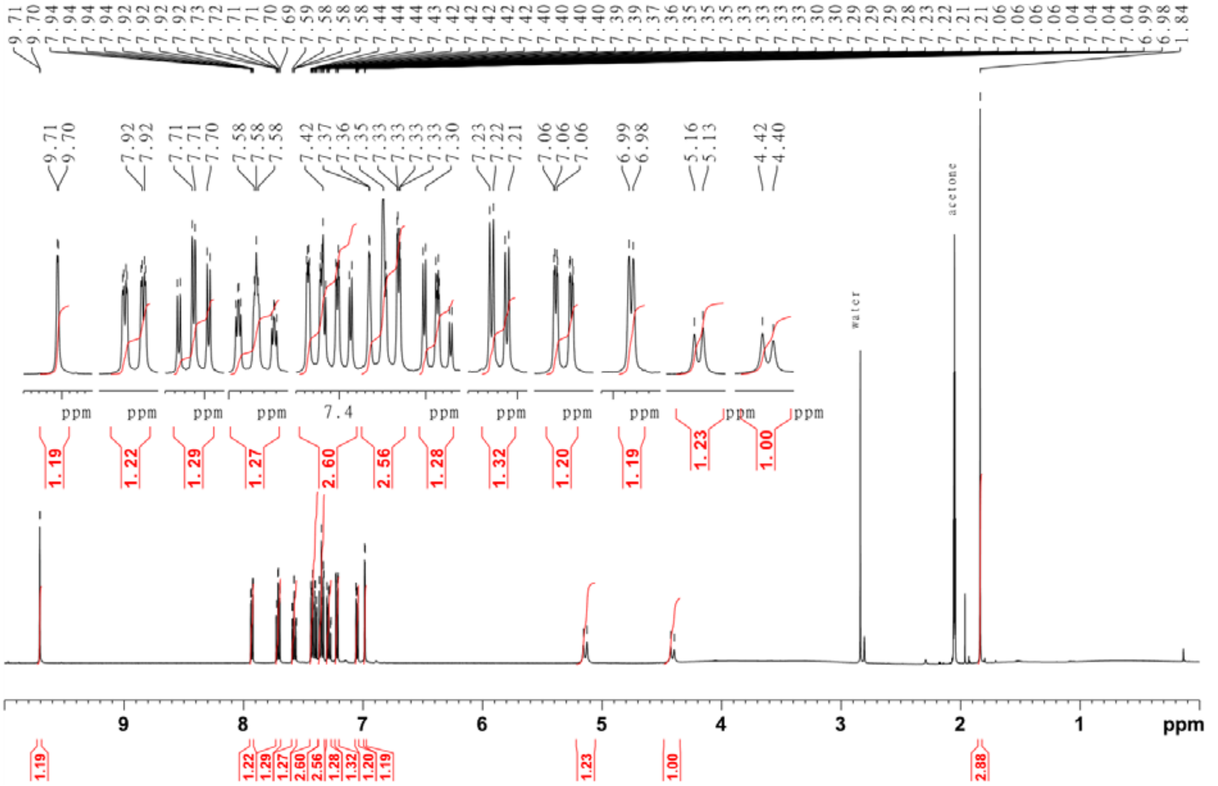

**Figure SII.67:** 600 MHz $^{1}$H-NMR spectrum of (*Z*)-2-(11-acetyl-11,12-dihydrodibenzo[*c*,*g*][1,2,5]triazocin-3-yl)benzaldehyde (**12**).

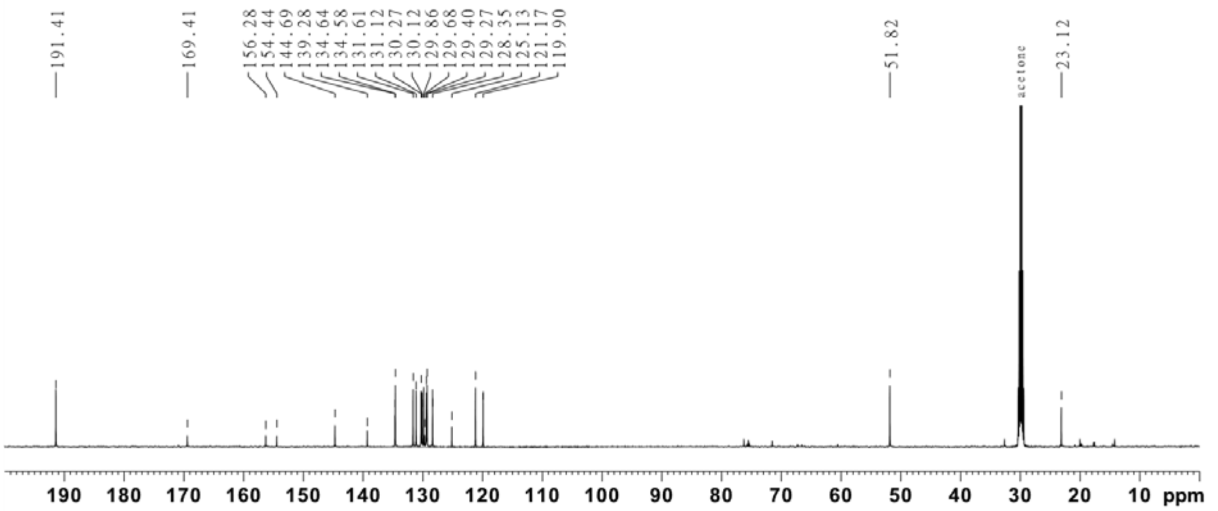

**Figure SII.68:** 150 MHz $^{13}C\{^{1}H\}$-NMR spectrum of (*Z*)-2-(11-acetyl-11,12-dihydrodibenzo[*c*,*g*][1,2,5]triazocin-3-yl)benzaldehyde (**12**).

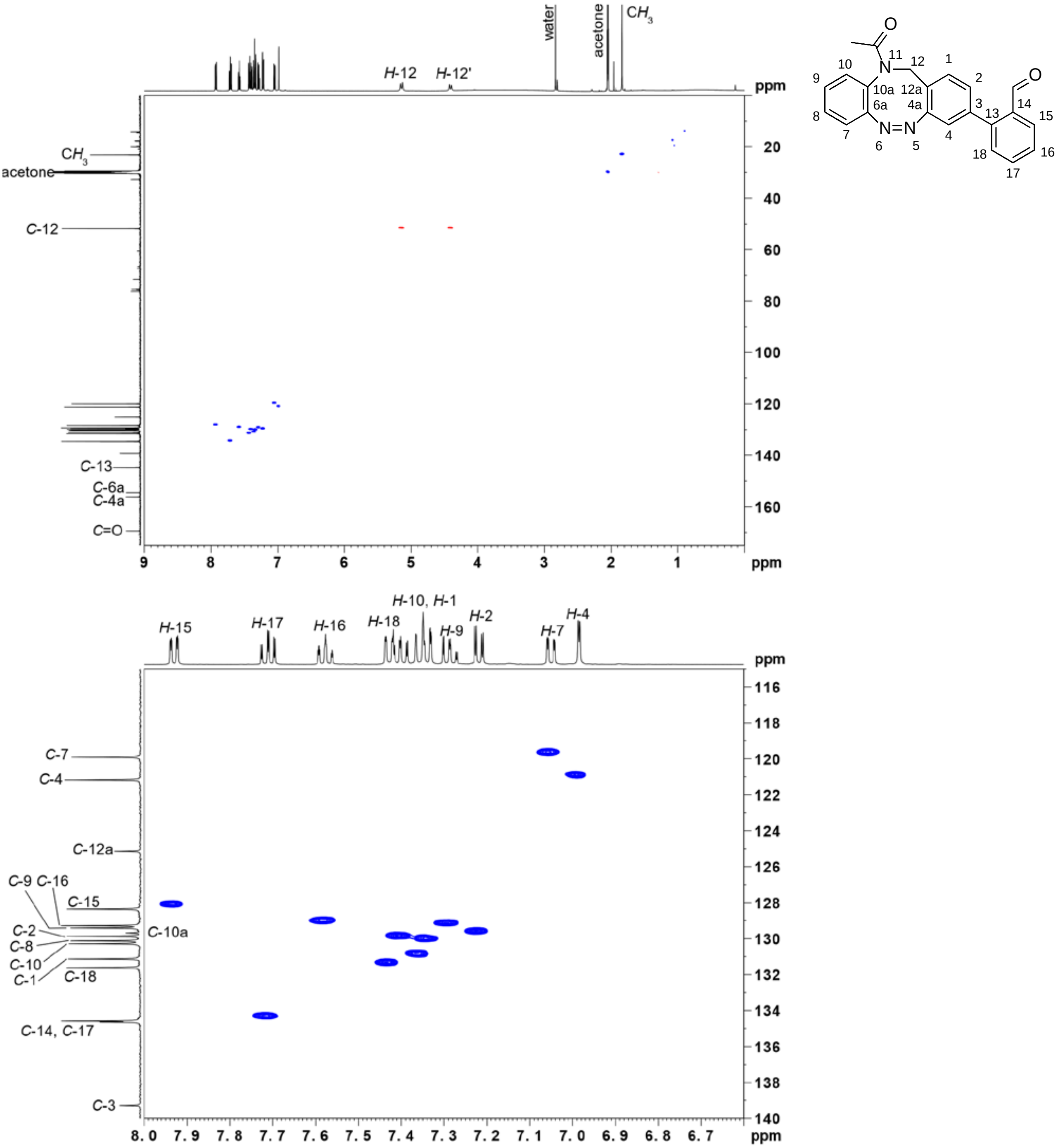


**Figure SII.69:** 600 MHz $^{1}$H-$^{13}$C HSQC spectrum of (*Z*)-2-(11-acetyl-11,12-dihydrodibenzo[*c*,*g*][1,2,5]triazocin-3-yl)benzaldehyde (**12**).

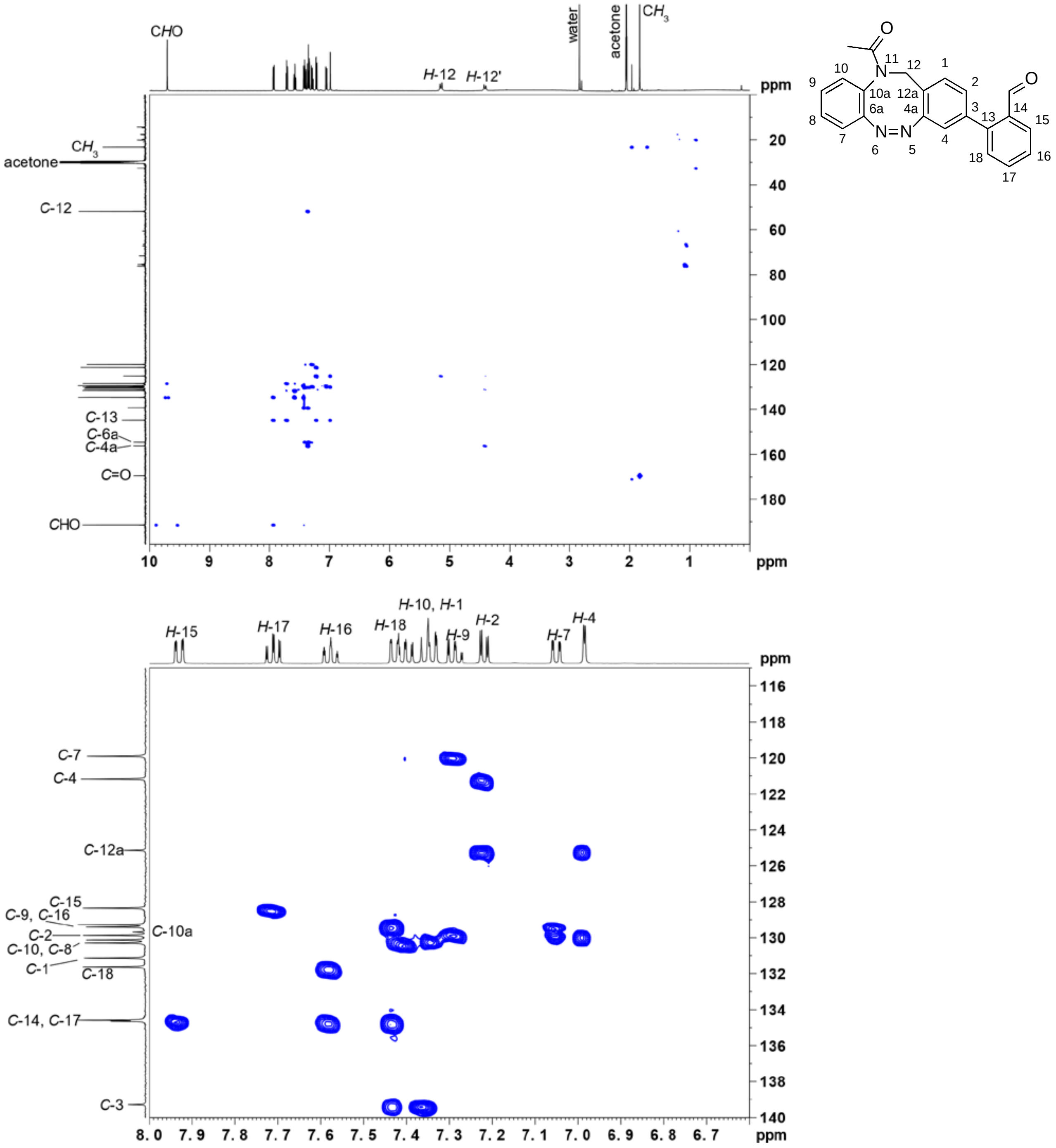


**Figure SII.70:** 600 MHz HMBC spectrum of (*Z*)-2-(11-acetyl-11,12-dihydrodibenzo[*c*,*g*][1,2,5]triazocin-3-yl)benzaldehyde (**12**).

## II.2.7 Synthesis of (*Z*)-2-(11-acetyl-11,12-dihydrodibenzo[*c,g*][1,2,5]triazocin-3-yl)benzoic acid (13)

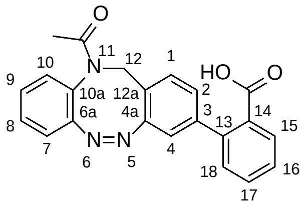


Halogenated *N*-acetyl-diazocine (**2**: 20.0 mg, 60.6 µmol, **3**: 23.0 mg, 60.6 µmol), 2-boronobenzoic acid (20.1 mg, 121 µmol), $Pd(PPh_3)_4$ (2.45 mg, 2.12 µmol) and potassium carbonate (33.4 mg, 212 µmol) were dissolved in 2.4 mL of dry DMF under a nitrogen atmosphere, heated to 100 °C with an oil bath and stirred for 16 h at given temperature. After cooling to rt. the reaction mixture was extracted with 10 mL of DCM twice, dried over $MgSO_4$ and the solvent was evaporated. Column chromatography on silica (ethyl acetate/cyclohexane 1:1 + 1% AcOH, $R_f$ = 0.33) gave the product as yellow solid (from bromide **2**: 17.1 mg, 46.1 µmol, 76%; from iodide **3**: 17.6 mg, 47.3 µmol, 79%).

**melting point:** 190 °C

**$^1$H-NMR** (600 MHz, acetone-$d_6$, 298 K): $\delta$ = 7.84 (dd, $^3J$ = 7.7 Hz, $^4J$ = 1.0 Hz, 1 H, *H*-15), 7.56 (td, $^3J$ = 7.6 Hz, $^4J$ = 1.4 Hz, 1 H, *H*-17), 7.47 (td, $^3J$ = 7.6 Hz, $^4J$ = 1.2 Hz, 1 H, *H*-16), 7.36 (td, $^3J$ = 7.7 Hz, $^4J$ = 1.3 Hz, 1 H, *H*-8), 7.31 (dd, $^3J$ = 7.9 Hz, $^4J$ = 1.1 Hz, 1 H, *H*-10), 7.29-7.24 (m, 2 H, *H*-9, *H*-18), 7.21 (d, $^3J$ = 7.9 Hz, 1 H, *H*-1), 7.12 (dd, $^3J$ = 7.8 Hz, $^4J$ = 1.2 Hz, 1 H, *H*-2), 7.02 (dd, $^3J$ = 7.8 Hz, $^3J$ = 1.2 Hz, 1 H, *H*-7), 6.89 (d, $^4J$ = 1.6 Hz, 1 H, *H*-4), 5.11 (d, $^2J$ = 14.5 Hz, 1 H, *H*-12), 4.33 (d, $^2J$ = 14.5 Hz, 1 H, *H*-12'), 1.82 (s, 3 H, -C$H_3$) ppm.

**$^{13}$C{$^1$H}-NMR** (150 MHz, acetone-$d_6$, 298 K): $\delta$ = 169.4 (*C*=O, COOH), 155.9 (*C*-4a), 154.4 (*C*-6a), 142.8 (*C*-3), 141.3 (*C*-13), 132.6 (*C*-14), 132.0 (*C*-17), 131.5 (*C*-18), 130.76 (*C*-15), 130.55 (*C*-1), 130.10 (*C*-10), 130.02 (*C*-8), 129.6 (*C*-10a), 129.2 (*C*-9), 128.6 (*C*-16), 128.5 (*C*-2), 123.9 (*C*-12a), 120.08 (*C*-7), 120.01 (*C*-4), 51.9 (*C*-12), 23.1 (*C*$H_3$) ppm.

**IR** (ATR): $\tilde{\nu}$ = 2921 (s), 2852 (m), 2022 (w), 1713 (m), 1613 (s), 1591 (w), 1516 (w), 1477 (s), 1448 (w), 1377 (s), 1235 (s), 1135 (m), 1078 (m), 1014 (w), 977 (w), 922 (w), 877 (w), 834 (m), 756 (s), 739 (w), 720 (w), 691 (w), 628 (m), 603 (w), 575 (m), 533 (w), 488 (w), 439 (m), 409 (m) $cm^{-1}$.

**HR-MS** (ESI, DCM): *m/z* $[M+H]^+$ calculated for $C_{22}H_{17}O_3N_3+H^+$: 372.1343 found: 372.1337 ± 1.64 ppm.

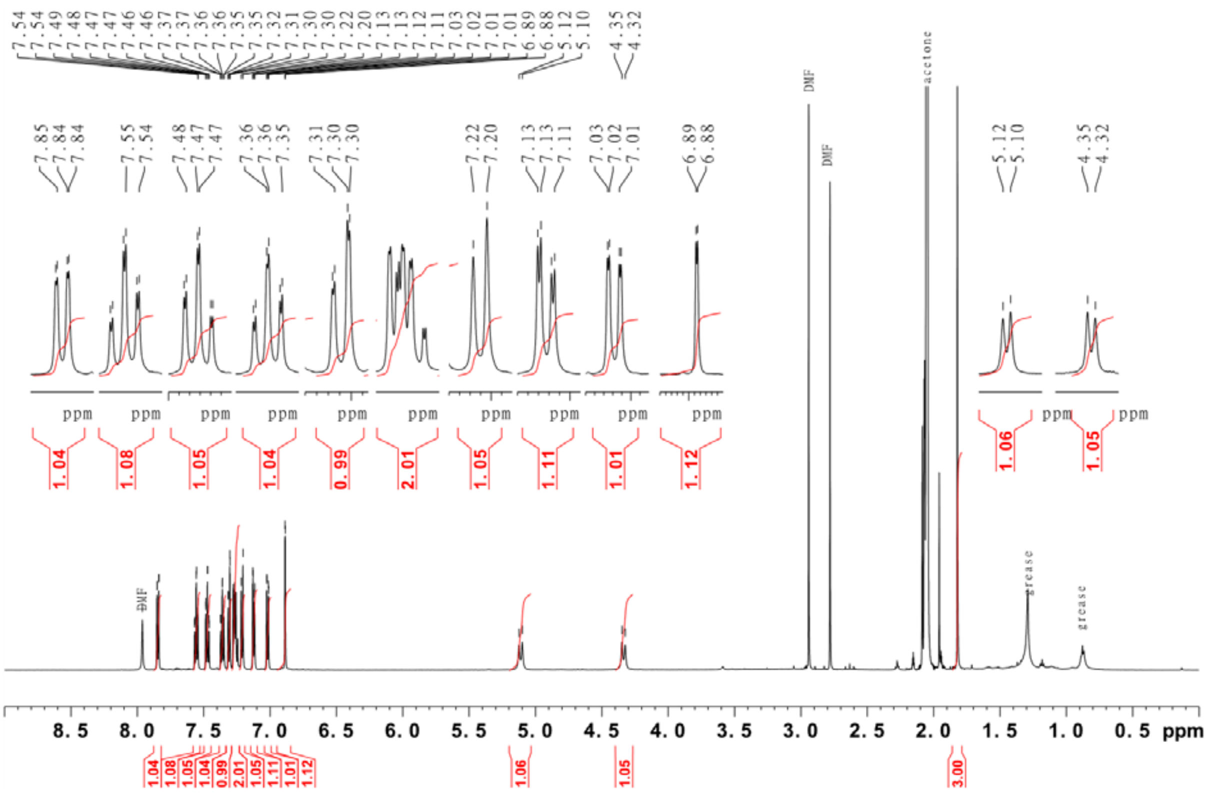

**Figure SII.71:** 600 MHz $^{1}$H-NMR spectrum of (*Z*)-2-(11-acetyl-11,12-dihydrodibenzo[*c*,*g*][1,2,5]triazocin-3-yl)benzoic acid (**13**).

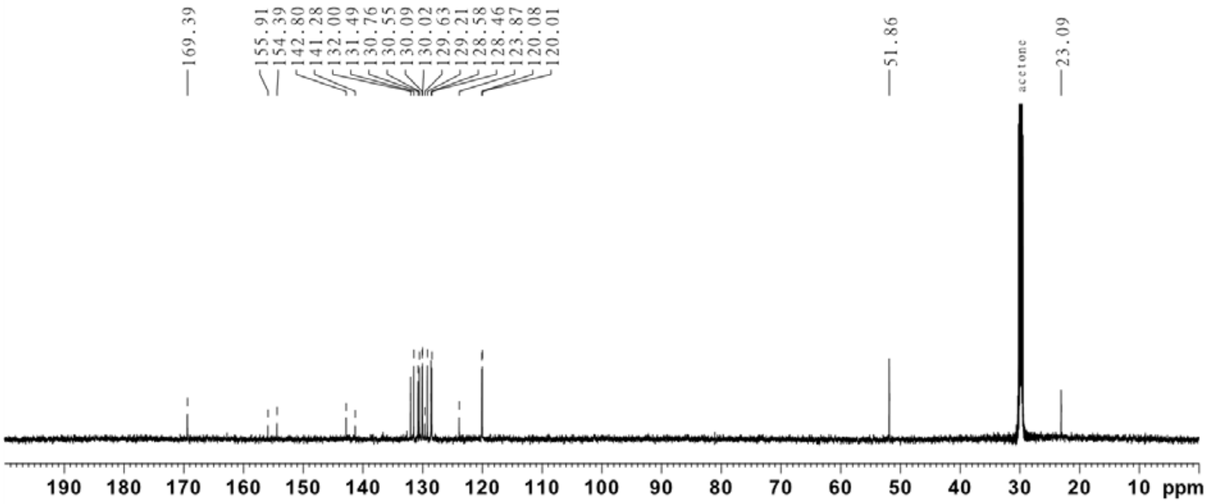

**Figure SII.72:** 150 MHz $^{13}$C{$^{1}$H}-NMR spectrum of (*Z*)-2-(11-acetyl-11,12-dihydrodibenzo[*c*,*g*][1,2,5]triazocin-3-yl)benzoic acid (**13**).

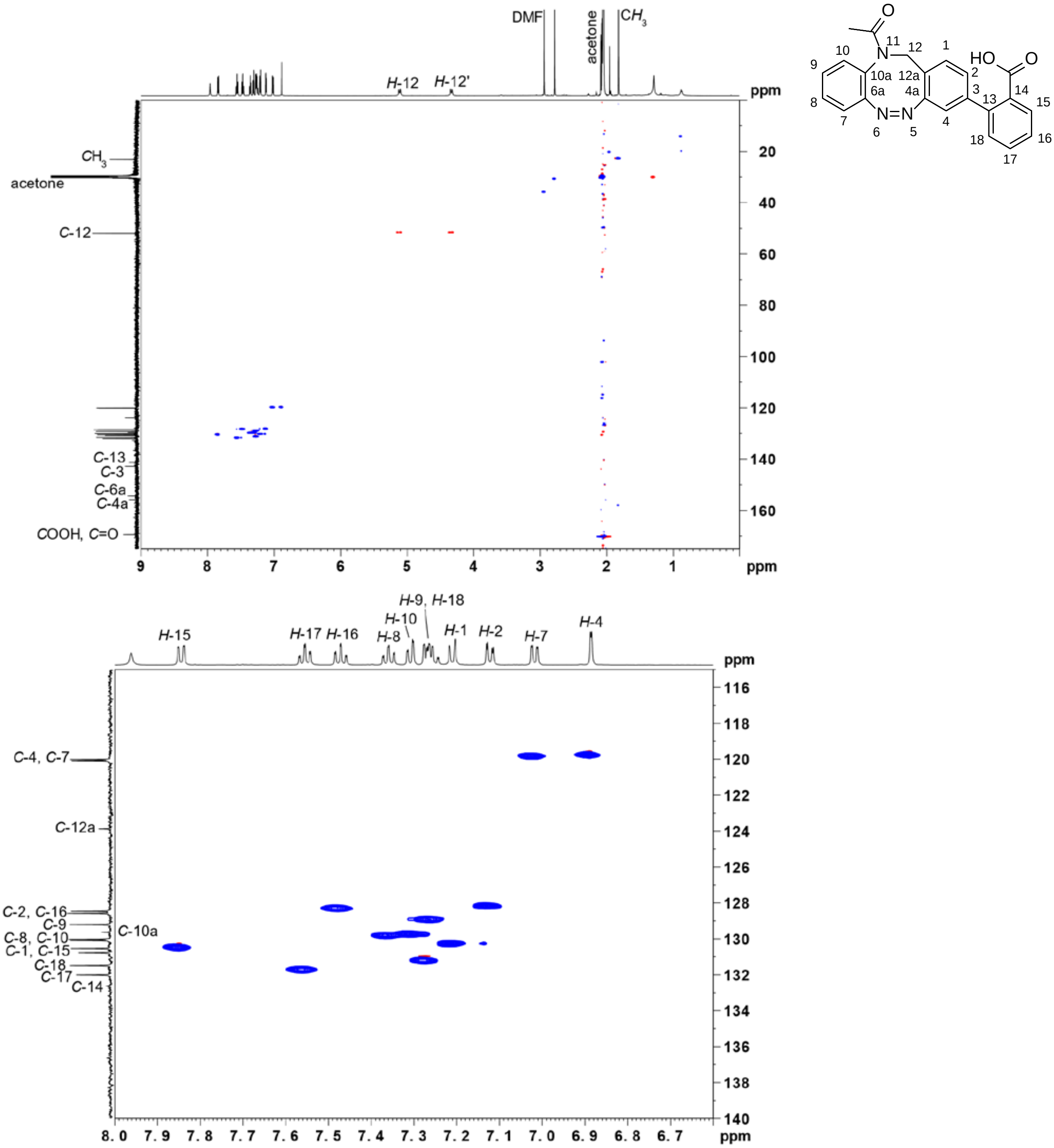


**Figure SII.73:** 600 MHz $^{1}$H-$^{13}$C HSQC spectrum of (*Z*)-2-(11-acetyl-11,12-dihydrodibenzo[*c*,*g*][1,2,5]triazocin-3-yl)benzoic acid (**13**).

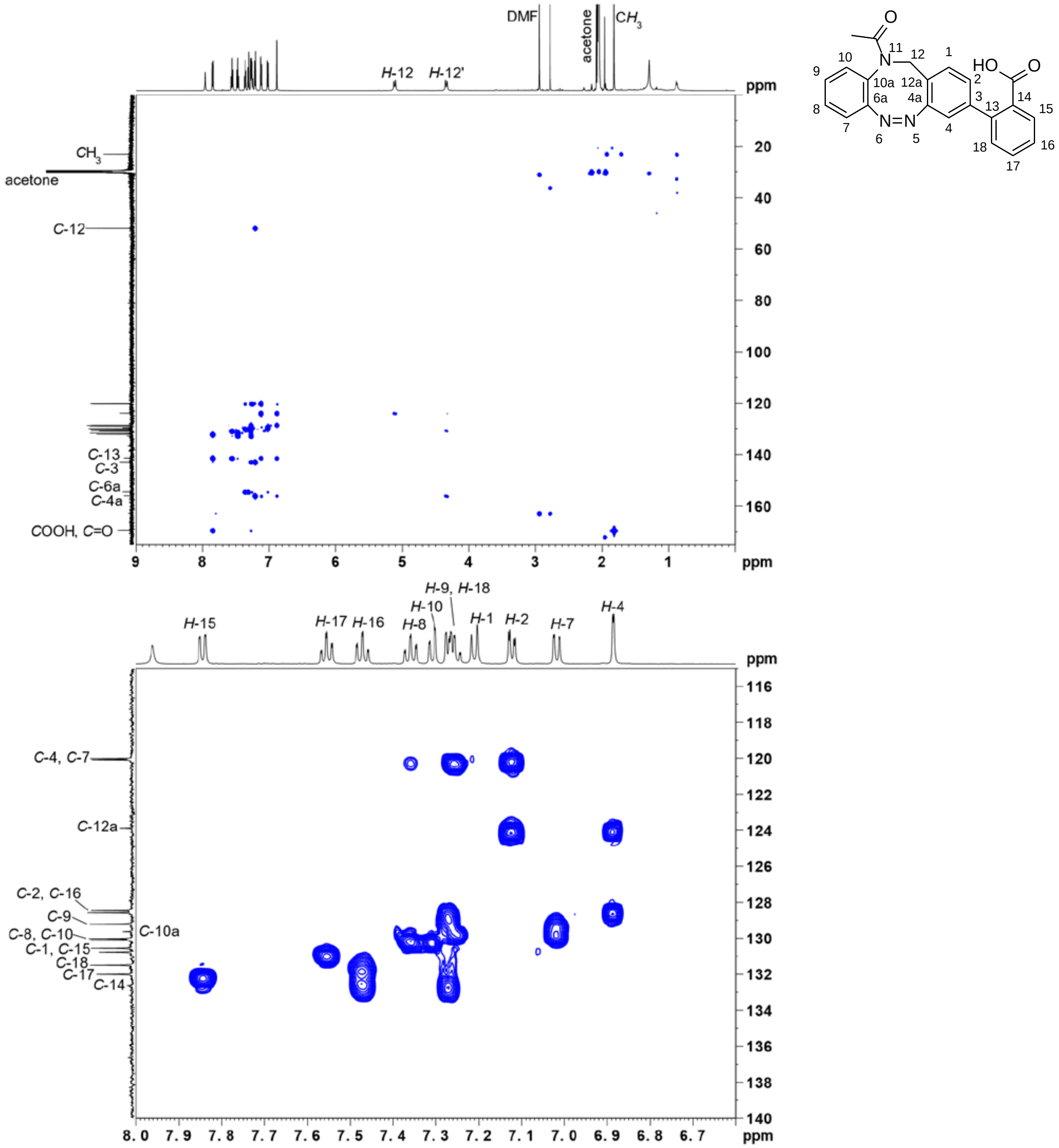


**Figure SII.74:** 600 MHz HMBC spectrum of (*Z*)-2-(11-acetyl-11,12-dihydrodibenzo[*c*,*g*][1,2,5]triazocin-3-yl)benzoic acid (**13**).

## II.2.8 Synthesis of (*Z*)-1-(3-(pyridine-4-yl)dibenzo[*c,g*][1,2,5]triazocin-11(12*H*)-ethan-1-one (14)

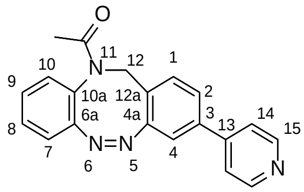

Halogenated *N*-acetyl-diazocine (**2**: 20.0 mg, 60.6 µmol, **3**: 23.0 mg, 60.6 µmol), pyridine-4-boronic acid (14.8 mg, 121 µmol), $Pd(PPh_3)_4$ (2.45 mg, 2.12 µmol) and potassium carbonate (33.4 mg, 212 µmol) were dissolved in 2.4 mL of toluene/MeOH/deionized $H_2O$ (5 : 1 : 2) under a nitrogen atmosphere, heated to 85 °C with an oil bath and stirred for 16 h at given temperature. After cooling to rt. the reaction mixture was extracted with 10 mL of DCM twice, dried over $MgSO_4$ and the solvent was evaporated. Column chromatography on silica (ethyl acetate/cyclohexane 1:1, $R_f$ = 0.07) gave the product as yellow solid (from bromide **2**: 1.4 mg, 4.26 µmol, 7%; from iodide **3**: 3.8 mg, 11.6 µmol, 19%).

**melting point:** 223 °C

**$^1$H-NMR** (600 MHz, acetone-$d_6$, 298 K): $\delta$ = 8.62 (dd, $^3J$ = 6.1 Hz, $^4J$ = 1.6 Hz, 2 H, *H*-15), 7.61 (dd, $^3J$ = 6.1 Hz, $^4J$ = 1.6 Hz, 2 H, *H*-14), 7.56 (dd, $^3J$ = 8.0 Hz, $^4J$ = 1.9 Hz, 1 H, *H*-2), 7.38-7.32 (4 H, *H*-1, *H*-4, *H*-7, *H*-9), 7.27 (dd, $^3J$ = 7.8 Hz, $^4J$ = 1.4 Hz, 1 H, *H*-8), 7.05 (dd, $^3J$ = 7.8 Hz, $^4J$ = 1.2 Hz, 1 H, *H*-10), 5.15 (d, $^2J$ = 14.6 Hz, 1 H, *H*-12), 4.38 (d, $^2J$ = 14.4 Hz, 1 H, *H*-12'), 1.81 (s, 3 H, -C*H*3) ppm.

**$^{13}$C{$^1$H}-NMR** (150 MHz, acetone-$d_6$, 298 K): $\delta$ = 169.4 (*C*=O), 156.8 (*C*-4a), 154.3 (*C*-6a), 151.4 (*C*-15), 146.7 (*C*-13), 139.2 (*C*-3), 131.8 (*C*-1), 130.21 (*C*-8/9), 130.16 (*C*-8/9), 129.57 (*C*-10a), 129.41 (*C*-7), 126.7 (*C*-2), 126.1 (*C*-12a), 122.0 (*C*-14), 120.1 (*C*-10), 118.3 (*C*-4), 51.8 (*C*-12), 23.0 (*C*H3) ppm.

**IR** (ATR): $\tilde{\nu}$ = 2922 (m), 2851 (w), 1726 (m), 1655 (s), 1599 (m), 1543 (w), 1513 (w), 1478 (m), 1451 (w), 1420 (w), 1379 (s), 1336 (s), 1291 (w), 1246 (w), 1074 (m), 1045 (w), 995 (w), 964 (w), 922 (w), 876 (m), 846 (w), 817 (s), 767 (m), 738 (m), 711 (m), 624 (s), 602 (w), 573 (m), 547 (m), 512 (m) $cm^{-1}$.

**HR-MS** (ESI, DCM): *m/z* $[M+H]^+$ calculated for $C_{20}H_{16}ON_4+H^+$: 329.1397; found: 329.1393 ± 1.07 ppm.

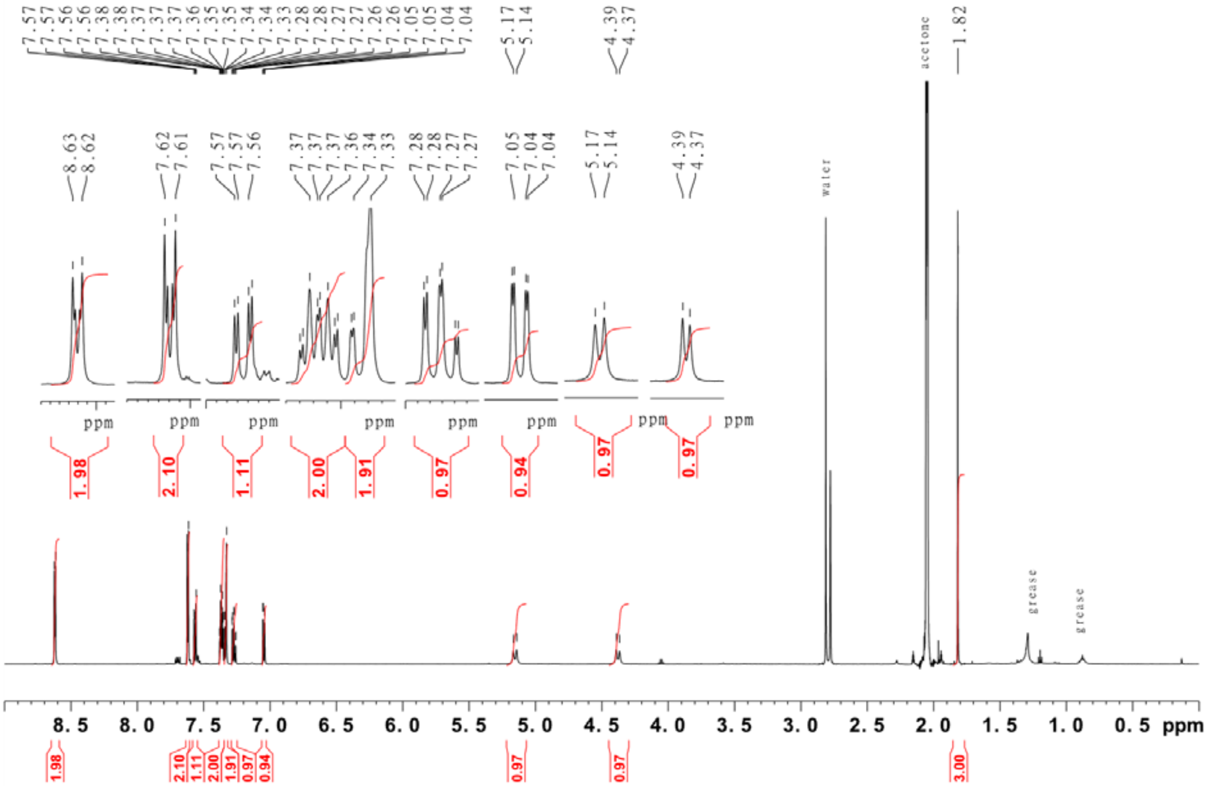

**Figure SII.75:** 600 MHz $^{1}$H-NMR spectrum of (*Z*)-1-(3-(pyridine-4-yl)dibenzo[*c,g*][1,2,5]triazocin-11(12*H*)-ethan-1-one (**14**).

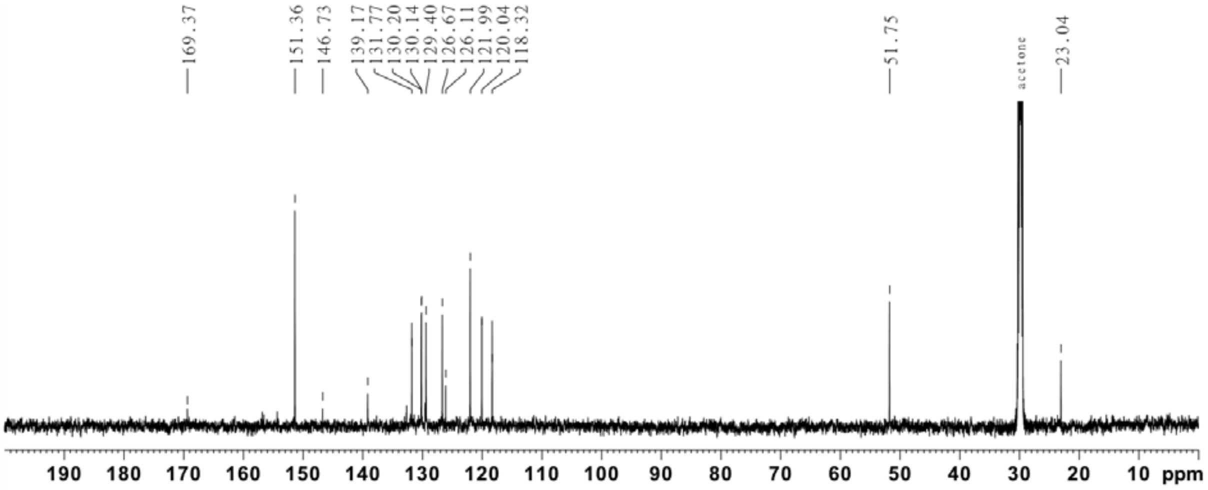

**Figure SII.76:** 150 MHz $^{13}$C{$^{1}$H}-NMR spectrum of (*Z*)-1-(3-(pyridine-4-yl)dibenzo[*c,g*][1,2,5]triazocin-11(12*H*)-ethan-1-one (**14**).

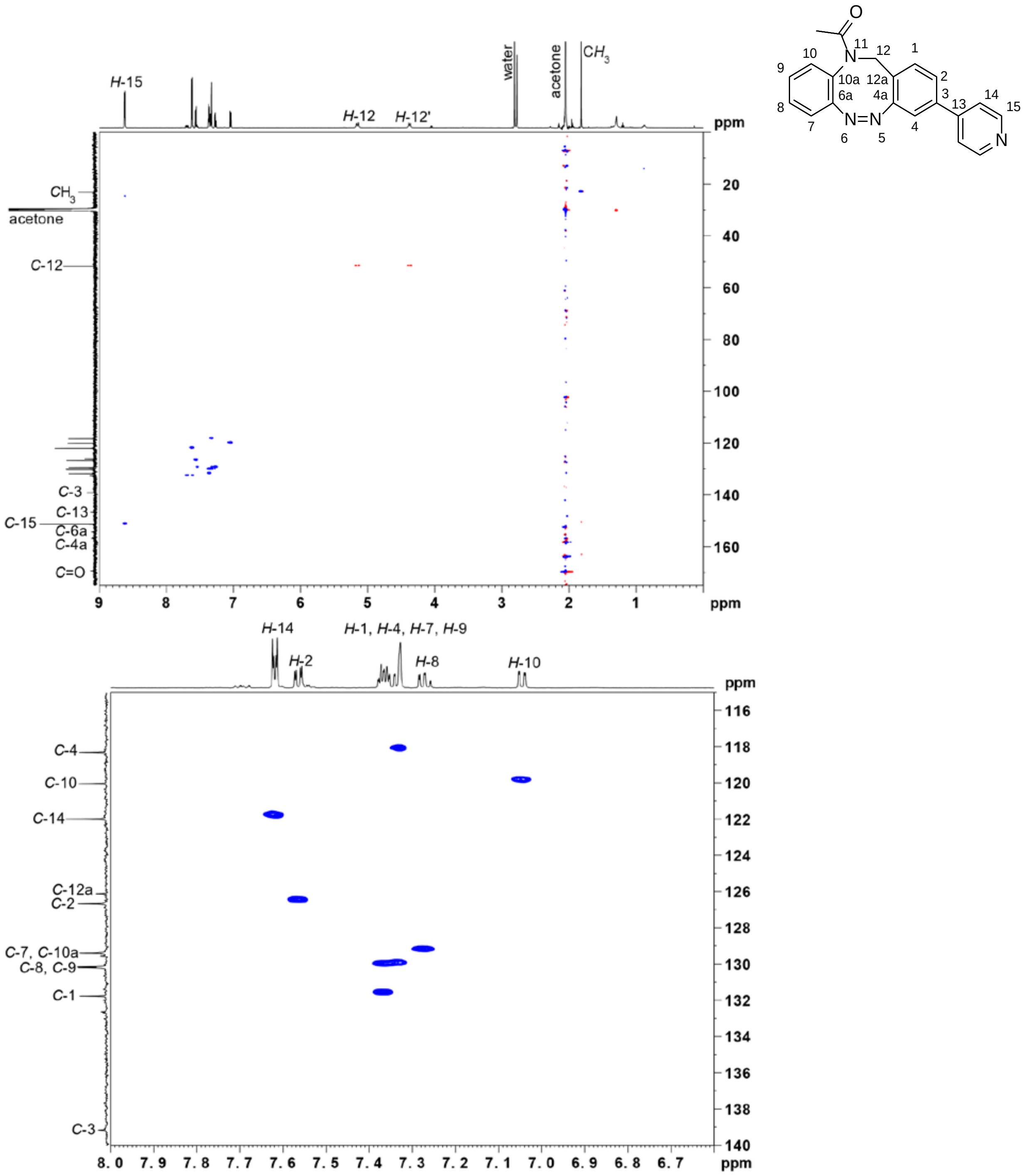


**Figure SII.77:** 600 MHz $^{1}H$-$^{13}C$ HSQC spectrum of (*Z*)-1-(3-(pyridine-4-yl)dibenzo[*c*,*g*][1,2,5]triazocin-11(12*H*)-ethan-1-one (**14**).

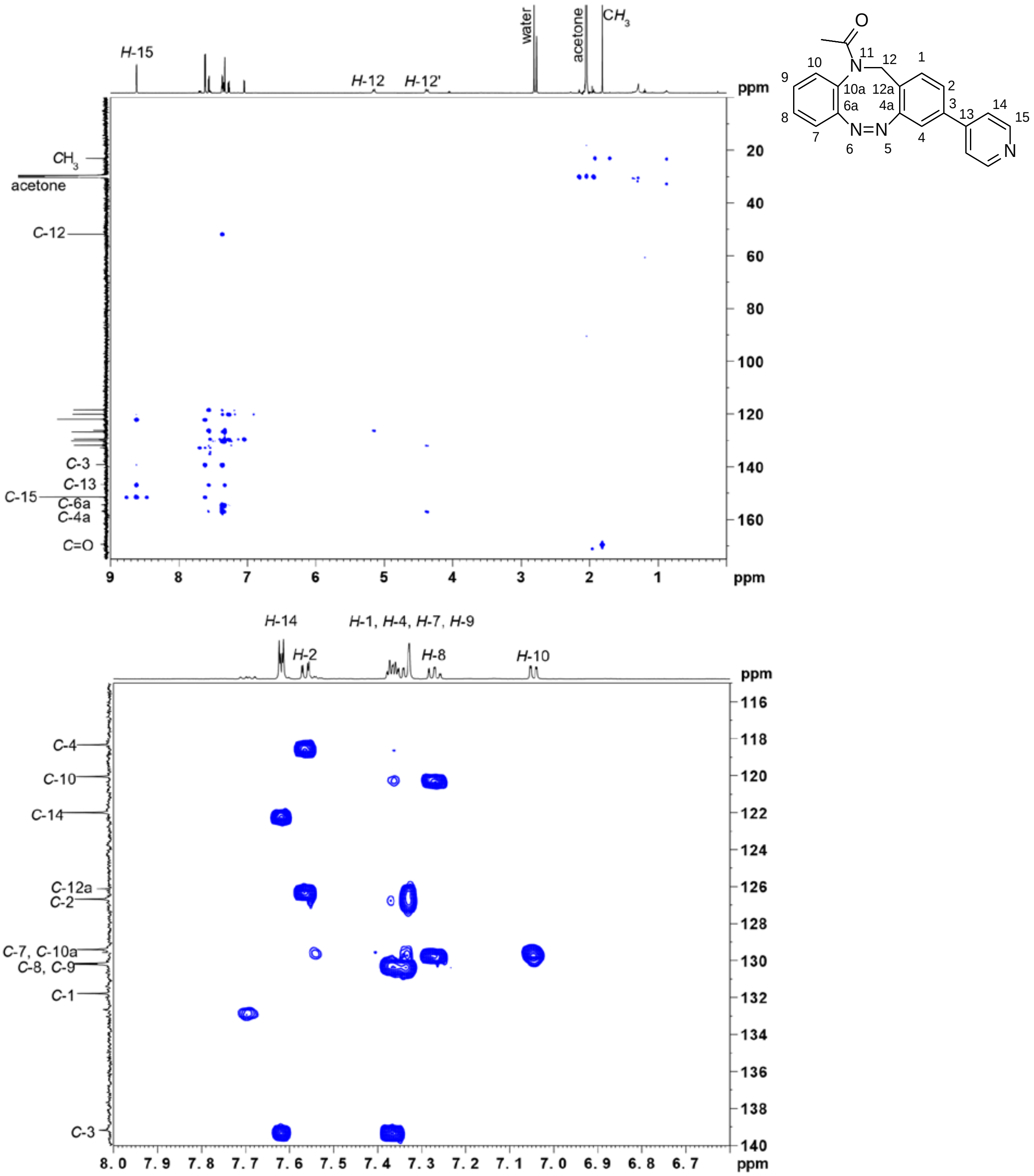


**Figure SII.78:** 600 MHz HMBC spectrum of (*Z*)-1-(3-(pyridine-4-yl)dibenzo[*c*,*g*][1,2,5]triazocin-11(12*H*)-ethan-1-one (**14**).

## II.2.9 Synthesis of (*Z*)-1-(3-benzyldibenzo[*c,g*][1,2,5]triazocin-11(12*H*)-ethan-1-one (17)

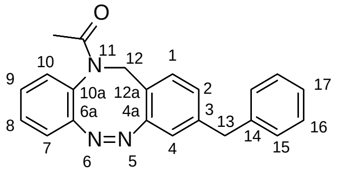


Halogenated *N*-acetyl-diazocine (**2**, 20.0 mg, 60.6 µmol, **3**: 23.0 mg, 60.6 µmol) 2-benzyl-4,4,5,5-tetramethyl-1,3,2-dioxoborolan (26.4 mg, 121 µmol), $Pd(PPh_3)_4$ (2.45 mg, 2.12 µmol) and potassium carbonate (33.4 mg, 212 µmol) were dissolved in 2.4 mL of toluene/MeOH/deionized $H_2O$ (5 : 1 : 2) under a nitrogen atmosphere, heated to 85 °C with an oil bath and stirred for 16 h at given temperature. After cooling to rt. the reaction mixture was extracted with 10 mL of DCM twice, dried over $MgSO_4$ and the solvent was evaporated. Column chromatography on silica (ethyl acetate/cyclohexane 1:1, $R_f$ = 0.33) gave the product as yellow solid (from bromide **2**: 9.4 mg, 27.5 µmol, 45%; from iodide **3**: no product could be obtained).

**melting point:** 147 °C

**$^1$H-NMR** (600 MHz, $CDCl_3$, 298 K): $\delta$ = 7.29-7.23 (m, 3 H, *H*-10, *H*-16), 7.19 (t, $^3J$ = 7.1 Hz, 1 H, *H*-17), 7.15 (td, $^3J$ = 7.7 Hz, $^4J$ = 1.5 Hz, 1 H, *H*-8), 7.04-7.00 (m, 3 H, *H*-9, *H*-15), 6.95 (d, $^3J$ = 8.1 Hz, 1 H, *H*-1), 6.88 (dd, $^3J$ = 7.9 Hz, $^4J$ = 1.4 Hz, 1 H, *H*-7), 6.86 (dd, $^3J$ = 8.0 Hz, $^4J$ = 1.7 Hz, 1 H, *H*-2), 6.68 (d, $^4J$ = 1.3 Hz, 1 H, *H*-4), 5.02 (d, $^2J$ = 14.6 Hz, 1 H, *H*-12), 4.31 (d, $^2J$ = 14.4 Hz, 1 H, *H*-12'), 3.88 (s, 2 H, *H*-13), 1.91 (s, 3 H, -C$H_3$) ppm.

**$^{13}$C{$^1$H}-NMR** (150 MHz, $CDCl_3$, 298 K): $\delta$ = 170.2 (*C*=O), 155.6 (*C*-4a), 153.2 (*C*-6a), 142.3 (*C*-3), 140.0 (*C*-14), 130.2 (*C*-1), 129.05 (*C*-10/16), 128.96 (*C*-15), 128.91 (*C*-9), 128.69 (*C*-10/16), 128.3 (*C*-2), 128.14 (*C*-8), 126.5 (*C*-17), 121.6 (*C*-12a), 119.57 (*C*-4), 119.46 (*C*-7), 51.8 (*C*-12), 41.4 (*C*-13) 23.4 ($CH_3$) ppm.

**IR** (ATR): $\tilde{\nu}$ = 2924 (m), 2853 (w), 2256 (m), 1654 (s), 1611 (w), 1593 (w), 1513 (w), 1482 (m), 1453 (m), 1437 (w), 1386 (s), 1337 (s), 1286 (w), 1245 (w), 1200 (m), 1147 (w), 1074 (m), 1040 (m), 974 (w), 914 (m), 902 (w), 772 (s), 739 (m), 723 (s), 703 (m), 644 (w), 623 (w), 607 (s), 591 (s), 462 (m) $cm^{-1}$.

**HR-MS** (ESI, EtOAc): *m/z* $[M+Na]^+$ calculated for $C_{22}H_{19}ON_3+Na^+$: 364.1420; found: 364.1418 ± 0.75 ppm.

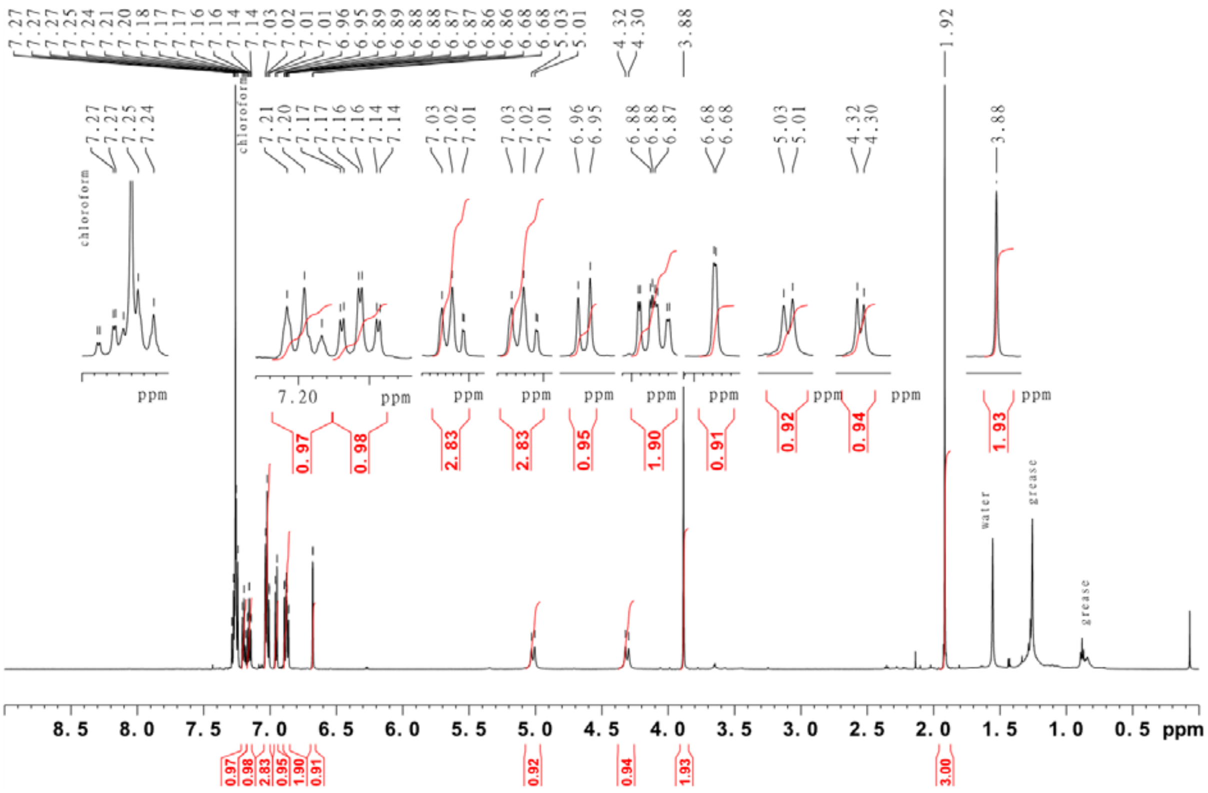


**Figure SII.79:** 600 MHz $^{1}H$-NMR spectrum of (*Z*)-1-(3-benzyldibenzo[*c*,*g*][1,2,5]triazocin-11(12*H*)-ethan-1-one (**17**).

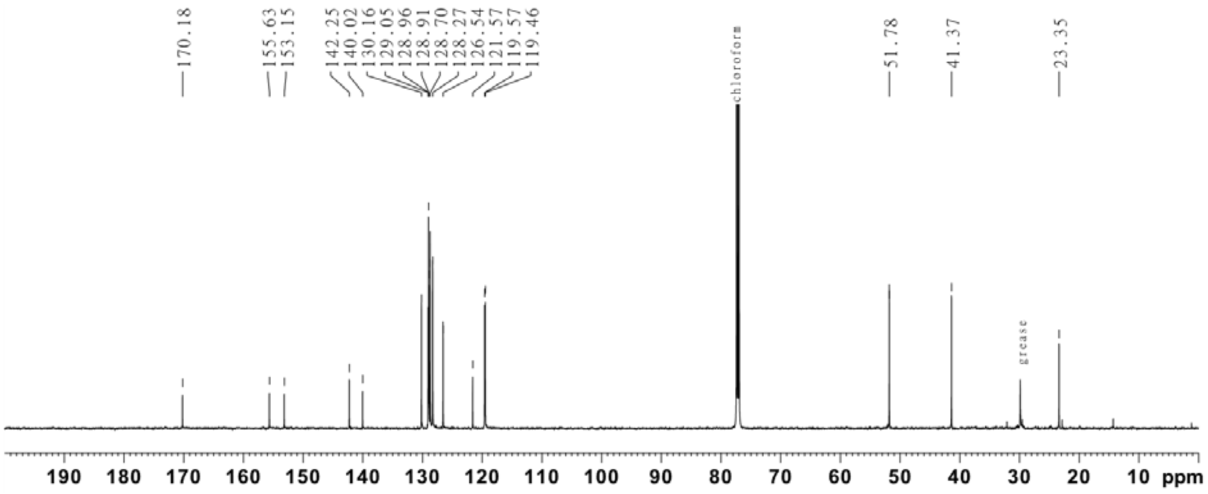


**Figure SII.80:** 150 MHz $^{13}C\{^{1}H\}$-NMR spectrum of (*Z*)-1-(3-benzyldibenzo[*c*,*g*][1,2,5]triazocin-11(12*H*)-ethan-1-one (**17**).

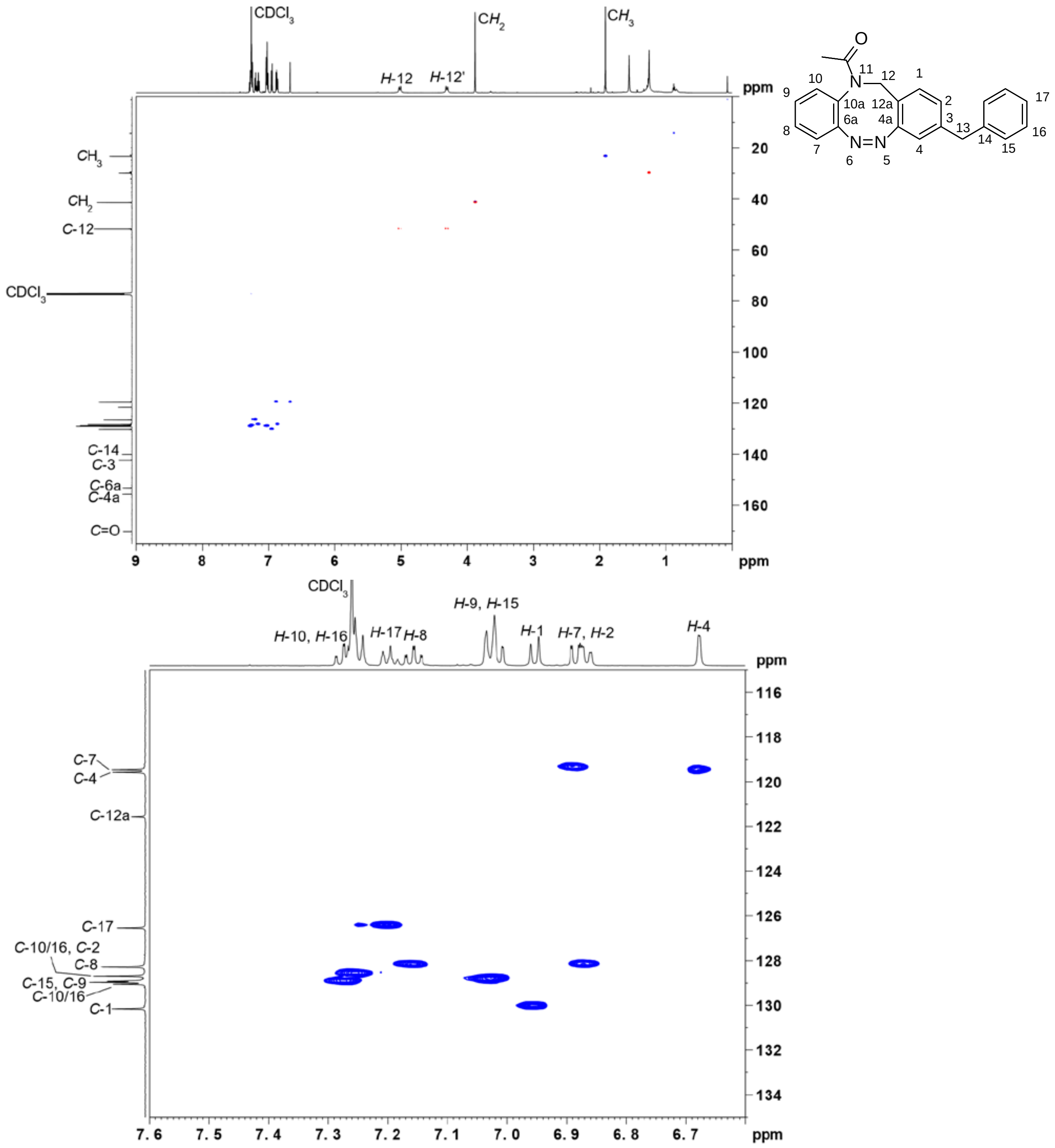


**Figure SII.81:** 600 MHz $^{1}$H-$^{13}$C HSQC spectrum of (*Z*)-1-(3-benzyldibenzo[*c,g*][1,2,5]triazocin-11(12*H*)-ethan-1-one (**17**).

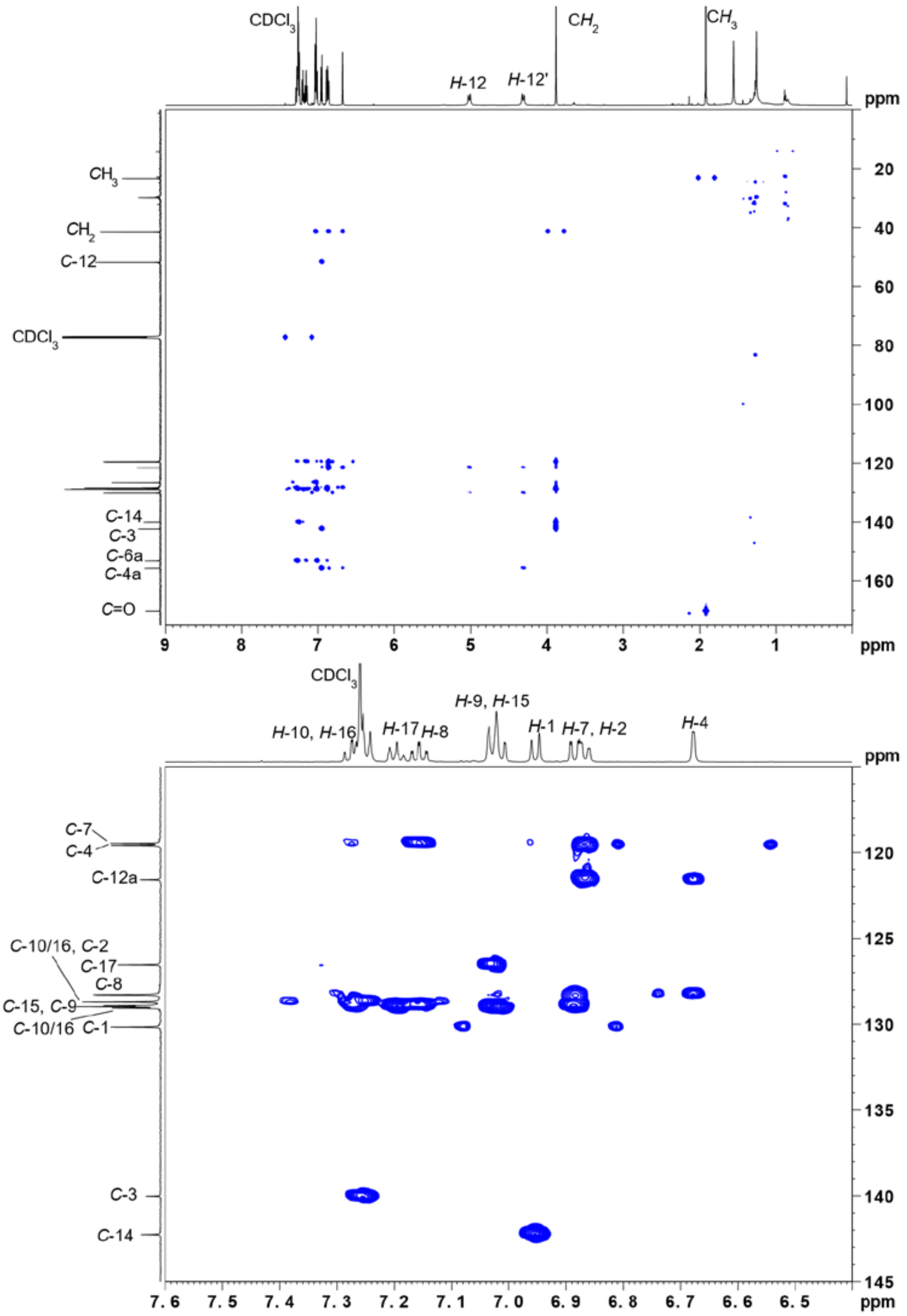


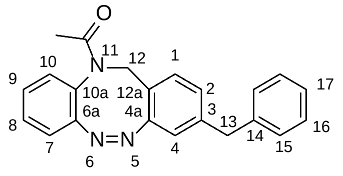

**Figure SII.82:** 600 MHz HMBC spectrum of (*Z*)-1-(3-benzyldibenzo[*c*,*g*][1,2,5]triazocin-11(12*H*)-ethan-1-one (**17**).

## II.2.11 Synthesis of *tert*-butyl (*Z*)-(11-acetyl-11,12-dihydrodiebenzo[*c,g*][1,2,5]triazocin-3-yl)carbamate (19)

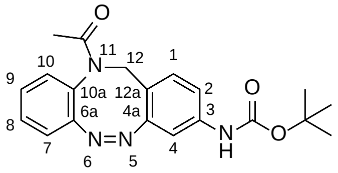


In a manner analogous to [2] halogenated *N*-acetyl-diazocine (**2**, 20.0 mg, 60.6 µmol, **3**: 23.0 mg, 60.6 µmol), *tert*-butyl carbamate (10.6 mg, 90.9 µmol), Xantphos Pd G3 (2.90 mg, 3.03 µmol) and caesium carbonate (19.7 mg, 60.6 µmol) were dissolved in 2.4 mL of dry 1,4-dioxane under a nitrogen atmosphere, heated to 100 °C with an oil bath and stirred for 16 h at given temperature. After cooling to rt. deionized $H_2O$ and 5 mL ethyl acetate were added and the reaction mixture was extracted with 10 mL of ethyl acetate twice, dried over $MgSO_4$ and the solvent was evaporated. Column chromatography on silica (ethyl acetate/cyclohexane 1:1, $R_f$ = 0.30) gave the product as yellow solid (from bromide **2**: no product could be obtained; from iodide **3**: 15.8 mg, 43.1 µmol, 72%).

**melting point:** 217°C

**$^1$H-NMR** (600 MHz, acetone-$d_6$, 298 K): $\delta$ = 8.56 (s, 1 H, -N*H*), 7.36 (td, $^3J$ = 7.6 Hz, $^4J$ = 1.7 Hz, 1 H, *H*-8), 7.28-7.20 (m, 4 H, *H*-2, *H*-4, *H*-9, *H*-10), 7.08 (d, $^3J$ = 8.2 Hz, 1 H, *H*-1), 7.00 (dd, $^3J$ = 7.7 Hz, $^4J$ = 1.3 Hz, 1 H, *H*-7), 4.93 (d, $^2J$ = 14.4 Hz, 1 H, *H*-12), 4.25 (d, $^2J$ = 14.5 Hz, 1 H, *H*-12'), 1.80 (s, 3 H, -C$H_3$), 1.45 (s, 9 H, -C(C$H_3$)$_3$) ppm.

**$^{13}$C{$^1$H}-NMR** (150 MHz, acetone-$d_6$, 298 K): $\delta$ = 170.9 (-NH*C*=O), 169.3 (*C*=O), 156.8 (*C*-4a), 154.2 (*C*-6a), 141.1 (*C*-3), 131.3 (*C*-1), 130.1 (*C*-10), 129.9 (*C*-8), 129.1 (*C*-9), 119.9 (*C*-7), 118.3 (*C*-12a), 117.5 (*C*-2/4), 108.9 (*C*-2/4), 80.5 (*C*($CH_3$)$_3$), 51.7 (*C*-12), 28.4 (C($CH_3$)$_3$) 23.2 ($CH_3$) ppm.

**IR** (ATR): $\tilde{\nu}$ = 3304 (m), 2976 (w), 2923 (m), 2164 (s), 1722 (s), 1638 (s), 1615 (m), 1590 (m), 1528 (s), 1479 (m), 1394 (s), 1367 (w), 1310 (m), 1239 (s), 1159 (s), 1075 (m), 1059 (m), 1034 (w), 974 (w), 953 (w), 917 (w), 881 (m), 856 (w), 829 (m), 796 (m), 763 (m), 694 (w), 618 (m), 570 (m), 449 (m) cm$^{-1}$.

**HR-MS** (ESI, DCM): *m/z* [M+H]$^+$ calculated for $C_{20}H_{22}O_3N_4$+$H^+$: 367.1765; found: 367.1761 ± 1.02 ppm.

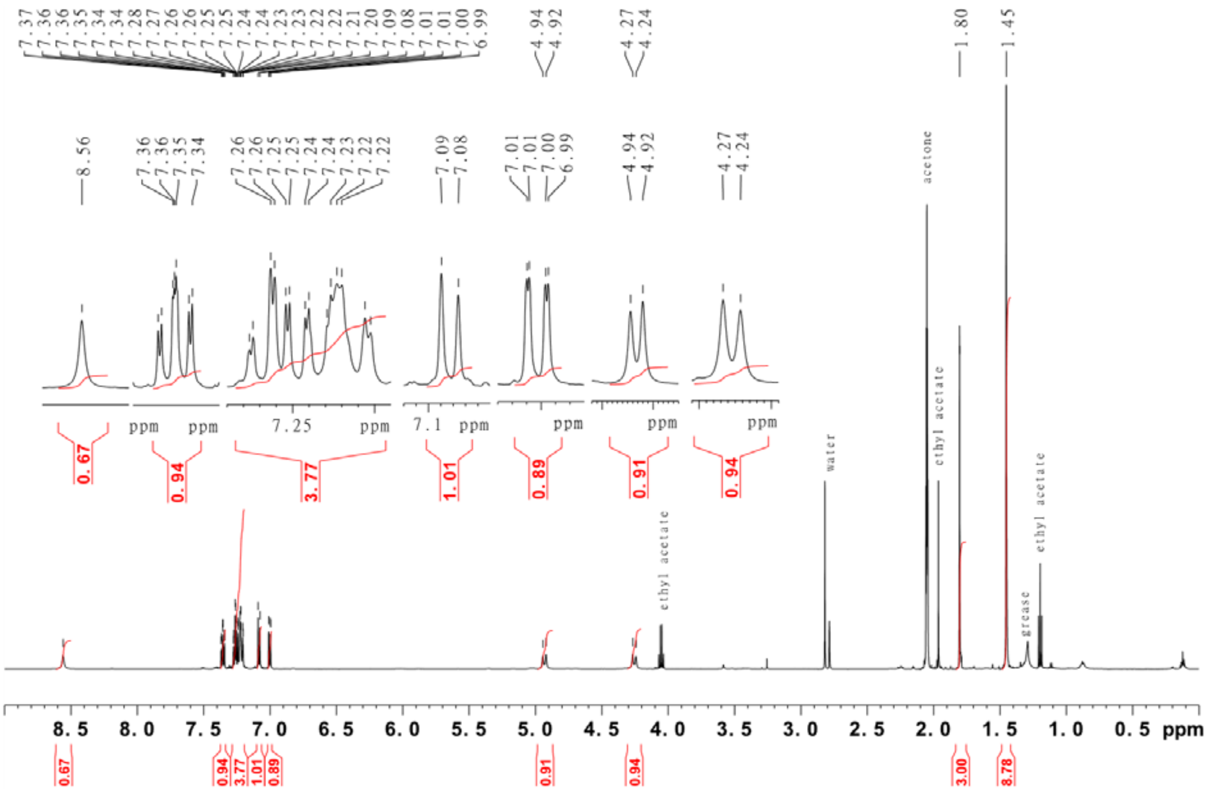


**Figure SII.83:** 600 MHz $^{1}H$-NMR spectrum of *tert*-butyl (*Z*)-(11-acetyl-11,12-dihydrodiebenzo[*c,g*][1,2,5]triazocin-3-yl)carbamate (**19**).

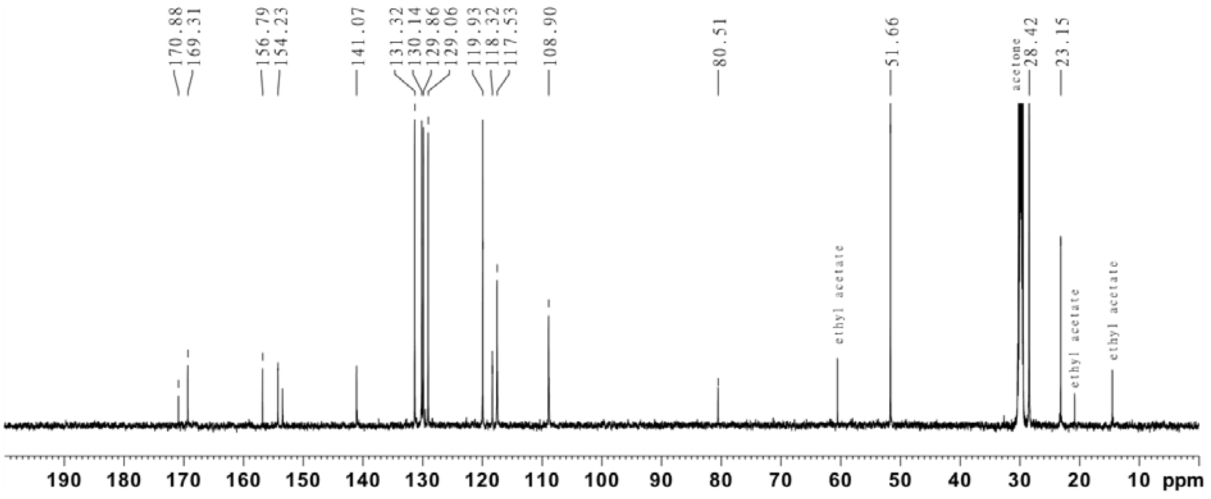


**Figure SII.84:** 150 MHz $^{13}C\{^{1}H\}$-NMR spectrum of *tert*-butyl (*Z*)-(11-acetyl-11,12-dihydrodiebenzo[*c,g*][1,2,5]triazocin-3-yl)carbamate (**19**).

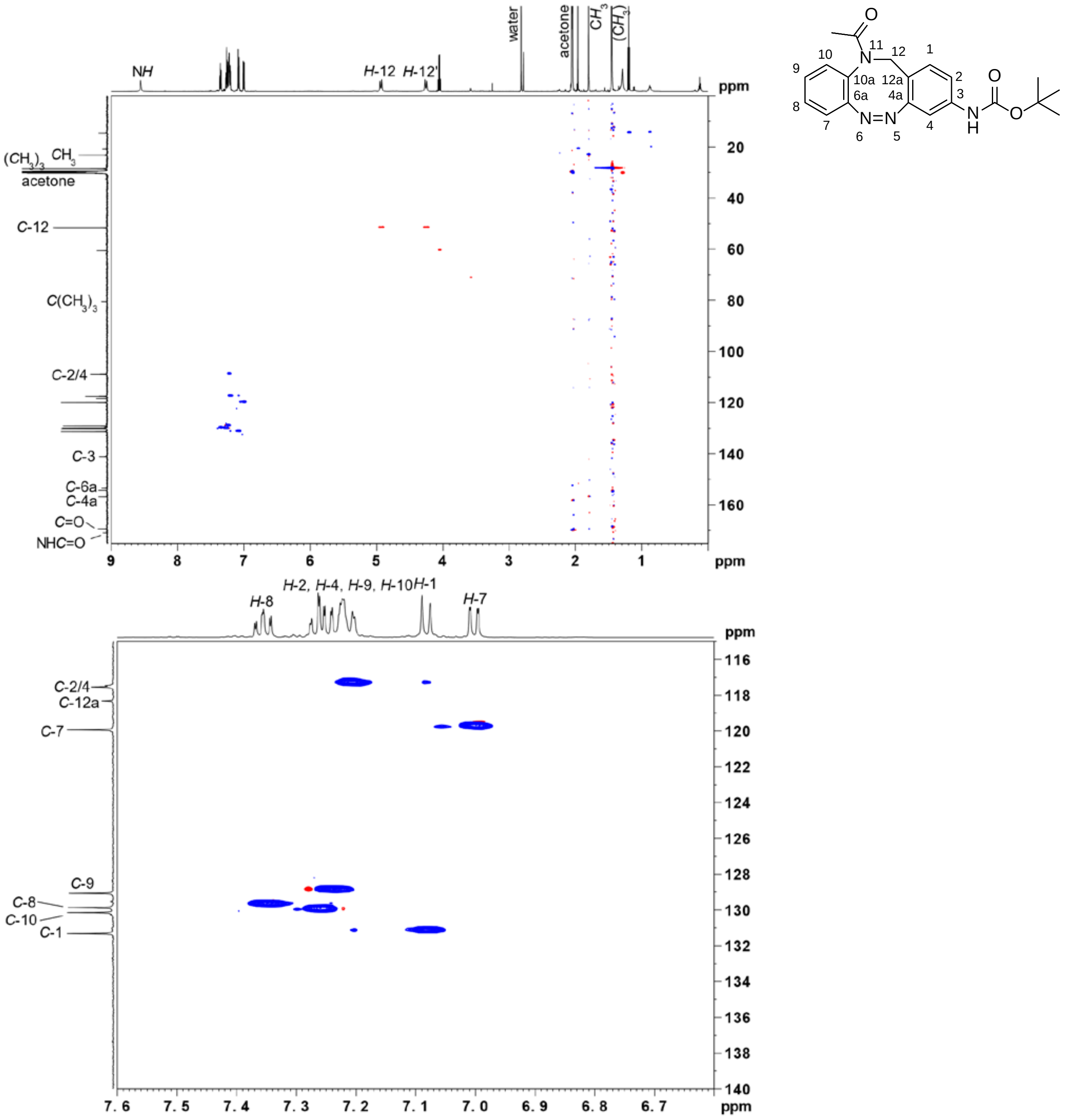


**Figure SII.85:** 600 MHz $^{1}H$-$^{13}C$ HSQC spectrum of *tert*-butyl (*Z*)-(11-acetyl-11,12-dihydrodiebenzo[*c*,*g*][1,2,5]triazocin-3-yl)carbamate (**19**).

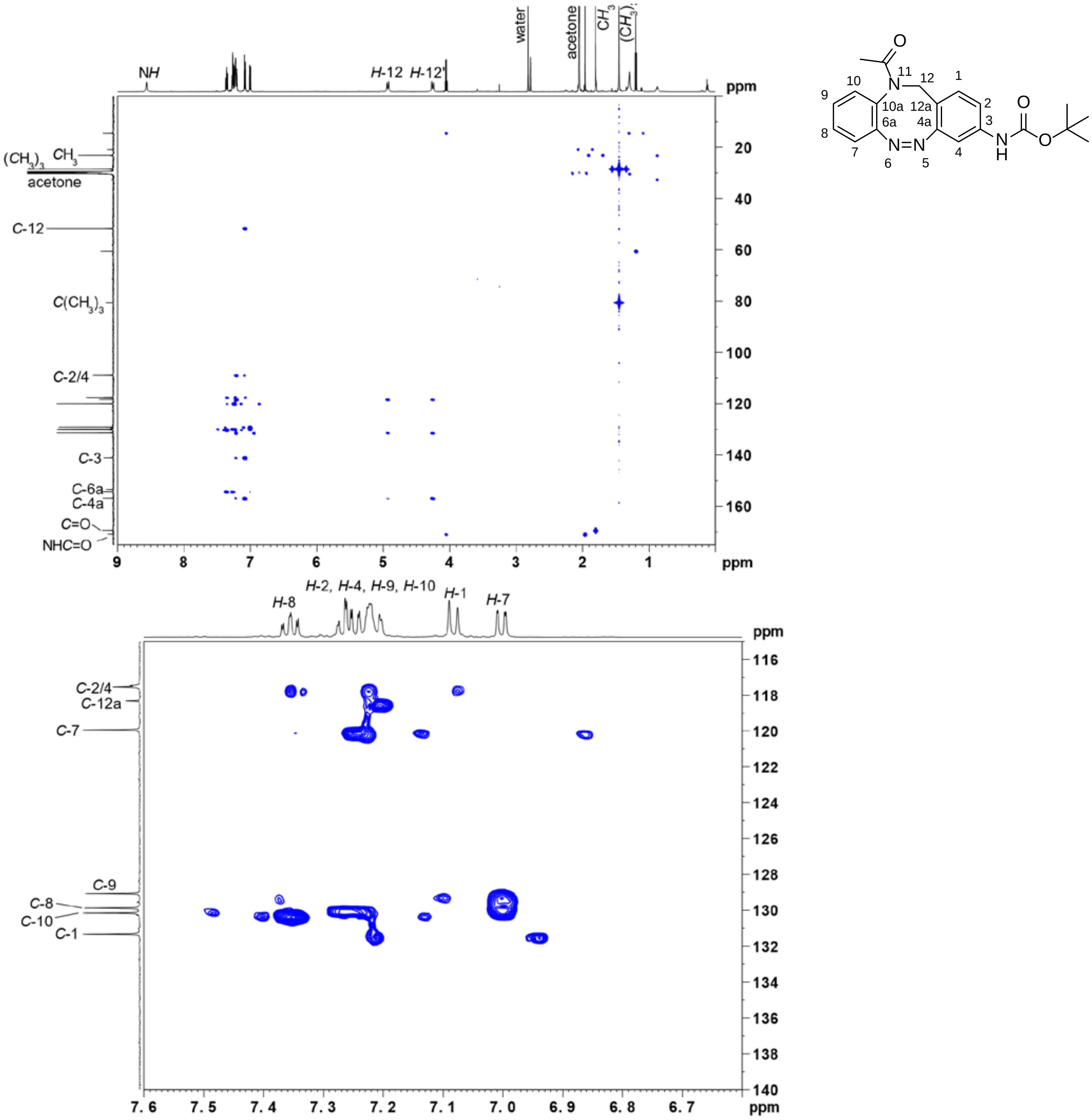


**Figure SII.86:** 600 MHz HMBC spectrum of *tert*-butyl (*Z*)-(11-acetyl-11,12-dihydrodiebenzo[*c,g*][1,2,5]triazocin-3-yl)carbamate (**19**).

### II.2.12 Synthesis of (*Z*)-1-(3-(diphenylamino)dibenzo[*c,g*][1,2,5]triazocin-11(12*H*)-yl)ethan-1-one (20)

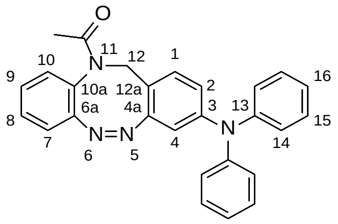


In a manner analogous to [2] halogenated *N*-acetyl-diazocine (2, 20.0 mg, 60.6 µmol, 3: 23.0 mg, 60.6 µmol), diphenylamine (15.4 mg, 90.9 µmol), Xantphos Pd G3 (2.90 mg, 3.03 µmol) and caesium carbonate (19.7 mg, 60.6 µmol) were dissolved in 2.4 mL of dry 1,4-dioxane under a nitrogen atmosphere, heated to 100 °C with an oil bath and stirred for 16 h at given temperature. After cooling to rt. deionized $H_2O$ and 5 mL ethyl acetate were added and the reaction mixture was extracted with 10 mL of ethyl acetate twice, dried over $MgSO_4$ and the solvent was evaporated. Column chromatography on silica (ethyl acetate/cyclohexane 1:1, $R_f$ = 0.30) gave the product as yellow solid (from bromide **2**: 6.30 mg, 15.1 µmol, 25%, **3**: 11.8 mg, 28.2 µmol, 47%).

**melting point:** 160 °C

**$^1$H-NMR** (500 MHz, acetone-$d_6$, 298 K): $\delta$ = 7.46 (td, $^3J$ = 7.9 Hz, $^4J$ = 1.9 Hz, 1 H, *H*-9), 7.32-7.27 (m, 6 H, *H*-7, *H*-8, *H*-14/15), 7.10-7.06 (m, 2 H, *H*-16), 7.04 (d, $^3J$ = 8.3 Hz, 1 H, *H*-1), 6.98-6.93 (5 H, *H*-10, *H*-14/15), 6.66 (dd, $^3J$ = 8.3 Hz, $^4J$ = 2.4 Hz, 1 H, *H*-2), 6.44 (d, $^4J$ = 2.4 Hz, 1 H, *H*-4), 4.77 (d, $^2J$ = 14.3 Hz, 1 H, *H*-12), 4.26 (d, $^2J$ = 14.3 Hz, 1 H, *H*-12'), 1.81 (s, 3 H, -C*H*$_3$) ppm.

**$^{13}$C{$^1$H}-NMR** (125 MHz, acetone-$d_6$, 298 K): $\delta$ = 169.3 (*C*=O), 157.4 (*C*-4a), 154.5 (*C*-6a), 149.2 (*C*-3), 147.9 (*C*-13), 131.9 (*C*-1), 130.42 (*C*-14/15), 130.34 (*C*-7), 130.04 (*C*-10a), 129.8 (*C*-9), 129.1 (*C*-8), 125.6 (*C*-14/15), 124.7 (*C*-16), 121.5 (*C*-2), 119.8 (*C*-10), 118.2 (*C*-12a), 112.8 (*C*-4), 51.6 (*C*-12) 23.2 (*C*H$_3$) ppm.

**IR** (ATR): $\tilde{\nu}$ = 2921 (w), 2228 (w), 2185 (w), 2084 (w), 2021 (w), 1659 (s), 1610 (w), 1592 (s), 1481 (s), 1384 (s), 1333 (s), 1304 (w), 1290 (m), 1265 (s), 1221 (w), 1171 (w), 1095 (w), 1075 (w), 1029 (w), 977 (w), 901 (w), 840 (w), 811 (m), 758 (m), 745 (m), 697 (s), 631 (m), 586 (m), 508 (s), 483 (m) cm$^{-1}$.

**HR-MS** (ESI, DCM): *m/z* [M+H]$^+$ calculated for $C_{27}H_{22}ON_4+H^+$: 419.1866; found: 419.1863 ± 0.85 ppm.

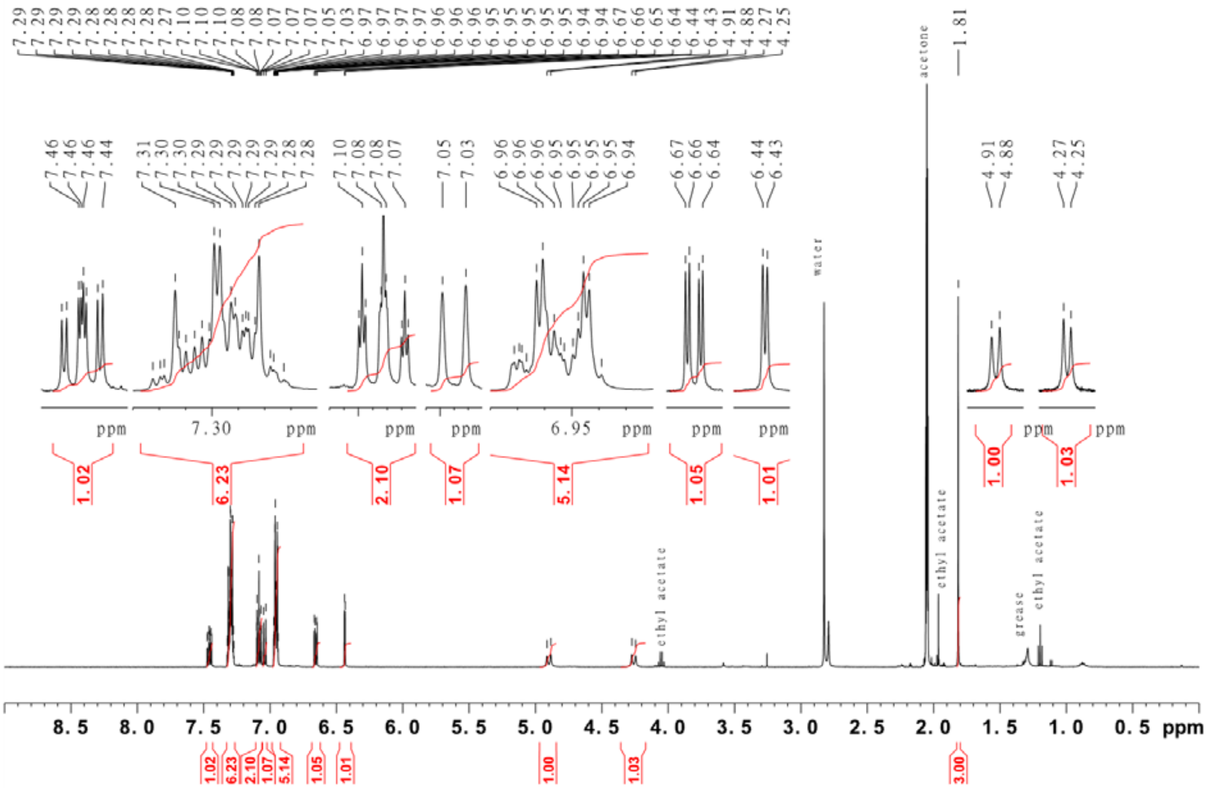


**Figure SII.87:** 500 MHz $^{1}H$-NMR spectrum of (*Z*)-1-(3-(diphenylamino)dibenzo[*c*,*g*][1,2,5]triazocin-11(12*H*)-yl)ethan-1-one (**20**).

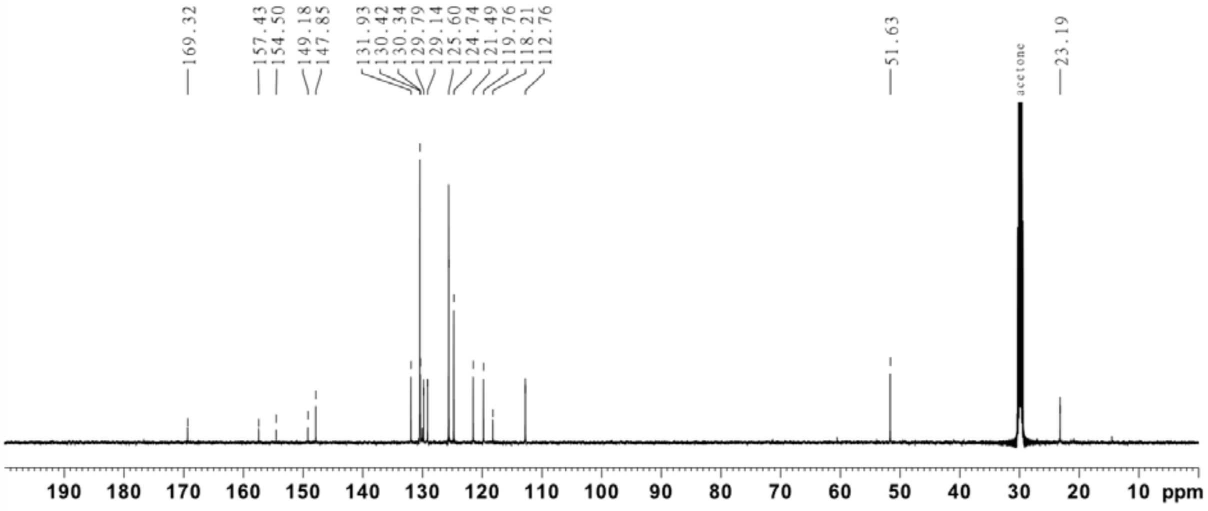


**Figure SII.88:** 125 MHz $^{13}C\{^{1}H\}$-NMR spectrum of (*Z*)-1-(3-(diphenylamino)dibenzo[*c*,*g*][1,2,5]triazocin-11(12*H*)-yl)ethan-1-one (**20**).

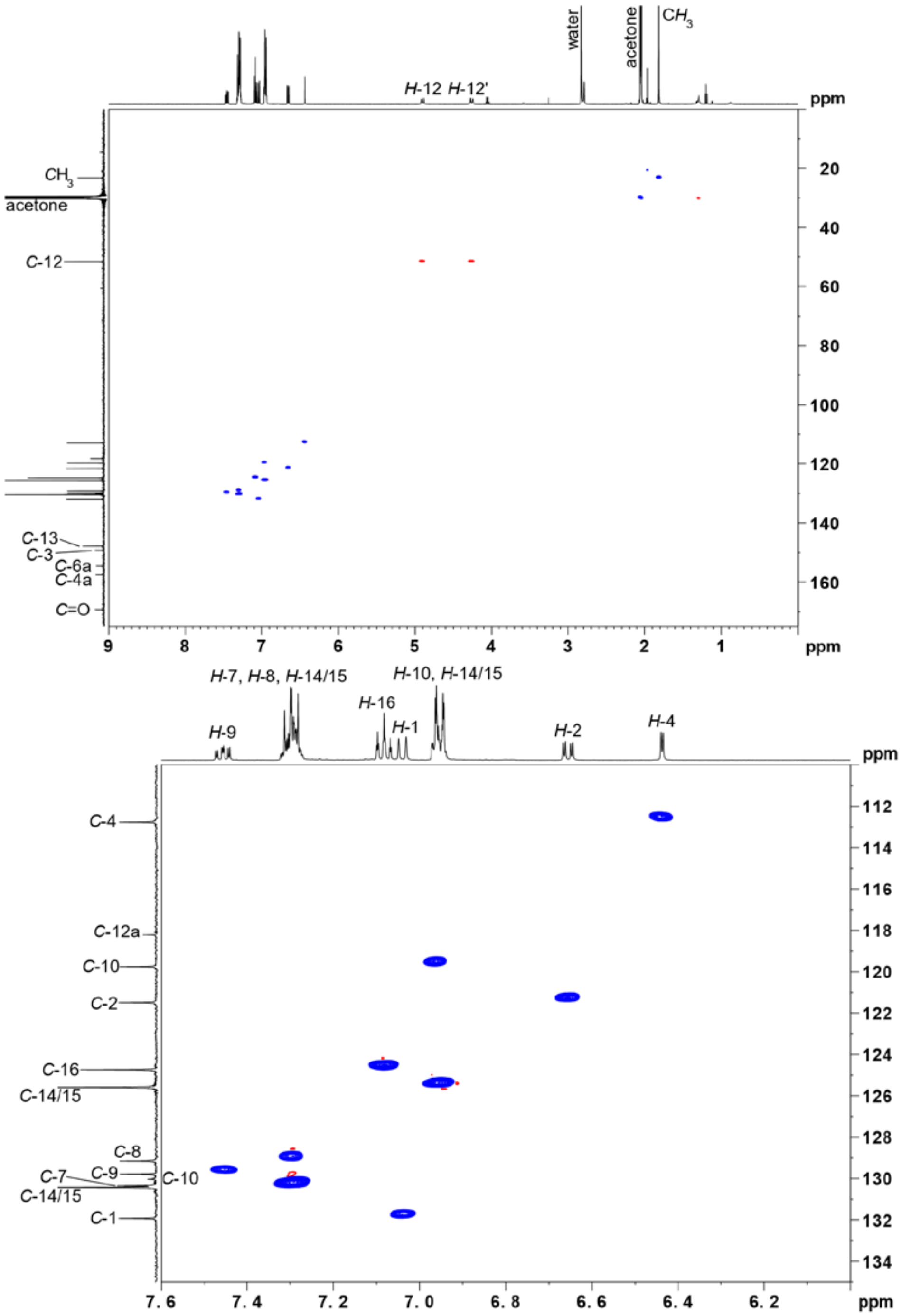


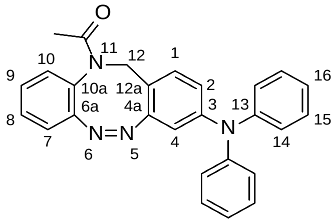


**Figure SII.89:** 500 MHz $^{1}H$-$^{13}C$ HSQC spectrum of (*Z*)-1-(3-(diphenylamino)dibenzo[*c*,*g*][1,2,5]triazocin-11(12*H*)-yl)ethan-1-one (**20**).

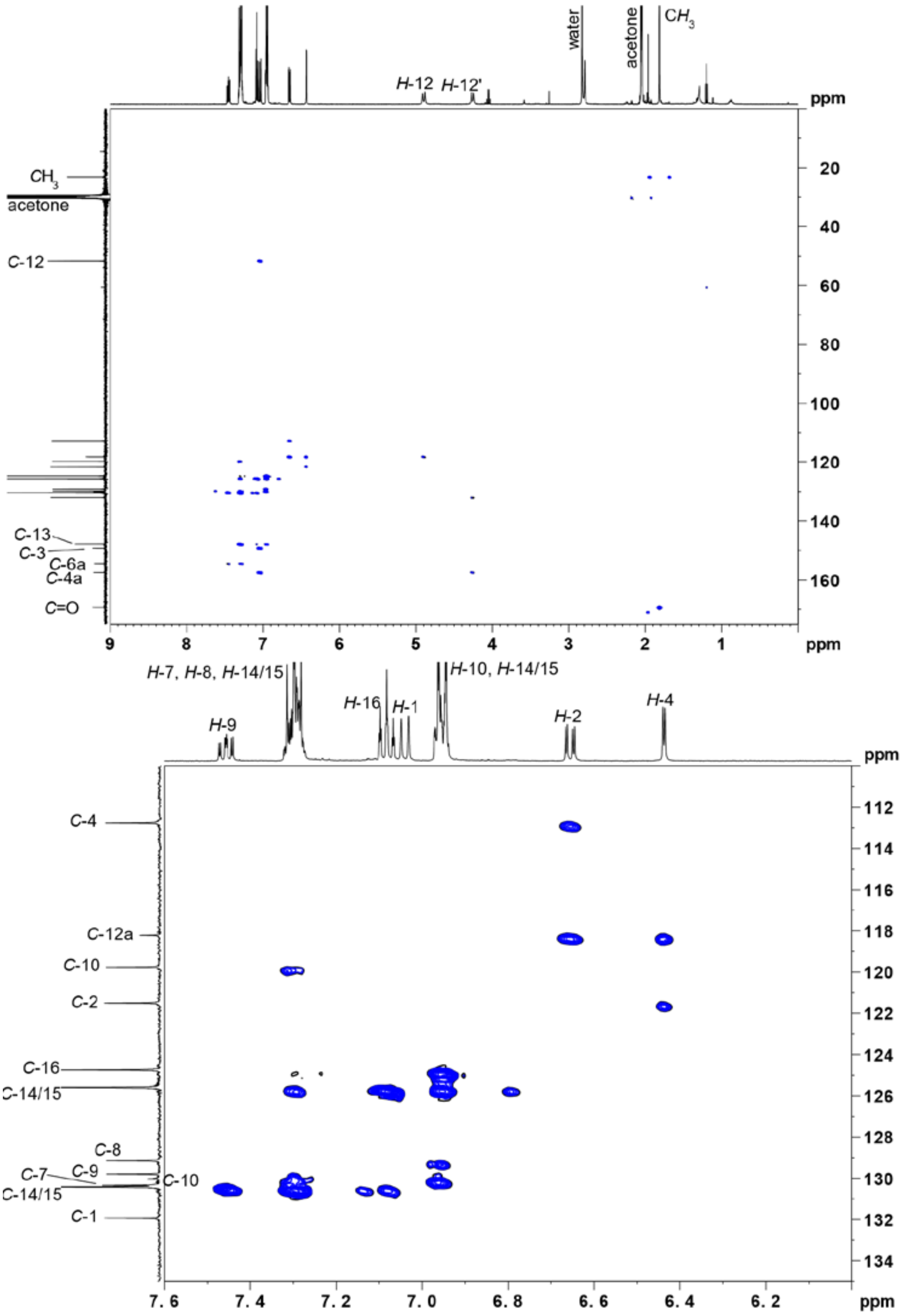


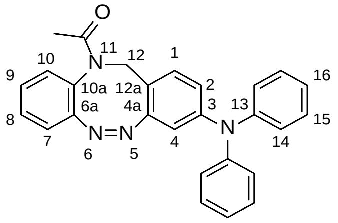


**Figure SII.90:** 500 MHz HMBC spectrum of (*Z*)-1-(3-(diphenylamino)dibenzo[*c*,*g*][1,2,5]triazocin-11(12*H*)-yl)ethan-1-one (**20**).

## II.2.13 Synthesis of (*Z*)-1-(3-aminodibenzo[*c,g*][1,2,5]triazocin-11(12*H*)-yl)ethan-1-one (21)

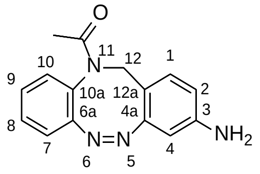


*Tert*-butyl (*Z*)-(11-acetyl-11,12-dihydrodiebenzo[*c,g*][1,2,5]triazocin-3-yl)carbamate (**21**, 17.3 mg, 47.2 µmol) was dissolved in 25 mL dichloromethane and 5 mL of trifluoroacetic acid were added. The reaction mixture was stirred at room temperature for 16 h and then neutralized with saturated aqueous $NaHCO_3$ solution. The organic layer was separated and the aqueous layer was extracted twice with 10 mL dichloromethane, dried over $MgSO_4$ and the solvent was evaporated. Column chromatography on silica (ethyl acetate/cyclohexane 1:1, $R_f$ = 0.15) gave the product as yellow solid (7.50 mg, 28.2 µmol, 60%).

**melting point:** 178 °C

**$^1$H-NMR** (600 MHz, $CDCl_3$, 298 K): $\delta$ = 7.27-7.24 (m, 1 H, *H*-8), 7.14 (td, $^3J$ = 7.7 Hz, $^4J$ = 1.4 Hz, 1 H, *H*-9), 6.99 (dd, $^3J$ = 7.9 Hz, $^4J$ = 0.9 Hz, 1 H, *H*-10), 6.87 (dd, $^3J$ = 7.8 Hz, $^4J$ = 1.2 Hz, 1 H, *H*-7), 6.80 (d, $^3J$ = 8.2 Hz, , 1 H, *H*-1), 6.33 (dd, $^3J$ = 8.1 Hz, $^4J$ = 2.4 Hz, 1 H, *H*-2), 6.16 (d, $^4J$ = 2.4 Hz, 1 H, *H*-4), 4.86 (d, $^2J$ = 14.3 Hz, 1 H, *H*-12), 4.27 (d, $^2J$ = 14.3 Hz, 1 H, *H*-12'), 3.72 (br. s, 2 H, -N*H*$_2$), 1.92 (s, 3 H, -C*H*$_3$) ppm.

**$^{13}$C{$^1$H}-NMR** (150 MHz, $CDCl_3$, 298 K): $\delta$ = 170.0 (*C*=O), 156.6 (*C*-4a), 152.9 (*C*-6a), 146.8 (*C*-3), 131.0 (*C*-1), 129.0 (*C*-10a), 128.82 (*C*-10), 128.67 (*C*-8), 127.9 (*C*-9), 119.3 (*C*-7), 113.9 (*C*-2), 113.2 (*C*-12a), 104.9 (*C*-4), 51.5 (*C*-12) 23.3 (*C*H$_3$) ppm.

**IR** (ATR): $\tilde{\nu}$ = 3346 (m), 2922 (m), 2852 (w), 1653 (s), 1608 (s), 1504 (w), 1480 (s), 1389 (s), 1372 (w), 1349 (s), 1326 (w), 1283 (m), 1241 (w), 1209 (w), 1148 (w), 1091 (w), 1071 (w), 975 (w), 907 (w), 867 (w), 827 (m), 802 (w), 760 (m), 742 (m), 718 (w), 696 (w), 605 (m), 585 (w), 541 (w), 515 (m) cm$^{-1}$.

**HR-MS** (ESI, EtOAc): *m/z* $[M+Na]^+$ calculated for $C_{15}H_{14}ON_4+Na^+$: 289.1060; found: 289.1057 ± 0.98 ppm.

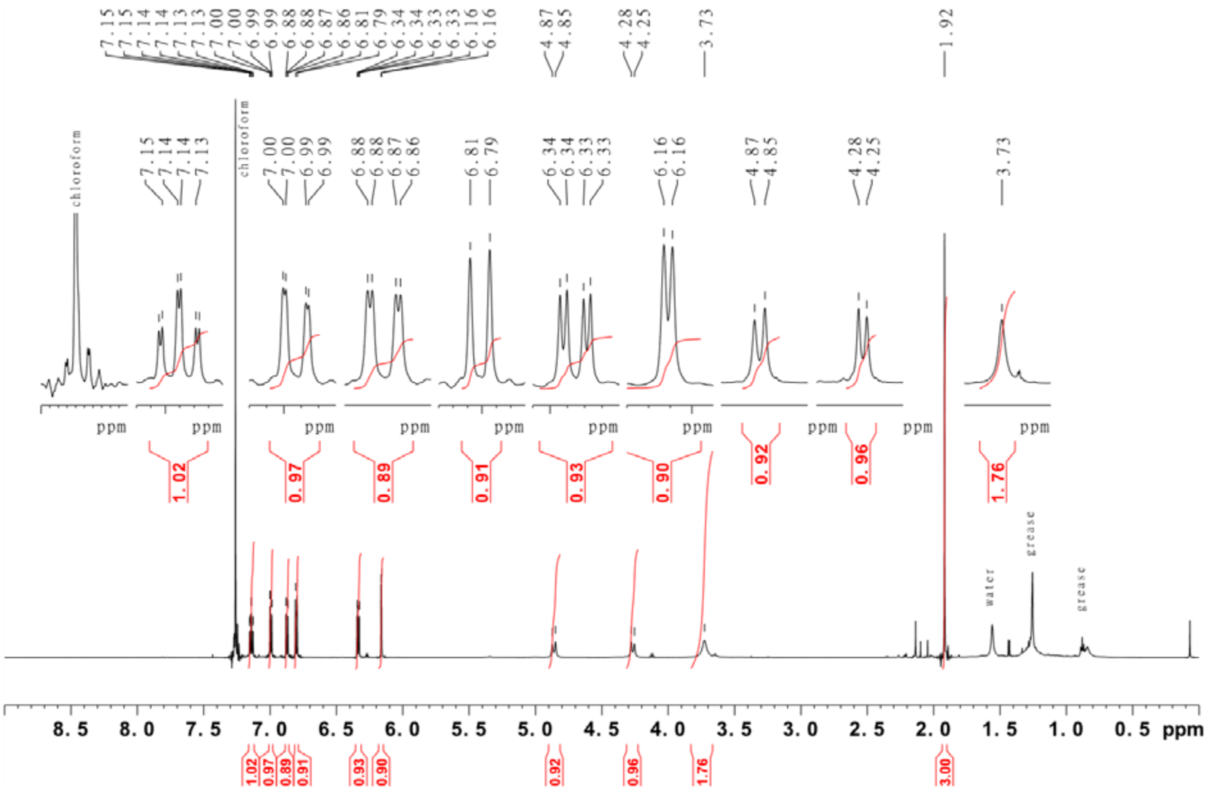


**Figure SII.91:** 600 MHz $^{1}$H-NMR spectrum of (*Z*)-1-(3-aminodibenzo[*c*,*g*][1,2,5]triazocin-11(12*H*)-yl)ethan-1-one (**21**).

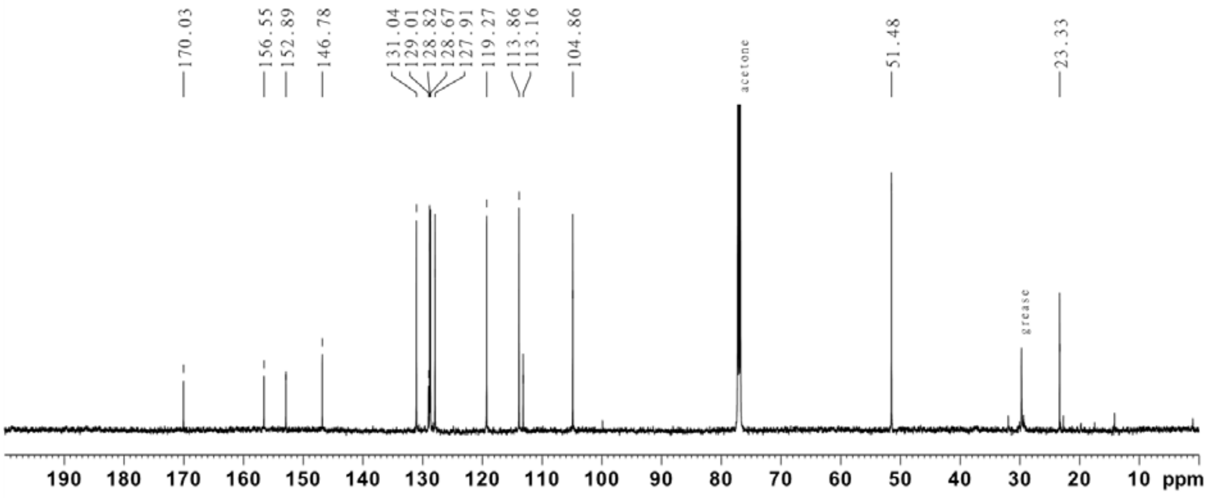


**Figure SII.92:** 150 MHz $^{13}$C{$^{1}$H}-NMR spectrum of (*Z*)-1-(3-aminodibenzo[*c*,*g*][1,2,5]triazocin-11(12*H*)-yl)ethan-1-one (**21**).

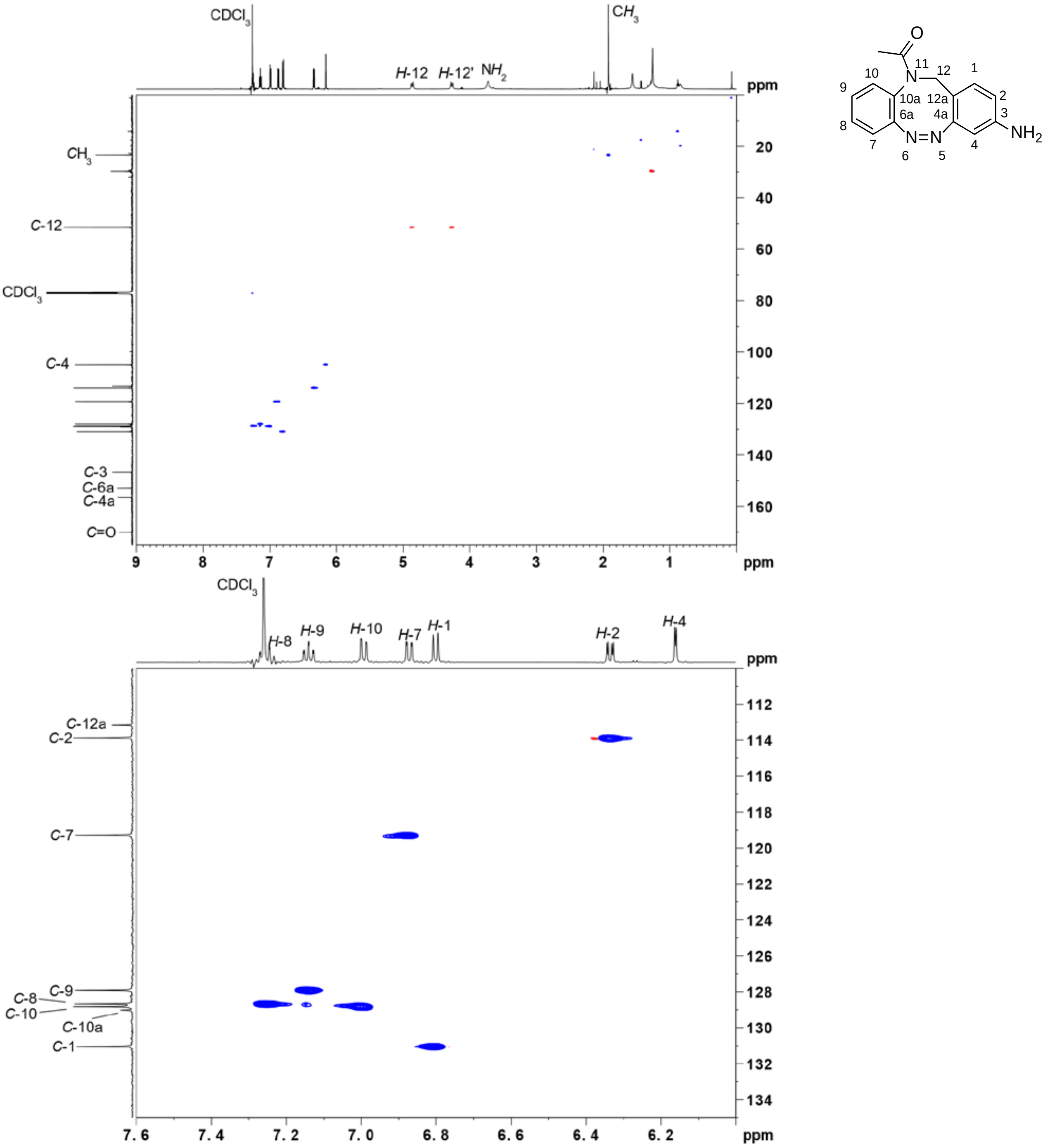


**Figure SII.93:** 600 MHz $^{1}H$-$^{13}C$ HSQC spectrum of (*Z*)-1-(3-aminodibenzo[*c*,*g*][1,2,5]triazocin-11(12*H*)-yl)ethan-1-one (**21**).

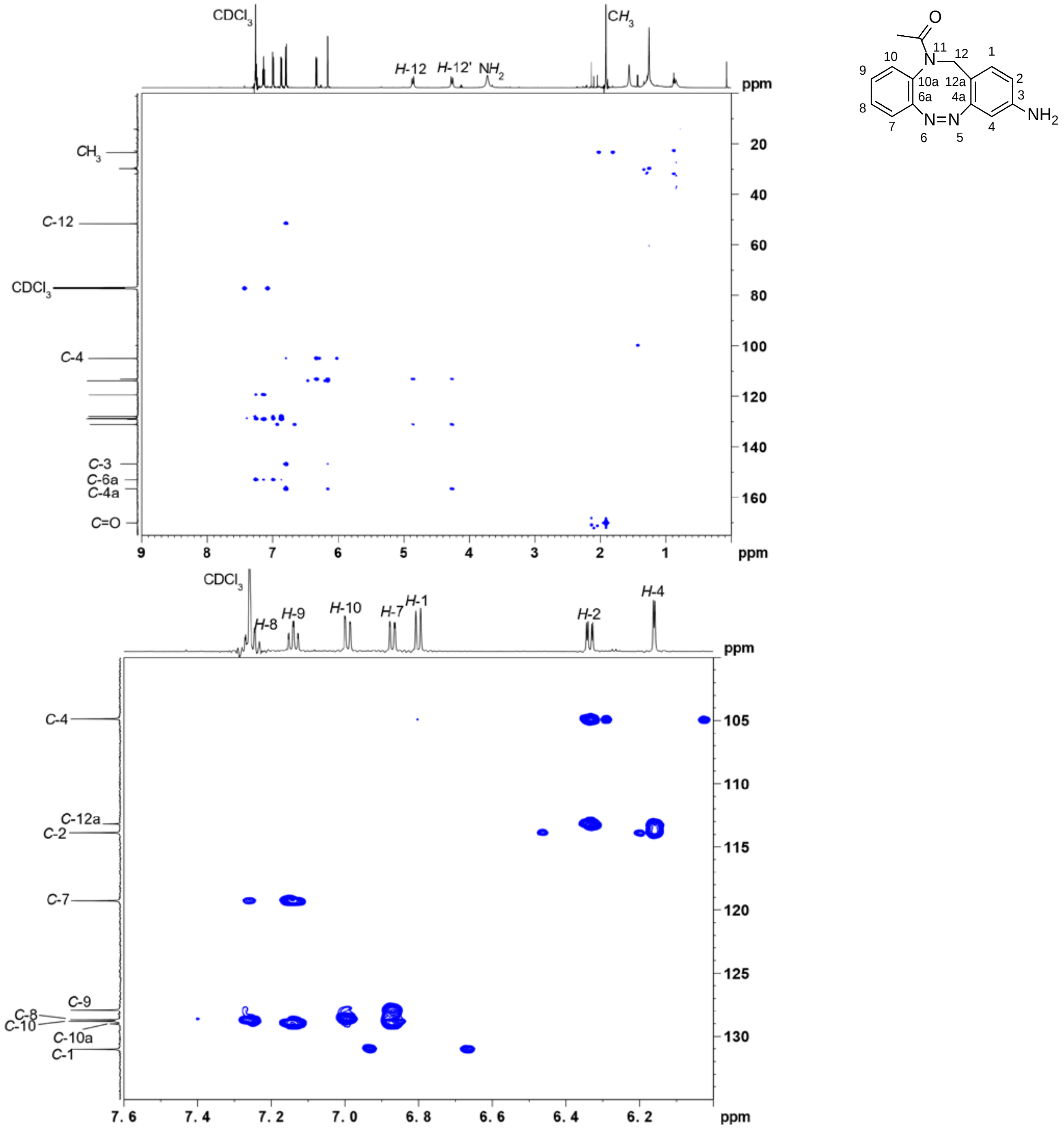


**Figure SII.94:** 600 MHz HMBC spectrum of (*Z*)-1-(3-aminodibenzo[*c*,*g*][1,2,5]triazocin-11(12*H*)-yl)ethan-1-one (**21**).

## II.2.14 Synthesis of (*Z*)-11-acetyl-11,12-dihydrodibenzo[*c,g*][1,2,5]triazocine-3-carbonitrile (23)

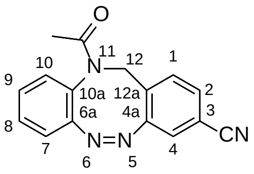


Halogenated *N*-acetyl-diazocine (**2**: 20.0 mg, 60.6 µmol, **3**: 23.0 mg, 60.6 µmol), 1,1'-bis(diphenylphosphino)ferrocene (1.00 mg, 1.81 µmol), Pd(dba)$_2$ (1.00 mg, 1.21 µmol) and zinc cyanide (3.55 mg, 30.3 µmol) were dissolved in 2.4 mL of dry DMAC under a nitrogen atmosphere, heated to 130 °C with an oil bath and stirred for 16 h at given temperature. After cooling to rt. the reaction mixture was extracted with 10 mL of DCM twice, dried over $MgSO_4$ and the solvent was evaporated. Column chromatography on silica (ethyl acetate/cyclohexane 1:1, $R_f$ = 0.23) gave the product as yellow solid (from bromide **2**: 10.2 mg, 36.9 µmol, 61%; from iodide **3**: 13.6 mg, 49.1 µmol, 81%).

**melting point:** 194 °C

**$^1$H-NMR** (600 MHz, acetone-*d*$_6$, 298 K): $\delta$ = 7.56 (dd, $^3J$ = 7.9 Hz, $^4J$ = 1.7 Hz, 1 H, *H*-2), 7.47 (d, $^3J$ = 8.0 Hz, 1 H, *H*-1), 7.42 (td, $^3J$ = 7.9 Hz, $^4J$ = 1.4 Hz, 1 H, *H*-8), 7.37 (d, $^4J$ = 1.5 Hz, 1 H, *H*-4), 7.35 (dd, $^3J$ = 8.0 Hz, $^4J$ = 1.1 Hz, 1 H, *H*-10), 7.31 (td, $^3J$ = 7.4 Hz, $^4J$ = 1.4 Hz, 1 H, *H*-9), 7.09 (dd, $^3J$ = 7.9 Hz, $^4J$ = 1.1 Hz, 1 H, *H*-7), 5.21 (d, $^2J$ = 15.1 Hz, 1 H, *H*-12), 4.41 (d, $^2J$ = 15.0 Hz, 1 H, *H*-12'), 1.80 (s, 3 H, -C*H*$_3$) ppm.

**$^{13}$C{$^1$H}-NMR** (150 MHz, acetone-*d*$_6$, 298 K): $\delta$ = 169.4 (*C*=O), 156.3 (*C*-4a), 154.1 (*C*-6a), 132.1 (*C*-1), 131.9 (*C*-2), 130.9 (*C*-12a), 130.6 (*C*-8), 130.2 (*C*-10), 129.8 (*C*-9), 129.1 (*C*-10a), 123.7 (*C*-4), 120.1 (*C*-7), 118.1 (-*C*N), 113.2 (*C*-3), 51.8 (*C*-12) 22.9 (*C*H$_3$) ppm.

**IR** (ATR): $\tilde{\nu}$ = 3058 (w), 2923 (m), 2853 (w), 2233 (m), 1662 (s), 1589 (w), 1556 (w), 1521 (w), 1475 (m), 1448 (w), 1377 (s), 1340 (w), 1299 (m), 1228 (w), 1148 (w), 1109 (w), 1078 (w), 1032 (w), 1019 (w), 973 (w), 930 (w), 884 (w), 844 (w), 832 (w), 813 (w), 770 (m), 741 (m), 693 (w), 634 (w), 596 (m) cm$^{-1}$.

**HR-MS** (ESI, DCM): *m/z* [M+H]$^+$ calculated for $C_{16}H_{12}ON_4$+H$^+$: 277.1084; found: 277.1080 ± 1.40 ppm.

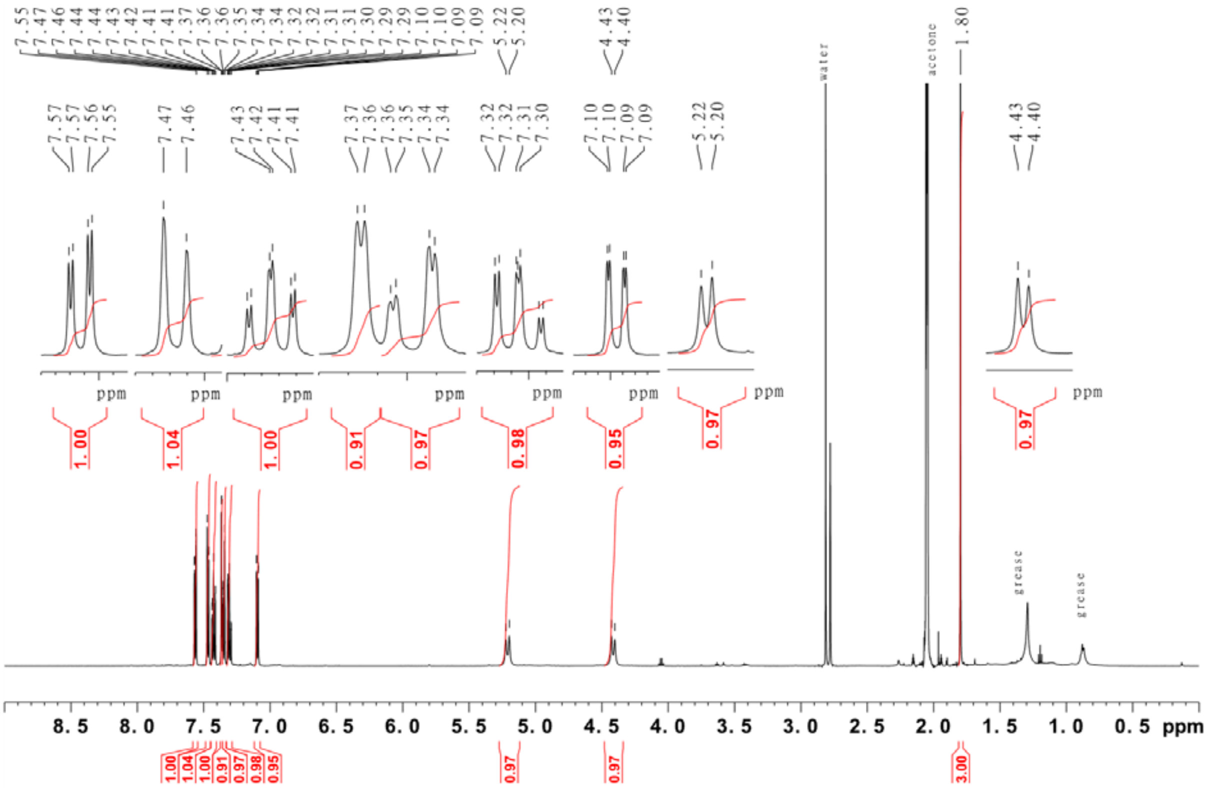

**Figure SII.95:** 600 MHz $^{1}$H-NMR spectrum of (*Z*)-11-acetyl-11,12-dihydrodibenzo[*c,g*][1,2,5]triazocine-3-carbonitrile (**23**).

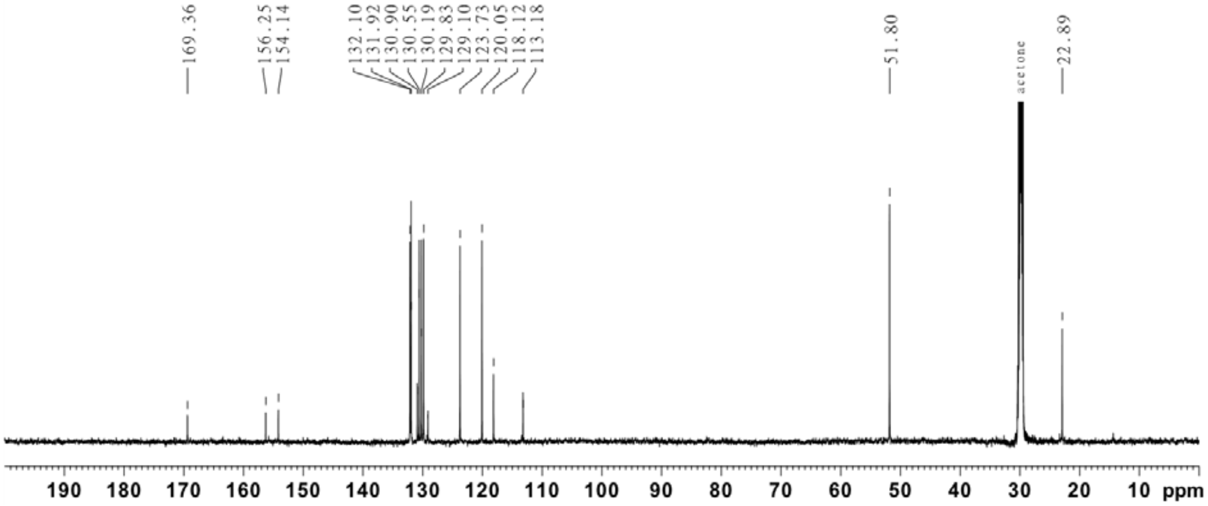

**Figure SII.96:** 150 MHz $^{13}$C{$^{1}$H}-NMR spectrum of (*Z*)-11-acetyl-11,12-dihydrodibenzo[*c,g*][1,2,5]triazocine-3-carbonitrile (**23**).

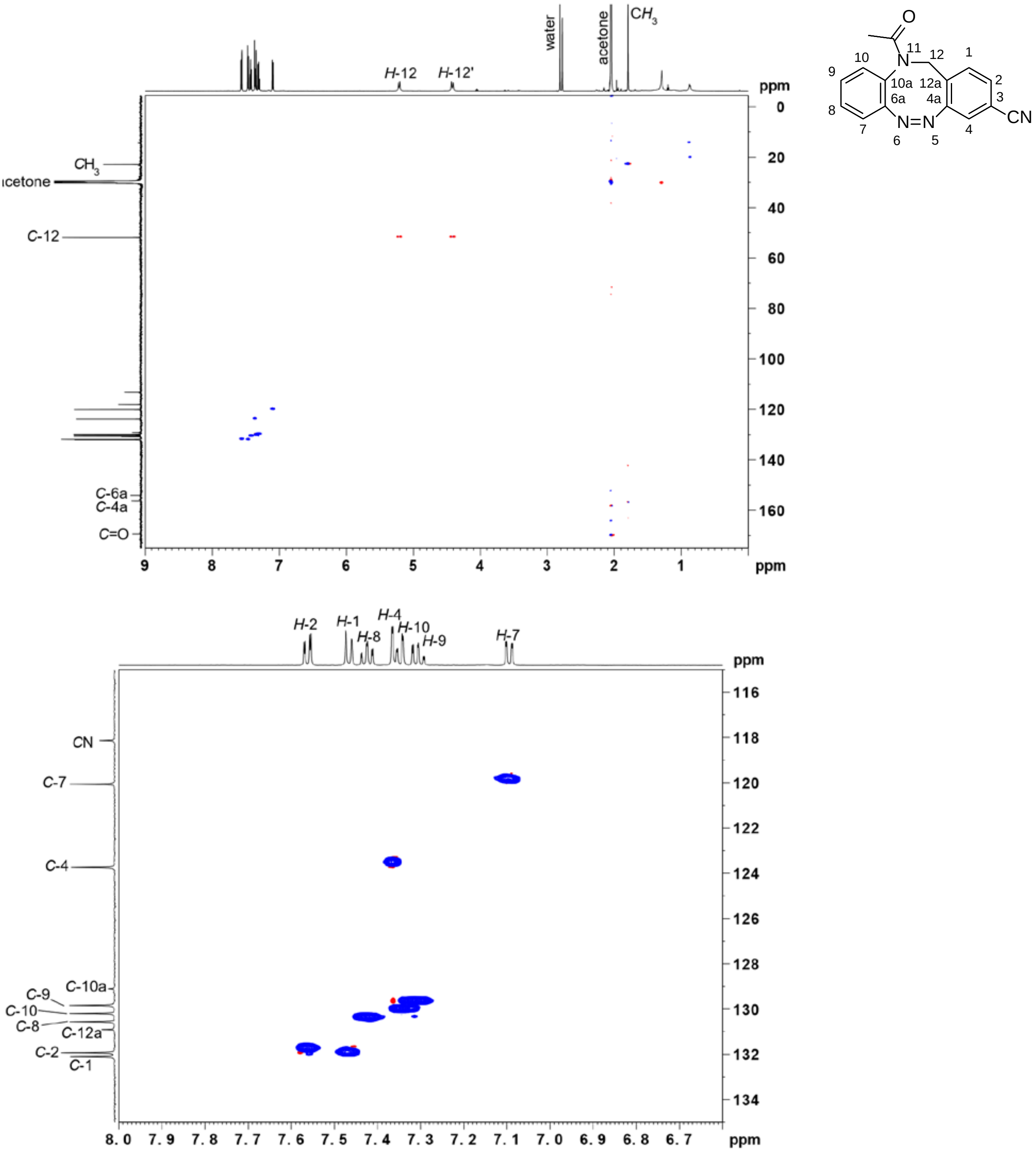


**Figure SII.97:** 600 MHz $^{1}H$-$^{13}C$ HSQC spectrum of *Z*)-11-acetyl-11,12-dihydrodibenzo[*c,g*][1,2,5]triazocine-3-carbonitrile (**23**).

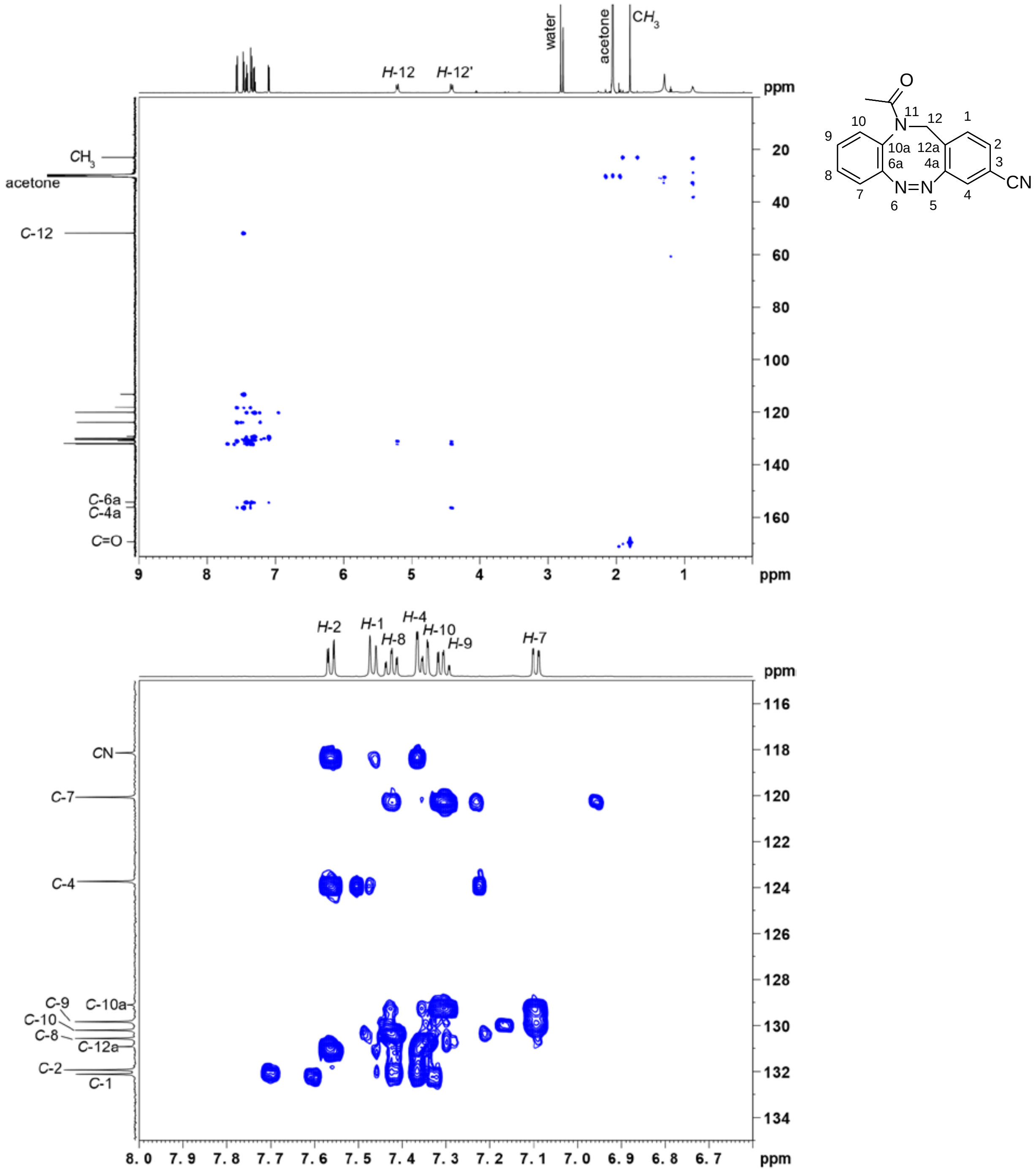


**Figure SII.98:** 600 MHz HMBC spectrum of *Z*)-11-acetyl-11,12-dihydrodibenzo[*c*,*g*][1,2,5]triazocine-3-carbonitrile (**23**).

# III. UV-vis switching experiments

All samples were irradiated to the photostationary state (PSS) with 405 nm in acetonitrile or water. The UV-vis spectra were measured at 250 µM concentrations at 25 °C, except pyridine-substituted *N*-acetyl-diazocine **14** (2.55 mM due to low synthesis yield).

Errors of the thermal relaxation were calculated via the following equation (1).[4] The standard error of the linear regression is given as $\sigma$ and a confidence interval of 95% was estimated.

$$\left[t_{1/2} \pm 1.96 \frac{log2}{k^2} \sigma\right] \quad (1)$$

## III.1 (Z)-(3,8-dibromodibenzo[*c,g*][1,2,5]triazocin-11(12H)-yl)ethan-1-one (4)

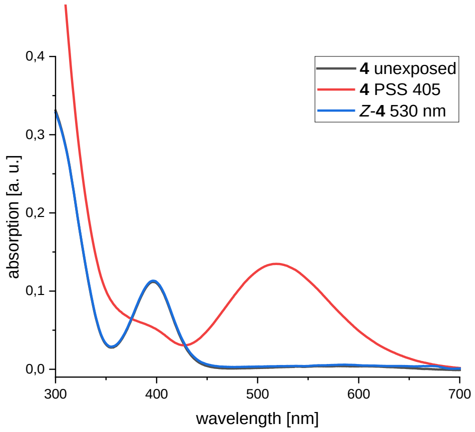


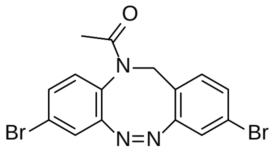


**4**

**Figure SIII.1:** UV-vis spectra of **4** in MeCN at 25 °C. The unexposed sample is plotted in black, the spectra of the PSS between *Z*-**4** and *E*-**4** after irradiation with 405 nm in red and the spectra of *Z*-**4** after irradiation with 530 nm in blue.

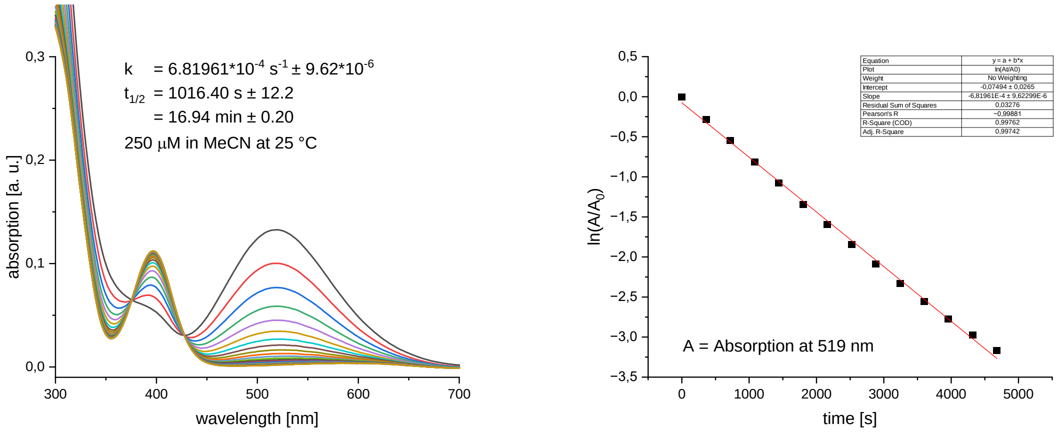


**Figure SIII.2:** UV-vis spectra of the half-life determination of **4** in MeCN at 25 °C (left) with the corresponding first order kinetics plot (right).

## III.2 (*Z*)-1-(3-phenyldibenzo[*c*,*g*][1,2,5]triazocin-11(12*H*)-yl)ethan-1-one (7)

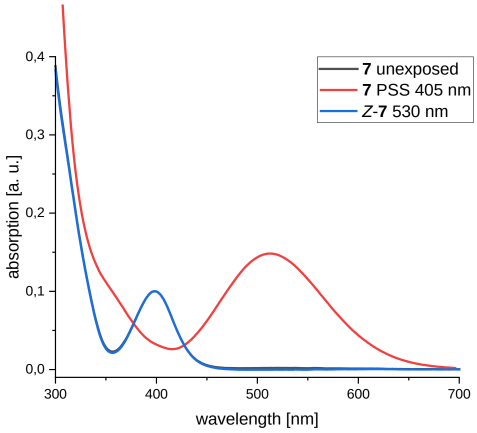


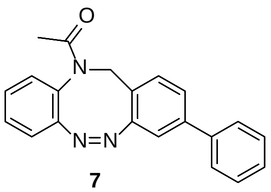


**7**

**Figure SIII.3:** UV-vis spectra of **7** in MeCN at 25 °C. The unexposed sample is plotted in black, the spectra of the PSS between *Z*-**7** and *E*-**7** after irradiation with 405 nm in red and the spectra of *Z*-**7** after irradiation with 530 nm in blue.

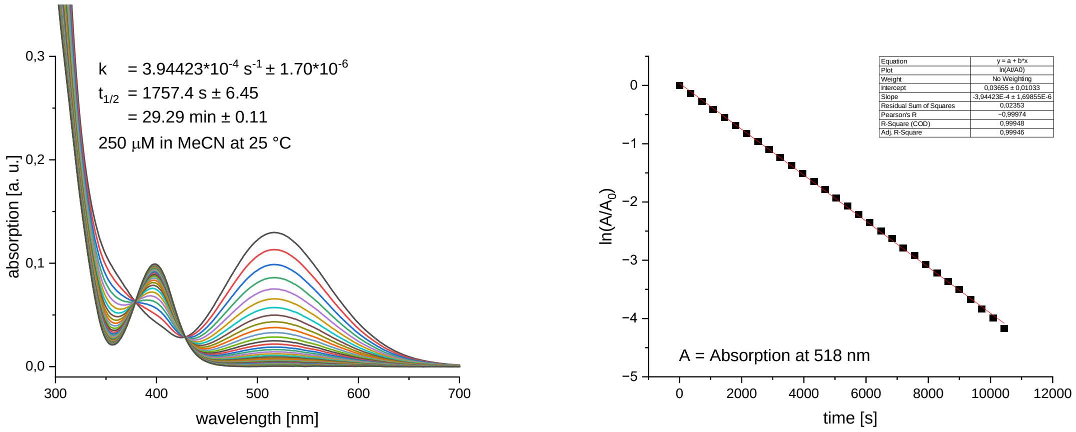


**Figure SIII.4:** UV-vis spectra of the half-life determination of **7** in MeCN at 25 °C (left) with the corresponding first order kinetics plot (right).

## III.3 (*Z*)-1-(3-vinyldibenzo[*c*,*g*][1,2,5]triazocin-11(12*H*)-yl)ethan-1-one (8)

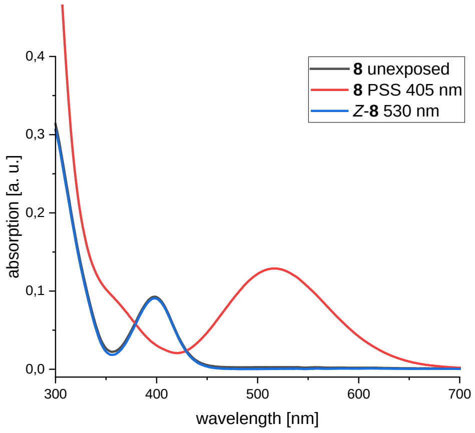


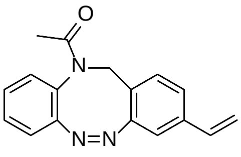


**8**

**Figure SIII.5:** UV-vis spectra of **8** in MeCN at 25 °C. The unexposed sample is plotted in black, the spectra of the PSS between *Z*-**8** and *E*-**8** after irradiation with 405 nm in red and the spectra of *Z*-**8** after irradiation with 530 nm in blue.

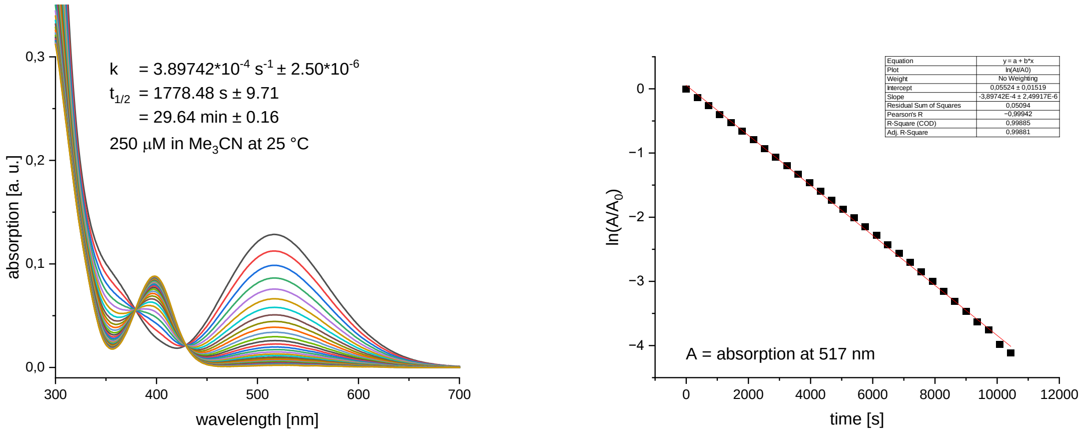


**Figure SIII.6:** UV-vis spectra of the half-life determination of **8** in MeCN at 25 °C (left) with the corresponding first order kinetics plot (right).

## III.4 (*Z*)-1-(3-(2,6-dimethylphenyl)dibenzo[*c*,*g*][1,2,5]triazocin-11(12*H*)-yl)ethan-1-one (9)

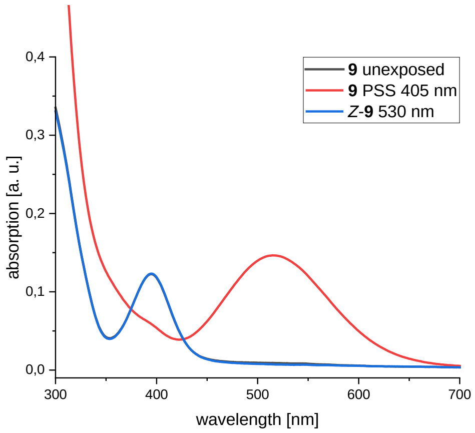


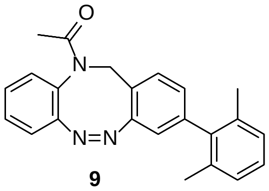

**9**

**Figure SIII.7:** UV-vis spectra of **9** in MeCN at 25 °C. The unexposed sample is plotted in black, the spectra of the PSS between *Z*-**9** and *E*-**9** after irradiation with 405 nm in red and the spectra of *Z*-**9** after irradiation with 530 nm in blue.

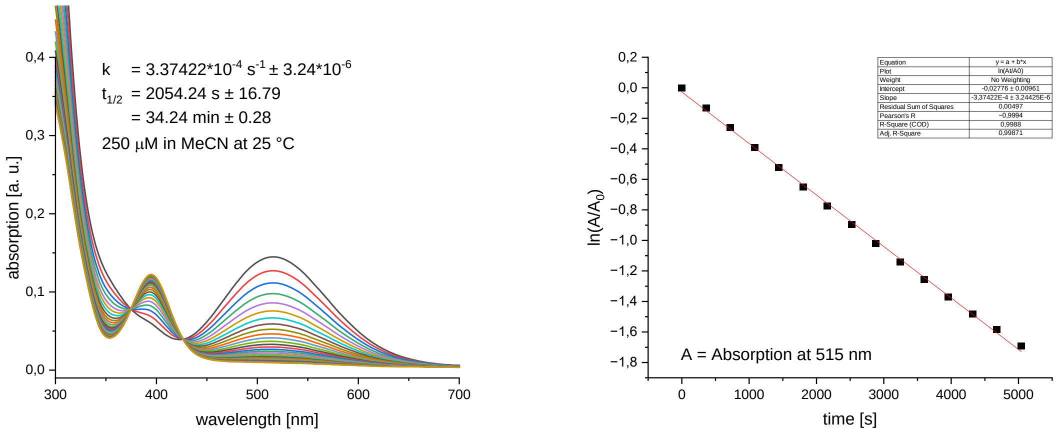


**Figure SIII.8:** UV-vis spectra of the half-life determination of **9** in MeCN at 25 °C (left) with the corresponding first order kinetics plot (right).

## III.5 (*Z*)-1-(3-(2-bromophenyl)dibenzo[*c*,*g*][1,2,5]triazocin-11(12*H*)-yl)ethan-1-one (10)

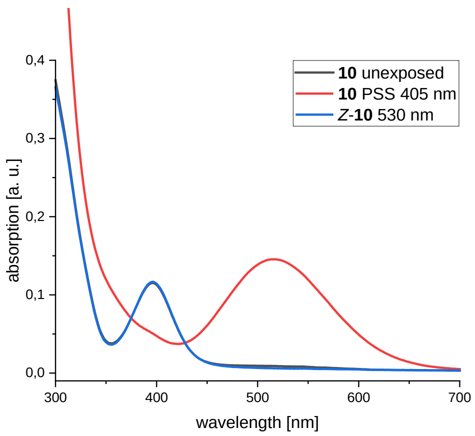


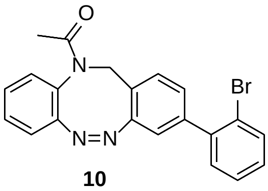


**10**

**Figure SIII.9:** UV-vis spectra of **10** in MeCN at 25 °C. The unexposed sample is plotted in black, the spectra of the PSS between *Z*-**10** and *E*-**10** after irradiation with 405 nm in red and the spectra of *Z*-**10** after irradiation with 530 nm in blue.

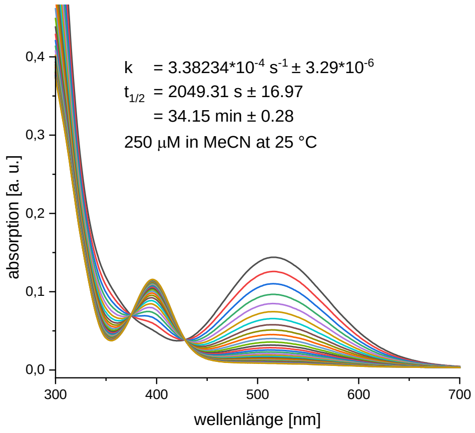


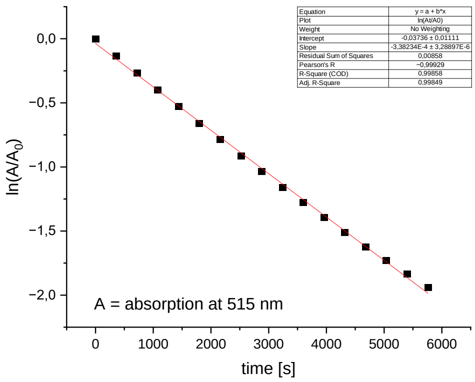


**Figure SIII.10:** UV-vis spectra of the half-life determination of **10** in MeCN at 25 °C (left) with the corresponding first order kinetics plot (right).

## III.6 (*Z*)-1-(3-(4-fluorophenyl)dibenzo[*c*,*g*][1,2,5]triazocin-11(12*H*)-yl)ethan-1-one (11)

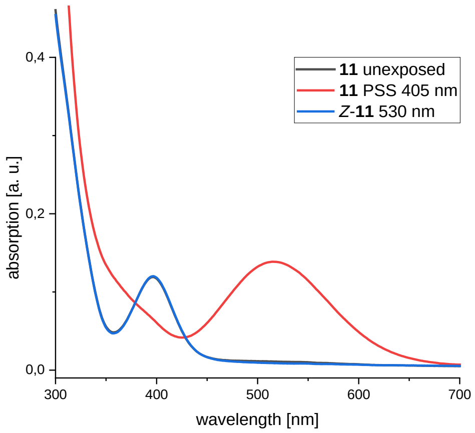


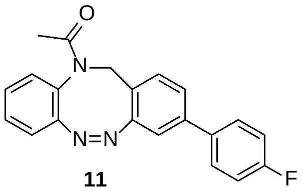


**11**

**Figure SIII.11:** UV-vis spectra of **11** in MeCN at 25 °C. The unexposed sample is plotted in black, the spectra of the PSS between *Z*-**11** and *E*-**11** after irradiation with 405 nm in red and the spectra of *Z*-**11** after irradiation with 530 nm in blue.

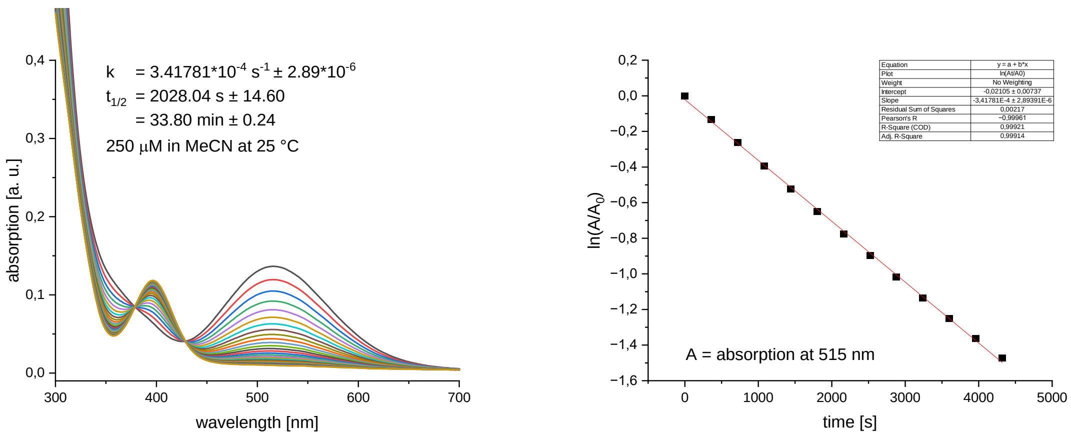


**Figure SIII.12:** UV-vis spectra of the half-life determination of **11** in MeCN at 25 °C (left) with the corresponding first order kinetics plot (right).

## III.7 (*Z*)-2-(11-acetyl-11,12-dihydrodibenzo[*c*,*g*][1,2,5]triazocin-3-yl)benzaldehyde (12)

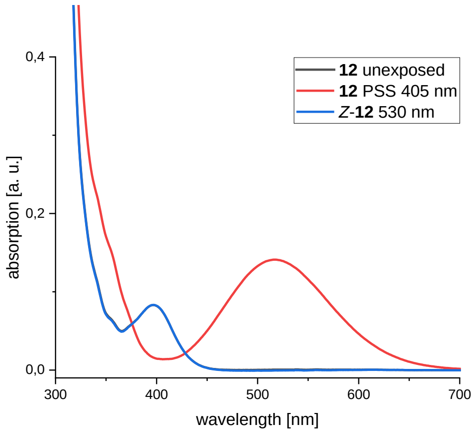


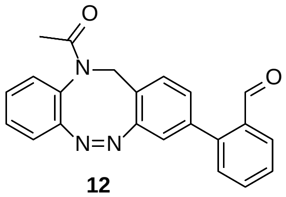

**12**

**Figure SIII.13:** UV-vis spectra of **12** in MeCN at 25 °C. The unexposed sample is plotted in black, the spectra of the PSS between *Z*-**12** and *E*-**12** after irradiation with 405 nm in red and the spectra of *Z*-**12** after irradiation with 530 nm in blue.

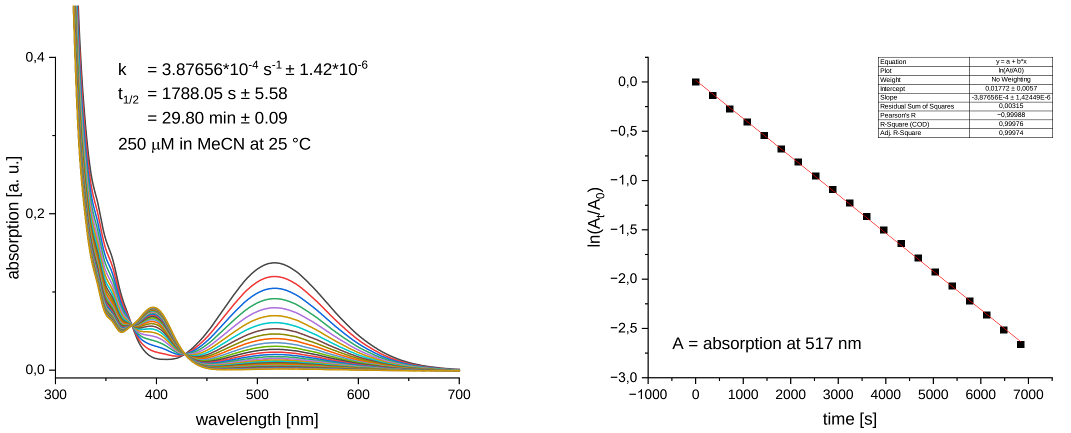


**Figure SIII.14:** UV-vis spectra of the half-life determination of **12** in MeCN at 25 °C (left) with the corresponding first order kinetics plot (right).

## III.8 (*Z*)-2-(11-acetyl-11,12-dihydrodibenzo[*c*,*g*][1,2,5]triazocin-3-yl)benzoic acid (13)

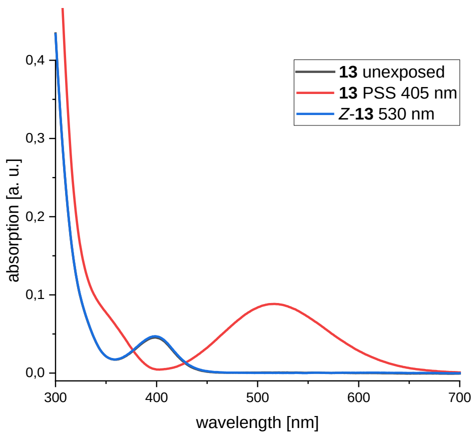


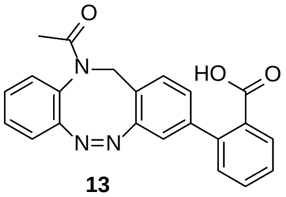


**13**

**Figure SIII.15:** UV-vis spectra of **13** in MeCN at 25 °C. The unexposed sample is plotted in black, the spectra of the PSS between *Z*-**13** and *E*-**13** after irradiation with 405 nm in red and the spectra of *Z*-**13** after irradiation with 530 nm in blue.

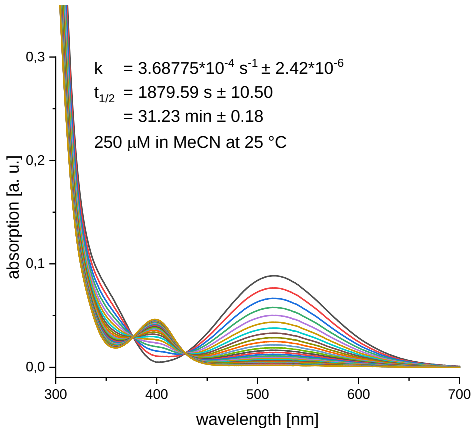


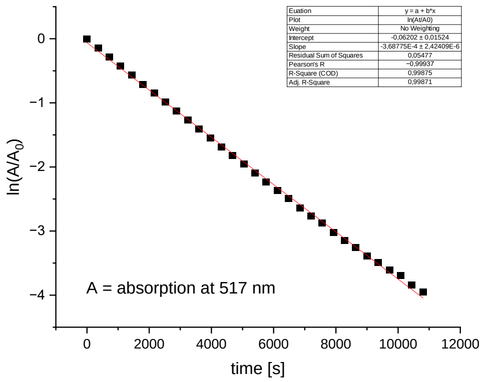


**Figure SIII.16:** UV-vis spectra of the half-life determination of **13** in MeCN at 25 °C (left) with the corresponding first order kinetics plot (right).

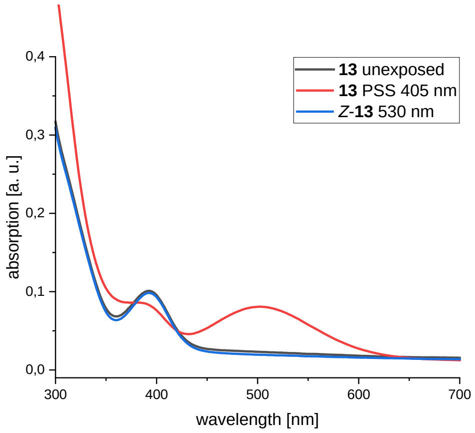


**Figure SIII.17:** UV-vis spectra of **13** in deionized water pH 9 at 25 °C. The unexposed sample is plotted in black, the spectra of the PSS between *Z*-**13** and *E*-**13** after irradiation with 405 nm in red and the spectra of *Z*-**13** after irradiation with 530 nm in blue.

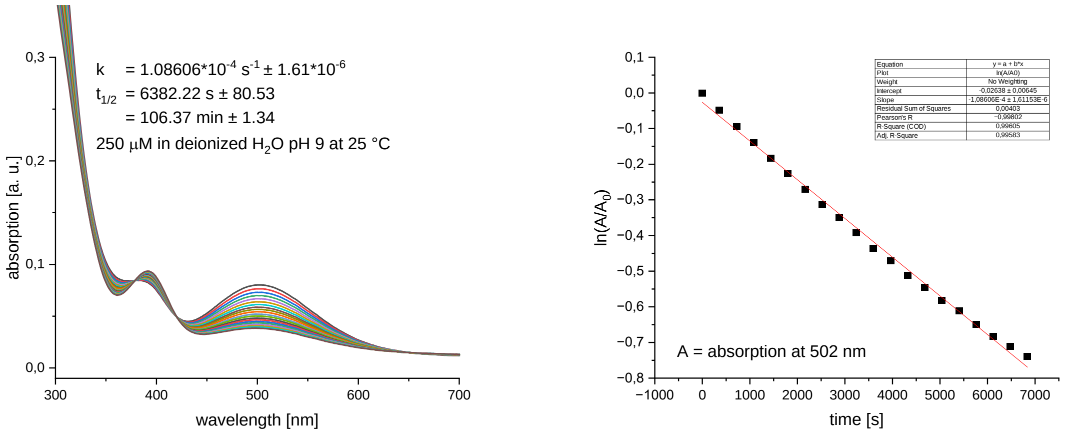


**Figure SIII.18:** UV-vis spectra of the half-life determination of **13** in deionized water pH 9 at 25 °C (left) with the corresponding first order kinetics plot (right).

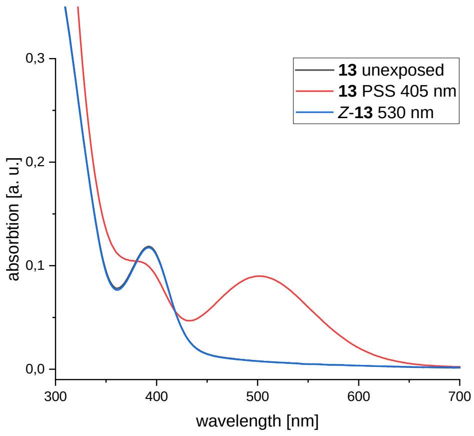


**Figure SIII.19:** UV-vis spectra of **13** in aqueous PBS buffer solution pH 7.4 at 25 °C. The unexposed sample is plotted in black, the spectra of the PSS between *Z*-**13** and *E*-**13** after irradiation with 405 nm in red and the spectra of *Z*-**13** after irradiation with 530 nm in blue.

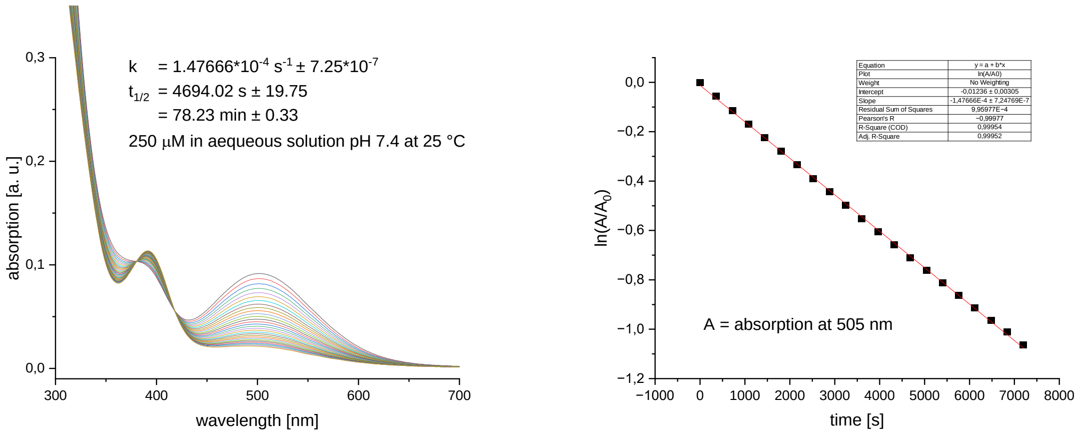


**Figure SIII.20:** UV-vis spectra of the half-life determination of **13** in aqueous PBS buffer solution pH 7.4 at 25 °C (left) with the corresponding first order kinetics plot (right).

## III.9 (*Z*)-1-(3-(pyridine-4-yl)dibenzo[*c*,*g*][1,2,5]triazocin-11(12*H*)-ethan-1-one (14)

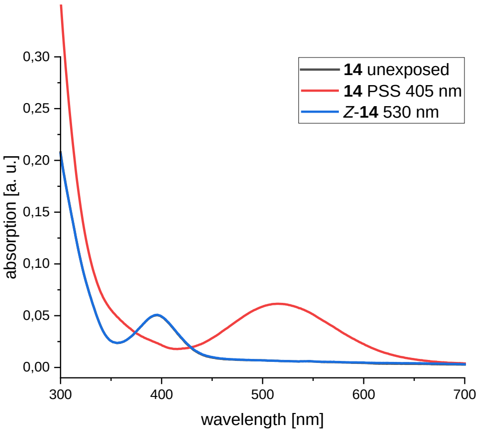


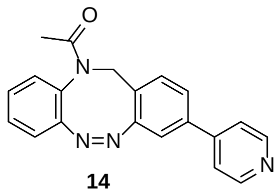


**14**

**Figure SIII.21:** UV-vis spectra of **14** in MeCN at 25 °C. The unexposed sample is plotted in black, the spectra of the PSS between *Z*-**14** and *E*-**14** after irradiation with 405 nm in red and the spectra of *Z*-**14** after irradiation with 530 nm in blue.

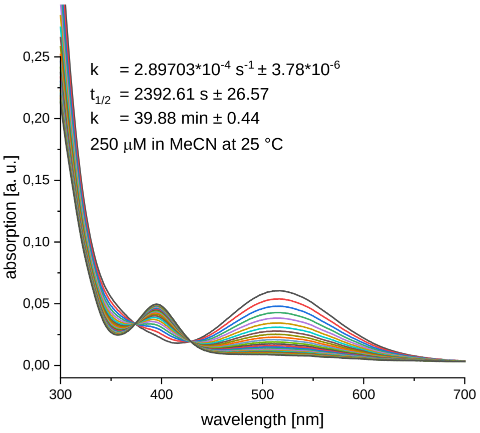


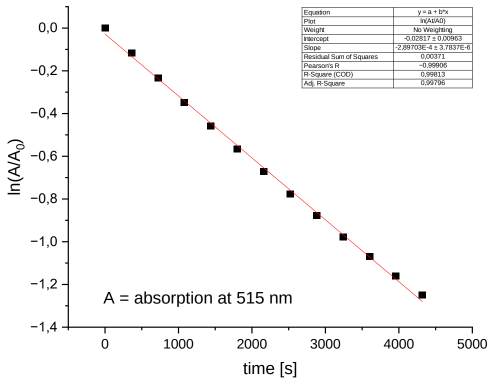


**Figure SIII.22:** UV-vis spectra of the half-life determination of **14** in MeCN at 25 °C (left) with the corresponding first order kinetics plot (right).

## III.10 (*Z*)-1-(3-benzyldibenzo[*c*,*g*][1,2,5]triazocin-11(12*H*)-ethan-1-one (17)

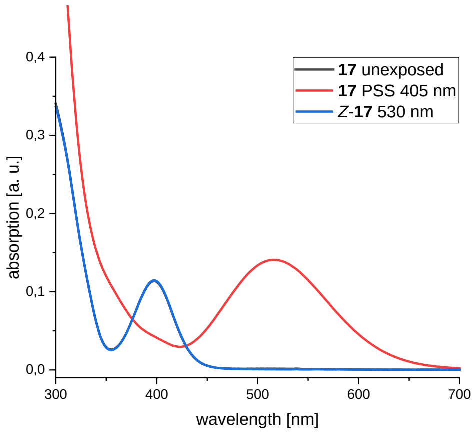


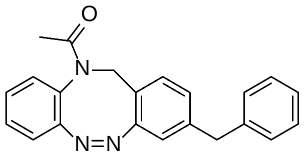


**17**

**Figure SIII.23:** UV-vis spectra of **17** in MeCN at 25 °C. The unexposed sample is plotted in black, the spectra of the PSS between *Z*-**17** and *E*-**17** after irradiation with 405 nm in red and the spectra of *Z*-**17** after irradiation with 530 nm in blue.

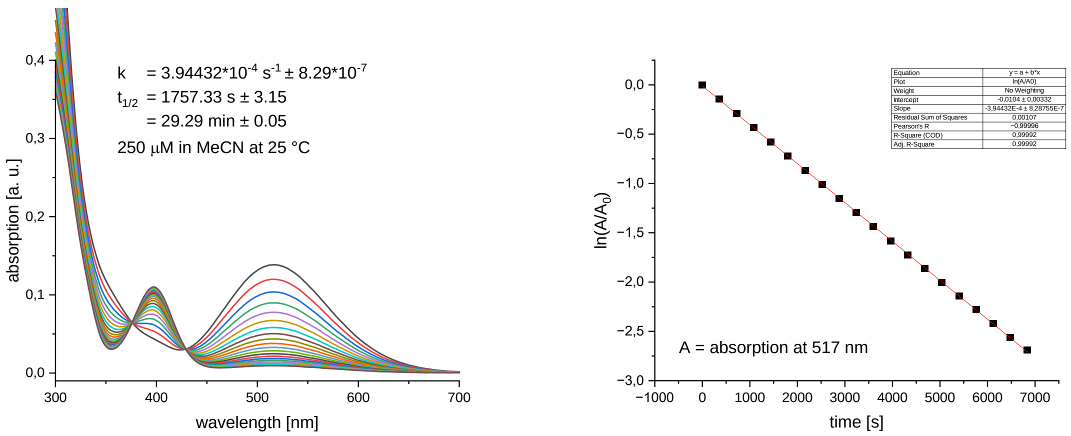


**Figure SIII.24:** UV-vis spectra of the half-life determination of **17** in MeCN at 25 °C (left) with the corresponding first order kinetics plot (right).

## III.11 *tert*-butyl (*Z*)-(11-acetyl-11,12-dihydrodiebenzo[*c*,*g*][1,2,5]triazocin-3-yl)carbamate (19)

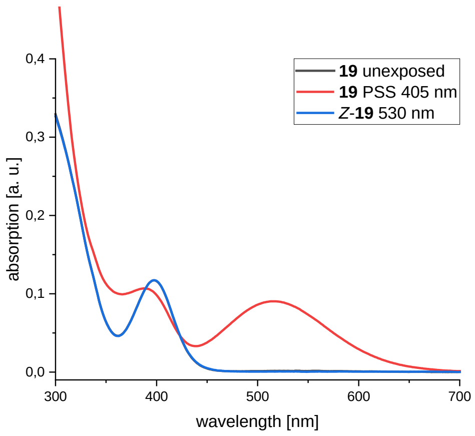


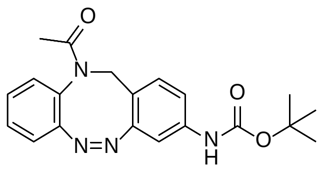


**19**

**Figure SIII.25:** UV-vis spectra of **19** in MeCN at 25 °C. The unexposed sample is plotted in black, the spectra of the PSS between *Z*-**19** and *E*-**19** after irradiation with 405 nm in red and the spectra of *Z*-**19** after irradiation with 530 nm in blue.

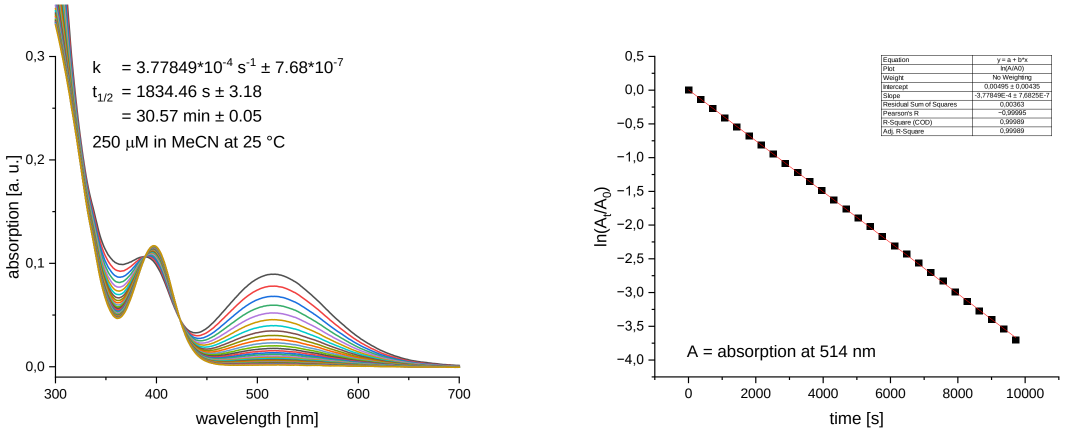


**Figure SIII.26:** UV-vis spectra of the half-life determination of **19** in MeCN at 25 °C (left) with the corresponding first order kinetics plot (right).

## III.12 (*Z*)-1-(3-(diphenylamino)dibenzo[*c,g*][1,2,5]triazocin-11(12*H*)-yl)ethan-1-one (20)

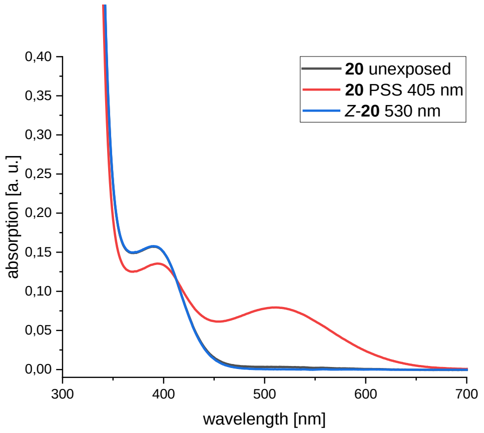


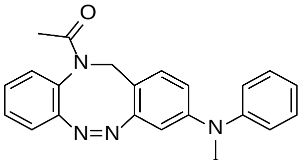


**20**

**Figure SIII.27:** UV-vis spectra of **20** in MeCN at 25 °C. The unexposed sample is plotted in black, the spectra of the PSS between *Z*-**20** and *E*-**20** after irradiation with 405 nm in red and the spectra of *Z*-**20** after irradiation with 530 nm in blue.

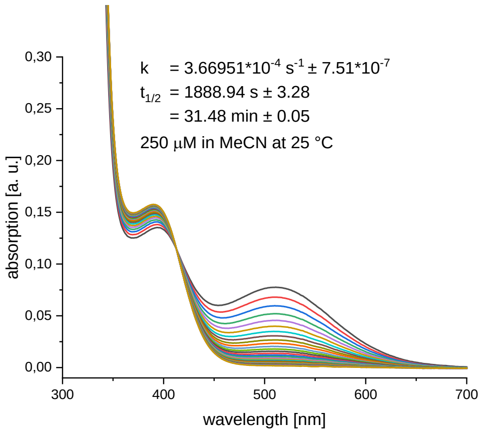


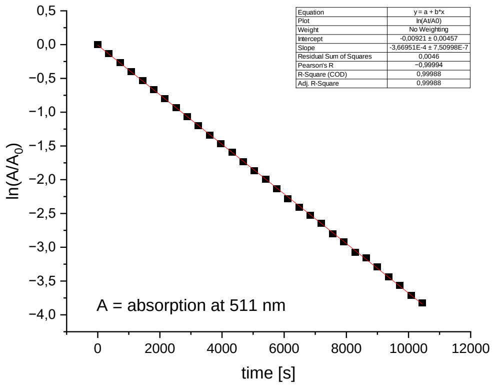


**Figure SIII.28:** UV-vis spectra of the half-life determination of **20** in MeCN at 25 °C (left) with the corresponding first order kinetics plot (right).

## III.13 (*Z*)-1-(3-aminodibenzo[*c,g*][1,2,5]triazocin-11(12*H*)-yl)ethan-1-one (21)

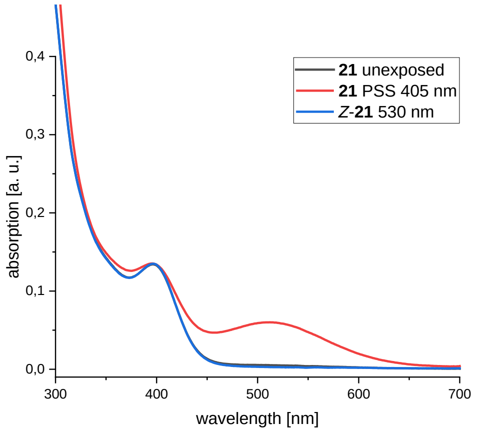


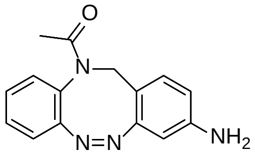


**21**

**Figure SIII.29:** UV-vis spectra of **21** in MeCN at 25 °C. The unexposed sample is plotted in black, the spectra of the PSS between *Z*-**21** and *E*-**21** after irradiation with 405 nm in red and the spectra of *Z*-**21** after irradiation with 530 nm in blue.

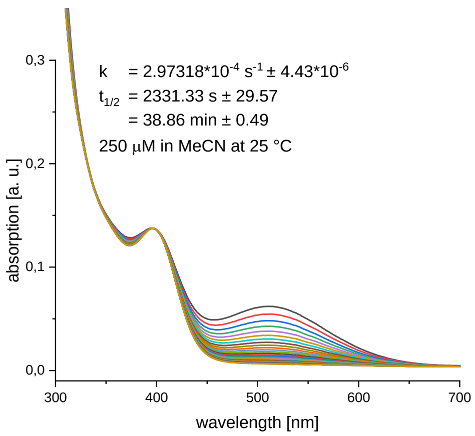


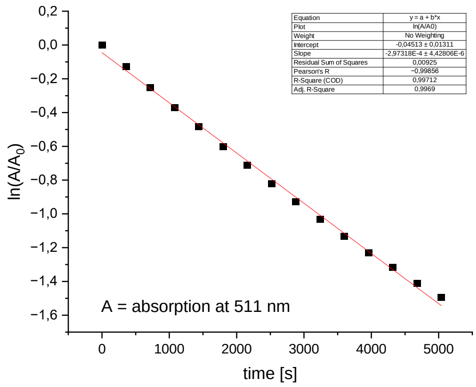


**Figure SIII.30:** UV-vis spectra of the half-life determination of **21** in MeCN at 25 °C (left) with the corresponding first order kinetics plot (right).

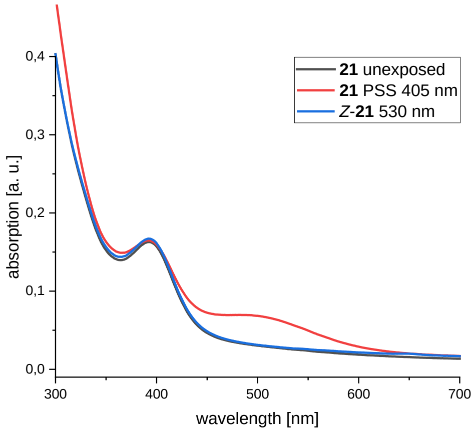


**Figure SIII.31:** UV-vis spectra of **21** in deionized water at 25 °C. The unexposed sample is plotted in black, the spectra of the PSS between *Z*-**21** and *E*-**21** after irradiation with 405 nm in red and the spectra of *Z*-**21** after irradiation with 530 nm in blue.

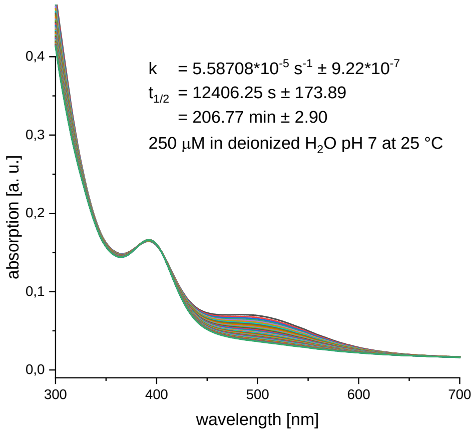


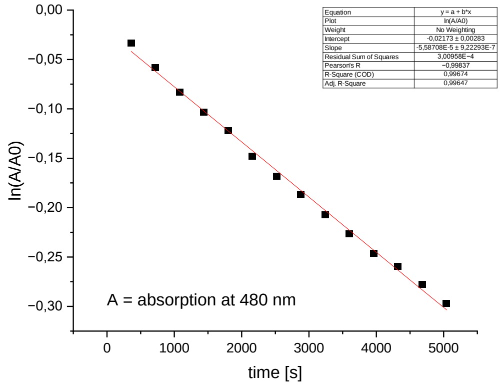


**Figure SIII.32:** UV-vis spectra of the half-life determination of **21** in deionized water at 25 °C (left) with the corresponding first order kinetics plot (right).

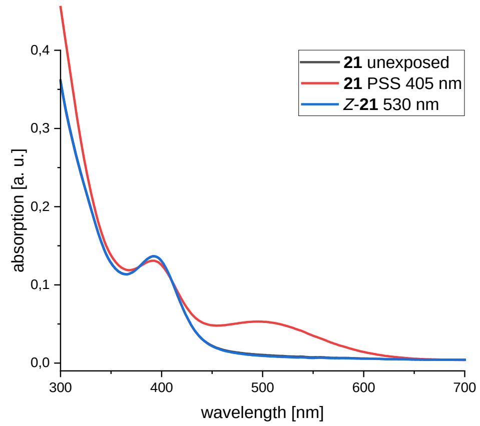


**Figure SIII.33:** UV-vis spectra of **21** in deionized water pH 3.5 at 25 °C. The unexposed sample is plotted in black, the spectra of the PSS between *Z*-**21** and *E*-**21** after irradiation with 405 nm in red and the spectra of *Z*-**21** after irradiation with 530 nm in blue.

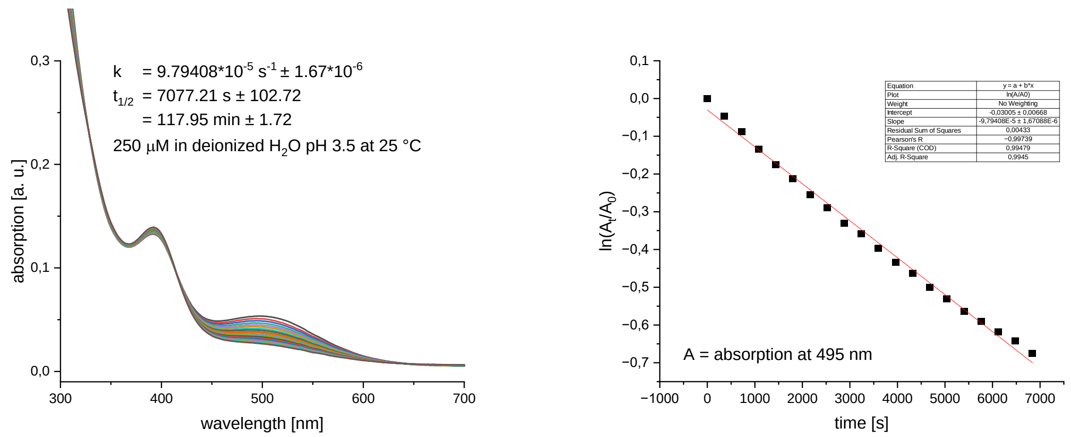


**Figure SIII.34:** UV-vis spectra of the half-life determination of **21** in deionized water pH 3.5 at 25 °C (left) with the corresponding first order kinetics plot (right).

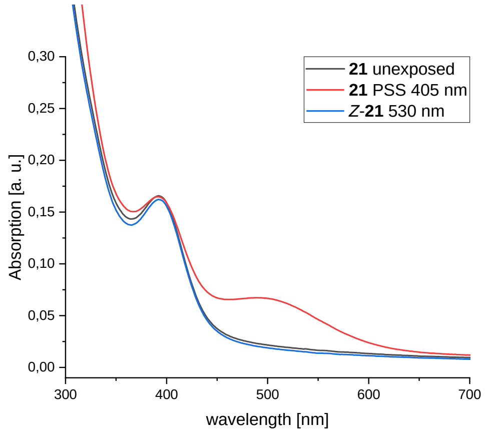


**Figure SIII.35:** UV-vis spectra of **21** in aqueous PBS buffer solution pH 7.4 at 25 °C. The unexposed sample is plotted in black, the spectra of the PSS between *Z*-**21** and *E*-**21** after irradiation with 405 nm in red and the spectra of *Z*-**21** after irradiation with 530 nm in blue.

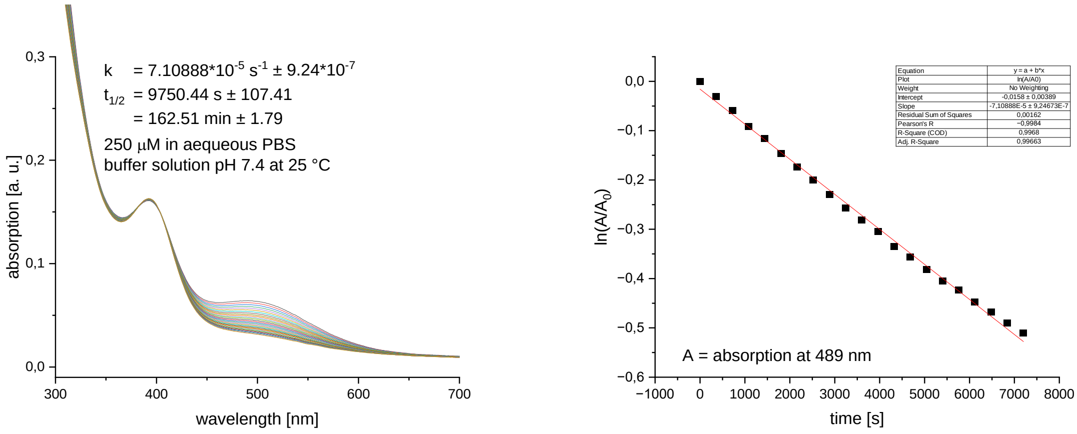


**Figure SIII.36:** UV-vis spectra of the half-life determination of **21** in aqueous PBS buffer solution pH 7.4 at 25 °C (left) with the corresponding first order kinetics plot (right).

## III.14 (*Z*)-11-acetyl-11,12-dihydrodibenzo[*c*,*g*][1,2,5]triazocine-3-carbonitrile (23)

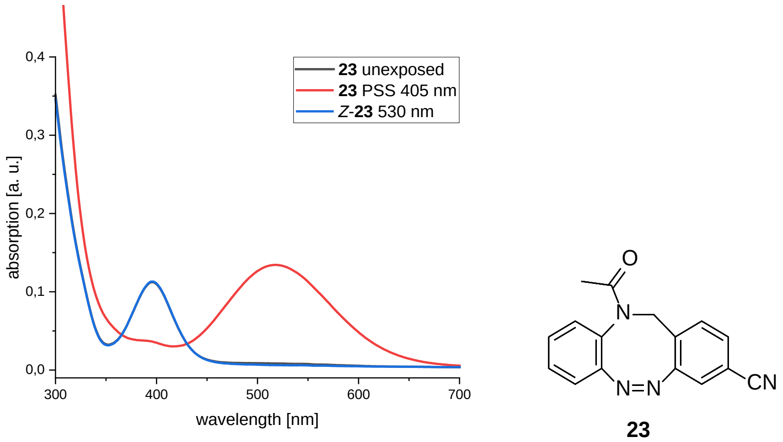


**23**

**Figure SIII.37:** UV-vis spectra of **23** in MeCN at 25 °C. The unexposed sample is plotted in black, the spectra of the PSS between *Z*-**23** and *E*-**23** after irradiation with 405 nm in red and the spectra of *Z*-**23** after irradiation with 530 nm in blue.

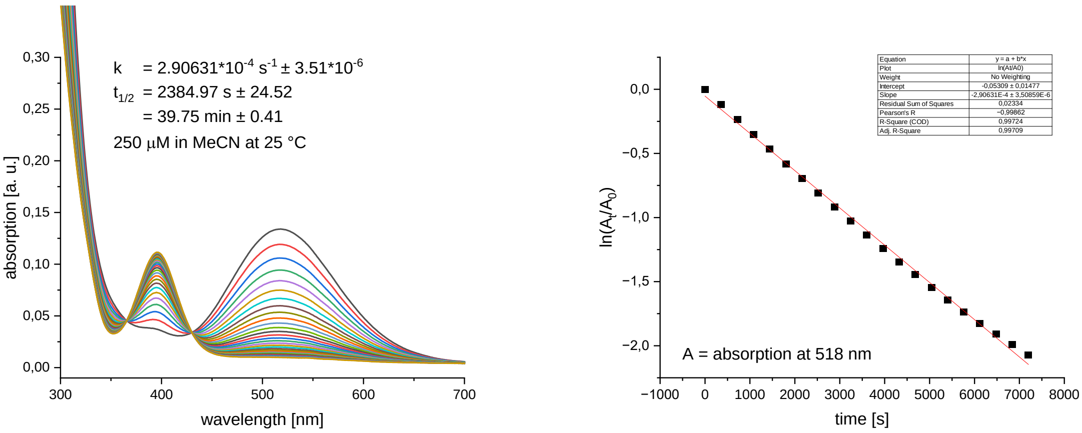


**Figure SIII.38:** UV-vis spectra of the half-life determination of **23** in MeCN at 25 °C (left) with the corresponding first order kinetics plot (right).

# IV. $^{1}$H-NMR switching experiments

Photostationary states (PSS) of compounds **4,7-14,17,19-21** and **23** were measured via $^1$H-NMR by integration of the different species after irradiation with 405 nm, whereby no distinction was made between *E-twist*- and *E-chair*-conformations. The value of the integrals and the extrapolated values are given in Table SIII.1. $^1$H-NMR spectra were measured at 5 mM concentrations, except pyridine-substituted *N*-acetyl-diazocine **14** (2.55 mM due to low synthesis yield). The following equation (2) was used for extrapolation of the photostationary states:

$$PSS_{extrapol.} = PSS_{measured} \cdot e^{kt} \quad (2)$$

*k* is the rate constant at 25 °C (see table 6 and 7) and *t* the measuring time (330 s): 120 s preparation (transfer into the spectrometer and shimming after excitation with light) and 210 s measuring time for 32 scans. All spectra were measured at a Bruker AV600 spectrometer.

**Table SIV.1:** Measured and extrapolated photostationary states of compounds **4,7-14,17,19-21** and **23** in acetonitrile.

| | measured PSS % | extrapolated PSS % |
|---|---|---|
| **4** | 68 | 85 |
| **7** | 69 | 79 |
| **8** | 67 | 76 |
| **9** | 69 | 77 |
| **10** | 73 | 82 |
| **11** | 70 | 78 |
| **12** | 71 | 80 |
| **13** | 8 | 77 |
| **14** | 72 | 79 |
| **17** | 72 | 82 |
| **19** | 54 | 61 |
| **20** | 43 | 49 |
| **21** | 37 | 41 |
| **23** | 76 | 83 |

**Table SIV.2:** Measured and extrapolated photostationary states of compounds **13** and **23** in water.

| | measured PSS % | extrapolated PSS % |
|---|---|---|
| **13** pH 7.4 | 51 | 53 |
| **13** pH 9 | 46 | 48 |
| **21** pH 7.4 | 35 | 37 |
| **21** pH 3.5 | 62 | 62 |

## IV.1 (Z)-(3,8-dibromodibenzo[*c,g*][1,2,5]triazocin-11(12H)-yl)ethan-1-one (4)

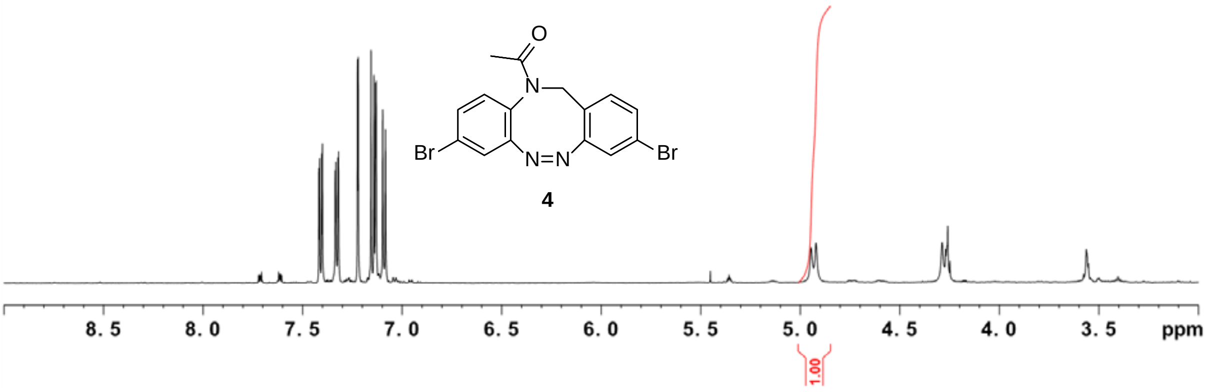


**Figure SIV.1:** $^1$H-NMR-spectra of *Z*-**4** in $CD_3CN$ at 298 K.

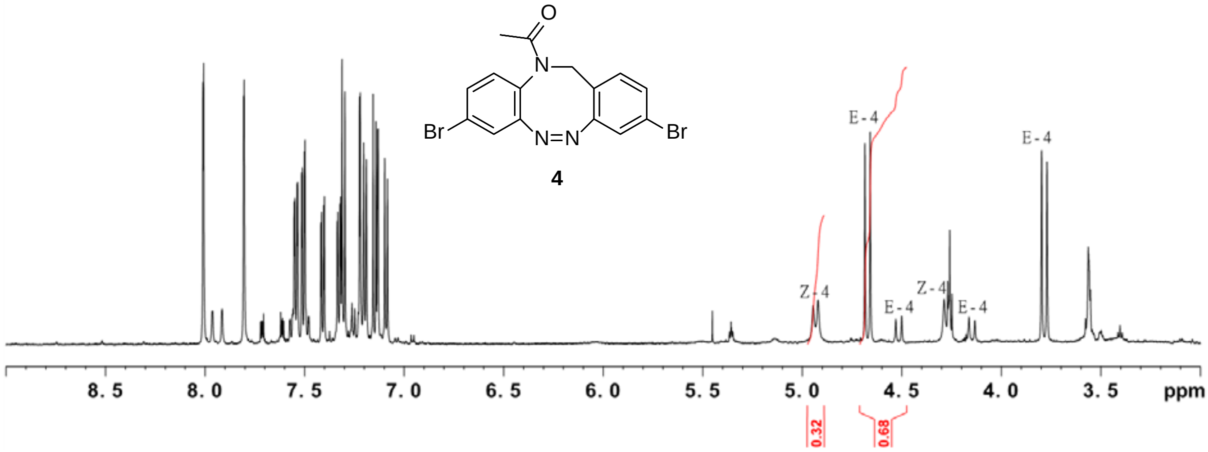


**Figure SIV.2:** $^1$H-NMR-spectra of **4** in $CD_3CN$ at 298 K after irradiation with 405 nm.

## IV.2 (*Z*)-1-(3-phenyldibenzo[*c,g*][1,2,5]triazocin-11(12*H*)-yl)ethan-1-one (7)

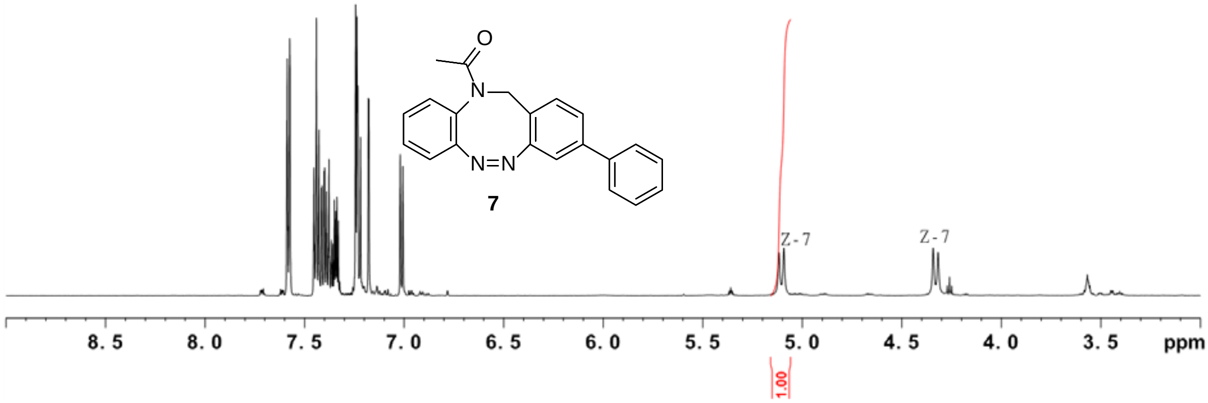


**Figure SIV.3:** $^1$H-NMR-spectra of *Z*-**7** in $CD_3CN$ at 298 K.

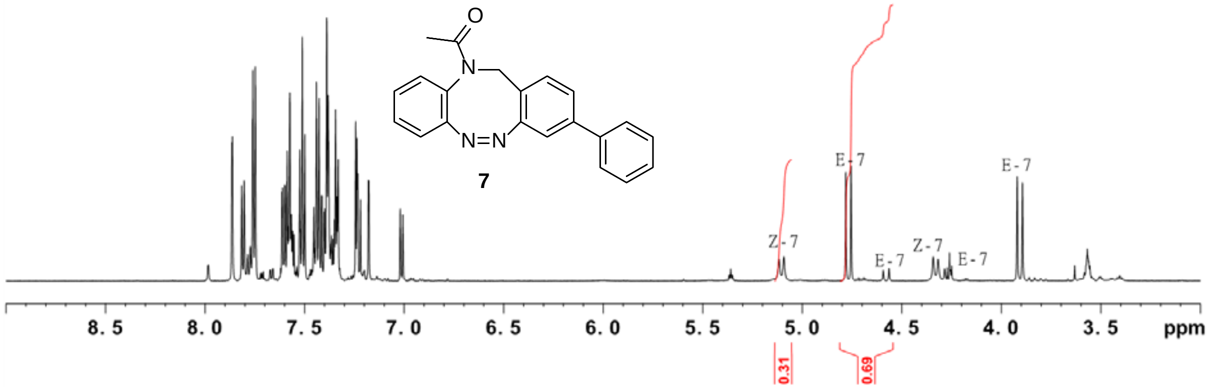


**Figure SIV.4:** $^1$H-NMR-spectra of **7** in $CD_3CN$ at 298 K after irradiation with 405 nm.

## IV.3 (*Z*)-1-(3-vinyldibenzo[*c,g*][1,2,5]triazocin-11(12*H*)-yl)ethan-1-one (8)

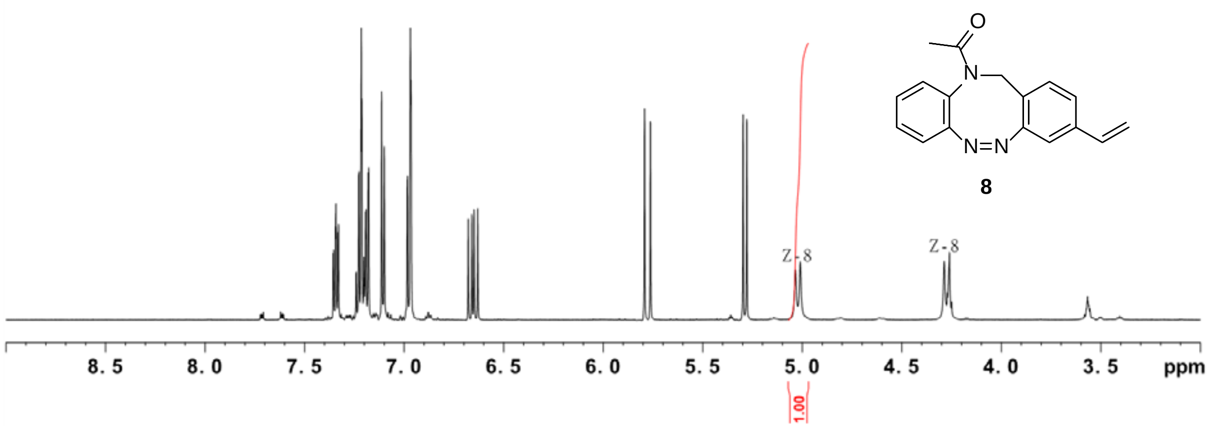


**Figure SIV.5:** $^{1}$H-NMR-spectra of *Z*-**8** in $CD_3CN$ at 298 K.

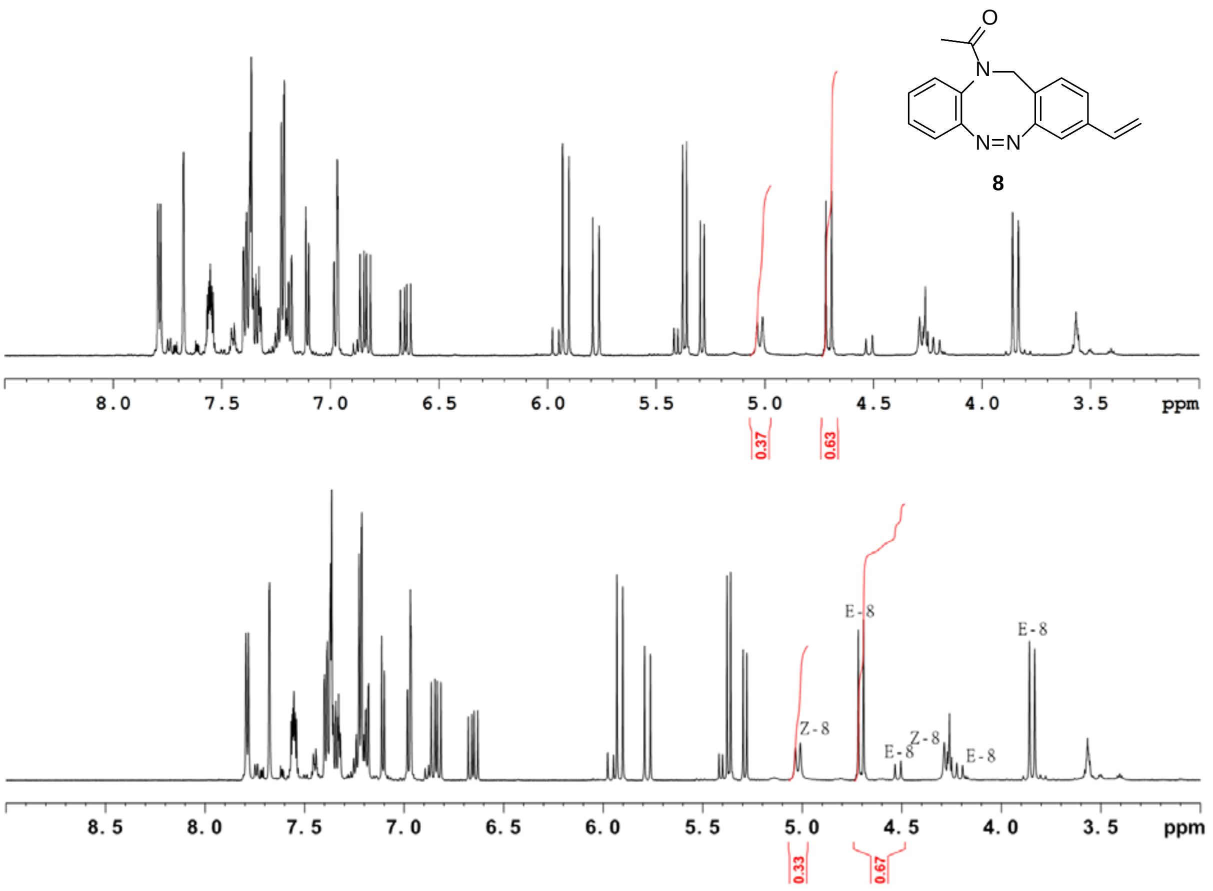


**Figure SIV.6:** $^{1}$H-NMR-spectra of **8** in $CD_3CN$ at 298 K after irradiation with 405 nm.

## IV.4 (*Z*)-1-(3-(2,6-dimethylphenyl)dibenzo[*c,g*][1,2,5]triazocin-11(12*H*)-yl)ethan-1-one (9)

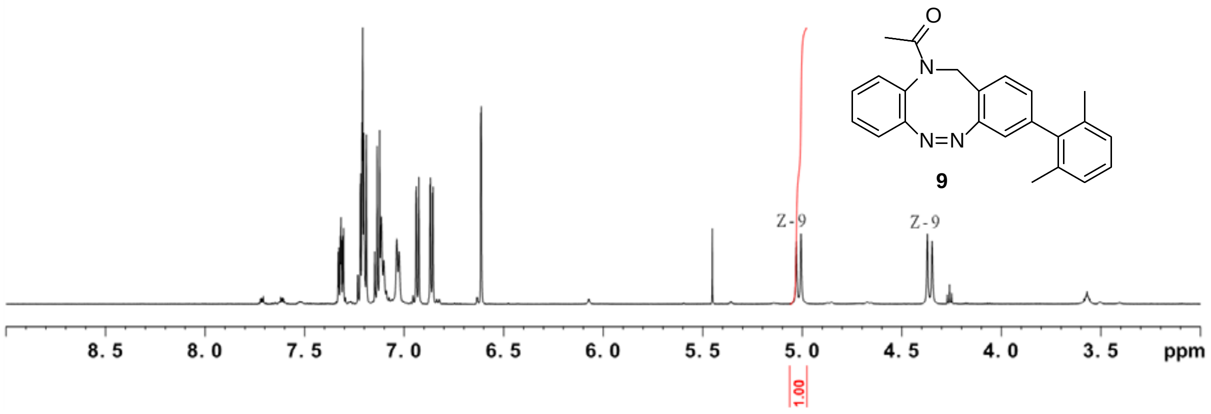


**Figure SIV.7:** $^{1}$H-NMR-spectra of *Z*-**9** in $CD_3CN$ at 298 K.

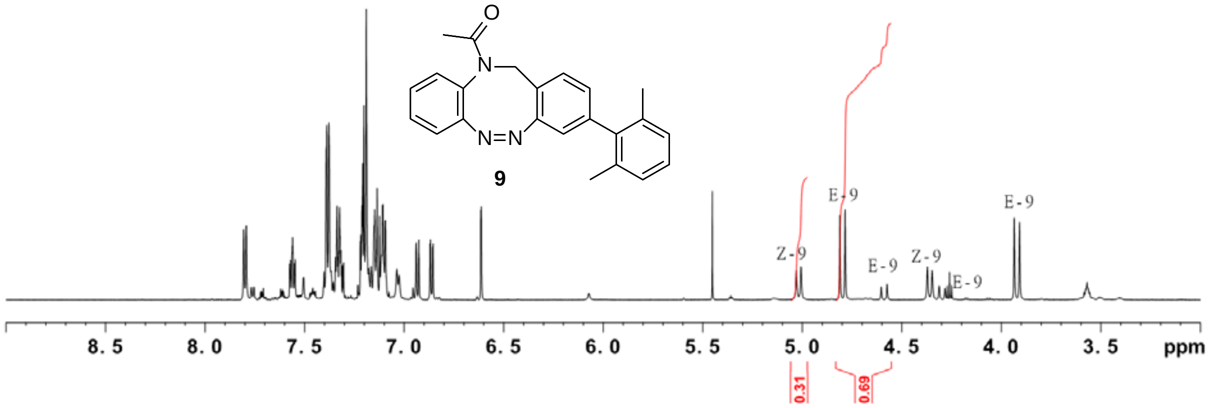


**Figure SIV.8:** $^{1}$H-NMR-spectra of **9** in $CD_3CN$ at 298 K after irradiation with 405 nm.

## IV.5 (*Z*)-1-(3-(2-bromophenyl)dibenzo[*c,g*][1,2,5]triazocin-11(12*H*)-yl)ethan-1-one (10)

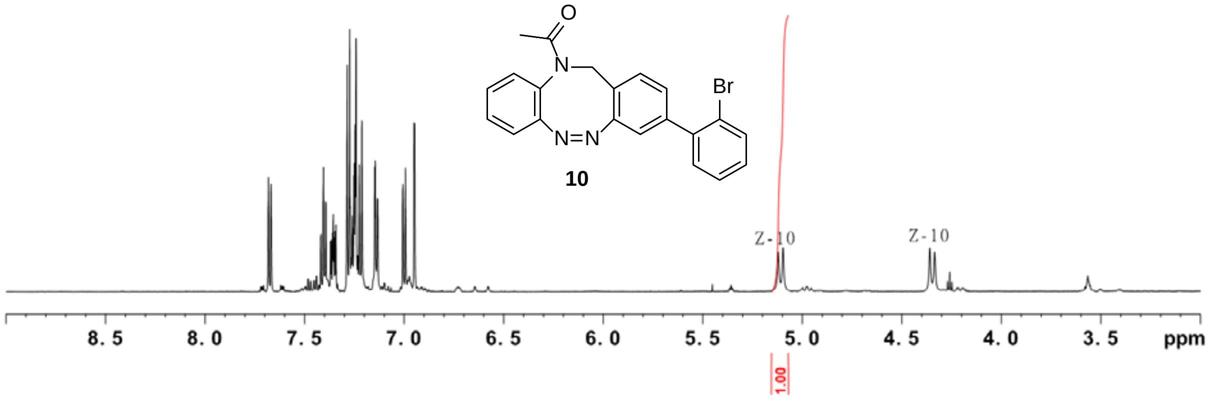


**Figure SIV.9:** $^{1}$H-NMR-spectra of *Z*-**10** in $CD_3CN$ at 298 K.

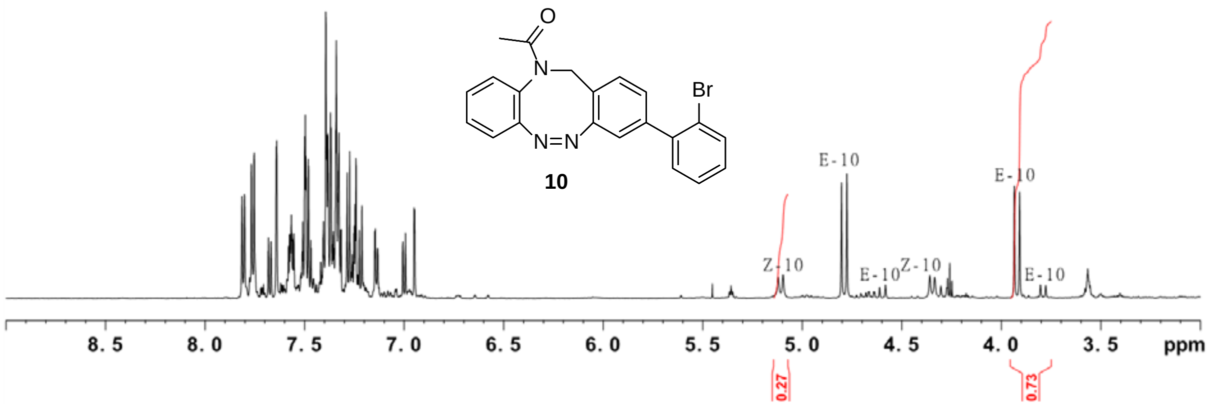


**Figure SIV.10:** $^{1}$H-NMR-spectra of **10** in $CD_3CN$ at 298 K after irradiation with 405 nm.

## IV.6 (*Z*)-1-(3-(4-fluorophenyl)dibenzo[*c,g*][1,2,5]triazocin-11(12*H*)-yl)ethan-1-one (11)

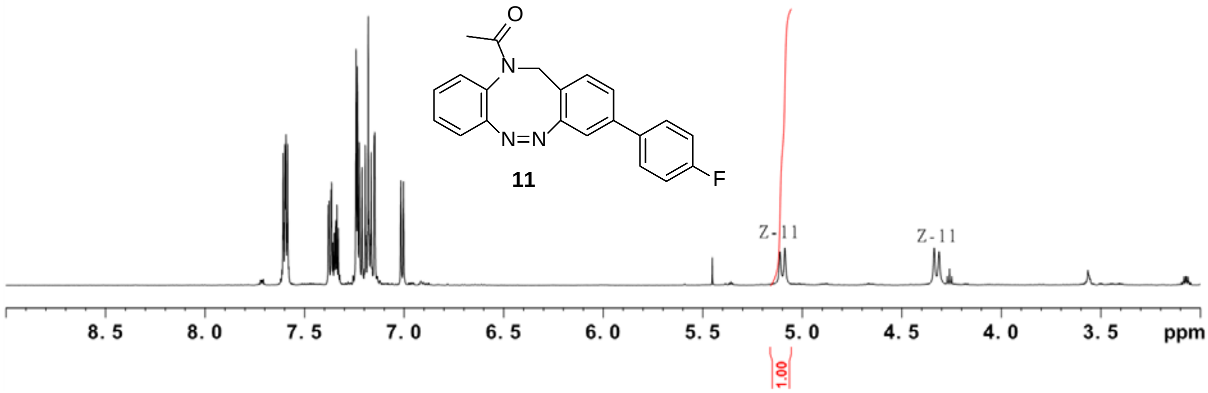


**Figure SIV.11:** $^{1}$H-NMR-spectra of *Z*-**11** in $CD_3CN$ at 298 K.

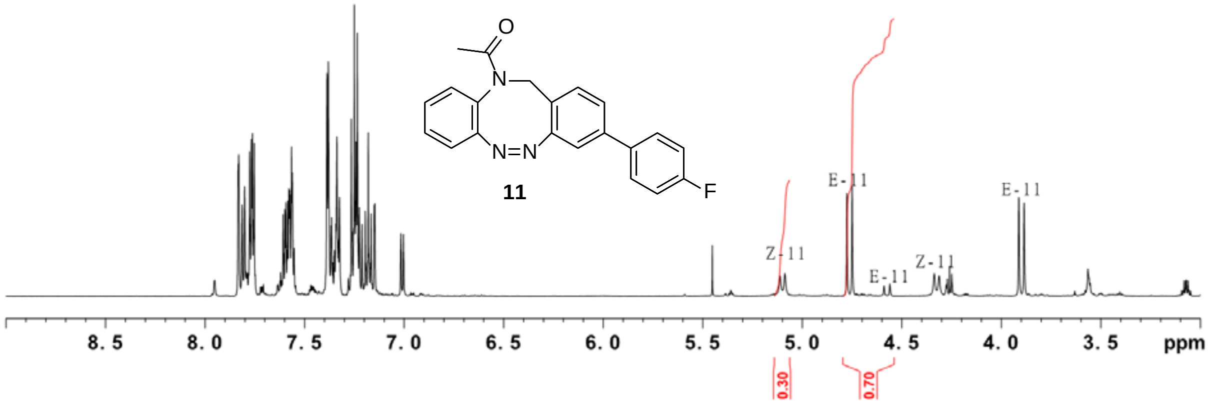


**Figure SIV.12:** $^{1}$H-NMR-spectra of **11** in $CD_3CN$ at 298 K after irradiation with 405 nm.

## IV.7 (*Z*)-2-(11-acetyl-11,12-dihydrodibenzo[*c,g*][1,2,5]triazocin-3-yl)benzaldehyde (12)

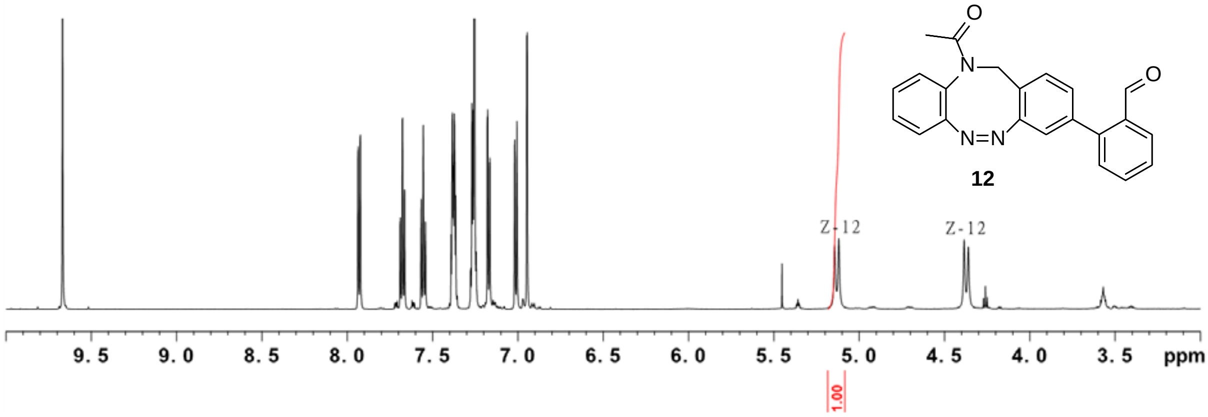


**Figure SIV.13:** $^1$H-NMR-spectra of *Z*-**12** in $CD_3CN$ at 298 K.

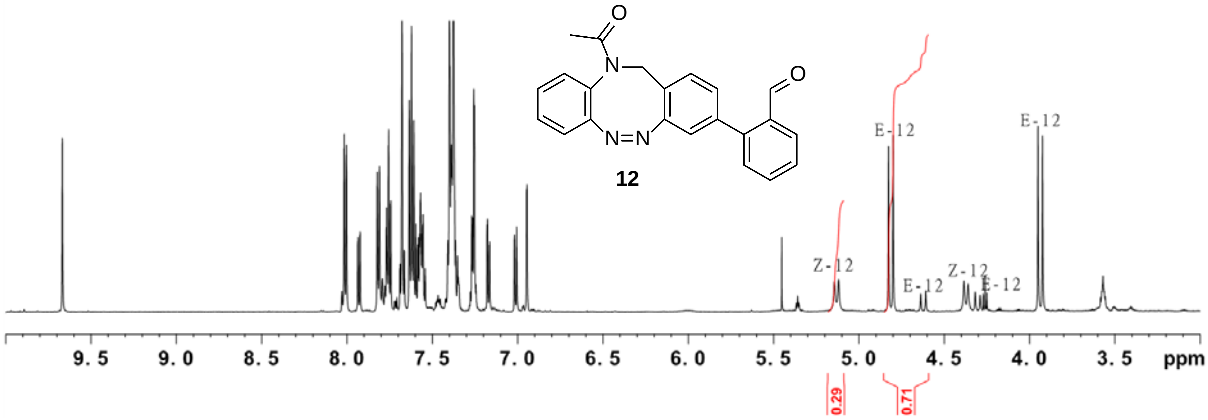


**Figure SIV.14:** $^1$H-NMR-spectra of **12** in $CD_3CN$ at 298 K after irradiation with 405 nm.

## IV.8 (*Z*)-2-(11-acetyl-11,12-dihydrodibenzo[*c,g*][1,2,5]triazocin-3-yl)benzoic acid (13)

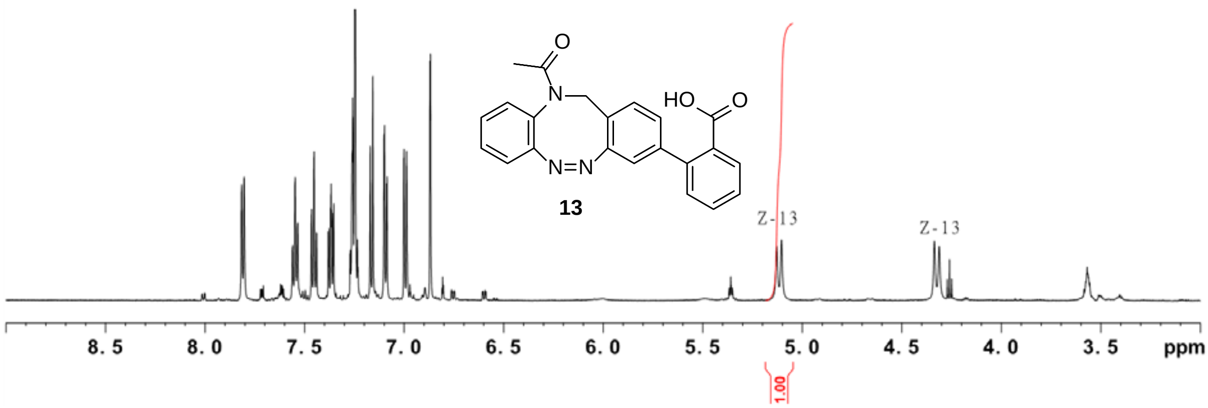


**Figure SIV.15:** $^1$H-NMR-spectra of *Z*-**13** in $CD_3CN$ at 298 K.

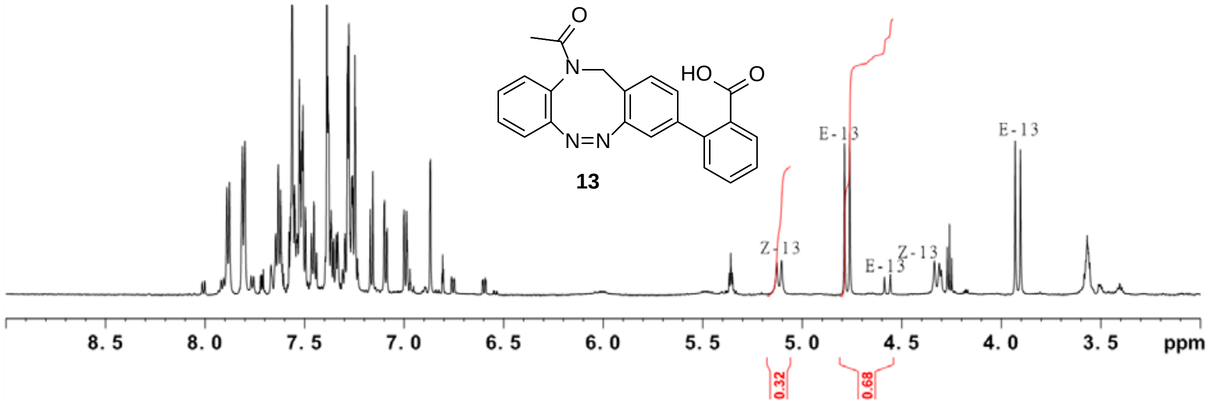


**Figure SIV.16:** $^{1}$H-NMR-spectra of **13** in $CD_3CN$ at 298 K after irradiation with 405 nm.

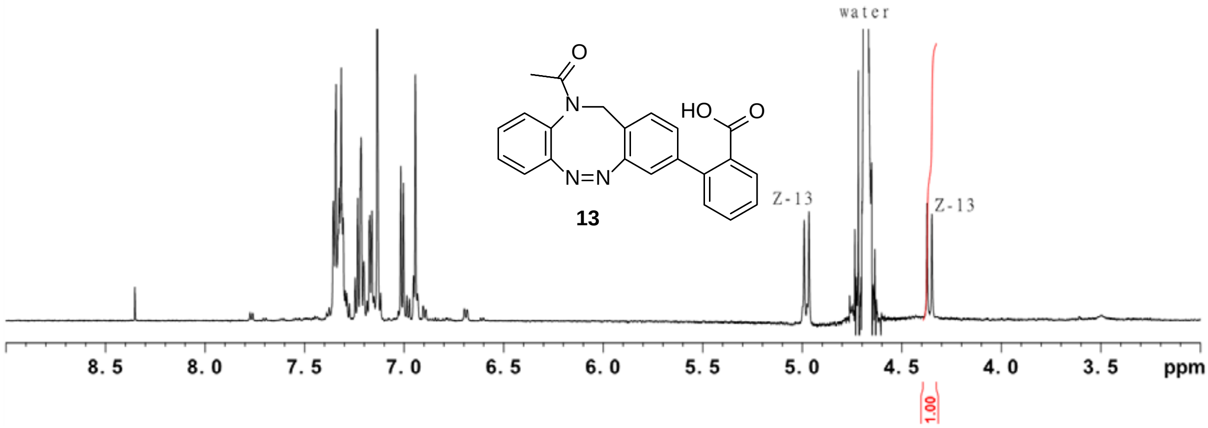


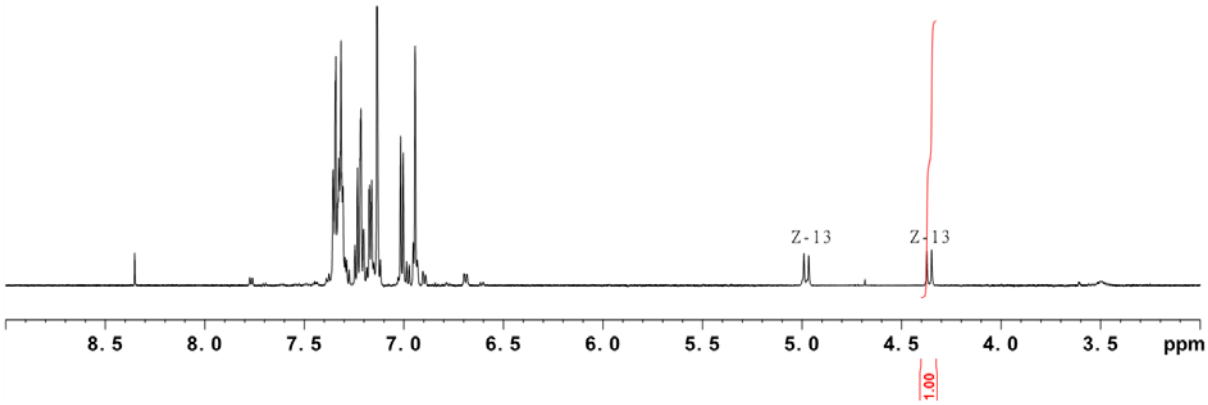


**Figure SIV.17:** $^{1}$H-NMR-spectra of *Z*-**13** in $D_2O$ pH 9 at 298 K (top) and the $^{1}$H-NMR-Spektrum of **13** in $D_2O$ pH 9 at 298 K with baseline correction and water suppression performed with MestReNova 14.3.1 NMR analysis Software by Mestrelab (bottom).

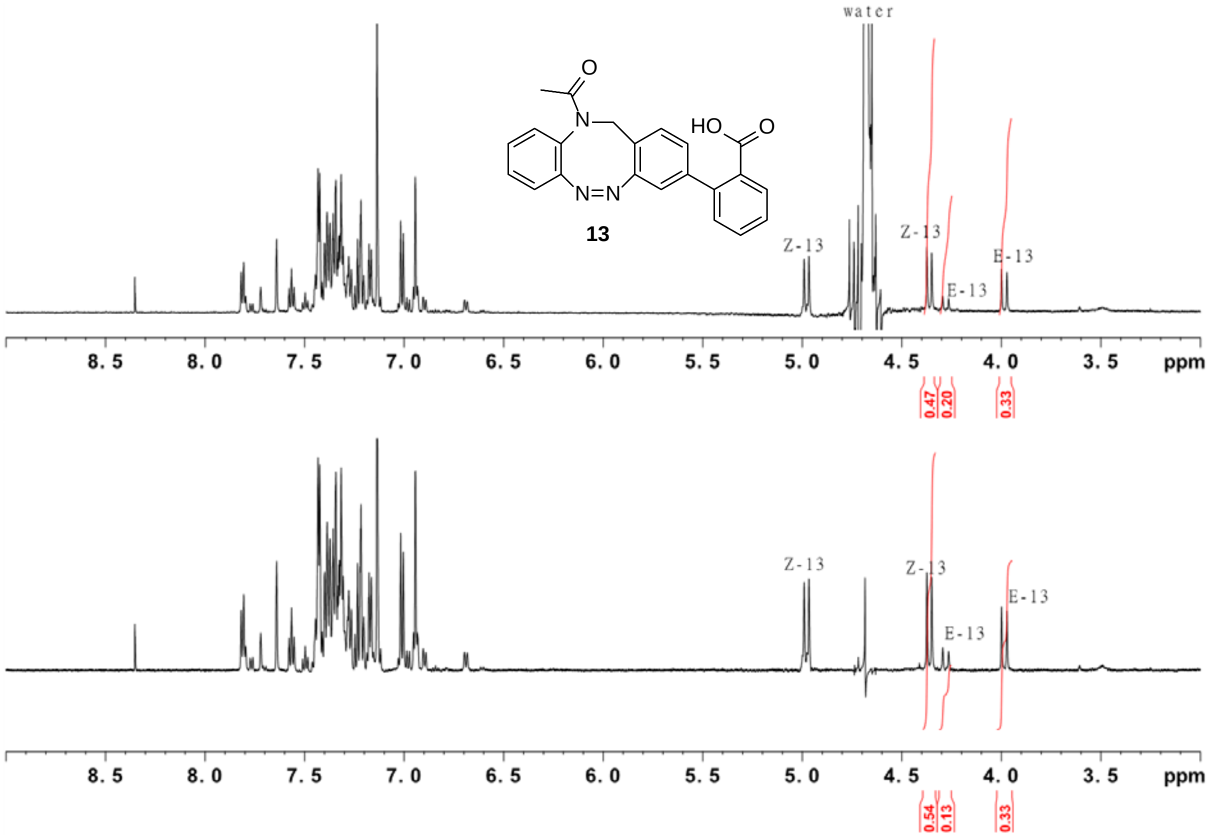


**Figure SIV.18:** $^{1}$H-NMR-spectra of **13** in $D_2O$ pH 9 at 298 K after irradiation with 405 nm (top) and the $^{1}$H-NMR-Spektrum of **13** in $D_2O$ pH 9 at 298 K after irradiation with 405 nm with baseline correction and water suppression performed with MestReNova 14.3.1 NMR analysis Software by Mestrelab (bottom)..

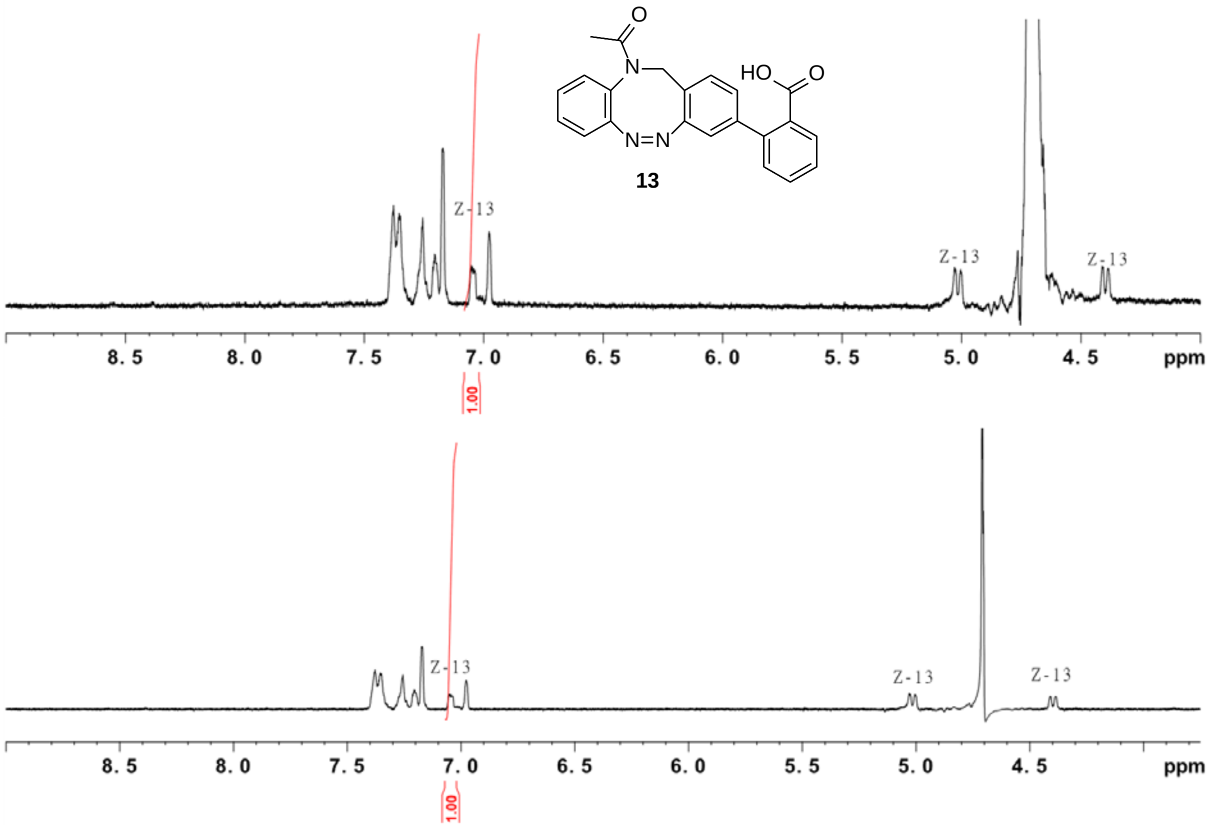


**Figure SIV.19:** $^{1}$H-NMR-spectra of **13** in $D_2O$ PBS buffer solution pH 7.4 at 298 K (top) and the $^{1}$H-NMR-Spektrum of **13** in $D_2O$ PBS buffer solution pH 7.4 at 298 K with baseline correction and water suppression performed with MestReNova 14.3.1 NMR analysis Software by Mestrelab (bottom).

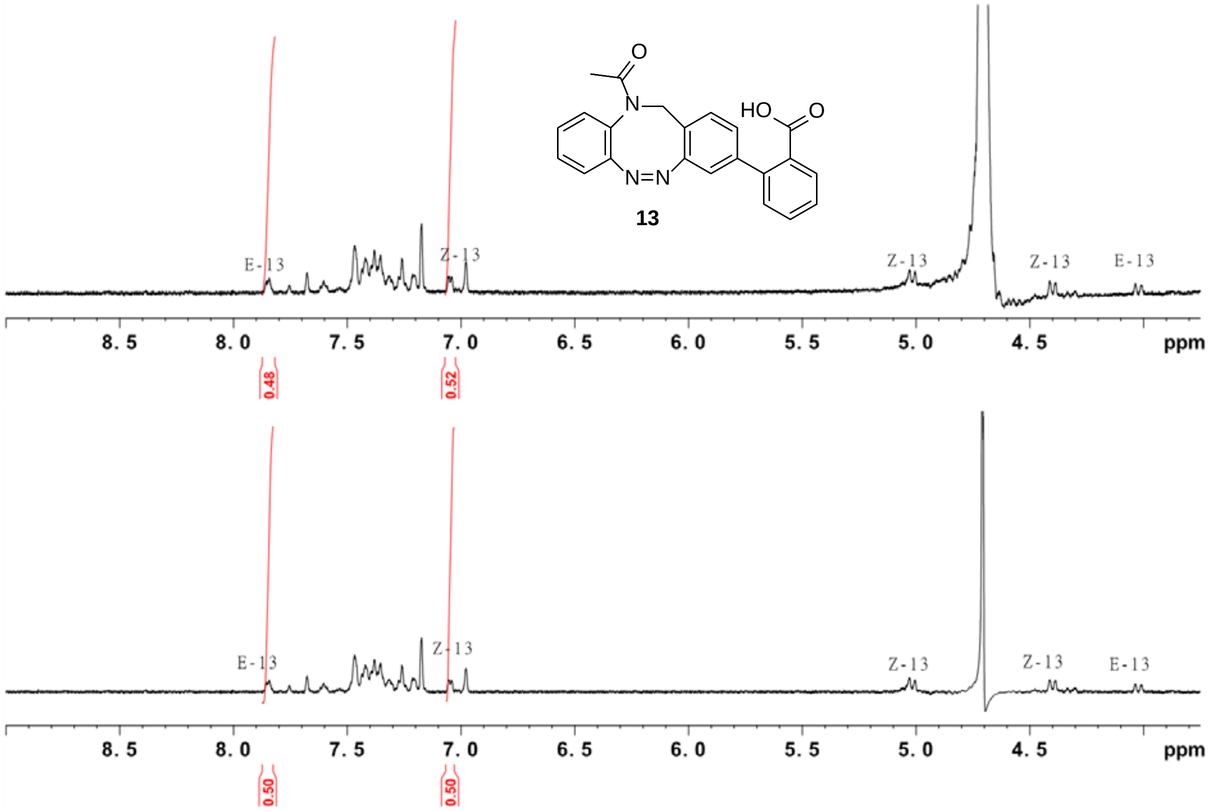


**Figure SIV.20:** $^{1}$H-NMR-spectra of **13** in $D_2O$ PBS buffer solution pH 7.4 at 298 K after irradiation with 405 nm (top) and the $^{1}$H-NMR-Spektrum of **13** in $D_2O$ PBS buffer solution pH 7.4 at 298 K after irradiation with 405 nm with

baseline correction and water suppression performed with MestReNova 14.3.1 NMR analysis Software by Mestrelab (bottom).

## IV.9 (*Z*)-1-(3-(pyridine-4-yl)dibenzo[*c*,*g*][1,2,5]triazocin-11(12*H*)-ethan-1-one (14)

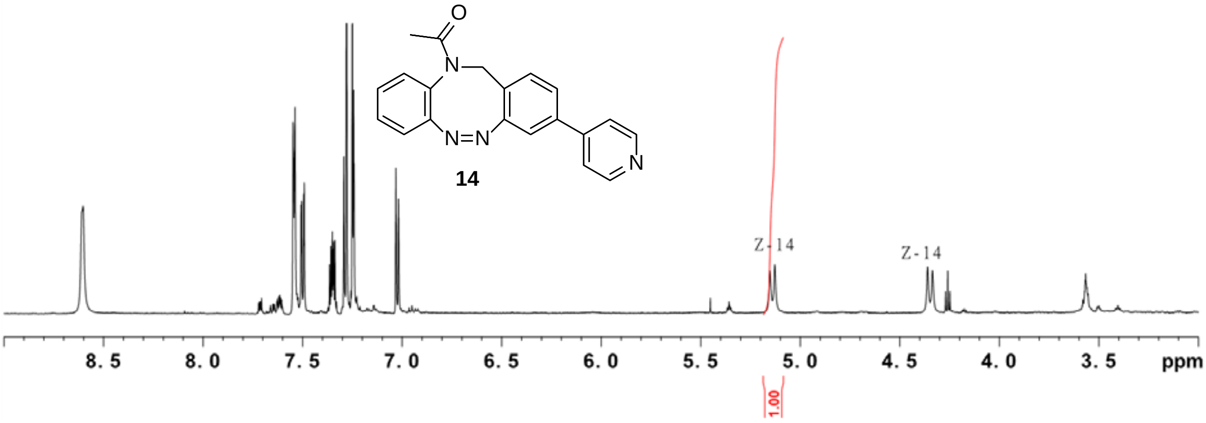


**Figure SIV.21:** $^1$H-NMR-spectra of **14** in $CD_3CN$ at 298 K.

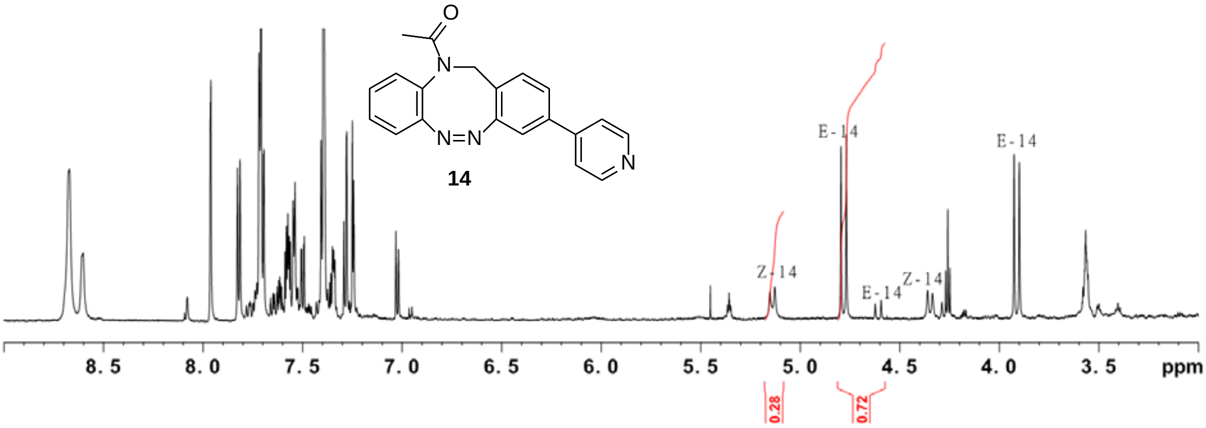


**Figure SIV.22:** $^1$H-NMR-spectra of **14** in $CD_3CN$ at 298 K after irradiation with 405 nm.

## IV.10 (*Z*)-1-(3-benzyldibenzo[*c*,*g*][1,2,5]triazocin-11(12*H*)-ethan-1-one (17)

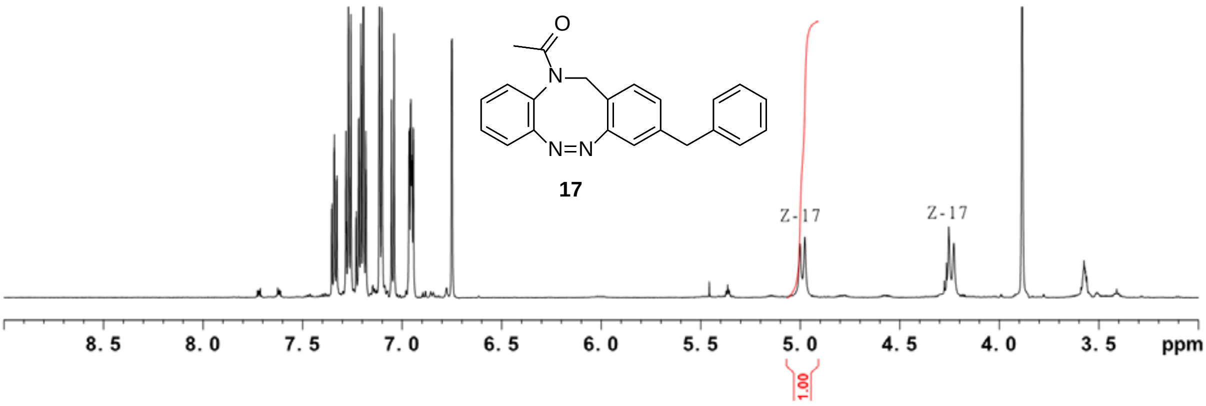


**Figure SIV.23:** $^1$H-NMR-spectra of **17** in $CD_3CN$ at 298 K.

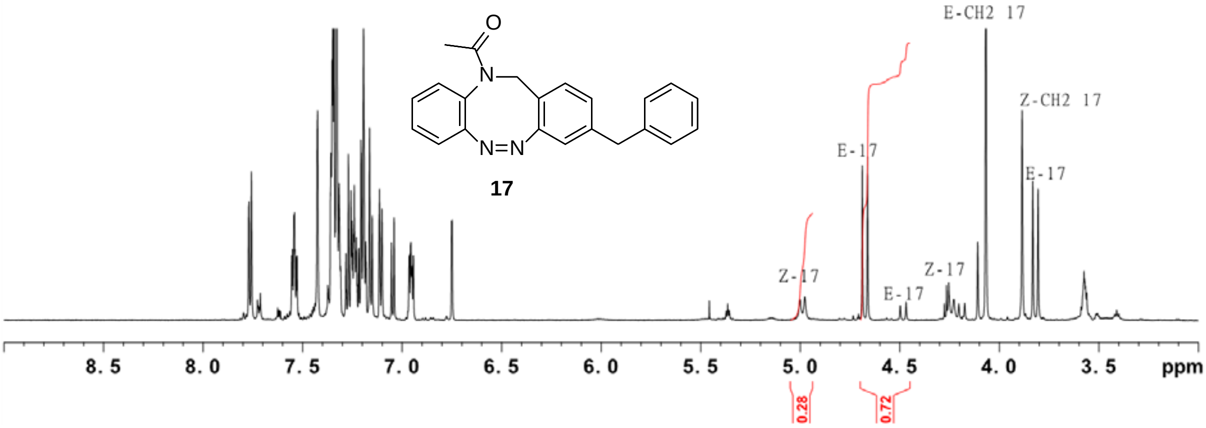


**Figure SIV.24:** $^{1}$H-NMR-spectra of **17** in $CD_3CN$ at 298 K after irradiation with 405 nm.

## IV.11 *tert*-butyl (*Z*)-(11-acetyl-11,12-dihydrodiebenzo[*c*,*g*][1,2,5]triazocin-3-yl)carbamate (19)

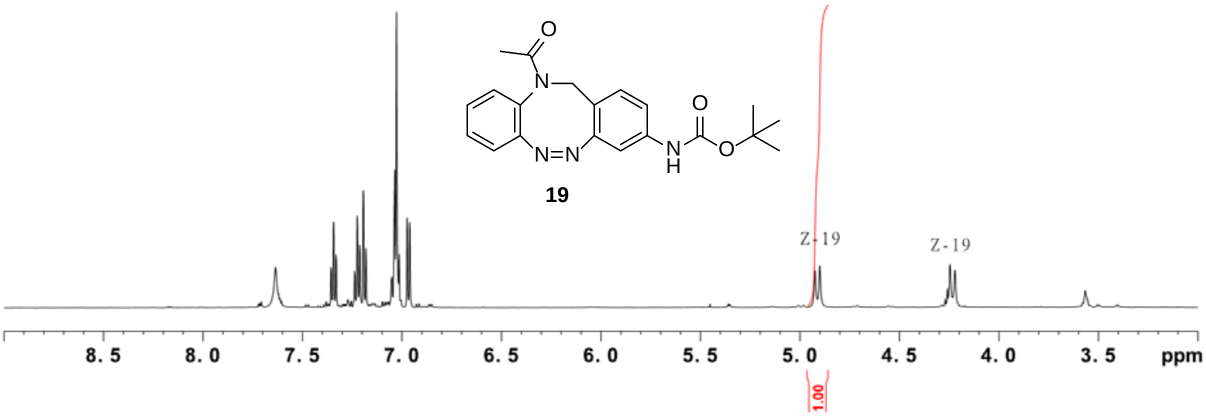


**Figure SIV.25:** $^{1}$H-NMR-spectra of **19** in $CD_3CN$ at 298 K.

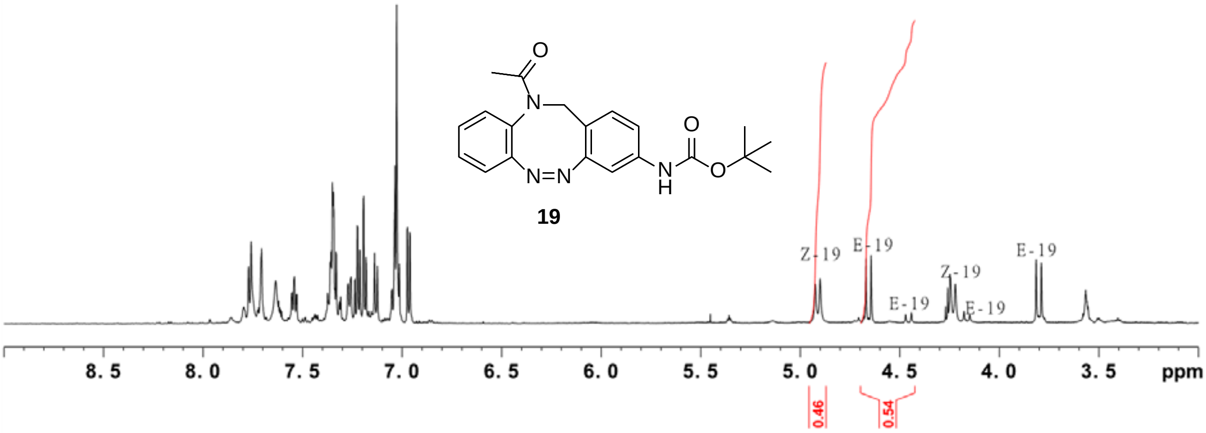


**Figure SIV.26:** $^{1}$H-NMR-spectra of **19** in $CD_3CN$ at 298 K after irradiation with 405 nm.

## IV.12 (*Z*)-1-(3-(diphenylamino)dibenzo[*c*,*g*][1,2,5]triazocin-11(12*H*)-yl)ethan-1-one (20)

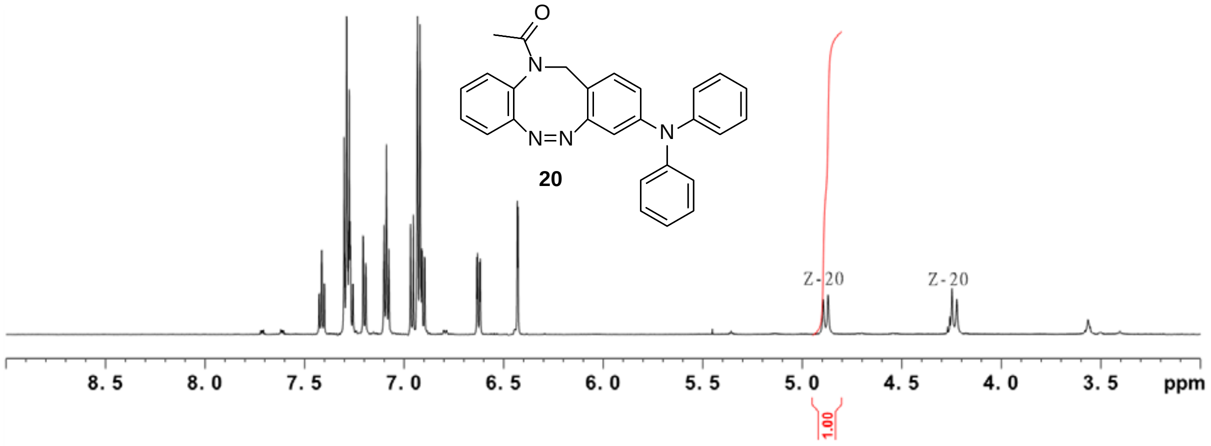


**Figure SIV.27:** $^1$H-NMR-spectra of **20** in $CD_3CN$ at 298 K.

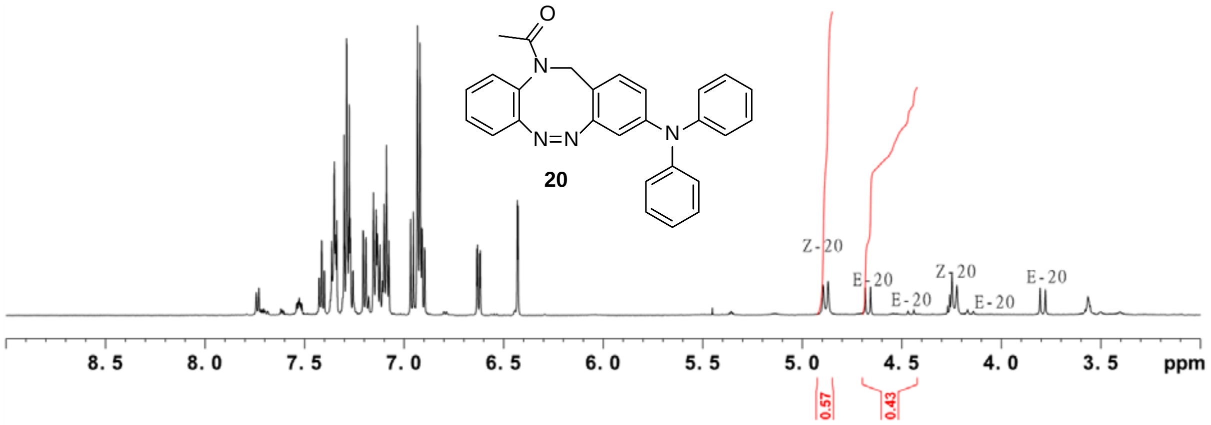


**Figure SIV.28:** $^1$H-NMR-spectra of **20** in $CD_3CN$ at 298 K after irradiation with 405 nm.

## IV.13 (*Z*)-1-(3-aminodibenzo[*c*,*g*][1,2,5]triazocin-11(12*H*)-yl)ethan-1-one (21)

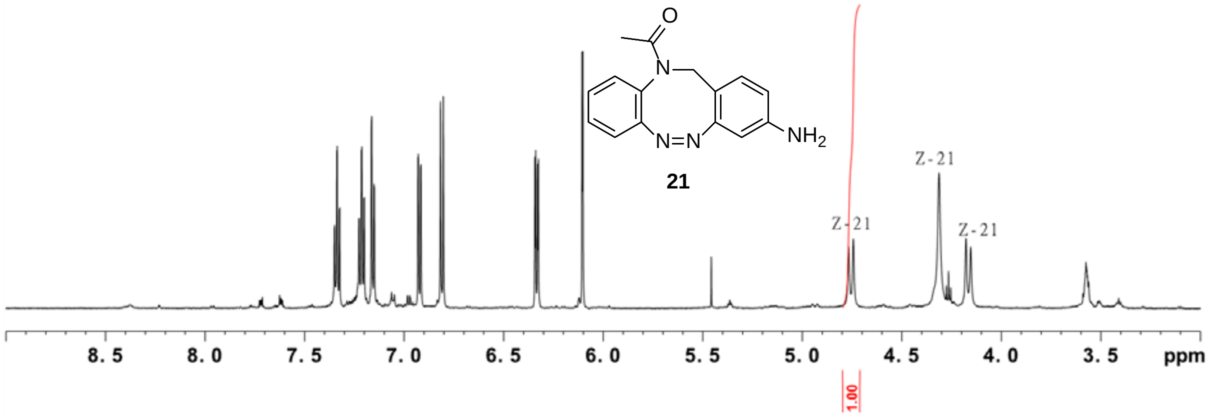


**Figure SIV.29:** $^1$H-NMR-spectra of **21** in $CD_3CN$ at 298 K.

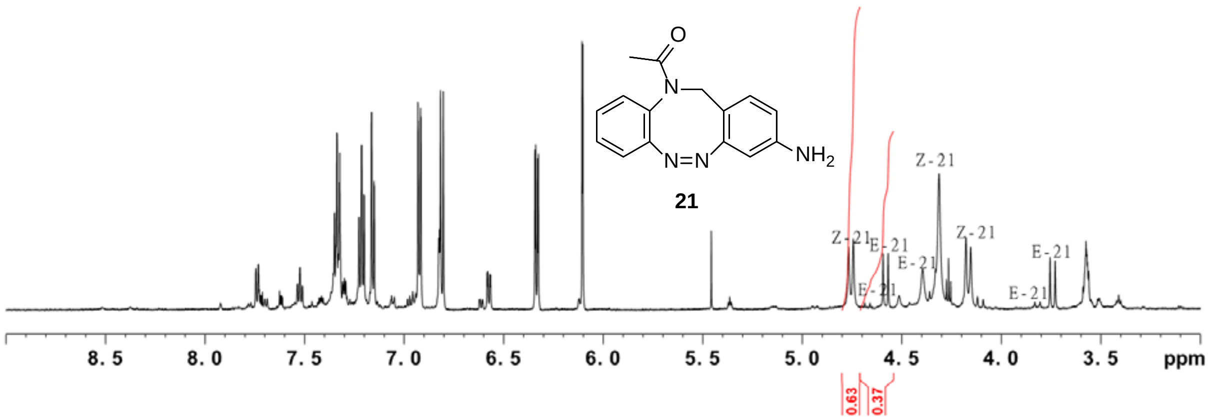


**Figure SIV.30:** $^{1}H$-NMR-spectra of **21** in $CD_3CN$ at 298 K after irradiation with 405 nm.

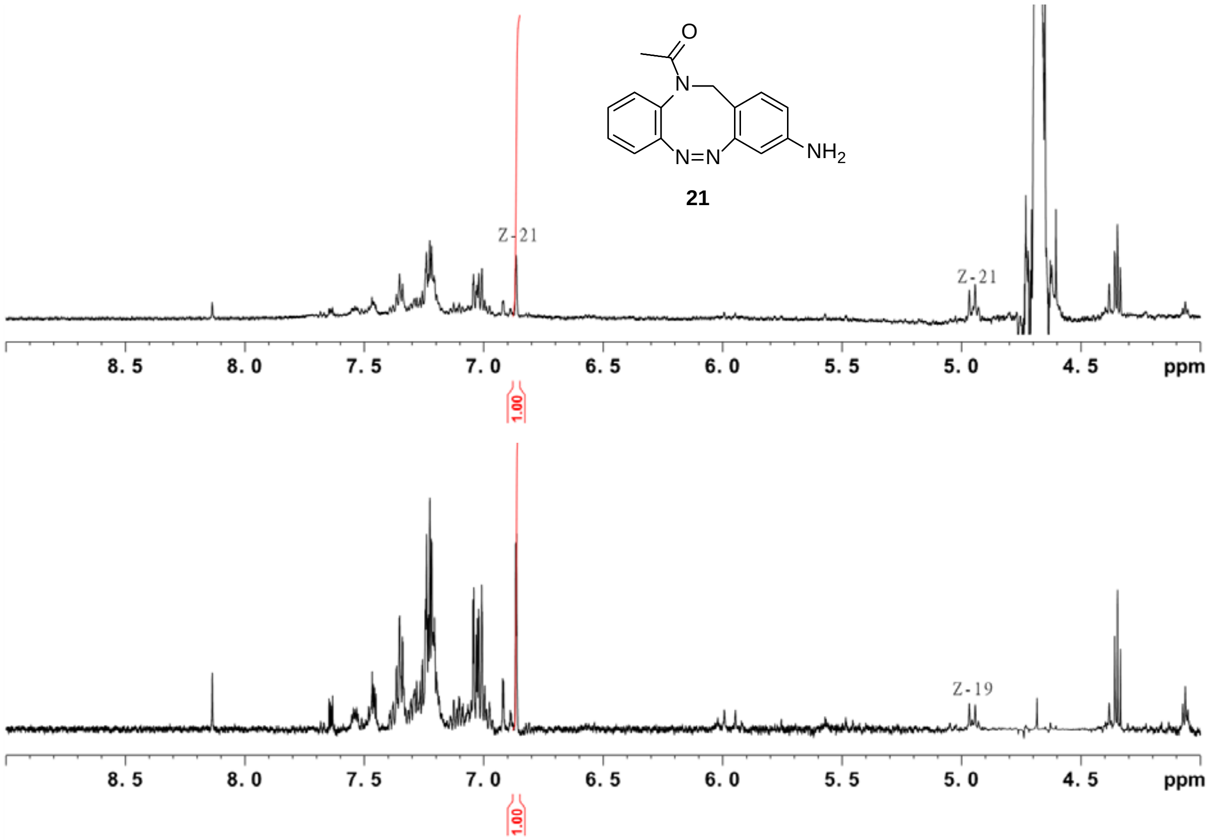


**Figure SIV.33:** $^{1}H$-NMR-spectra of **21** in $D_2O$ pH 3.5 at 298 K (top) and the $^{1}H$-NMR-Spektrum of **23** in $D_2O$ pH 3.5 at 298 K with baseline correction and water suppression performed with MestReNova 14.3.1 NMR analysis Software by Mestrelab (bottom).

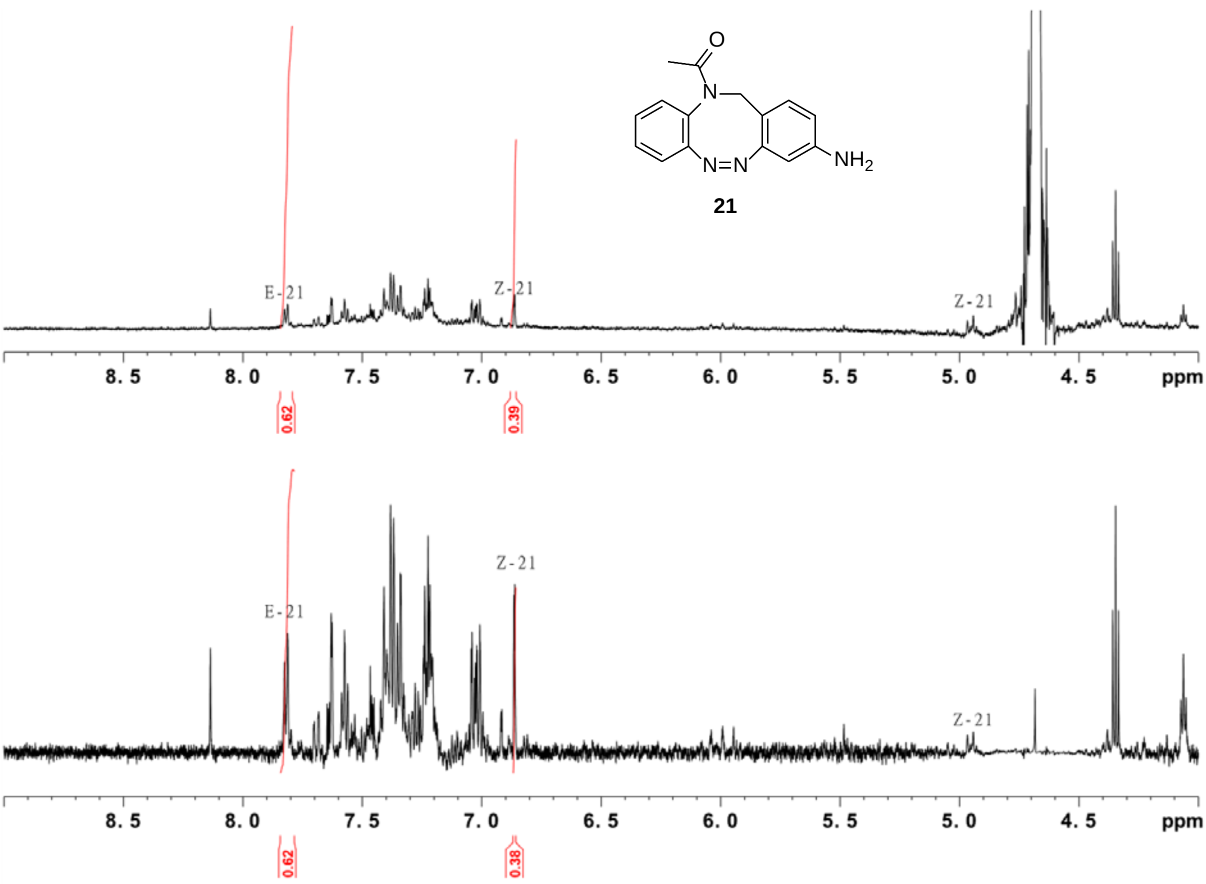


**Figure SIV.34:** $^{1}$H-NMR-spectra of **21** in $D_2O$ pH 3.5 at 298 K after irradiation with 405 nm (top) and the $^{1}$H-NMR-Spektrum of **23** in $D_2O$ pH 3.5 at 298 K after irradiation with 405 nm with baseline correction and water suppression performed with MestReNova 14.3.1 NMR analysis Software by Mestrelab (bottom)

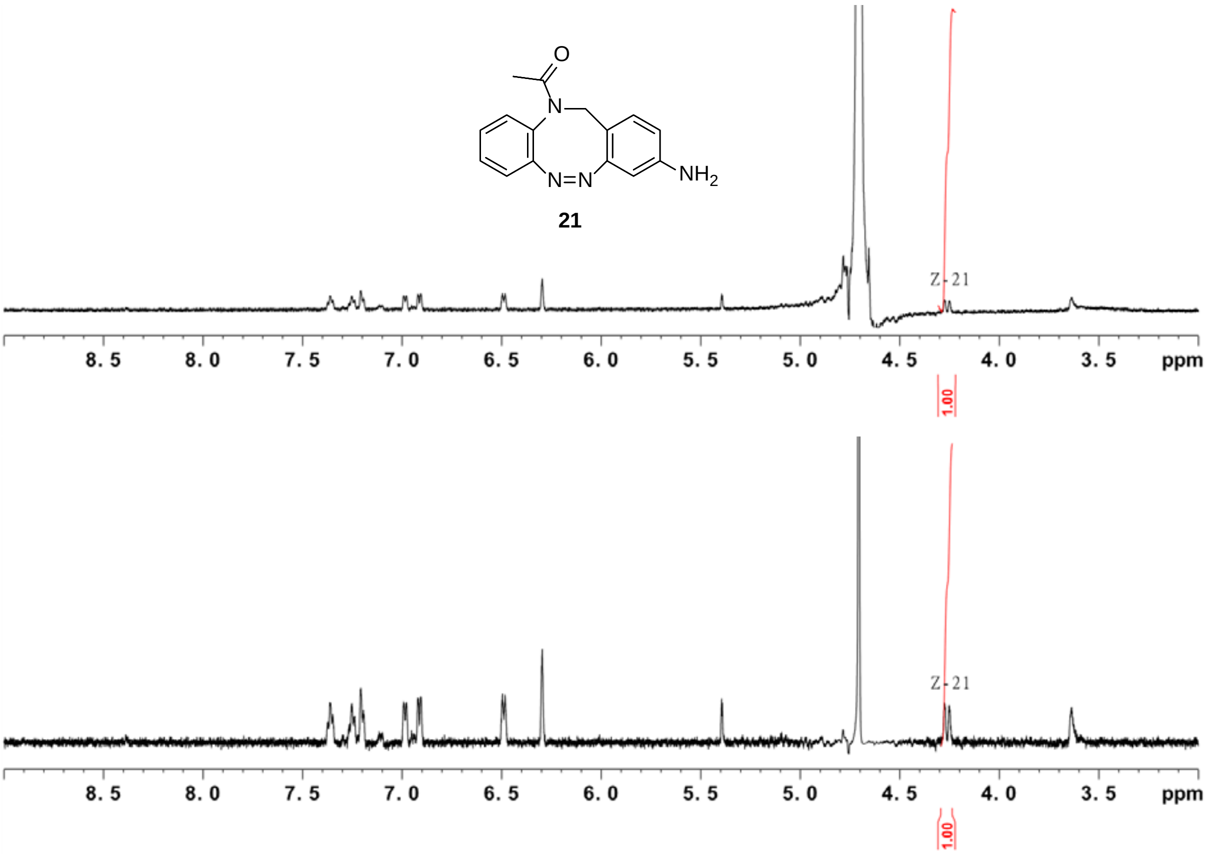

**Figure SIV.35:** $^{1}$H-NMR-spectra of **21** in $D_2O$ PBS buffer solution pH 7.4 at 298 K (top) and the $^{1}$H-NMR-Spektrum of **23** in $D_2O$ PBS buffer solution pH 7.4 at 298 K with baseline correction and water suppression performed with MestReNova 14.3.1 NMR analysis Software by Mestrelab (bottom).

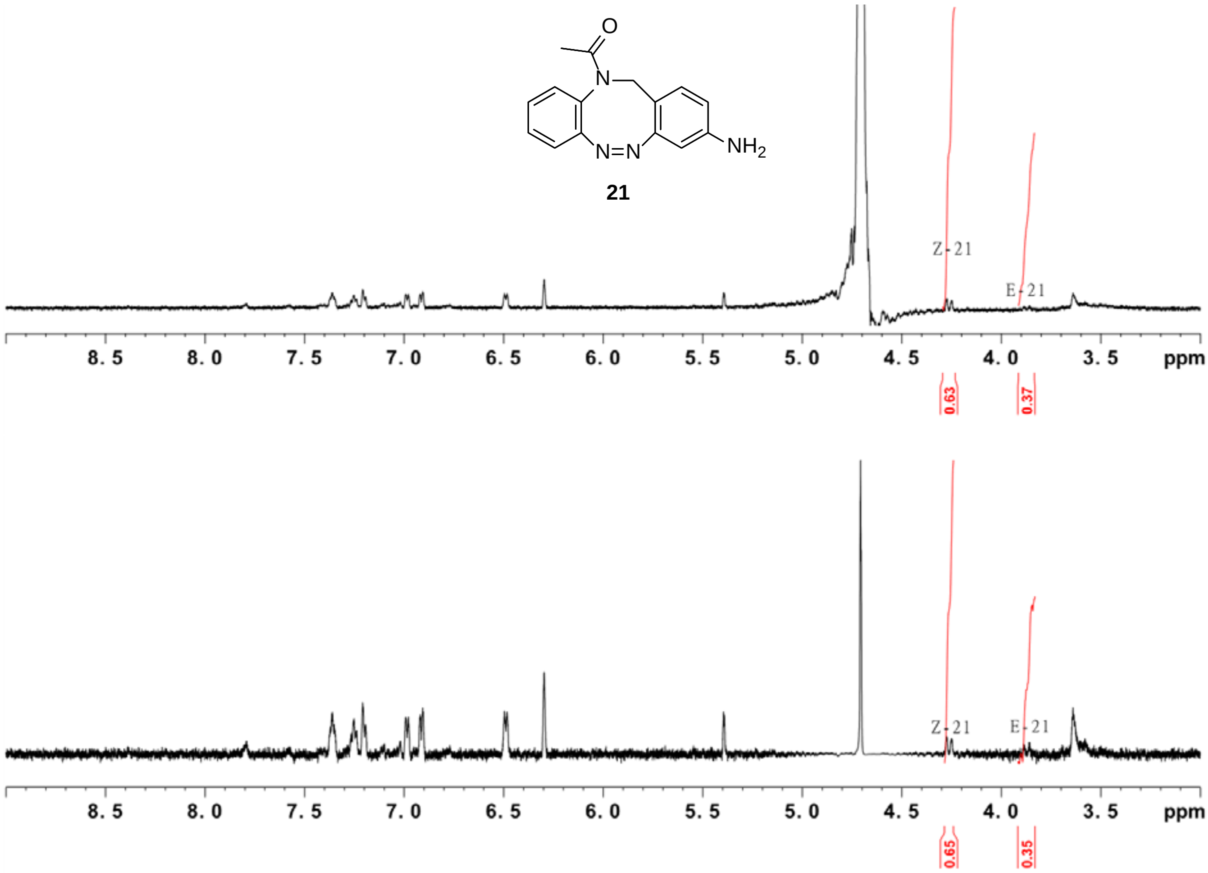


**Figure SIV.36:** $^{1}$H-NMR-spectra of **21** in $D_2O$ PBS buffer solution pH 7.4 at 298 K after irradiation with 405 nm (top) and the $^{1}$H-NMR-Spektrum of **23** in $D_2O$ PBS buffer solution pH 7.4 at 298 K after irradiation with 405 nm with baseline correction and water suppression performed with MestReNova 14.3.1 NMR analysis Software by Mestrelab (bottom).

## IV.14 (*Z*)-11-acetyl-11,12-dihydrodibenzo[*c,g*][1,2,5]triazocine-3-carbonitrile (23)

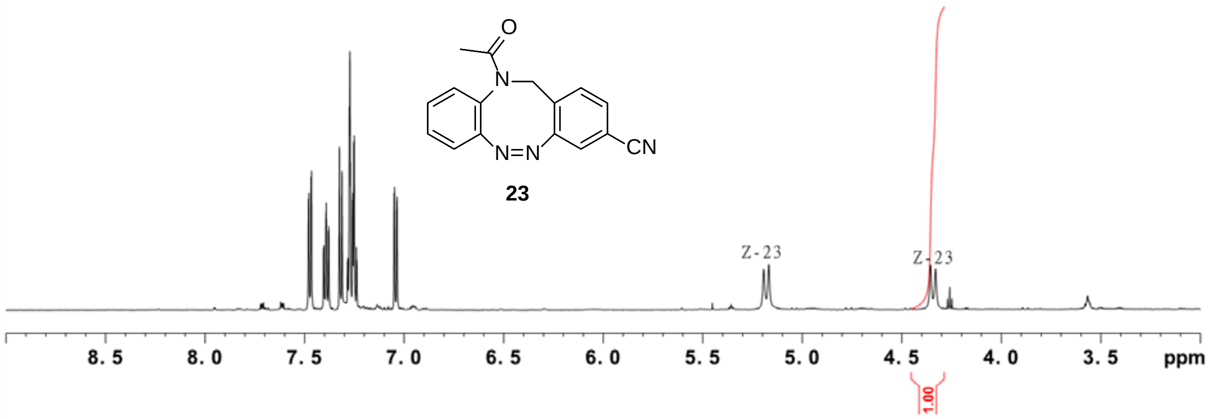


**Figure SIV.37:** $^{1}$H-NMR-spectra of **23** in $CD_3CN$ at 298 K.

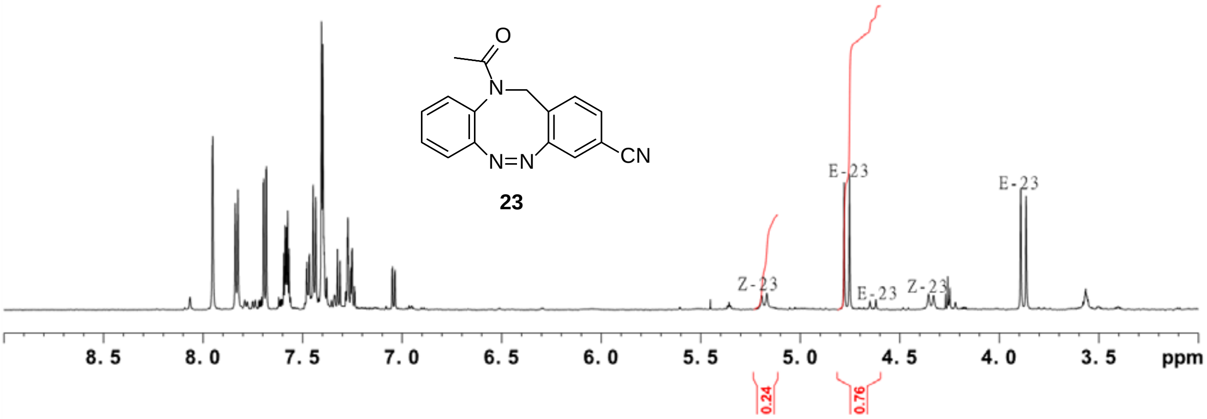


**Figure SIV.38:** $^1$H-NMR-spectra of **23** in $CD_3CN$ at 298 K after irradiation with 405 nm.

# V. X-ray crystallographic data

## Preparation of crystal samples

For crystal growth 10 mg of the given compound were dissolved in 0.2 ml acetone in a small glass vial and a drop of water was added. The vial was closed with a perforated cap and was stored in the dark for 3 weeks for slow evaporation of the solvent. Suitable crystals were selected subsequently.

## General

The data collections were performed with a XtaLAB Synergy, Dualflex, HyPix diffractometer with a micro focus tube using Cu-Kα radiation (λ = 1.54184 Å). The structures were solved with SHELXT [1] and refined with SHELXL-2016 [2] using Least Squares minimisation. All non-hydrogen atoms were refined anisotropic. The C-H H atoms were positioned with idealized geometry, methyl H atoms allowed to rotate but not to tip, and were refined isotropic with $U_{iso}$(H) = 1.2 $U_{eq}$(C) (1.5 for methyl H atoms) using a riding model. In compound **1**, the hydrogen atoms of the methyl group are disordered and were refined in two orientations, each rotated by 60 ° (AFIX 127).
ORTEP plots of all compounds can be found in figure SV.1, SV.2 and SV.3.

CCDC-2329263 (**1**), CCDC-2329261 (**2**), and CCDC- 2329262 (**7**) contain the supplementary crystallographic data for this paper. These data can be obtained free charge from the Cambridge Crystallographic Data Centre via http://www.ccdc.cam.ac.uk/data_request/cif.


1. Sheldrick, G.M. (2015). *Acta Cryst*. A71, 3-8.
2. Sheldrick, G.M. (2015). *Acta Cryst*. C71, 3-8.


**Table SV.1:** Selected crystal data and details of the structure determinations for the diazocine compounds **1**, **2** and **7**.

| | **1** | **2** | **7** |
|---|---|---|---|
| Formula | $C_{15}H_{13}N_3O$ | $C_{15}H_{12}BrN_3O$ | $C_{21}H_{17}N_3O$ |
| MW /g mol$^{-1}$ | 251.28 | 330.19 | 327.38 |
| Crystal system | monoclinic | triclinic | monoclinic |
| Space group | $P2_1/n$ | $P$-1 | $P2_1/n$ |
| $a$ /Å | 8.7563(1) | 7.4471(2) | 11.1465(1) |
| $b$ /Å | 8.7647(1) | 9.2088(2) | 13.0070(1) |
| $c$ /Å | 16.8947(1) | 10.8243(2) | 11.8469(1) |
| $\alpha$ /deg | 90 | 84.031(2) | 90 |
| $\beta$ /deg | 101.974(1) | 77.293(2) | 91.709(1) |
| $\gamma$ /deg | 90 | 81.905(2) | 90 |
| $V$ /Å$^3$ | 1268.40(2) | 714.87(3) | 1716.83(2) |
| $T$ /K | 293 | 293 | 293 |
| $Z$ | 4 | 2 | 4 |
| $D_{calcd}$ /g cm$^{-3}$ | 1.316 | 1.534 | 1.267 |
| $\mu$ /mm$^{-1}$ | 0.687 | 3.911 | 0.634 |
| 2 $\theta_{max}$ /deg | 159.062 | 160.064 | 159.654 |
| Refl. collected | 21811 | 12222 | 18993 |
| Refl. unique | 2704 | 3001 | 3685 |

| | | | |
|---|---|---|---|
| $R_{int}$ | 0.0214 | 0.0263 | 0.0188 |
| Refl. $[F_0 > 4\ \sigma(F_0)]$ | 2592 | 2885 | 3440 |
| Parameters | 174 | 182 | 228 |
| $R_1$ $[F_0 > 4\ \sigma(F_0)]$ | 0.0342 | 0.0381 | 0.0426 |
| $wR_2$ (all data) | 0.0986 | 0.1067 | 0.1230 |
| GOF | 1.054 | 1.035 | 1.058 |
| $\Delta\rho_{max;\ min}$ /e Å$^{-3}$ | 0.19; –0.17 | 0.92; –0.70 | 0.20; –0.17 |

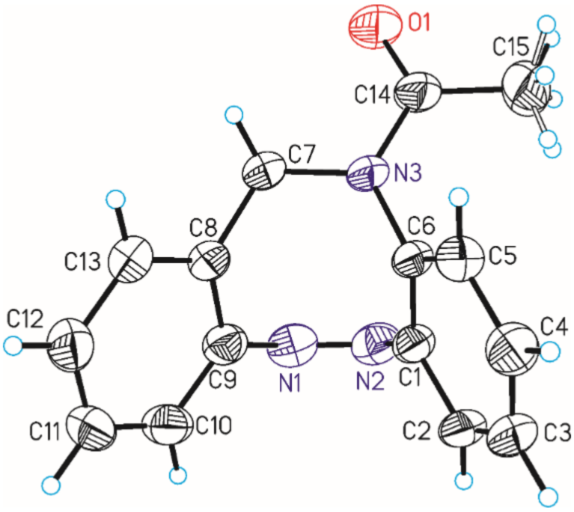


**Figure SV.1:** Crystal structure of *N*-acetyl diazocine **1** with labeling and displacement ellipsoids drawn at the 50% probability level.

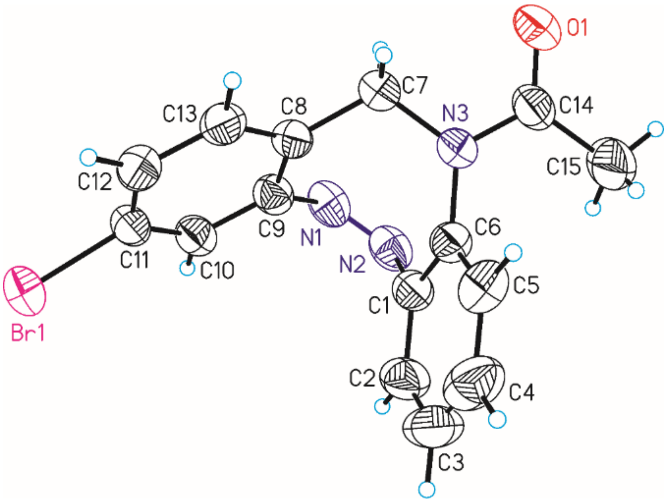


**Figure SV.2:** Crystal structure of bromo-*N*-acetyl diazocine **2** with labeling and displacement ellipsoids drawn at the 50% probability level.

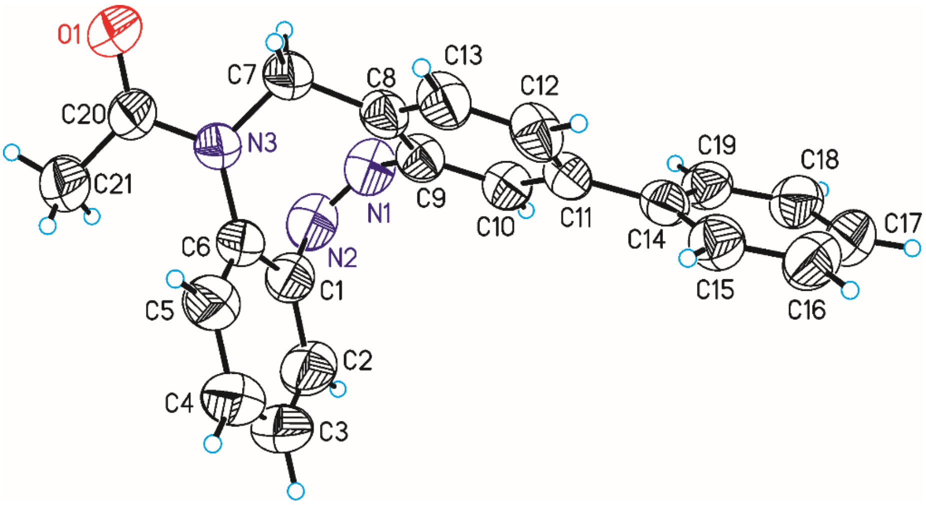


**Figure SV.3:** Crystal structure of phenyl-*N*-acetyl diazocine **7**.

# VI. References


[1] Lentes, P.; Rudtke, J.; Griebenow, T.; Herges, R. *Beilstein J. Org. Chem.* **2021**, *17*, 1503-1508. doi: 10.3762/bjoc.17.107.

[2] Maier, M. S.; Hüll, K.; Reynders, M.; Matsuura, B. S.; Leippe, P.; Ko, T.; Schäffer, L.; Trauner, D. *J. Am. Chem. Soc.* **2019**, *141*, 17295-17304. doi: 10.1021/jacs.9b08794.

[3] Denmark, S. E.; Butler, C. R. *Org. Lett.* **2006**, *8*, 63-66. doi: 10.1021/ol052517r.

[4] M. Rohan, A. K. Sarmah, *Sci. Total Environ*. **2023**, *893*, 164773.
